# Vagus Nerve Stimulation Attenuates Acute Skeletal Muscle Injury Induced by Hepatic Ischemia/Reperfusion Injury in Rats

**DOI:** 10.3389/fphar.2021.756997

**Published:** 2022-01-03

**Authors:** Ying Xin, Yifeng Zhang, Simin Deng, Xinqun Hu

**Affiliations:** Department of Cardiovascular Medicine, The Second Xiangya Hospital, Central South University, Changsha, China

**Keywords:** ischemia reperfusion, remote organs, vagus nerve stimulation, hepatic injury, skeletal muscle injury

## Abstract

Vagus nerve stimulation (VNS) has a protective effect on distal organ injury after ischemia/reperfusion (I/R) injury. We aimed to investigate the protective efficacy of VNS on hepatic I/R injury-induced acute skeletal muscle injury and explore its underlying mechanisms. To test this hypothesis, male Sprague-Dawley rats were randomly divided into three groups: sham group (sham operation, n = 6); I/R group (hepatic I/R with sham VNS, n = 6); and VNS group (hepatic I/R with VNS, n = 6). A hepatic I/R injury model was prepared by inducing hepatic ischemia for 1 h (70%) followed by hepatic reperfusion for 6 h. VNS was performed during the entire hepatic I/R process. Tissue and blood samples were collected at the end of the experiment for biochemical assays, molecular biological preparations, and histological examination. Our results showed that throughout the hepatic I/R process, VNS significantly reduced inflammation, oxidative stress, and apoptosis, while significantly increasing the protein levels of silent information regulator 1 (SIRT1) and decreasing the levels of acetylated forkhead box O1 and Ac-p53, in the skeletal muscle. These data suggest that VNS can alleviate hepatic I/R injury-induced acute skeletal muscle injury by suppressing inflammation, oxidative stress, and apoptosis, potentially via the SIRT1 pathway.

## Introduction

For Hepatic ischemia/reperfusion (I/R) injury during liver surgery, such as liver transplantation and liver resection, is an important pathophysiological process leading to liver injury, which accelerates the progression of diseases such as acute liver injury and liver failure (Al-Saeedi et al., 2018; Ni et al., 2019). The exposure of a single organ to ischemia and reperfusion may subsequently cause a cascade of inflammation and oxidative stress in other organs, damage tissues/organs, and eventually lead to multifunctional failure, which is related to the high mortality rate of hepatic I/R injury (Eltzschig and Eckle, 2011; König et al., 2016). Because the body’s sensitivity to ischemia varies from organ to organ, skeletal muscle is the least tolerant of all tissues in the limbs (Blaisdell, 2002). Skeletal muscles account for approximately 40% of body weight in lean men and women, making up the largest organ in non-obese people (Guridi et al., 2015). It is also an important metabolic organ of the body, which is believed to have the ability to produce hundreds of secretory factors, which can then be released into the circulation, directly or indirectly affecting the functions of other organs, establish connections with other tissues and organs, participate in the regulation of oxidative stress, and play a role in anti-I/R (Pedersen and Febbraio, 2012; Szabó et al., 2020). As a distant organ, skeletal muscle is susceptible to hepatic I/R injury, which leads to acute skeletal muscle injury and accelerates the progression of disease (Weinbroum et al., 1995; Chang et al., 2015). Unfortunately, the current clinical treatment for remote organ damage caused by I/R injury is mainly symptomatic supportive treatment, and the effect is unsatisfactory. Therefore, it is necessary to develop effective adjuvant therapy to alleviate skeletal muscle injury caused by acute liver injury.

Vagus nerve stimulation (VNS) therapy is considered a safe and effective method for treating a variety of diseases. VNS has been shown to be effective in epilepsy, refractory depression, inflammatory bowel disease, and septic shock (Chadwick, 2001; Wu et al., 2007; Furmaga et al., 2011; Meroni et al., 2019). Meanwhile, VNS has also shown promising therapeutic effects in the I/R injury of some tissues and organs, such as the liver and skeletal muscle (Zhang et al., 2019a; Zhang et al., 2019b). Further, other studies have shown that VNS can alleviate the injury of distant organs caused by I/R injury (Jing et al., 2014; Lauz Medeiros et al., 2016; Lai et al., 2019; Liu et al., 2020). Therefore, we speculate that VNS can treat skeletal muscle injury caused by hepatic I/R. However, it is not yet clear whether VNS relieves the skeletal muscle damage mechanism by hepatic I/R injury. Therefore, we conducted this study using a rat model of hepatic I/R injury, aiming to explore whether VNS has a protective effect on skeletal muscle injury caused by hepatic I/R and its possible mechanism.

## Materials and Methods

### Animals and Experimental Groups

**FIGURE 1:**
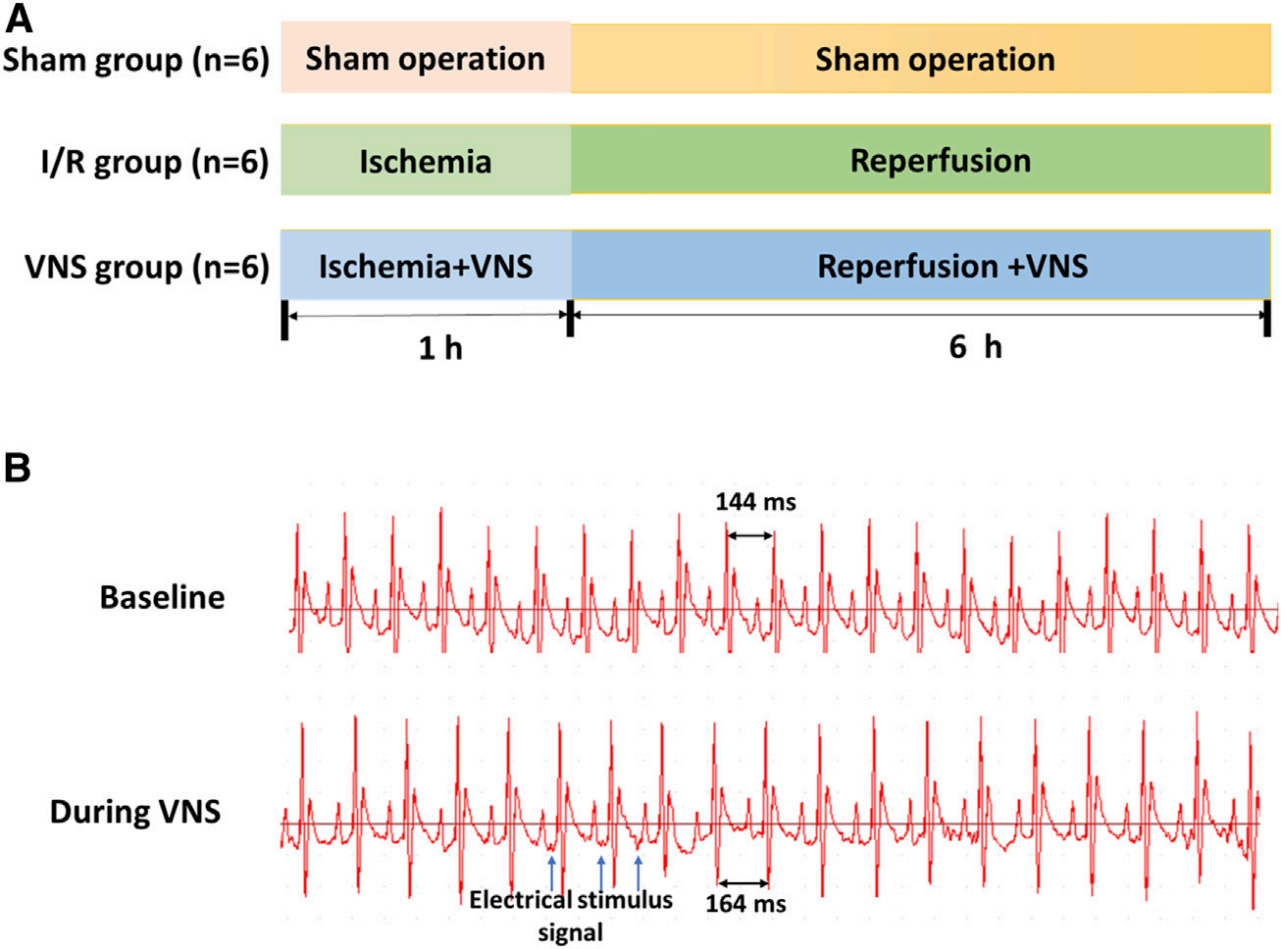
Experimental protocol **(A)** and sinus heart rate changes before and after VNS **(B)**. Sham: sham operation; I/R: ischemia-reperfusion; VNS: vagus nerve stimulation.

All experimental procedures were approved by the Animal Care and Use Committee of the Second Xiangya Hospital of Central South University and strictly conformed to guidelines of the Guide for the Care and Use of Laboratory Animals by the United States National Institutes of Health (NIH Publication No. 85–23, revised 1996). Healthy male Sprague-Dawley rats (6-8w, each weighing 280–350 g) were used in this study. Eighteen rats were randomly allocated to one of three groups that received different treatments: sham group (sham operation; n = 6), I/R group (hepatic I/R with sham VNS, n = 6), and VNS group (hepatic I/R + VNS, n = 6). Rats were given food and water ad libitum in their cages, and were maintained on an alternating 12-h light/dark cycle at a constant temperature (24 °C). All experimental animals were fasted for 8 h before the surgery. A flow chart of the experimental design is shown in Figure 1 A. During the experiment, a surface electrocardiogram (ECG) was recorded using a TECHMAN biological signal acquisition system (BL-420F, Chengdu City, China).

### Acute Hepatic I/R Injury Model

An acute segmental (70%) hepatic I/R injury model was employed in this experiment, as previously described (Zhang et al., 2019b). All rats were anesthetized with 1% pentobarbital sodium (40 mg/kg body weight) by intraperitoneal injection before the operation. Then, a midline abdominal incision was made, and the arterial and portal veins of the left and middle lobes of the liver were blocked using noninvasive vascular forceps. The vascular forceps were removed 1 h later, and liver reperfusion was performed for 6 h. A rectal probe was used to monitor body temperature throughout the procedure, and a heating pad was used to maintain the body temperature at 37°C. At the end of the experiment, rats were anesthetized by inhalation of methoxyflurane and killed by exsanguination.

### Vagus Nerve Stimulation

In this study, the left cervical vagus nerve trunk was selected as the stimulation target, the vagus nerve was carefully separated from the surrounding connective tissue and stimulated with a bipolar silver electrode on a stimulator (SDZ-IIB, Hwato, Suzhou City, China). Subsequently, continuous low-frequency stimulation (LFS, 20 Hz, 0.2 ms in duration, square waves) was performed. The intensity of stimulation should reach a 10% reduction in the sinus rate and be adjusted hourly (Zhang et al., 2019b) (Figure 1B).

### Blood and Tissue Samples Collection

At the end of the experiment, blood samples were collected from the inferior vena cava. Blood samples were centrifuged at 3000 rpm for 15 min to obtain the serum. Tissue specimens were collected from the left gastrocnemius muscles. Each tissue specimen was divided into three uneven sections. The main components were used for histological analysis. The two smaller sections were frozen in liquid nitrogen for biochemical and molecular analyses. All blood and tissue samples for biochemical and molecular biological analyses were stored at −80°C until use.

### Histological Examination

At the end of the experiment, the skeletal muscle tissue samples collected and were immersed in a 10% paraformaldehyde solution for 48 h, dehydrated in ethanol, and rinsed with xylene. The tissues were then embedded in paraffin. The sections were cut at 4 μm and stained with hematoxylin and eosin (H&E). The morphological characteristics of the skeletal muscle tissue were observed under a light microscope. According to previous studies, the score of tissue damage was as follows: muscle fiber disorder and stripe loss:0, normal; 1, mild; 2, moderate; 3, severe), and inflammatory cell infiltration:0, normal; 1, mild; 2, moderate; 3, severe) (Erkanli et al., 2005). Apoptosis of skeletal muscle cells was detected by terminal deoxynucleotidyl transferase-mediated deoxyuridine triphosphate (dUTP) nick end-labeling (TUNEL) staining. Skeletal muscle tissue sections were dewaxed in xylene and then hydrated in fractional ethanol. After protease K treatment, sections were rinsed with phosphate buffered saline (PBS) and incubated with TUNEL reagent at 37°C, in the dark, for 60 min. After washing three times with PBS, sections were treated with 4, 6-diamidino-2-phennylindole (DAPI). The stained sections were analyzed using a fluorescence microscope (Nikon DS-U3, Tokyo, Japan). Cells with irregular green granules in the nucleus were TUNEL-positive cells, and cell death was observed and measured in three random regions of each muscle cross-section and averaged. The rate of TUNEL-positive cells was recorded.

### Analysis of Serum Creatine Kinase (CK), muscle-type creatine kinase (MM-CK) Lactate Dehydrogenase (LDH), Alanine Aminotransferase (ALT) and Aspartate Aminotransferase (AST) in Serum Levels

Serum CK, MM -CK and LDH levels, which reflect skeletal muscle injury severity, were assessed using commercial kits (ELK Biotechnology, Wuhan City, China) according to the manufacturer’s protocol. ALT and AST levels were measured by automatic biochemical analyzer (Chemray 800, Shenzhen Redu Life Technology).

### Determination of Inflammatory Cytokines

The hepatic I/R injury-induced expression of inflammatory cytokines in skeletal tissues, specifically tumor necrosis factor alpha (TNF-α), interleukin 6 (IL-6), and interleukin 1-beta (IL-1β), was determined by ELISA (ELK Biotechnology, Wuhan City, China) according to the manufacturer’s instructions. In addition, qRT-PCR was used to detect the mRNA levels of inflammatory factors at the transcriptional level. According to the manufacturer’s protocol, total RNA was extracted from frozen skeletal muscle tissue using Trizol reagent (Invitrogen Life Technologies). First strand cDNA was synthesized using PrimeScript™ RT Reagent Kit with gDNA Eraser (TaKaRa Bio Inc.). Candidate gene expression levels were measured using an RT-PCR thermocycler (StepOne™ Life Technologies) with the following specific primers: IL-1β, forward: 5′-GTG​GCA​GCT​ACC​TAT​GTC​TTG​C-3′, reverse: 5′-CCA​CTT​GTT​GGC​TTA​TGT​TCT​GT-3′; IL-6, forward: 5′-GCC​AGA​GTC​ATT​CAG​AGC​AAT-3′, reverse: 5′-CTT​GGT​CCT​TAG​CCA​CTC​CT-3′; and TNF-α, forward: 5′-CAC​CAC​GCT​CTT​CTG​TCT​ACT​G-3′, reverse: 5′-GCT​ACG​GGC​TTG​TCA​CTC​G-3′. The mRNA level for each target gene was calculatedusing the Delta-Delta-CT method and normalized to the β-actin mRNA level from the same sample. The primers for β-actin were as follows: forward: 5′-CGT​TGA​CAT​CCG​TAA​AGA​CCT​C-3′, reverse: 5′-TAG​GAG​CCA​GGG​CAG​TAA​TCT-3′.

### Measurements of Oxidative Stress in Skeletal Muscle Tissues After Hepatic I/R Injury

The levels of malondialdehyde (MDA), myeloperoxidase (MPO), glutathione (GSH), and superoxide dismutase (SOD) in skeletal muscle tissue samples from each group were detected using different assay kits (Nanjing Jiancheng Bioengineering Institute, Nanjing City, China). The total oxidative status (TOS) and total antioxidant capacity (T-AOC) in skeletal muscle tissues were measured using commercial kits (Nanjing Jiancheng Bioengineering Institute, Nanjing City, China). The oxidative stress index (OSI)was used to reflect the degree of oxidative stress. The formula used to calculate the OSI value is as follows: OSI = TOS (μmol/gprot)/T-AOC (mmol/gprot) ×100.

### Western Blot Analysis

In skeletal muscle tissues, the protein expression levels of Bcl-2, Bax, cleaved PARP, and cleaved caspase 3, are often used as indicators of apoptosis. Silent information regulator 1 (SIRT1), acetylated forkhead box O1(Ac-Foxo1), Ac-p53, Bcl2, Bax, cleaved PARP, cleaved caspase 3, and β-tubulin expression was measured by western blot. Frozen skeletal muscle tissue was cleaved with a lyse solution. After homogenization of the skeletal muscle tissues, the supernatant was collected, and the protein concentration was determined using a BCA Protein Assay Kit. Equal amounts of denatured protein solution were separated by SDS-PAGE and then transferred to a Polyvinylidene fluoride (PVDF) membrane. The membranes were blocked with 5% skim milk at 25°C for 1 h and then incubated with rabbit anti-β-tubulin antibody (1:2,000 dilution, Abcam,Cambridge, United Kingdom), rabbit anti-Bax antibody (1:2,000 dilution, CST, Boston, United States), rabbit anti-Bcl-2 antibody (1:2,000 dilution, Abcam, Cambridge, United Kingdom), rabbit anti-cleaved caspase3 antibody (1:500 dilution, Affbiotech, Shanghai City, China), rabbit anti-cleaved PARP antibody (1:500 dilution, CST, Boston, United States), rabbit anti-silent information regulator 1(SIRT1, 1:1,000 dilution, CST, Boston, United States), rabbit anti-acetylated forkhead box protein O1 (Ac-FoxO1, 1:500 dilution, Affbiotech, Shanghai City, China), rabbit anti-acetylated P53 (Ac-P53, 1:500 dilution, Abcam, Cambridge, United Kingdom) overnight at 4°C. The PVDF membrane was washed in Tris-buffered saline containing Tween (TBST) and incubated with horseradish peroxidase -goat anti-rabbit secondary antibody for 1 h at room temperature. Finally, the relative protein expression of the same samples was normalized to β-tubulin and quantified using AlphaEase FC image analysis software (Alpha Innotech, Alameda, California, United States).

### Statistical Analysis

All continuous variables were expressed as mean ± standard deviation. GraphPad Prism 7.0 (GraphPad Software, Inc. San Diego, CA) was used for the statistical analysis and graphing of results. One-way analysis of variance was used for between-group differences, and a two-tailed *p* ≤ 0.05, was considered statistically significant.

## Results

### Vagus Nerve Stimulation Significantly Ameliorated Acute Skeletal Muscle Injury After Hepatic I/R Injury

**FIGURE 2:**
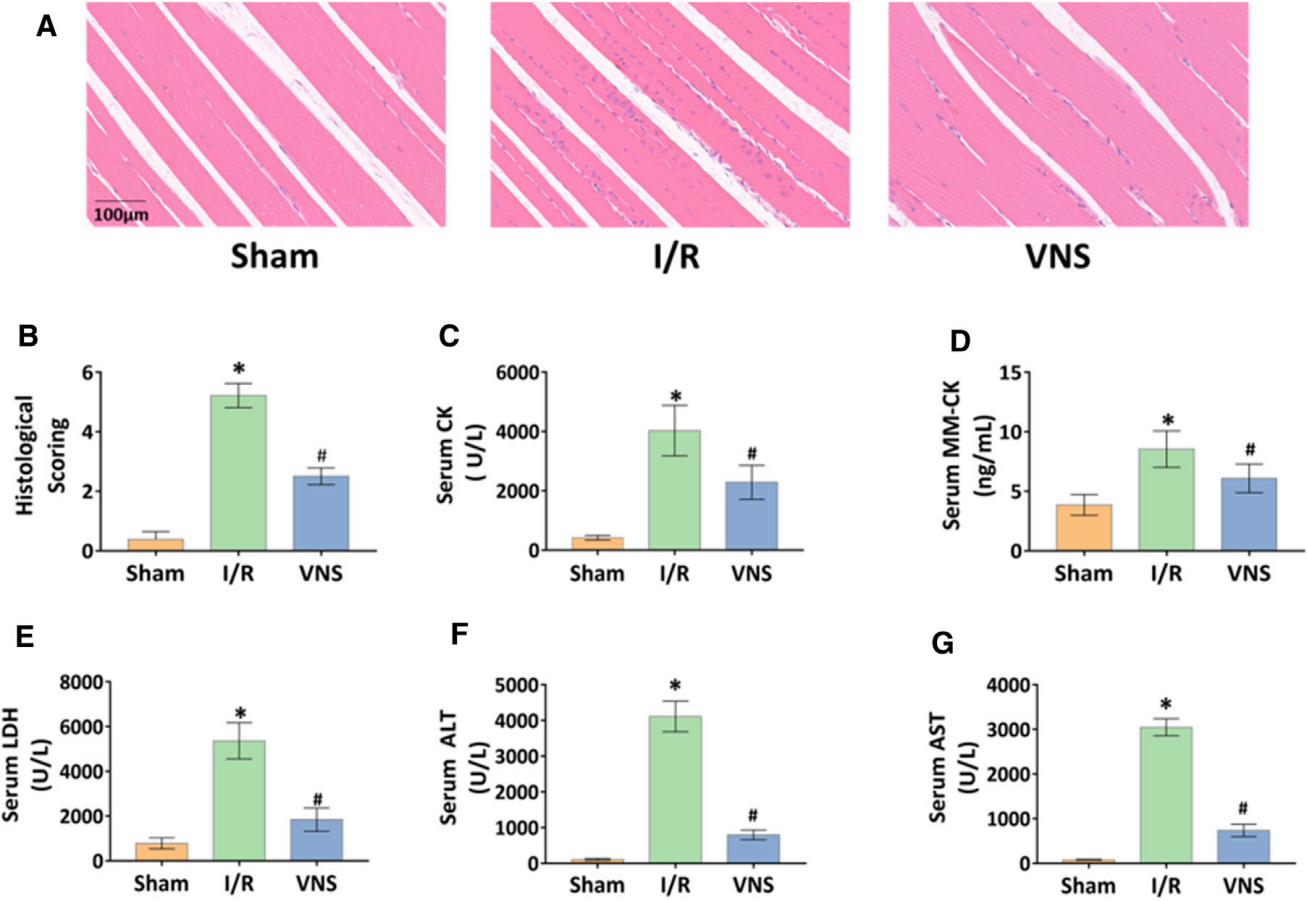
VNS alleviates skeletal muscle injury. **(A)** Representative H&E staining images of skeletal muscle tissues in the three groups are shown. **(B)** skeletal muscle injury histological scores are shown. Levels of serum **(C)** CK, **(D)** MM-CK, **(E)** LDH, **(F)** ALTand **(G)** AST are presented. *p < 0.05 vs the Sham group; #*p* < 0.05 vs the I/R group. CK: creatine kinase; MM-CK: muscle-type creatine kinase; LDH: lactate dehydrogenase; ALT: alanine aminotransferase; AST:aspartate aminotransferase; VNS: vagus nerve stimulation.

Skeletal muscle injury was determined by histological examination and corresponding blood tests. H&E staining revealed that the skeletal muscles in the I/R group showed significant muscle fiber disorder, stripe loss, and inflammatory cell infiltration compared with those in the sham group (Figure 2A). Compared with the I/R group, the severity of skeletal muscle injury reduced in the VNS group (Figure 2A). Tissue damage scores of the I/R group were higher than those of the sham group, but the scores of the VNS group were lower than those of the I/R group (Figure 2B). Serum examination results were consistent with those of the histological examination. Serum levels of CK, MM-CK, and LDH in the I/R group were significantly higher than those in the sham group (Figure 2C–E). Serum levels of CK, MM-CK, and LDH significantly reduced in the VNS group compared with those in the I/R group (Figure 2C–E). These results indicate that VNS may have a protective effect against skeletal muscle injury after hepatic I/R injury.

### Vagus Nerve Stimulation Significantly Suppressed the Inflammatory Effects of Hepatic I/R Injury in Skeletal Muscle Tissues

**FIGURE 3:**
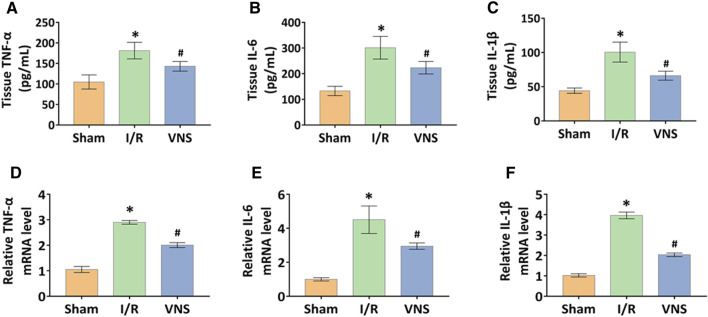
VNS mitigates inflammation in the skeletal muscle after hepatic I/R injury. The effect of VNS in the skeletal muscle tissues levels of **(A)** TNF-α, **(B)** IL-6, and **(C)** IL-1β is shown. Relative mRNA levels of **(D)** TNF-α, **(E)** IL-6, and **(F)** IL-1β are shown. Data are presented as mean ± standard deviation. *p < 0.05 versus the Sham group; #*p* < 0.05 versus the I/R group. TNF-α: tumor necrosis factor alpha; IL-6: interleukin 6; IL-1β: interleukin1-beta; VNS: vagus nerve stimulation.

The levels of inflammatory factors in skeletal muscle tissue after hepatic I/R injury were measured in order to determine whether VNS can regulate the skeletal muscle inflammatory response. Compared with the sham operation group, TNF-α, IL-6 and IL-1β levels were significantly increased not only in protein expression level but also in transcription level in the I/R group (Figure 3A–F ). In contrast, VNS treatment significantly reduced the levels of TNF-α, IL-6, and IL-1β in skeletal muscle tissue compared to the I/R group (Figure 3A–F).

### Vagus Nerve Stimulation Significantly Inhibits Oxidative Stress and Improved Redox Status in Skeletal Muscle After Hepatic I/R Injury

**FIGURE 4:**
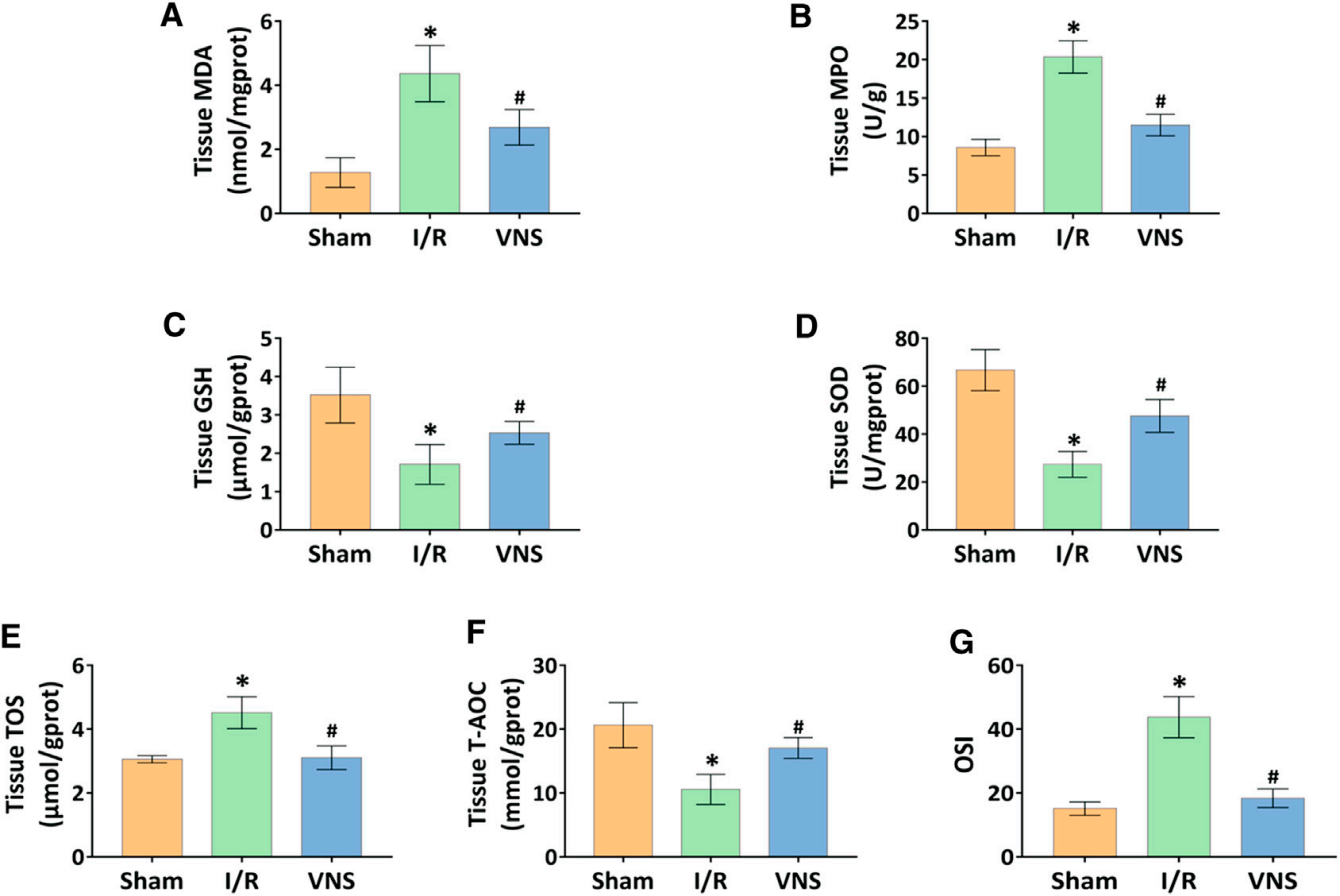
VNS attenuates oxidative stress and improves redox status in the skeletal muscle after hepatic I/R injury. The effect of VNS on the levels of **(A)** MDA and **(C)** GSH in skeletal muscle tissues is shown. The effect of VNS on the activity of **(B)** MPO and **(D)** SOD in skeletal muscle tissues is shown. The effect of VNS on the levels of **(E)** TOS and **(F)** T-AOC in skeletal muscle tissues is shown. **(G)** The OSI values are shown. Data are expressed as mean ± standard deviation. *p < 0.05 versus the Sham group; #*p* < 0.05 versus the I/R group. MDA: myeloperoxidase; MPO: malondialdehyde; GSH: glutathione; SOD: superoxide dismutase; TOS: total oxidant status; T-AOC: total antioxidant capacity; OSI: oxidative stress index; VNS: vagus nerve stimulation.

Acute reperfusion leads to oxidative stress and overproduction of reactive oxygen species (ROS). The expression of MDA and MPO was increased, suggesting and increase in oxidative stress. The expression levels of GSH, SOD, and other antioxidant enzymes decreased. The levels of MDA and GSH, as well as the activity of MPO and SOD in skeletal muscle tissue, were measured to evaluate the relative oxidative stress. Compared with the sham group, MDA levels and MPO activity were significantly increased in the I/R group (Figures 4A,B). However, VNS treatment significantly reversed these changes in skeletal muscle injury due to hepatic I/R injury, compared to the I/R group (Figures 4A,B). Compared with the sham group, GSH levels and SOD activity significantly decreased in the I/R group (Figures 4C,D). Interestingly, VNS treatment significantly reversed this decline compared to the I/R group (Figures 4C,D). To further assess redox status, TOS and T-AOC levels in the skeletal muscle were measured. Compared with the sham group, the level of TOS was significantly increased, and the level of TAS was significantly decreased, in the I/R group (Figures 4E,F). VNS significantly reversed these changes compared to the I/R group (Figures 4E,F). Meanwhile, the OSI value of the I/R group was higher than that of the sham group (Figure 4G). The OSI value in the VNS group was lower than that of the I/R group (Figure 4G). These data suggest that VNS significantly alleviates oxidative stress in skeletal muscle after hepatic I/R injury and may have a protective effect.

### Vagus Nerve Stimulation Significantly Reduced Skeletal Muscle Apoptosis After Hepatic I/R Injury

**FIGURE 5:**
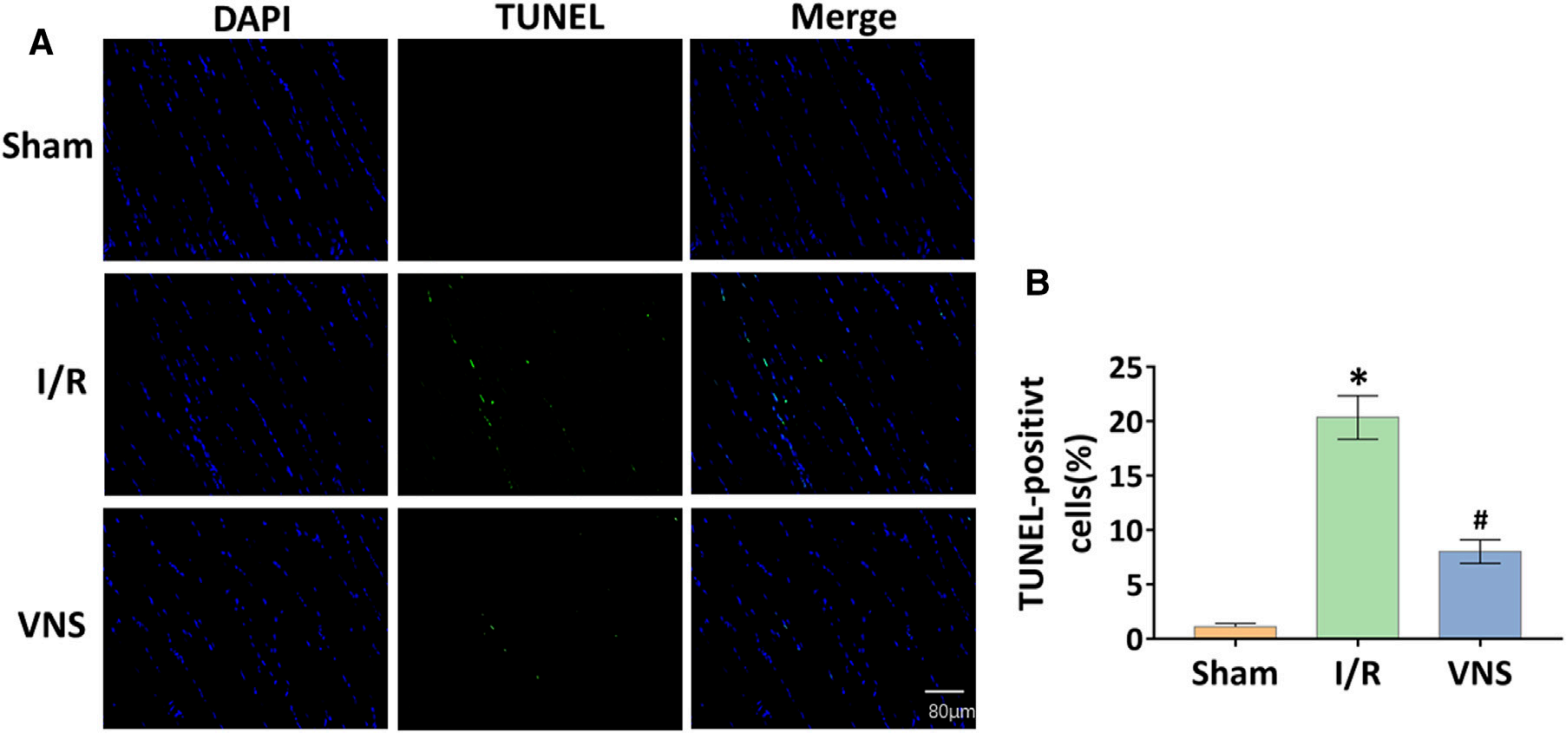
VNS reduces cell apoptosis in skeletal muscle after hepatic I/R injury. **(A)** Representative micrographs of immunofluorescence staining for DAPI (blue) and TUNEL (green) in skeletal muscle cell nuclei from the three groups are shown. **(B)** Quantification of skeletal muscle cell apoptosis from the three groups is shown. *p < 0.05 vs the Sham group; #*p* < 0.05 vs the I/R group.

**FIGURE 6:**
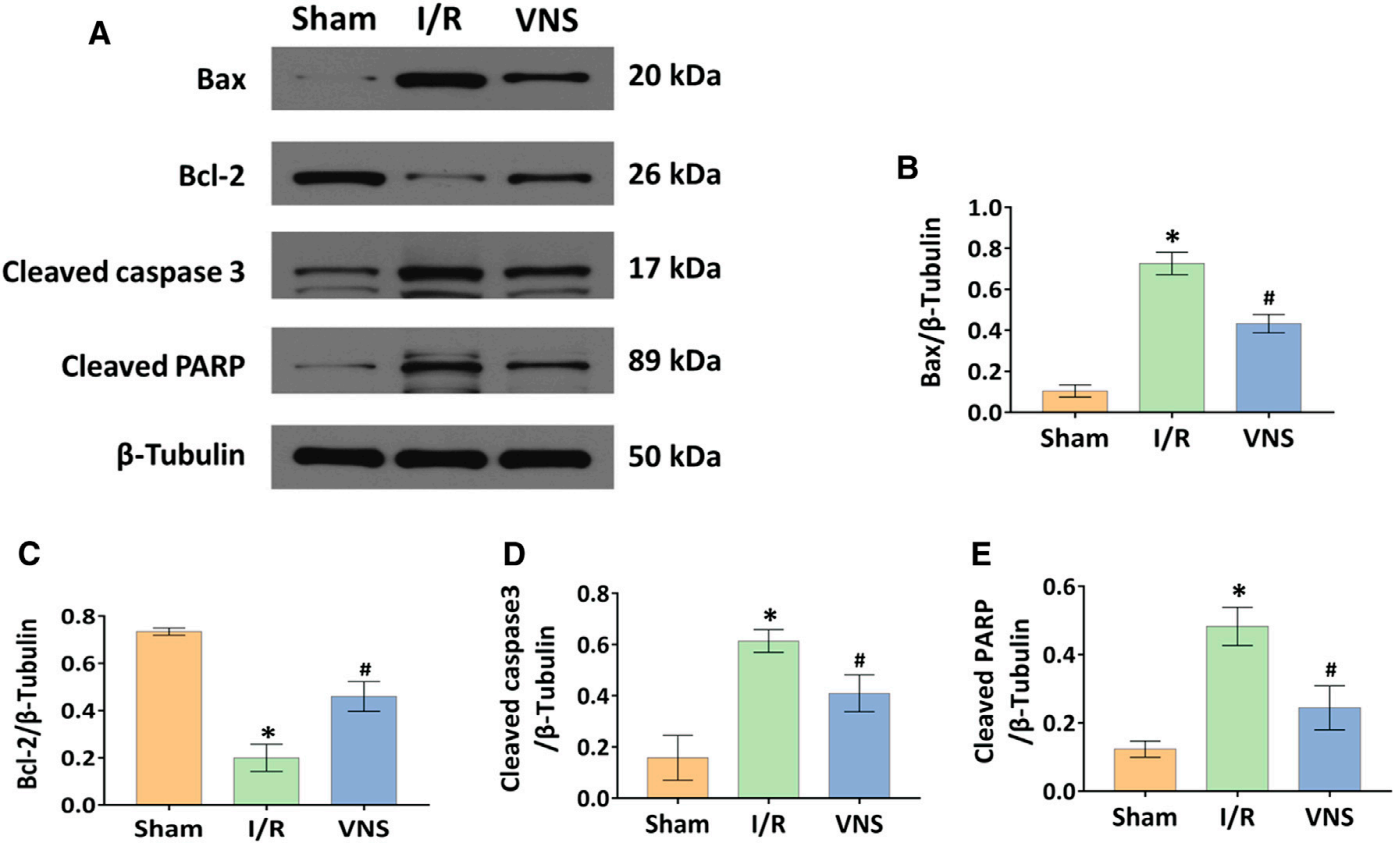
VNS reduces cell apoptosis by regulating Bax, Bcl-2, Cleaved caspase3, and Cleaved PARP. **(A)** Representative blots and relative protein levels of **(B)** Bax, **(C)** Bcl-2, **(D)** Cleaved caspase3, and (König et al., 2016) Cleaved PARP in skeletal muscle tissues from the three groups are shown. *p < 0.05 vs the Sham group; #*p* < 0.05 vs the I/R group. I/R: ischemia-reperfusion; VNS: vagus nerve stimulation.

Apoptotic cells in the skeletal muscle were detected by TUNEL staining (Figure 5A). Compared with the sham group, the number and percentage of TUNEL-positive cells were significantly increased in the I/R group (Figure 5B). However, the percentage of TUNEL-positive cells was significantly lower in the VNS group than in the I/R group (Figure 5B). Western blotting was performed to further assess skeletal muscle apoptosis (Figure 6A). Compared with the Sham group, the relative protein expression level of Bax-inducing apoptosis was significantly increased, and the relative protein expression level of Bcl-2-inhibiting apoptosis was significantly decreased in the I/R group (Figures 6B,C). In the I/R group, the relative expression of cleaved caspase3 and cleaved PARP proteins was significantly increased (Figures 6D,E). VNS reversed these changes compared to the I/R group (Figure 6B,C,E). These data suggest that VNS significantly reduced skeletal muscle cell apoptosis following hepatic I/R injury.

### Vagus Nerve Stimulation Significantly Activated the SIRT1 Signaling Pathway in Skeletal Muscle After Hepatic I/R Injury

**FIGURE 7:**
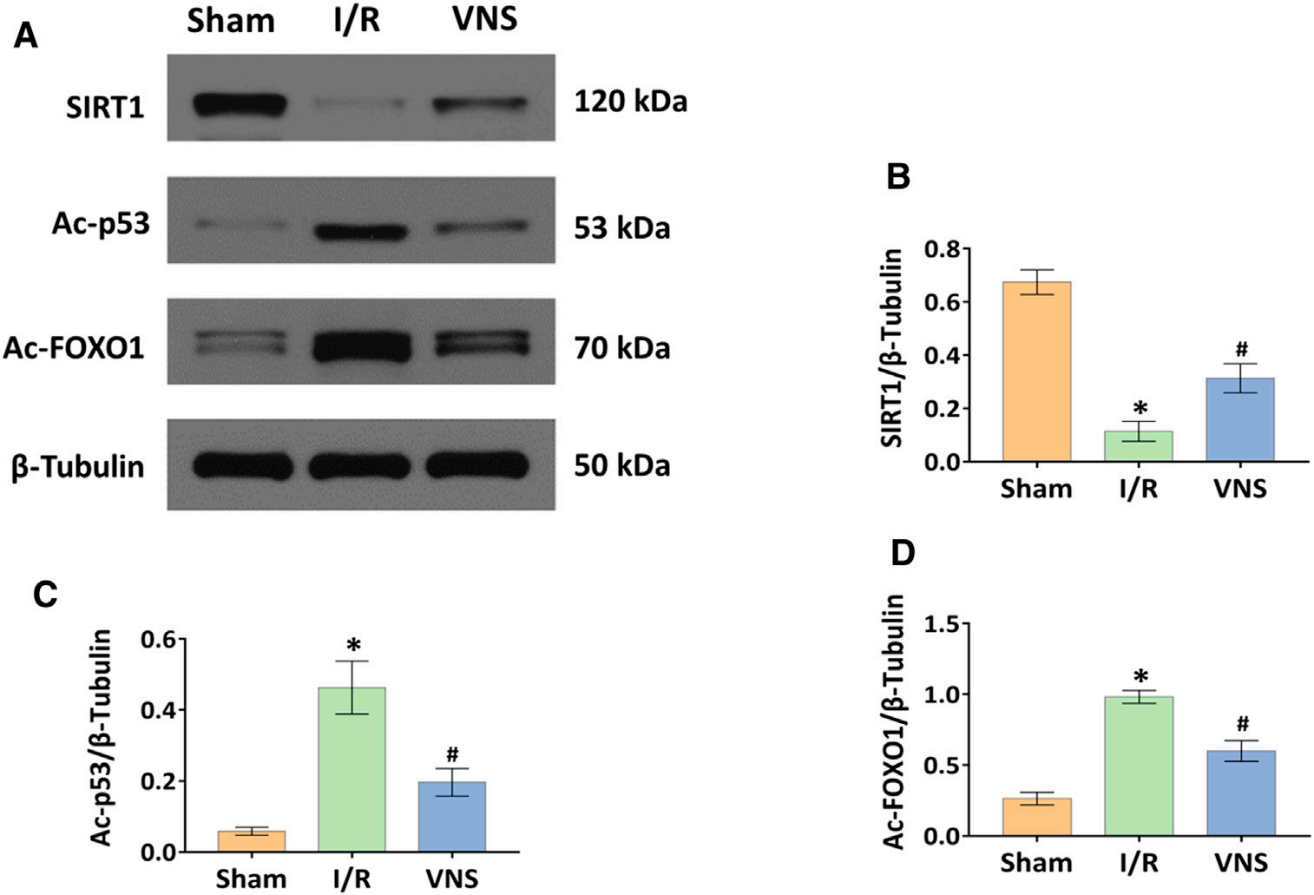
VNS activates the SIRT1 pathway in the skeletal muscle after hepatic I/R injury. **(A)** Representative blots and relative protein levels of **(B)** SIRT1, **(C)** Ac-p53 and **(D)** Ac-FOXO1 in skeletal muscle tissues from the three groups are shown. *p < 0.05 vs the Sham group; #*p* < 0.05 vs the I/R group. SIRT1: silent information regulator 1; Ac-p53: acetylated p53; Ac-FOXO1: acetylated forkhead box O1. I/R: ischemia-reperfusion; VNS: vagus nerve stimulation.

Western blotting was used to analyze the protein expression levels of SIRT1, Ac-p53, and Ac-FoxO1 (Figure 7A). Compared with the sham operation group, hepatic I/R injury significantly inhibited the relative protein expression level of SIRT1 and increased the relative protein expression levels of Ac-p53 and Ac-FoxO1 in skeletal muscle (Figures 7B–D). In contrast, VNS significantly reversed these changes compared to the I/R group (Figures 7B–D). These data suggest that the SIRT1 pathway is inhibited in skeletal muscle after hepatic I/R injury, and that VNS treatment can significantly activate the SIRT1 pathway in skeletal muscle during hepatic I/R injury.

## Discussion

**FIGURE 8:**
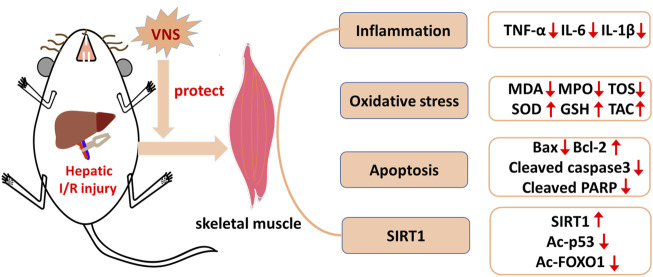
Schematic diagram depicting the protective effect of VNS on skeletal muscle after hepatic I/R injury and its potential mechanisms. VNS protects against skeletal muscle after hepatic I/R injury by inhibiting inflammation, oxidative stress, and apoptosis probably via the SIRT1

In this study, we found that hepatic I/R injury induced obvious skeletal muscle injury, characterized by muscle fiber disorder, stripe loss, and inflammatory cell infiltration; however, VNS alleviated this injury. Our study provides new evidence that VNS may have a protective effect on skeletal muscle injury caused by hepatic I/R, and provides evidence that VNS reduces skeletal muscle injury caused by hepatic I/R by inhibiting inflammation, oxidative stress, and apoptosis, specifically by activating the SIRT1 signaling pathway in skeletal muscle (Figure 8). To our knowledge, this is the first study to use VNS for the treatment of skeletal muscle injury caused by hepatic I/R.

Sympathetic neural activation may be detrimental to tissue injury caused by I/R (Lambert et al., 2016; Sun et al., 2020). I/R injury can lead to tissue ischemia hypoxia, which is the most common stress-induced sympathetic activation, and may further increase damaged tissues. The VNS can increase the vagus tension and offset the sympathetic vasoconstriction effect, thus playing an important role in organ protection. Vagus nerve includes afferent fibers and efferent fibers. Efferent fibers are the main protective fibers in I/R injury (Bonaz et al., 2016; Nuntaphum et al., 2018). The role of VNS for the treatment of organ injury caused by I/R has been reported. Wang et al. (Wang et al., 2020) found that VNS improved renal I/R injury. VNS also showed potential therapeutic effects in distant organ injury caused by I/R in some organs. Lai et al. (Lai et al., 2019) found that VNS protected against liver injury caused by renal I/R through anti-oxidative stress and anti-inflammatory effects. The results obtained by Zhang et al. (Zhang et al., 2019b) showed that VNS can alleviate hepatic I/R injury. In our study, H&E staining of skeletal muscle in the VNS treatment group showed a significant improvement in skeletal muscle injury compared with that in the I/R group, showing reduced muscle fiber disorder and stripe loss and decreased inflammatory cell infiltration. TUNEL-positive cells also showed decreased apoptosis in the VNS group. Compared with the I/R group, the VNS group also had lower levels of TNF-α, IL-6, and IL-1β and oxidative stress levels of MDA and MPO, the levels of SOD and GSH against oxidative stress were increased, and serum levels of AST, ALT, LDH, CK, and MM-CK were decreased. Although CK could not distinguish skeletal muscle injury from myocardial injury, combined with other indicators, such as HE histological score, MM-CK level and TUNEL staining, we found that VNS may alleviate skeletal muscle injury caused by hepatic I/R.

Excessive inflammation and oxidative stress are the most important mechanisms of distant organ injury caused by I/R (Lambert et al., 2016). VNS may be involved in maintaining oxidative homeostasis and in response to external stimuli or injury (Sun et al., 2020). ROS play an important role in a variety of pathological processes in I/R injury; although ROS is involved as a mitochondrial oxidation by-product in the process of breathing, under low oxygen conditions (I/R injury can cause lack of oxygen), it is overproduced, which may cause I/R of organs and remote organs i.e., skeletal muscle injury (Weinbroum et al., 1995). A previous study has shown that the sensitivity of gastrocnemius and soleus muscles to I/R is different, possibly due to their different metabolic phenotypes. glycolytic superficial gastrocnemius muscles are more prone to I/R than oxidative soleus ones. Less-IR induced damage is observed in oxidative muscle. The fact that oxidative muscles naturally equip with higher antioxidant contents than glycolytic muscles likely protect them in the setting of IR (Charles et al., 2017). During hepatic I/R injury, liver tissue releases cytokines and oxygen free radicals to produce destructive pro-inflammatory effects that damage distant organs. Borovikova et al. (2000), Bonaz et al. (2016) reported that VNS reduced the systemic inflammatory response and significantly inhibited TNF-α, IL-6, and IL-1β levels. Cui et al. (Cui et al., 2018) found that the protection of short-acting opioids against I/R injury in multiple organs, including the liver, acts through the central vagus pathway. Many studies have reported that VNS can alleviate hepatic I/R injury and skeletal muscle I/R injury by inhibiting inflammation and alleviating oxidative stress (Zhang et al., 2019a; Zhang et al., 2019b; Lai et al., 2019; Xia et al., 2020). Medeiros et al. (Lauz Medeiros et al., 2016) found that antioxidants protect against intestinal damage caused by hepatic I/R injury by reducing inflammation and oxidative stress. Our study found that inflammation and oxidative stress levels of TNF-α, IL-6, IL-1β, MPO, and MAD were significantly decreased in the VNS treatment group. Based on the results of previous studies and our current findings, we hypothesized that inflammation and oxidative stress also play important roles in skeletal muscle damage caused by hepatic I/R injury. In conclusion, VNS may treat skeletal muscle damage caused by hepatic I/R injury.

Reducing apoptosis is another important mechanism for alleviating distant organ injury caused by I/R. Studies have found that the vagus can reduce the hepatic I/R-induced apoptosis by activating α7nicotinic acetylcholine receptor-containing Kupfer cells (Ni et al., 2016), suggesting that the vagus can mediate the protective effect of hepatic I/R via anti-apoptosis, while, VNS can reduce apoptosis and protect against I/R injury induced acute skeletal muscle injury (Zhang et al., 2019a). Jing et al. (Jing et al., 2014) found that omega-3 polyunsaturated fatty acids can reduce cell apoptosis in lung injury caused by intestinal I/R, indicating that cell apoptosis plays an important role in distant organ injury caused by I/R. Lai et al. (Lai et al., 2019) showed that VNS also protects against liver injury caused by renal I/R by reducing apoptosis. Our study showed that the levels of Bax, cleaved caspase-3, and cleaved PARP decreased, while the levels of Bcl-2 were increased in the VNS group compared with the I/R group. Therefore, we hypothesized that VNS may alleviate the skeletal muscle injury caused by hepatic I/R by reducing apoptosis.

SIRT1 is a nicotinamide adenine dinucleotide (NAD) -dependent histone deacetylase that regulates oxidative stress, inflammation, apoptosis, metabolism, and skeletal muscle function through the deacetylation of various substrates (Chung et al., 2010; Zhang et al., 2017). It has been reported that SIRT1 can inhibit the oxidative stress of vascular endothelial cells, thus producing beneficial effects on the cardiovascular system and alleviating myocardial I/R injury and hepatic I/R injury (Zhang et al., 2017). Knight et al. (Knight et al., 2011) found that cutting off the vagus inhibited the expression of SIRT1, suggesting that VNS is associated with activation of the SIRT1 pathway. SIRT1 is thought to be involved in the protective effect of skeletal muscle and protects endothelial cells from aging under ischemic conditions (Pinton et al., 2007; Ugwu et al., 2017). Cheng et al. (Cheng et al., 2016) also found that the activation of SIRT1 protects against skeletal muscle I/R injury by alleviating oxidative stress and mitochondrial dysfunction. Liu et al. (Liu et al., 2020) found that upregulation of SIRT1 expression in lung tissue alleviated renal I/R-induced lung injury. Based on these studies, we hypothesized that VNS could alleviate skeletal muscle injury caused by hepatic I/R by activating SIRT1. Our study found that the expression of SIRT1 in the VNS treatment group was significantly higher than that of the I/R group, which was accompanied by a decrease in the expression of Ac-p53 and Ac-FoxO1, which confirmed our speculation.

This study has some limitations. First, we investigated skeletal muscle injury caused by I/R, but did not evaluate its effect on skeletal muscle function and muscle strength change, and it is not clear whether VNS has a therapeutic effect on those factors. Second, we only studied activation of the SIRT1 pathway after VNS, and the role of VNS in the activation of SIRT1 has yet to be mechanistically described. Third, although we have uncovered several potential mechanisms by which VNS protects skeletal muscle injury due to hepatic I/R, the exact ways in which these mechanisms functionally interact remains to be further explored.

Hepatic I/R injury is a common clinical condition that leads to acute skeletal muscle injury, which ultimately increases mortality rate. To date, there have been no effective interventions to alleviate this type of damage. In the present study, we provided the first evidence that VNS exerted skeletal muscle protection in a hepatic I/R rat model by inhibiting inflammation and apoptosis, enhancing the antioxidant capability, and activating the SIRT1 signaling pathway. This indicates that VNS may be a promising adjuvant therapeutic strategy for treating skeletal muscle damage caused by liver injury and improve the outcomes of patients with liver injury and remote organ dysfunction.

## Data Availability

The raw data supporting the conclusions of this article will be made available by the authors, without undue reservation.
