# Supplementary figures and images for: Vagus Nerve Stimulation Attenuates Acute Skeletal Muscle Injury Induced by Hepatic Ischemia/Reperfusion Injury in Rats (part 1 of 3)
Source: Front Pharmacol. 2022 Jan 3;12:756997. doi: 10.3389/fphar.2021.756997 (PMC8762262; doi:10.3389/fphar.2021.756997)

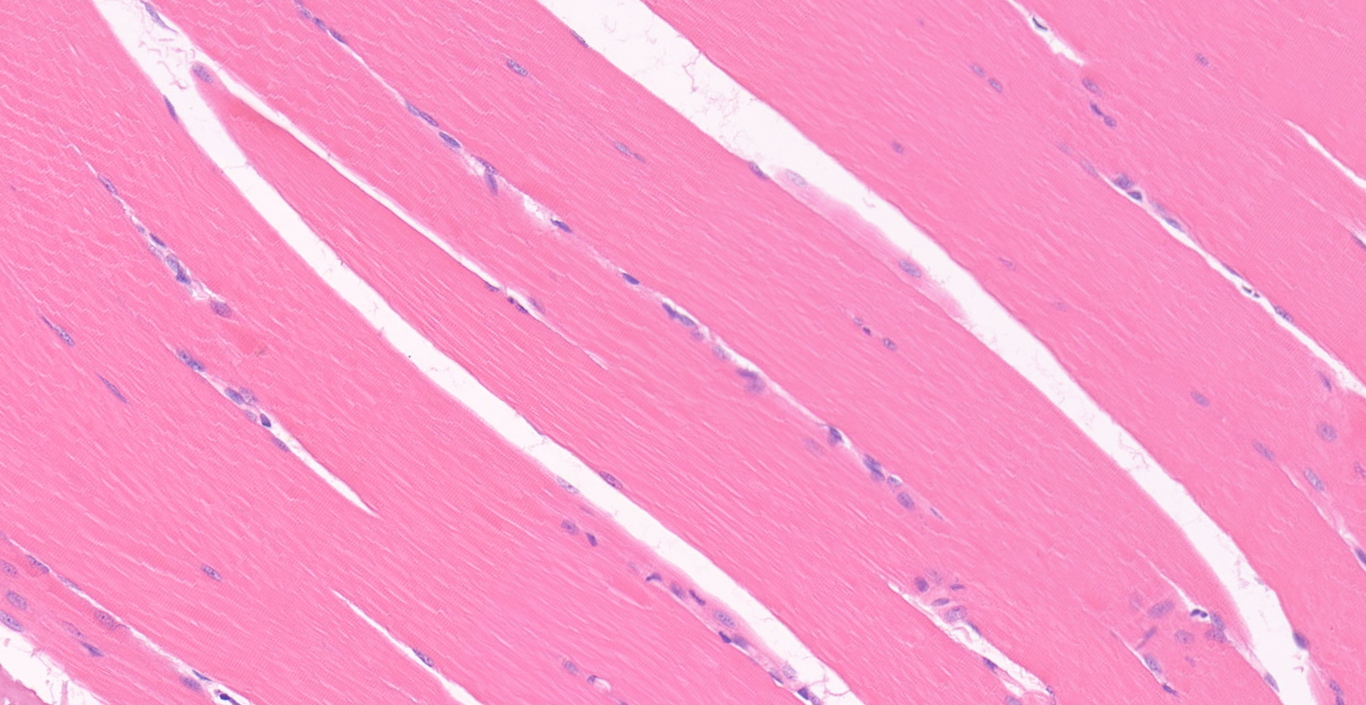

Supplement: Supplementary file 1 [file DataSheet3.ZIP › VNS/1-1.jpg]

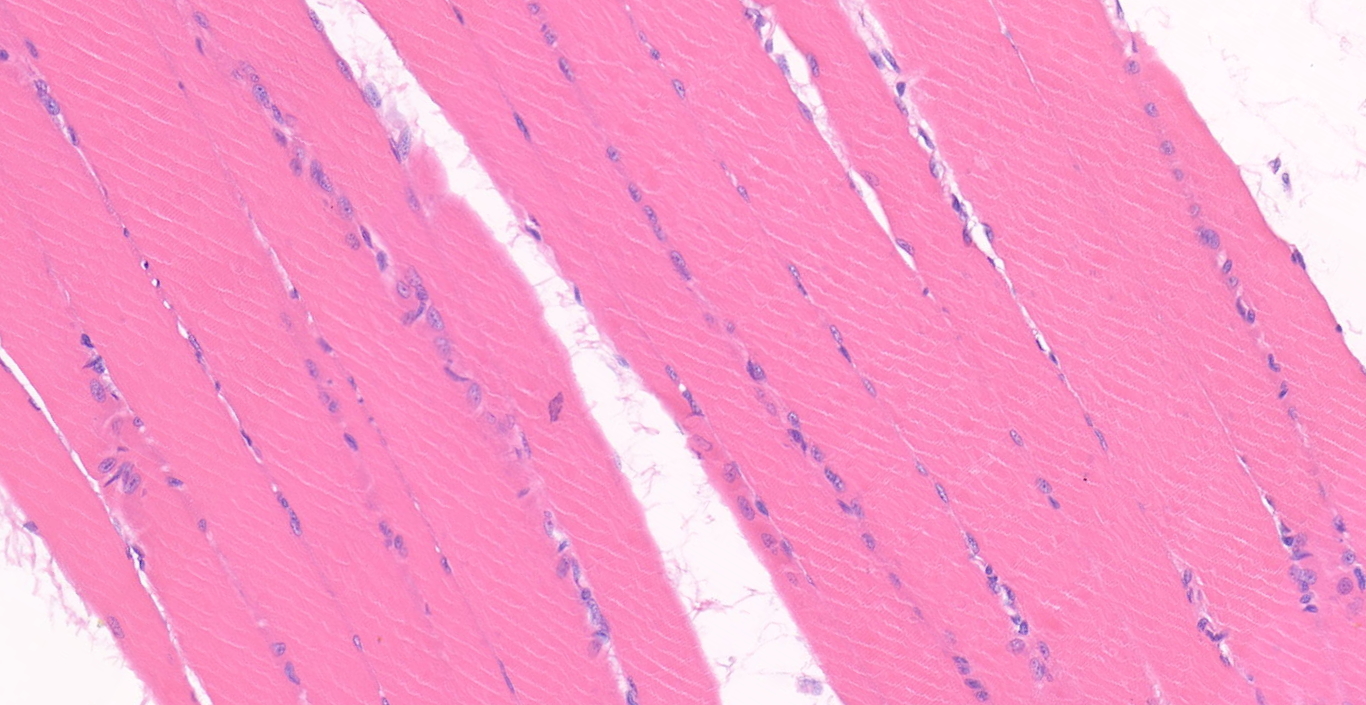

Supplement: Supplementary file 1 [file DataSheet3.ZIP › VNS/1-2.jpg]

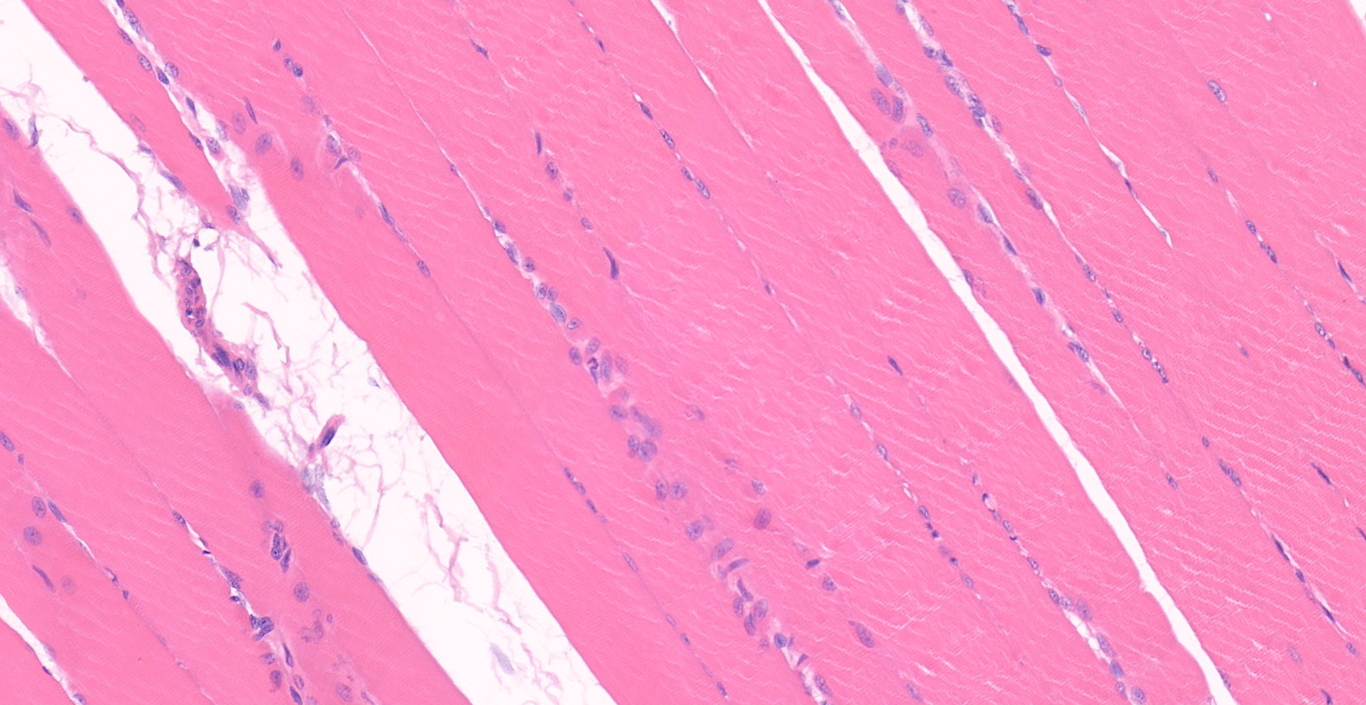

Supplement: Supplementary file 1 [file DataSheet3.ZIP › VNS/1-3.jpg]

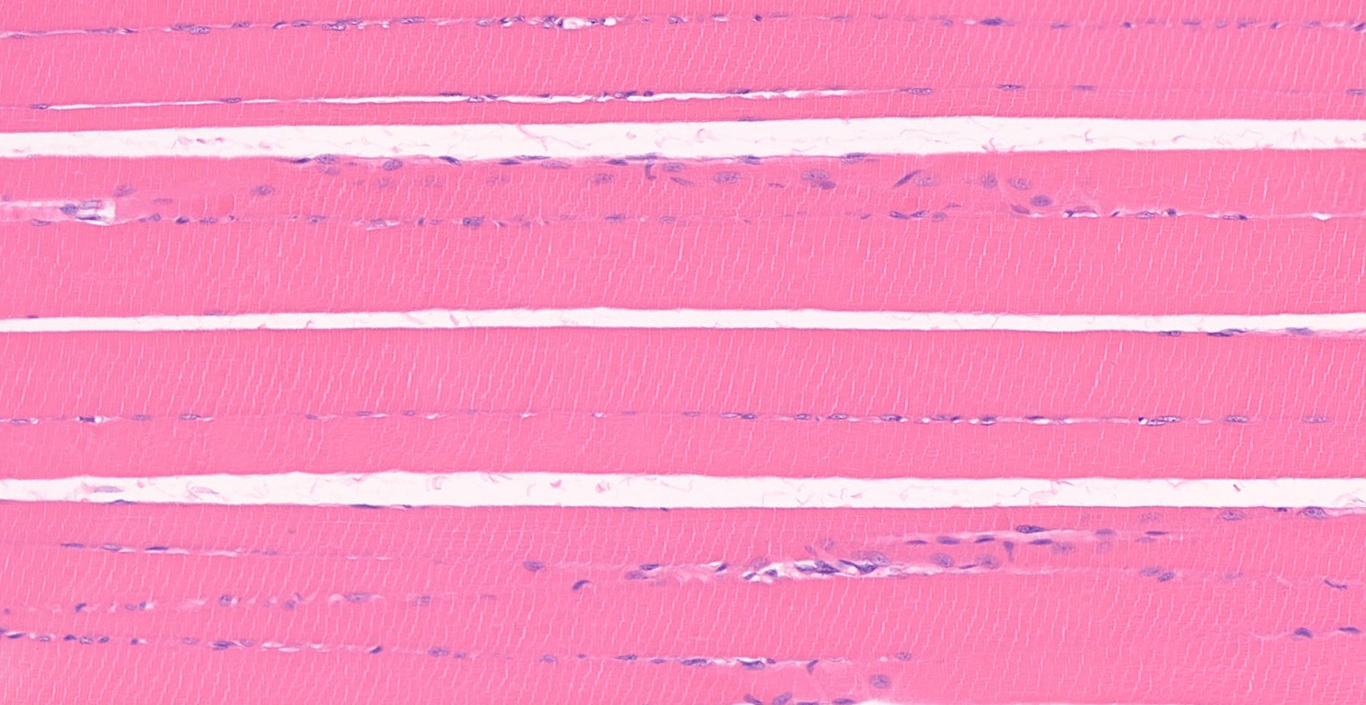

Supplement: Supplementary file 1 [file DataSheet3.ZIP › VNS/2-1.jpg]

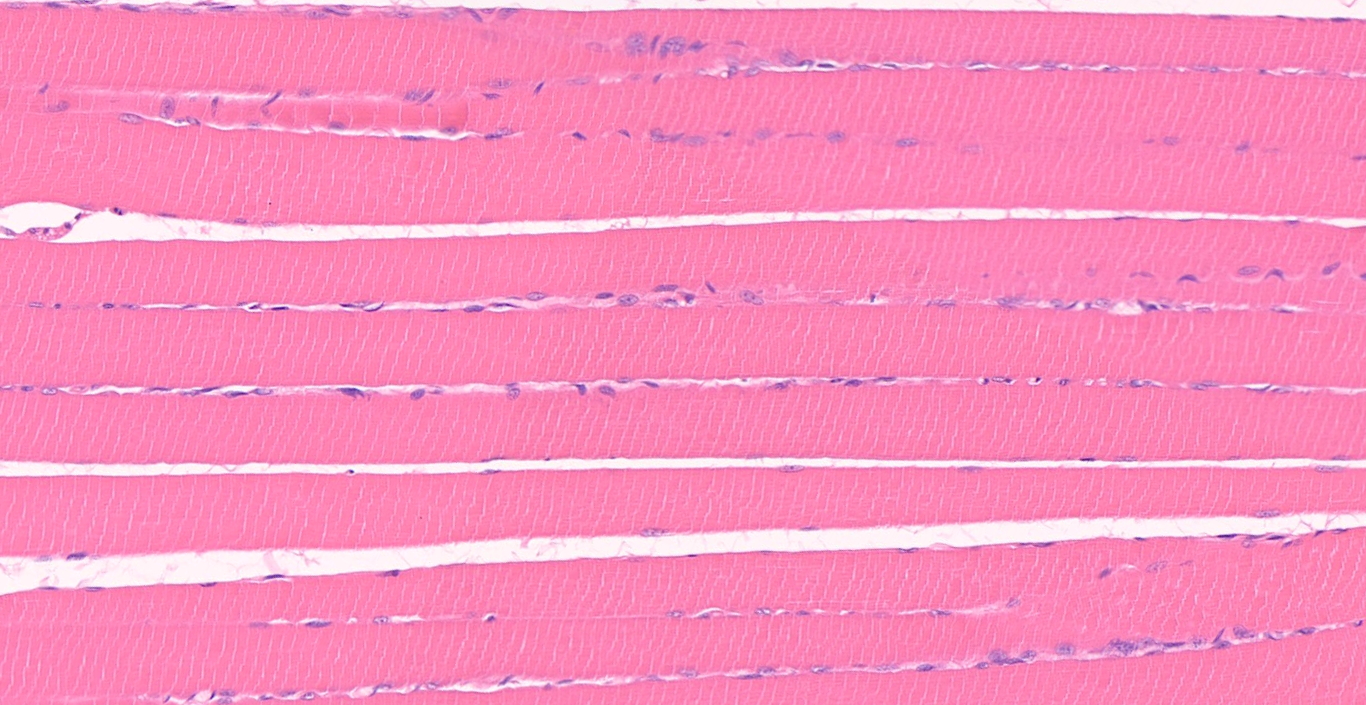

Supplement: Supplementary file 1 [file DataSheet3.ZIP › VNS/2-2.jpg]

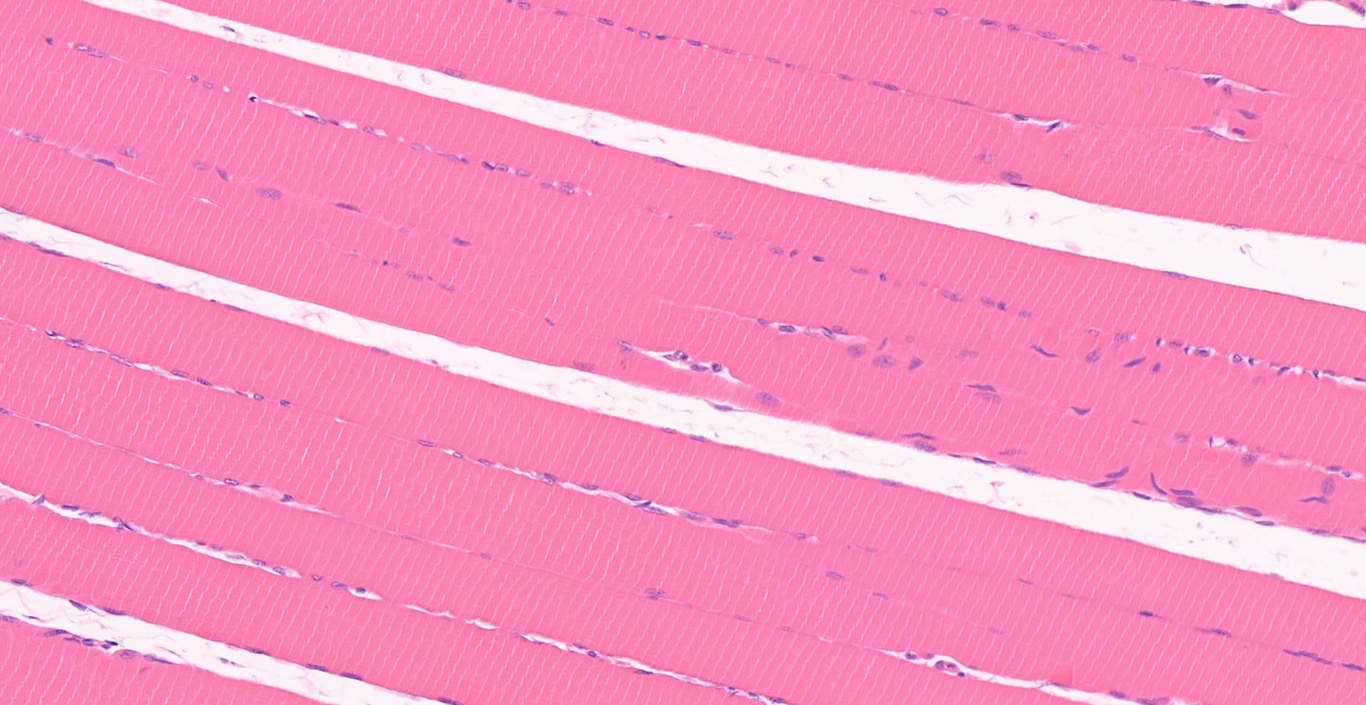

Supplement: Supplementary file 1 [file DataSheet3.ZIP › VNS/2-3.jpg]

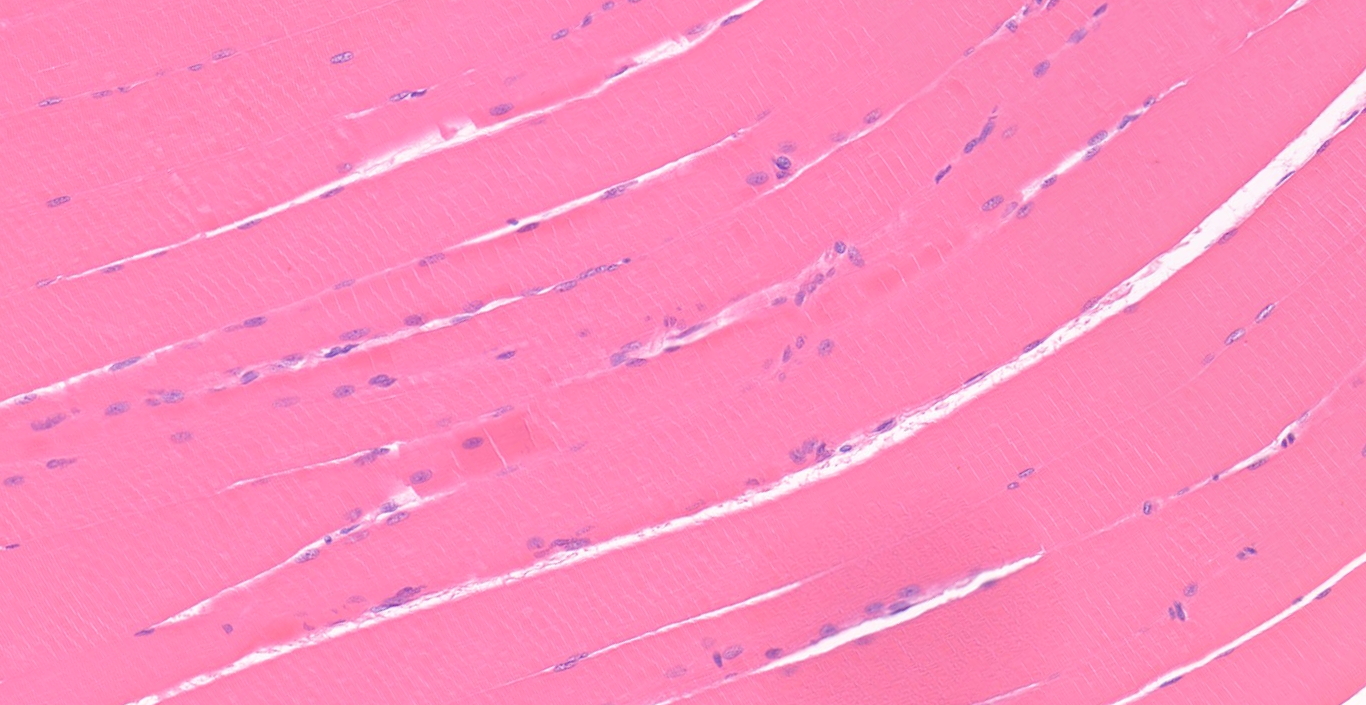

Supplement: Supplementary file 1 [file DataSheet3.ZIP › VNS/3-1.jpg]

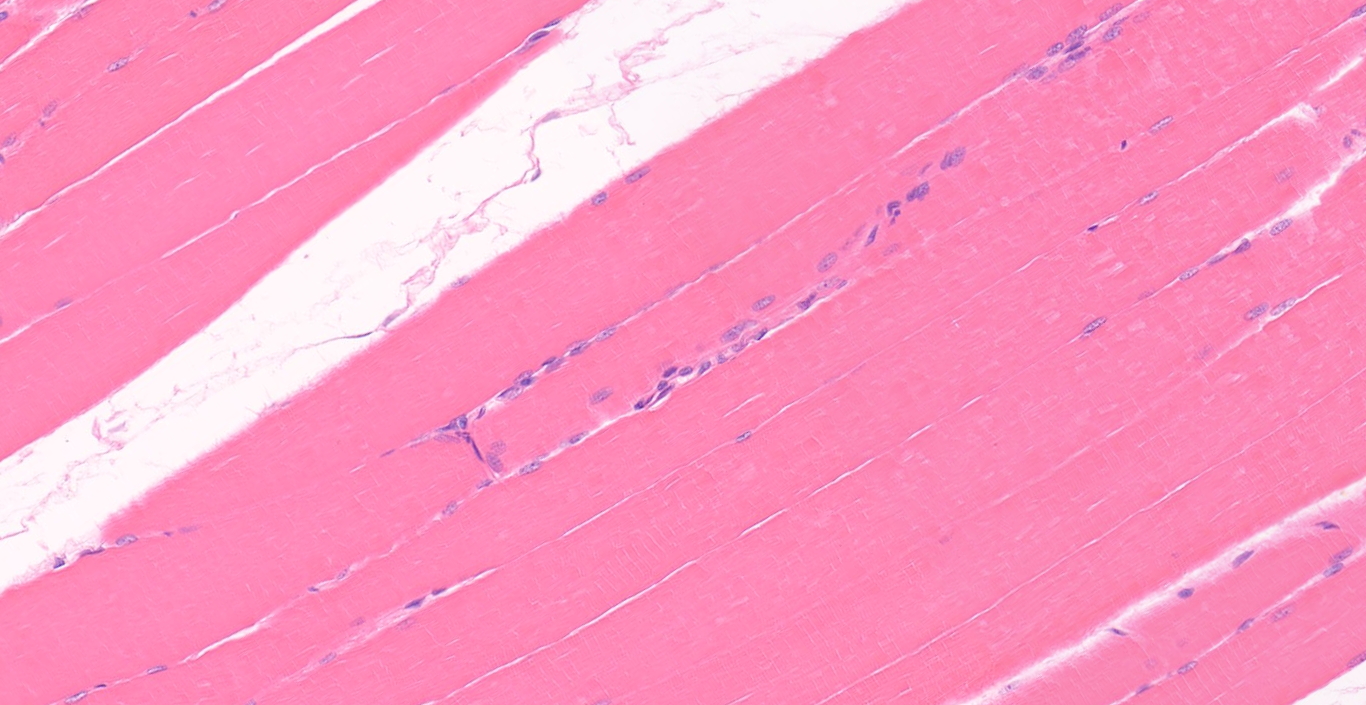

Supplement: Supplementary file 1 [file DataSheet3.ZIP › VNS/3-2.jpg]

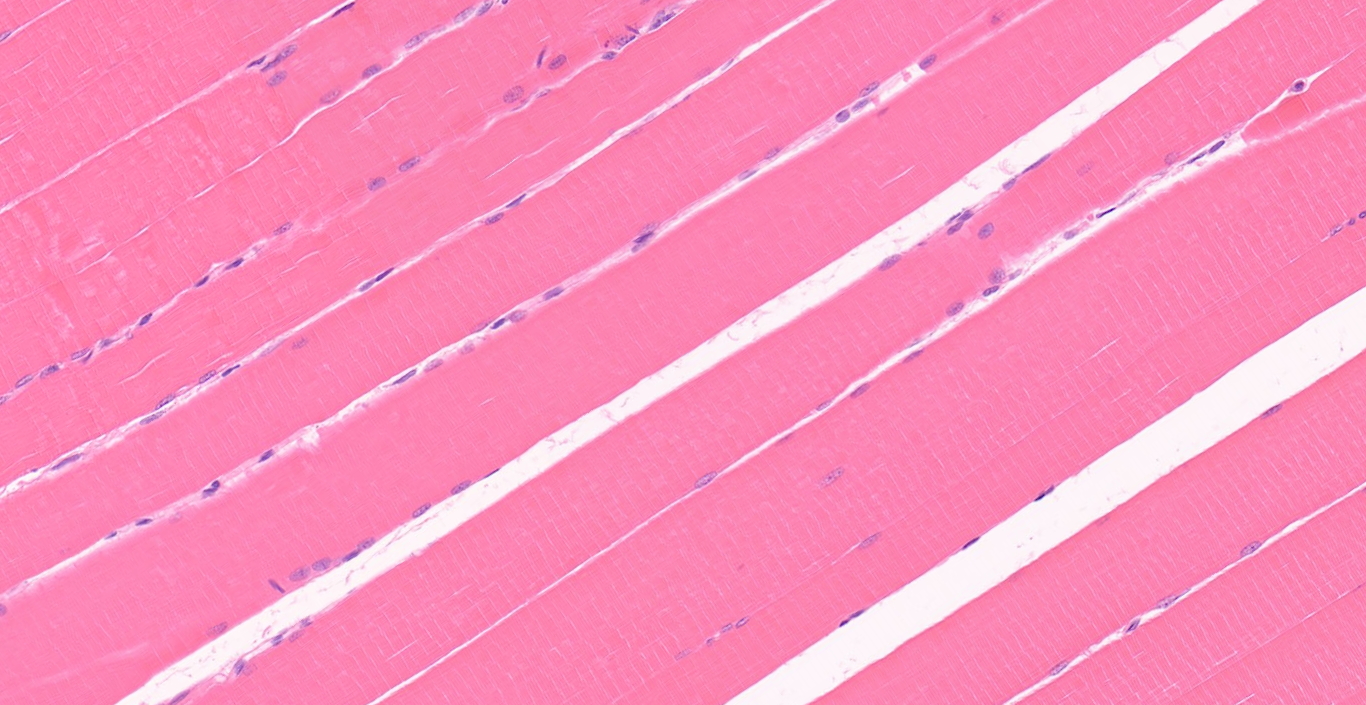

Supplement: Supplementary file 1 [file DataSheet3.ZIP › VNS/3-3.jpg]

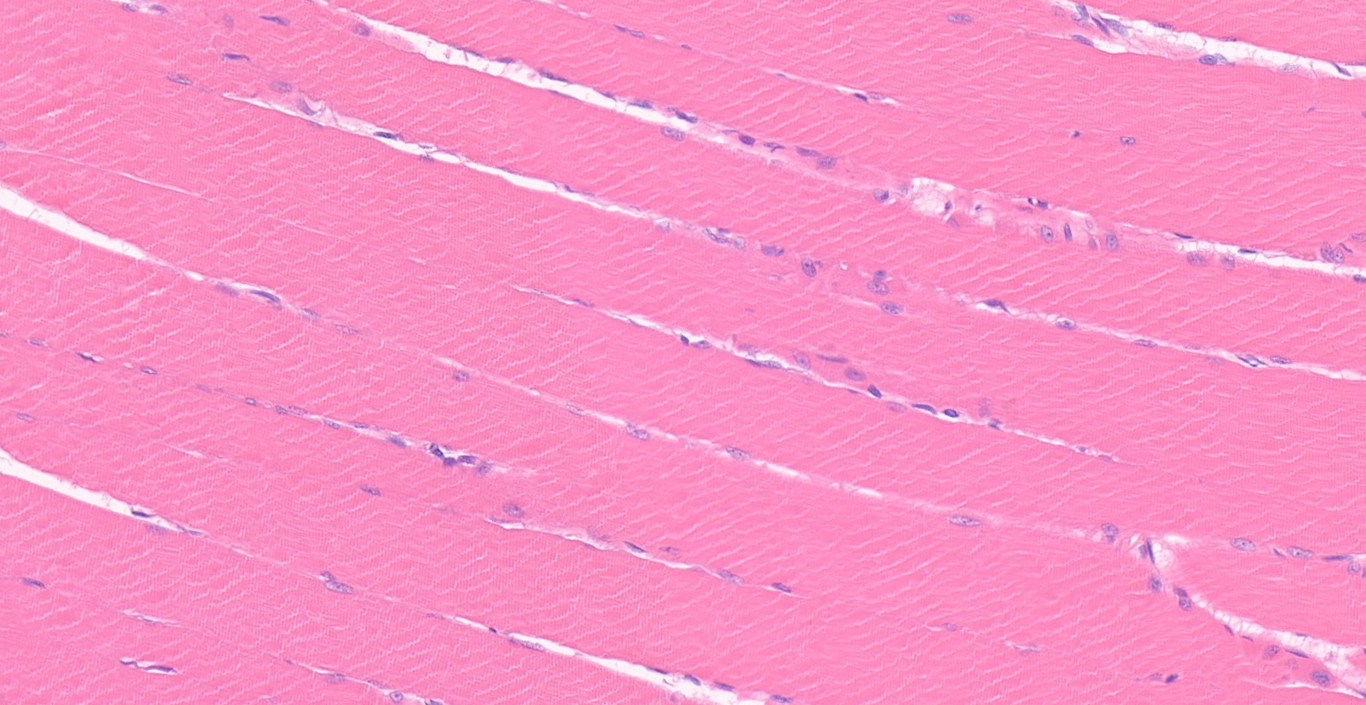

Supplement: Supplementary file 1 [file DataSheet3.ZIP › VNS/5-3.jpg]

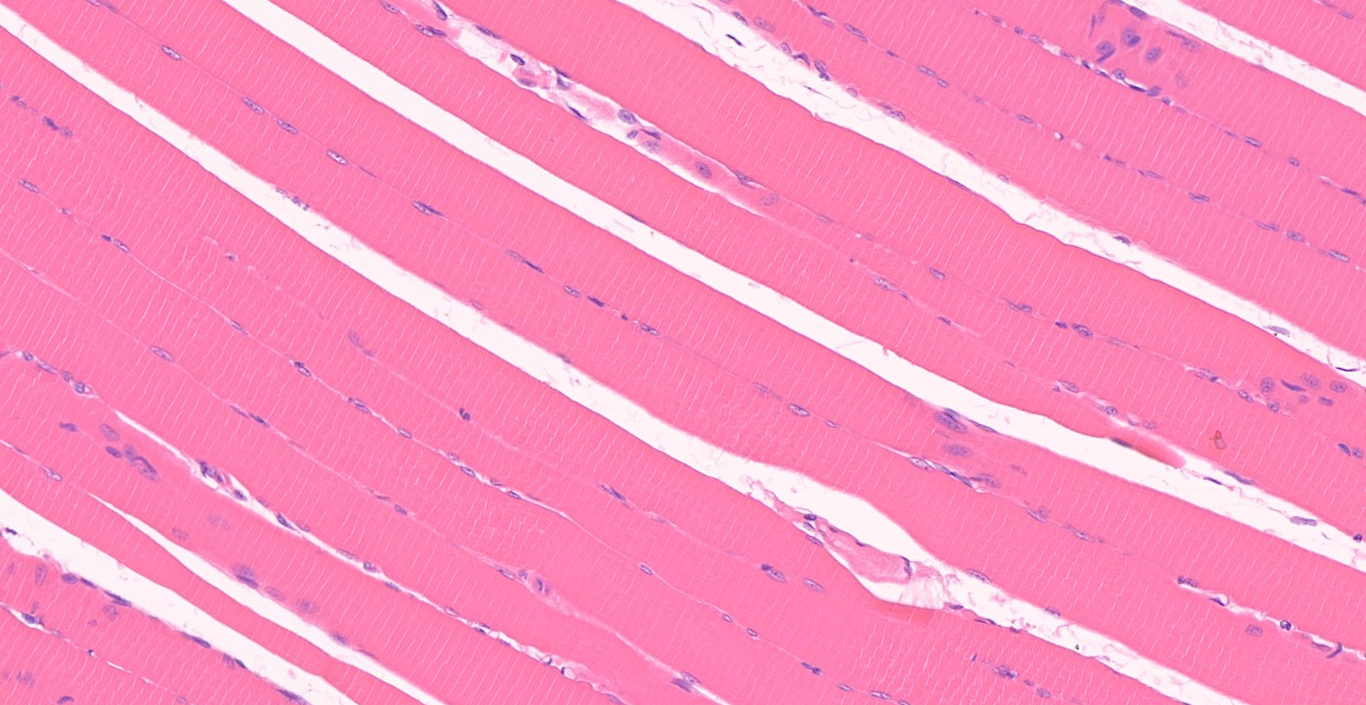

Supplement: Supplementary file 1 [file DataSheet3.ZIP › VNS/6-1.jpg]

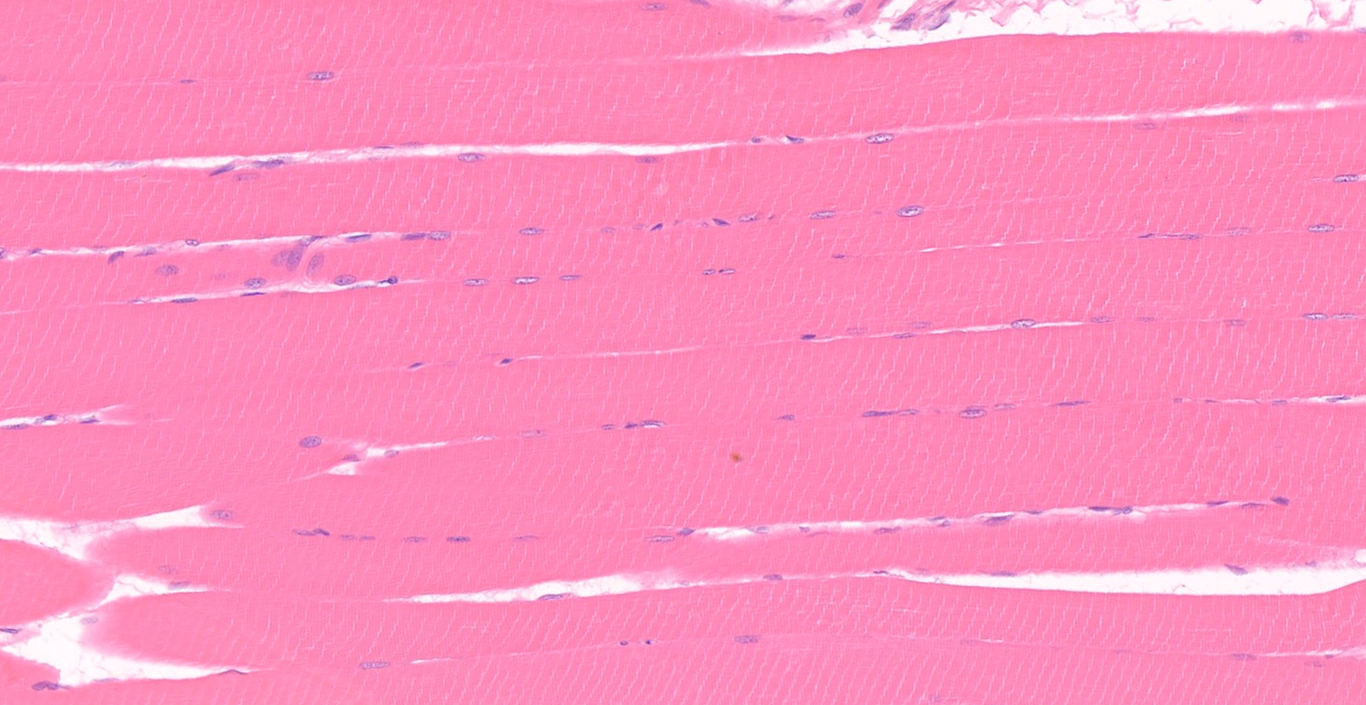

Supplement: Supplementary file 1 [file DataSheet3.ZIP › VNS/6-2.jpg]

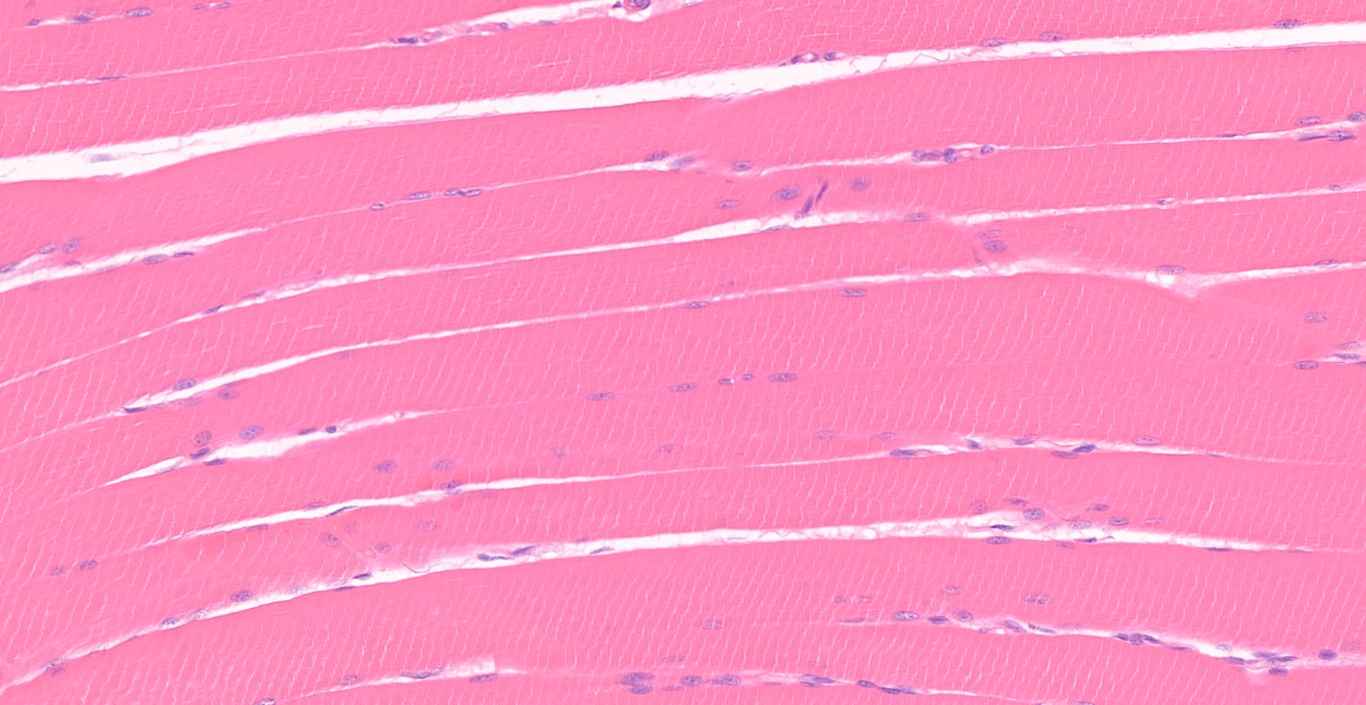

Supplement: Supplementary file 1 [file DataSheet3.ZIP › VNS/6-3.jpg]

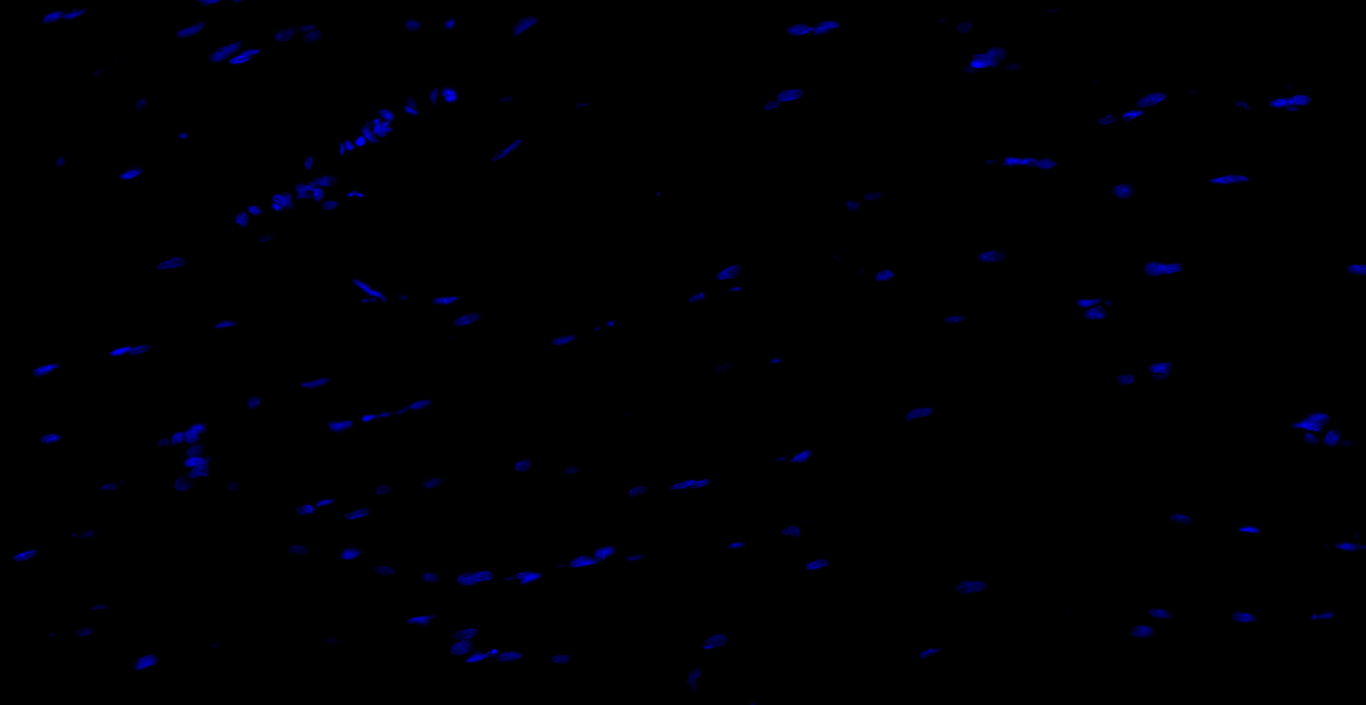

Supplement: Supplementary file 2 [file DataSheet4.ZIP › Supplemental materials 1/TUNEL/IR/1-1 DAPI.jpg]

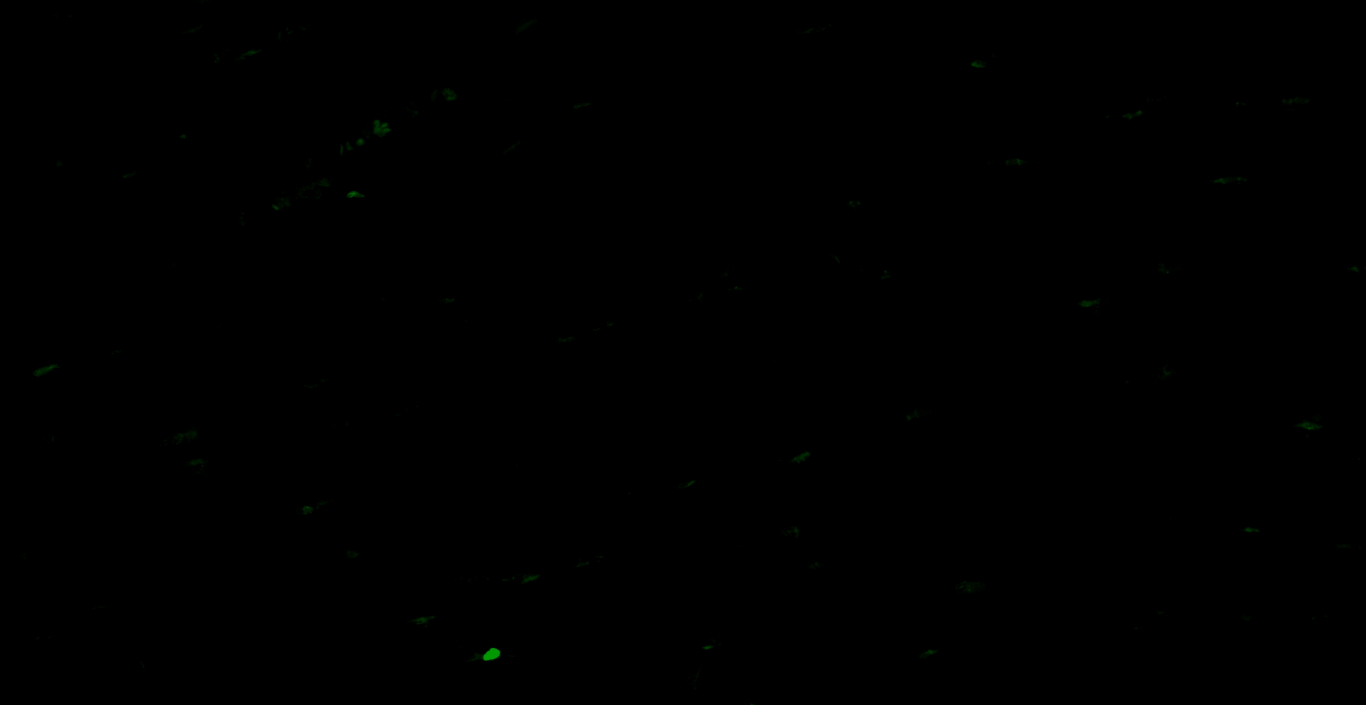

Supplement: Supplementary file 2 [file DataSheet4.ZIP › Supplemental materials 1/TUNEL/IR/1-1 TUNEL.jpg]

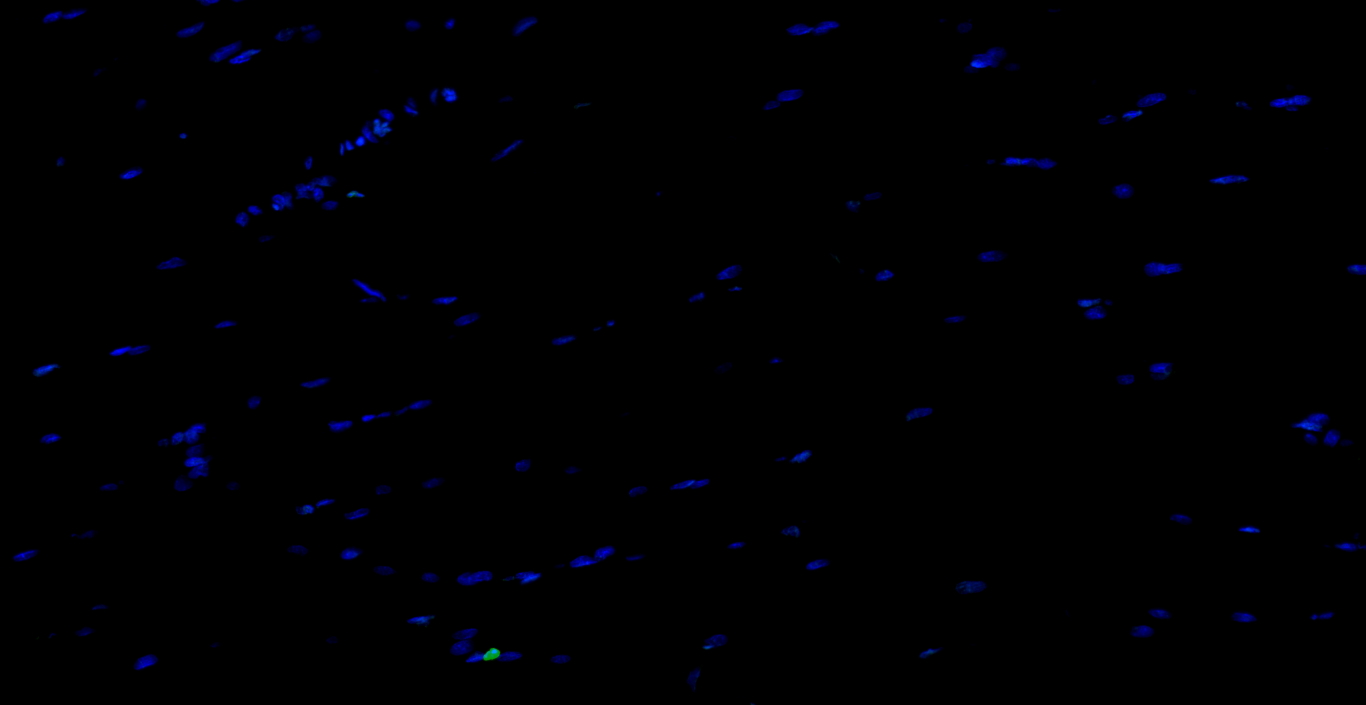

Supplement: Supplementary file 2 [file DataSheet4.ZIP › Supplemental materials 1/TUNEL/IR/1-1 merge.jpg]

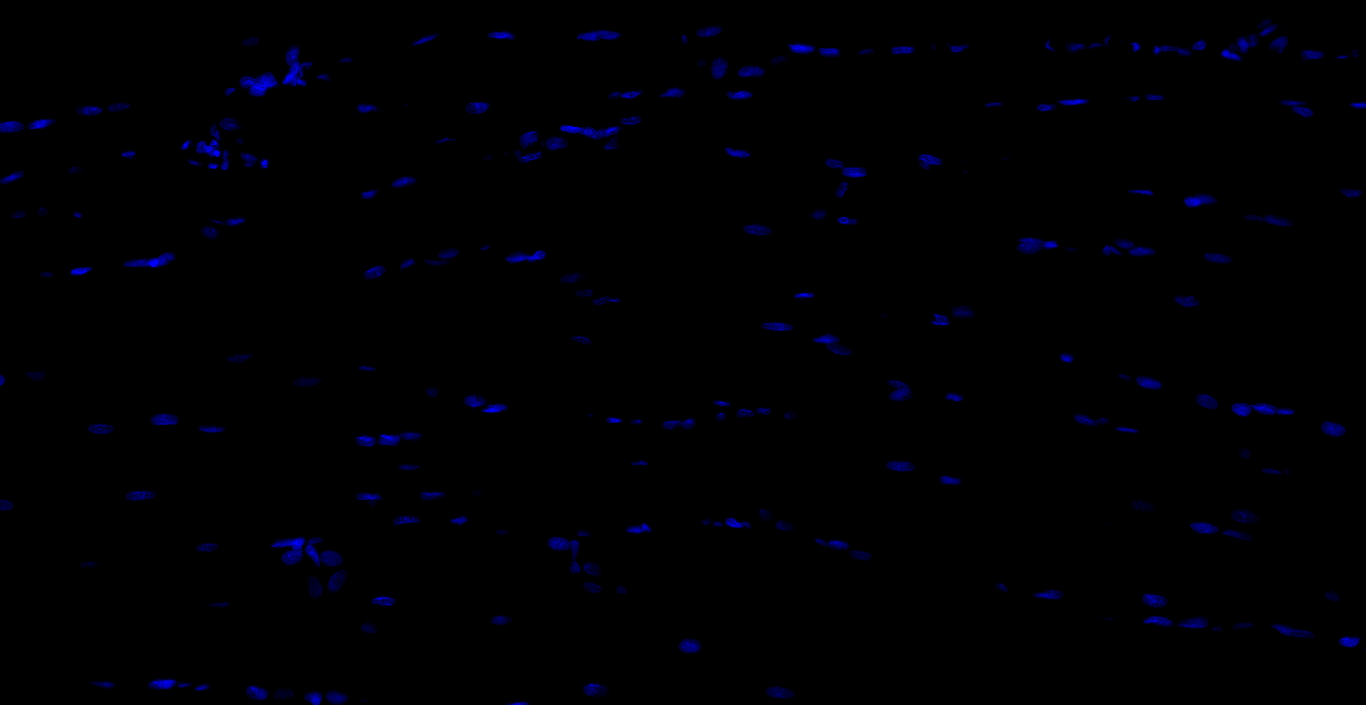

Supplement: Supplementary file 2 [file DataSheet4.ZIP › Supplemental materials 1/TUNEL/IR/1-2 DAPI.jpg]

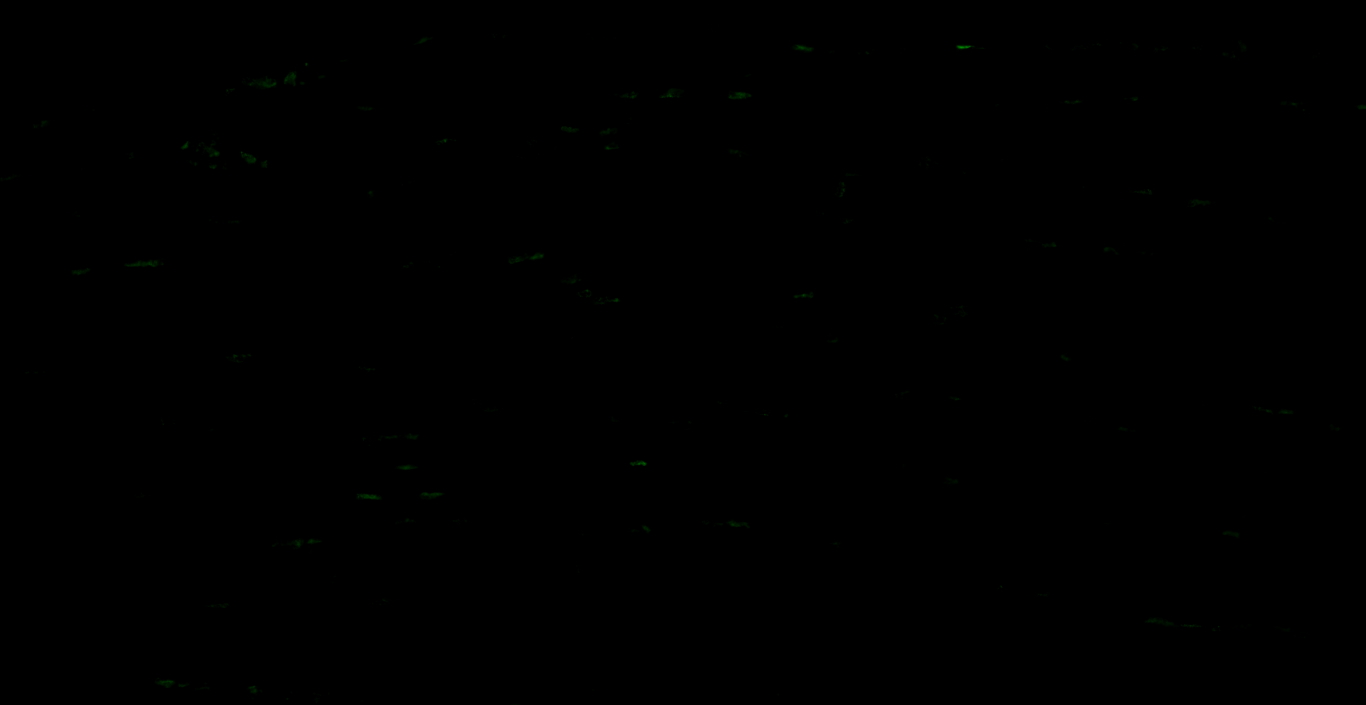

Supplement: Supplementary file 2 [file DataSheet4.ZIP › Supplemental materials 1/TUNEL/IR/1-2 TUNEL.jpg]

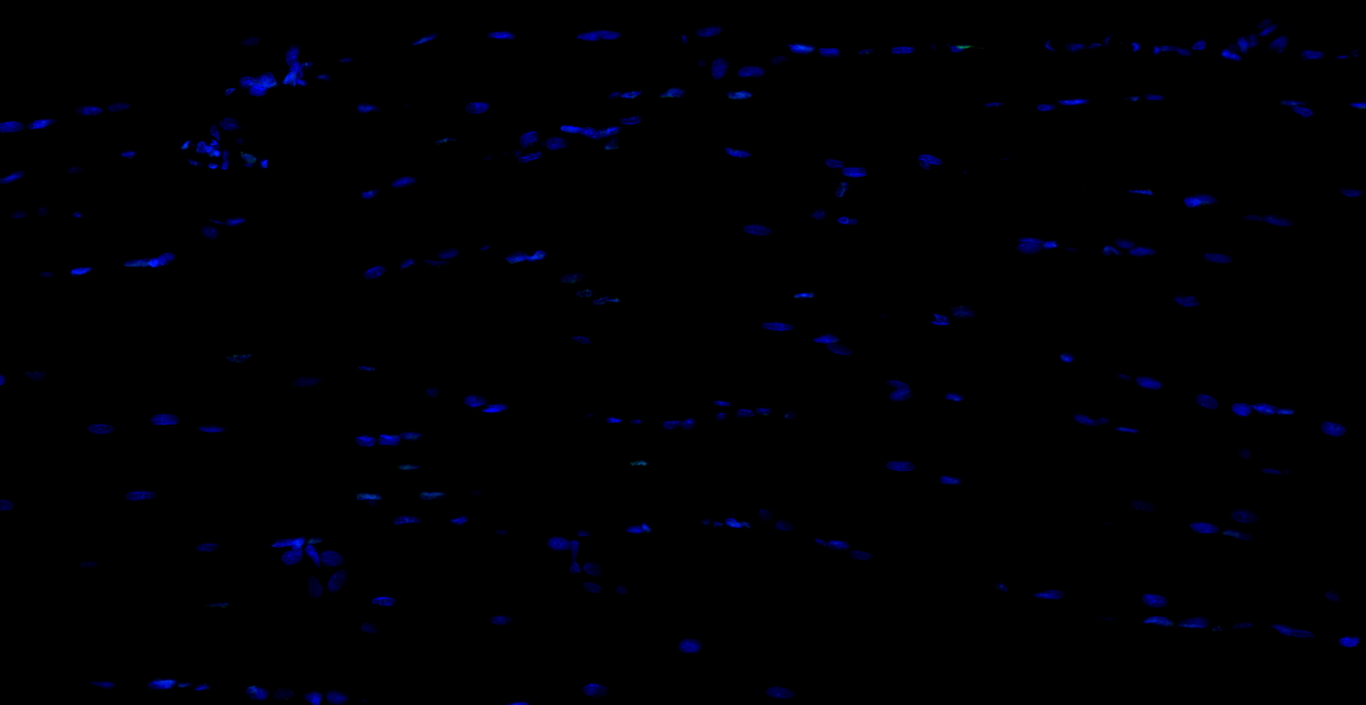

Supplement: Supplementary file 2 [file DataSheet4.ZIP › Supplemental materials 1/TUNEL/IR/1-2 merge.jpg]

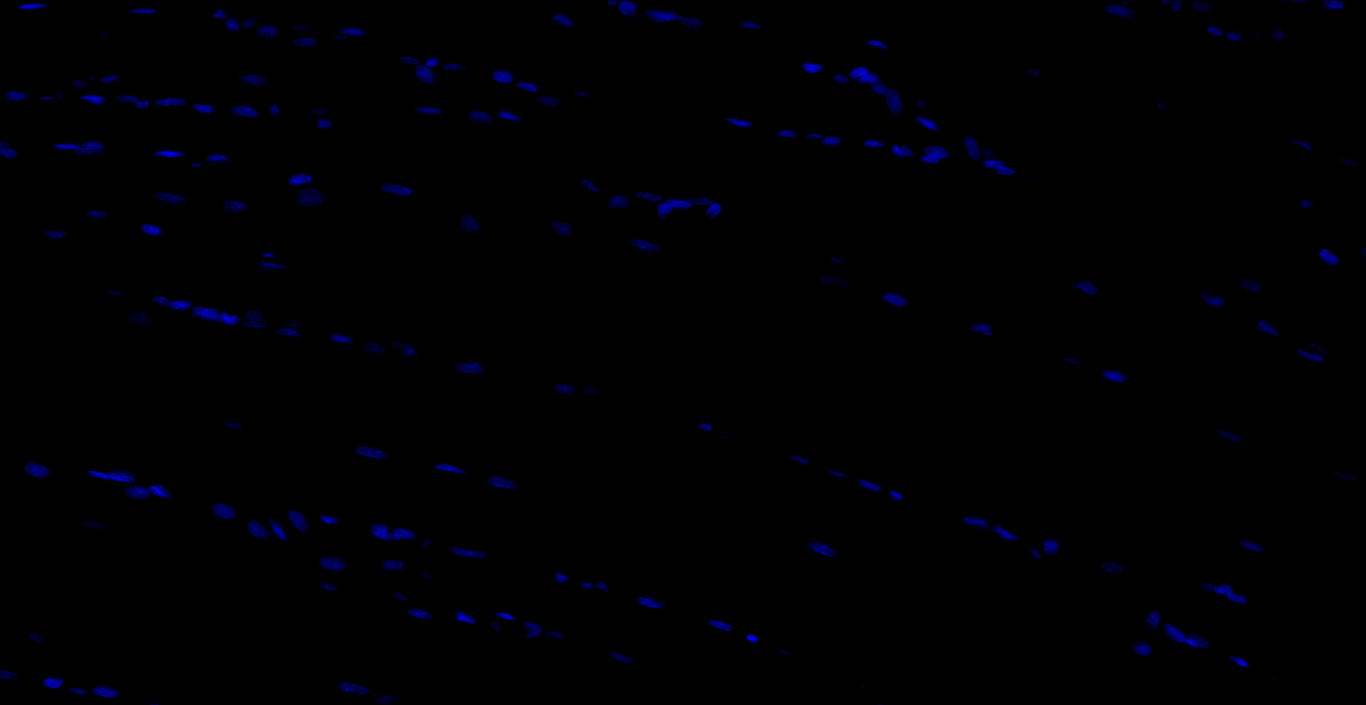

Supplement: Supplementary file 2 [file DataSheet4.ZIP › Supplemental materials 1/TUNEL/IR/1-3 DAPI.jpg]

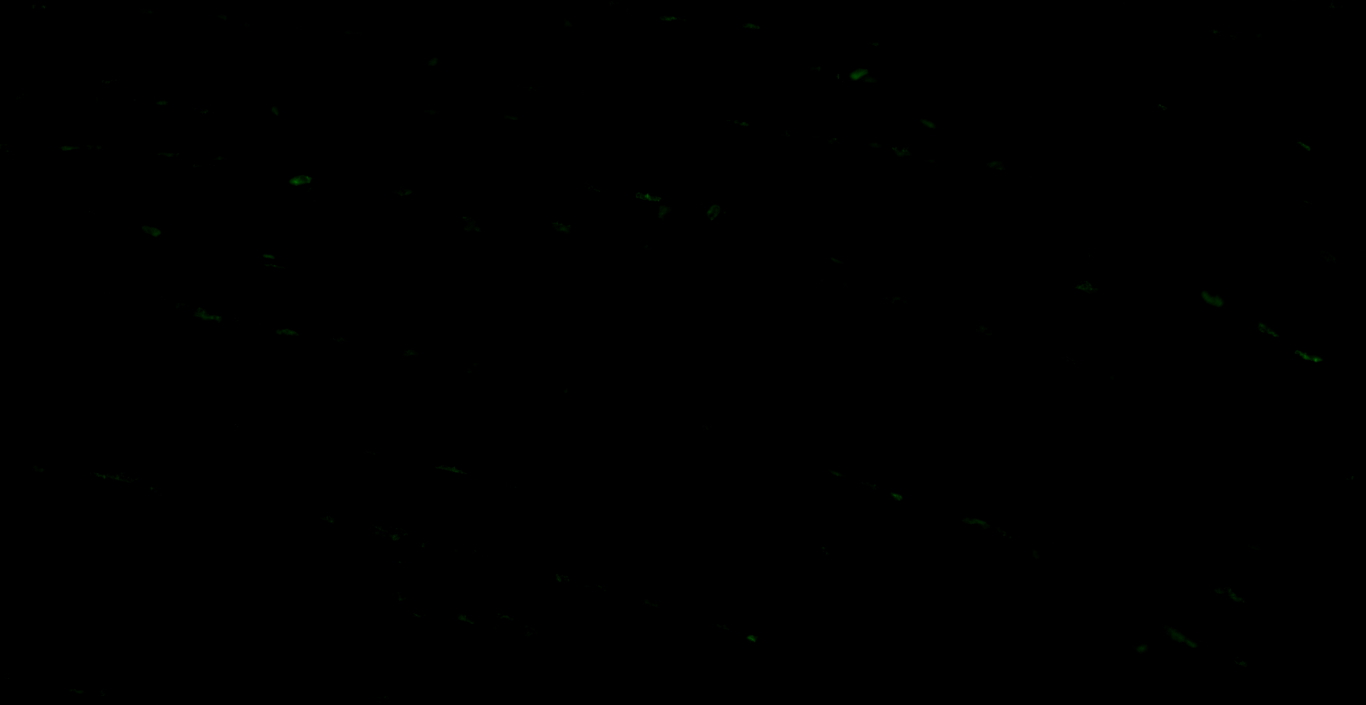

Supplement: Supplementary file 2 [file DataSheet4.ZIP › Supplemental materials 1/TUNEL/IR/1-3 TUNEL.jpg]

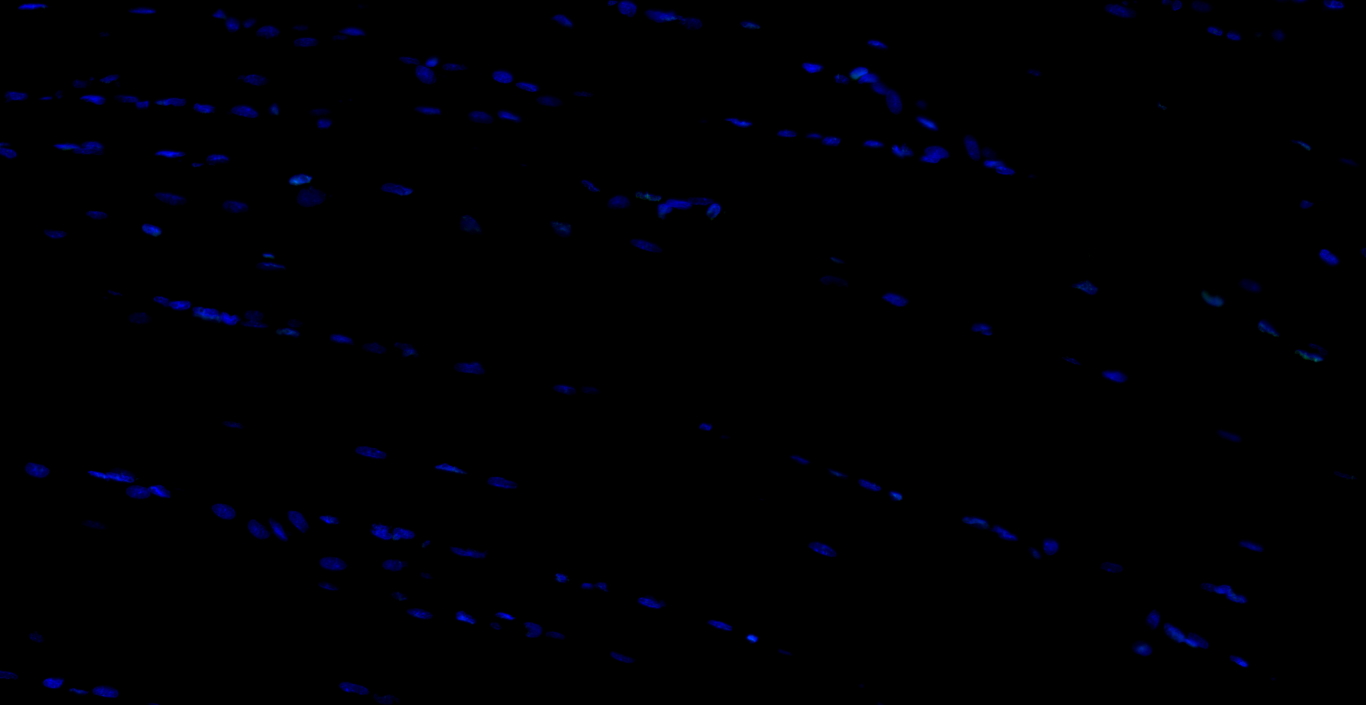

Supplement: Supplementary file 2 [file DataSheet4.ZIP › Supplemental materials 1/TUNEL/IR/1-3 merge.jpg]

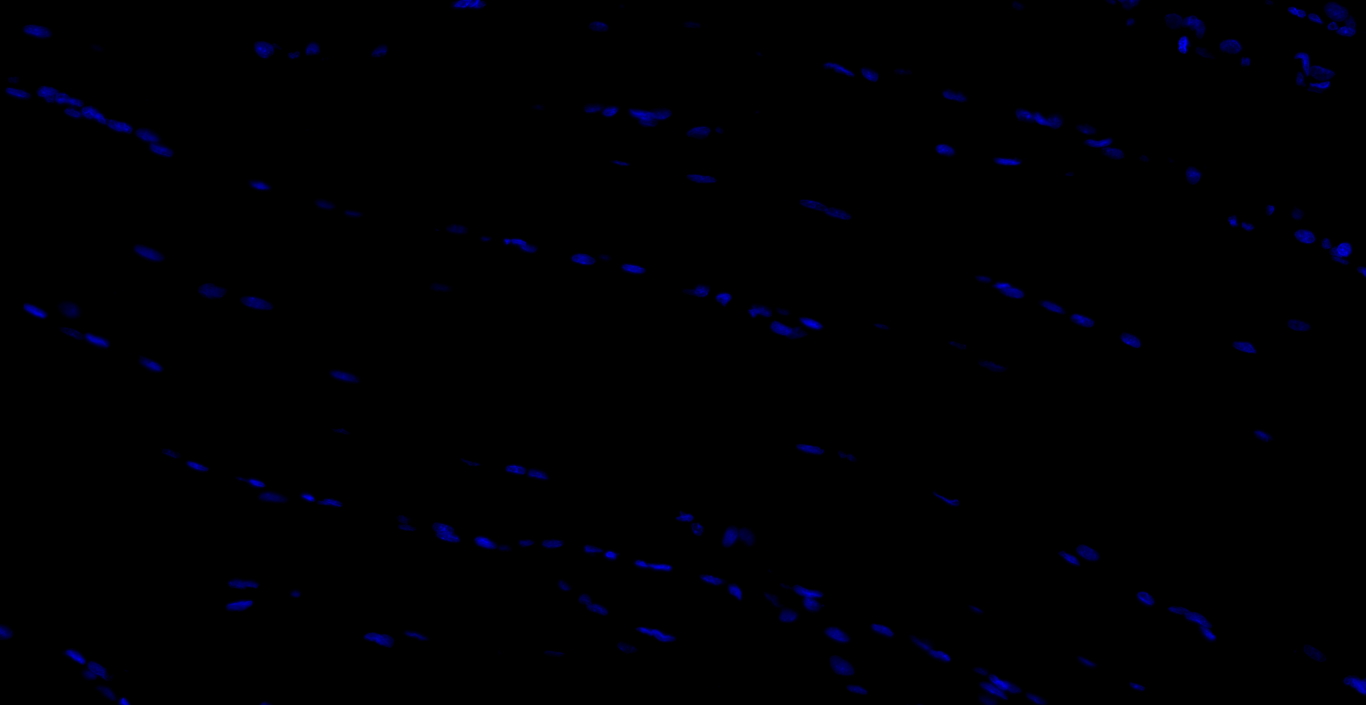

Supplement: Supplementary file 2 [file DataSheet4.ZIP › Supplemental materials 1/TUNEL/IR/2-1 DAPI.jpg]

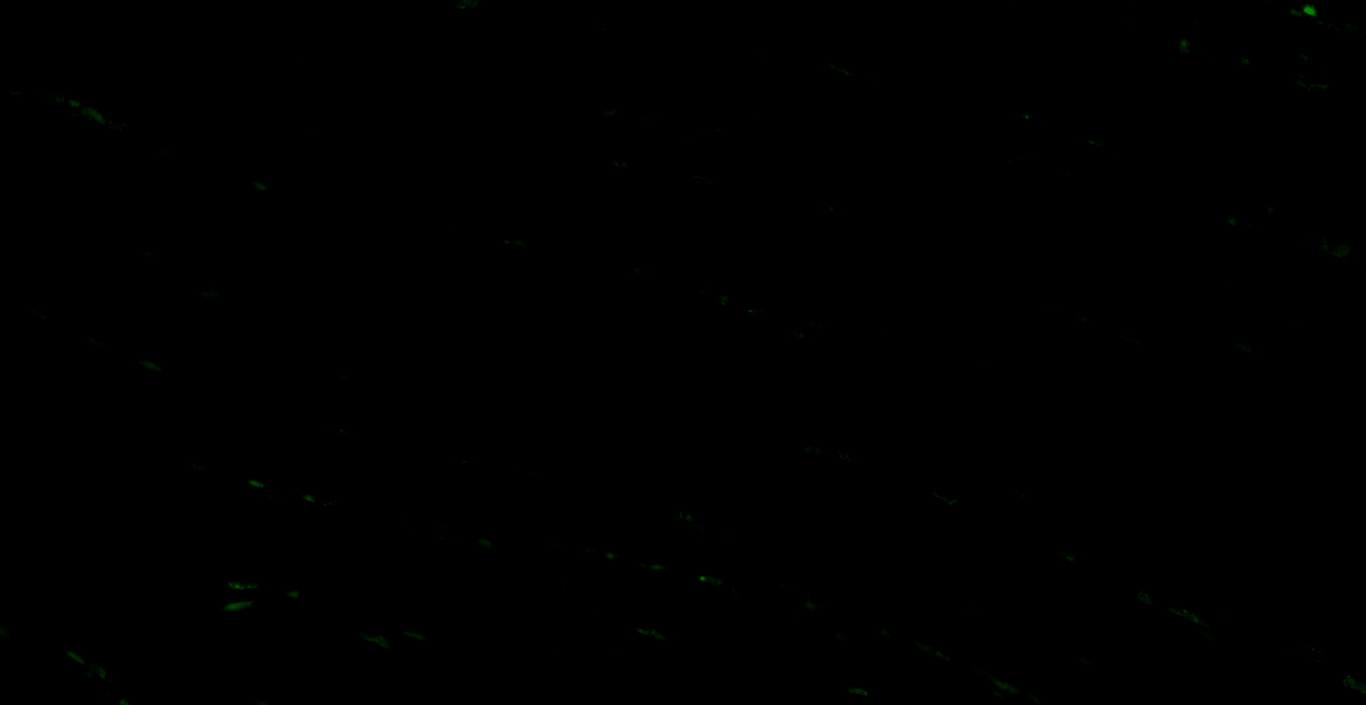

Supplement: Supplementary file 2 [file DataSheet4.ZIP › Supplemental materials 1/TUNEL/IR/2-1 TUNEL.jpg]

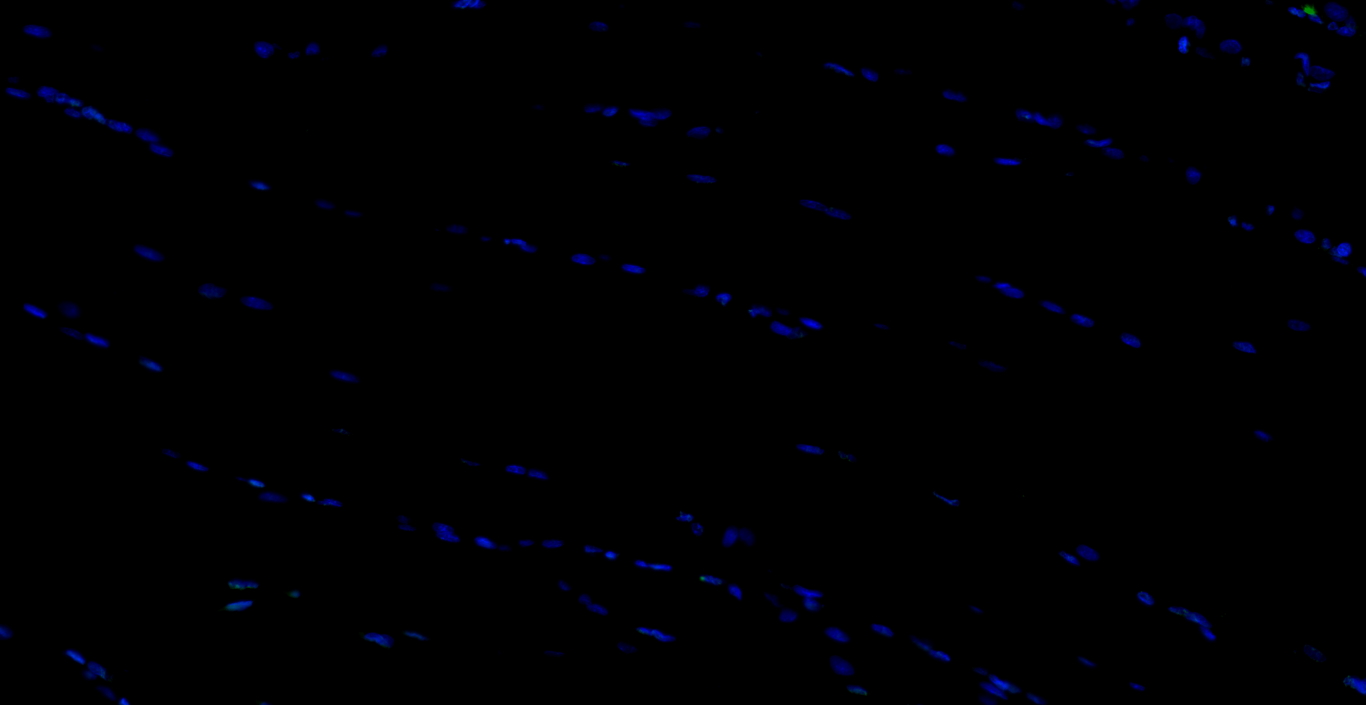

Supplement: Supplementary file 2 [file DataSheet4.ZIP › Supplemental materials 1/TUNEL/IR/2-1 merge.jpg]

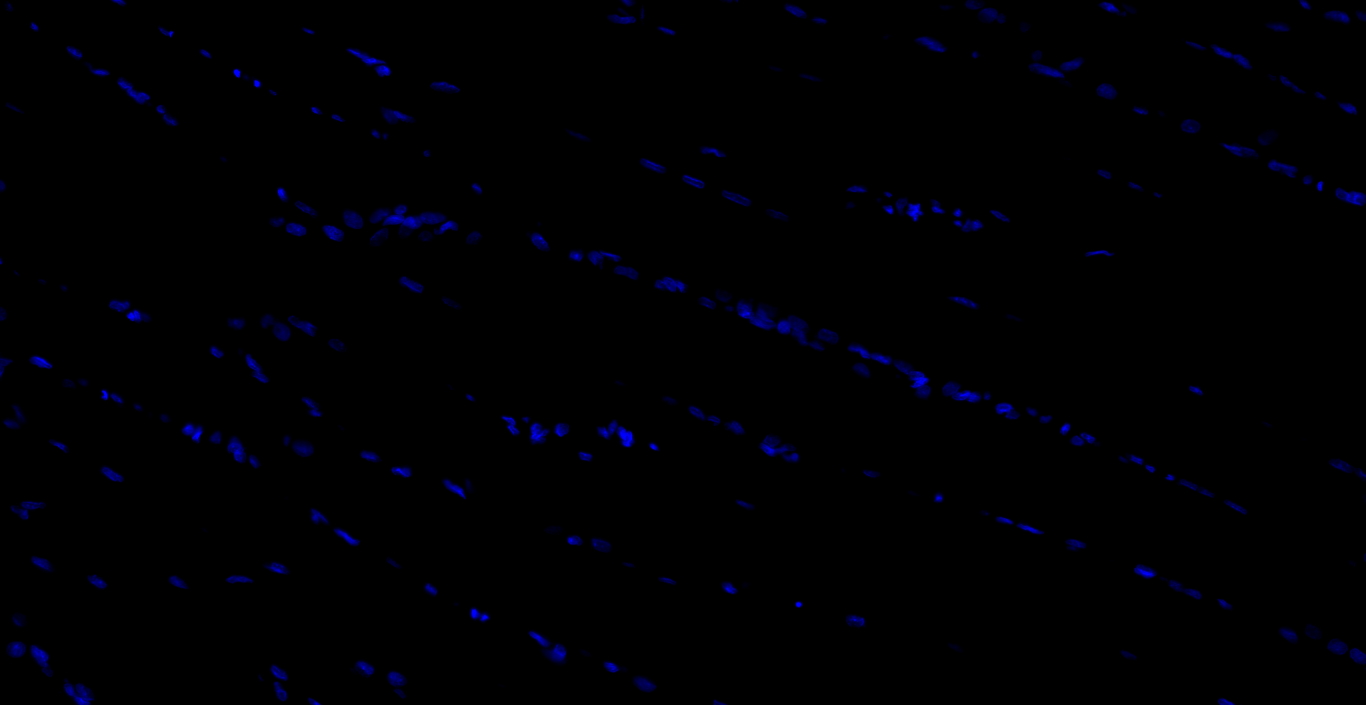

Supplement: Supplementary file 2 [file DataSheet4.ZIP › Supplemental materials 1/TUNEL/IR/2-2 DAPI.jpg]

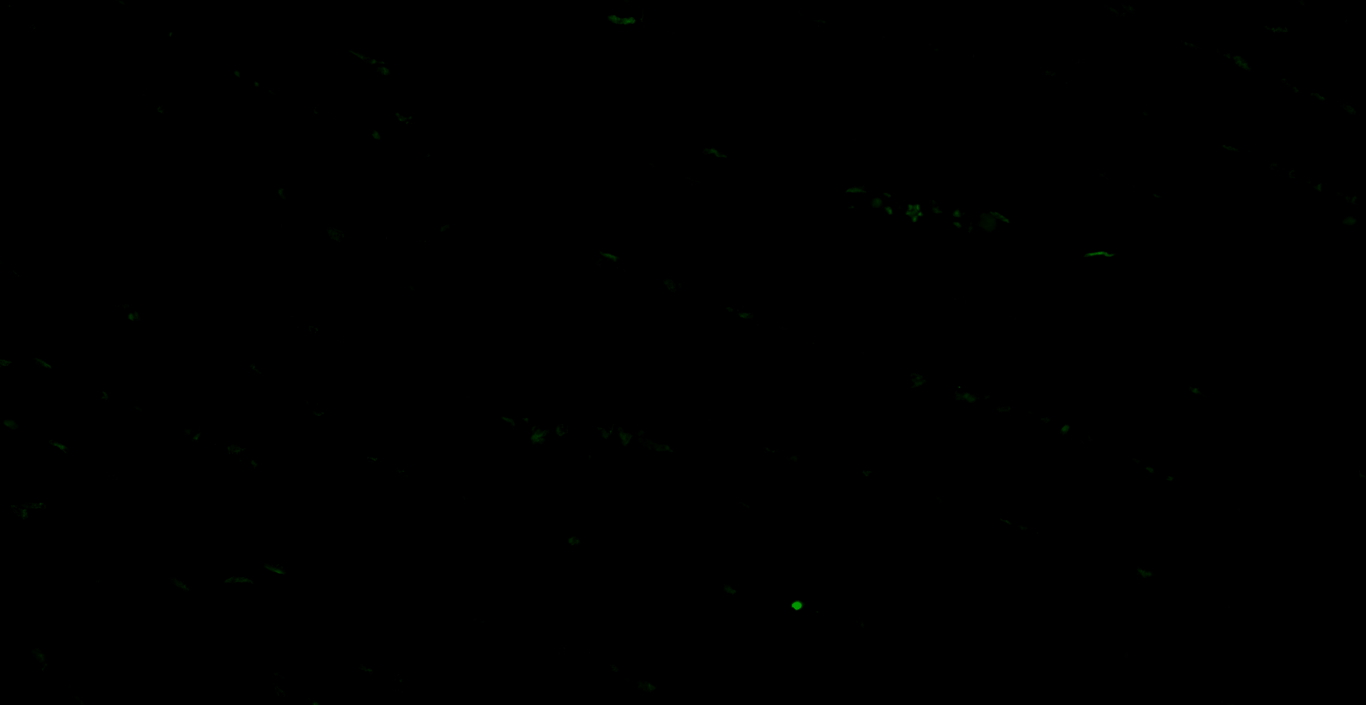

Supplement: Supplementary file 2 [file DataSheet4.ZIP › Supplemental materials 1/TUNEL/IR/2-2 TUNEL.jpg]

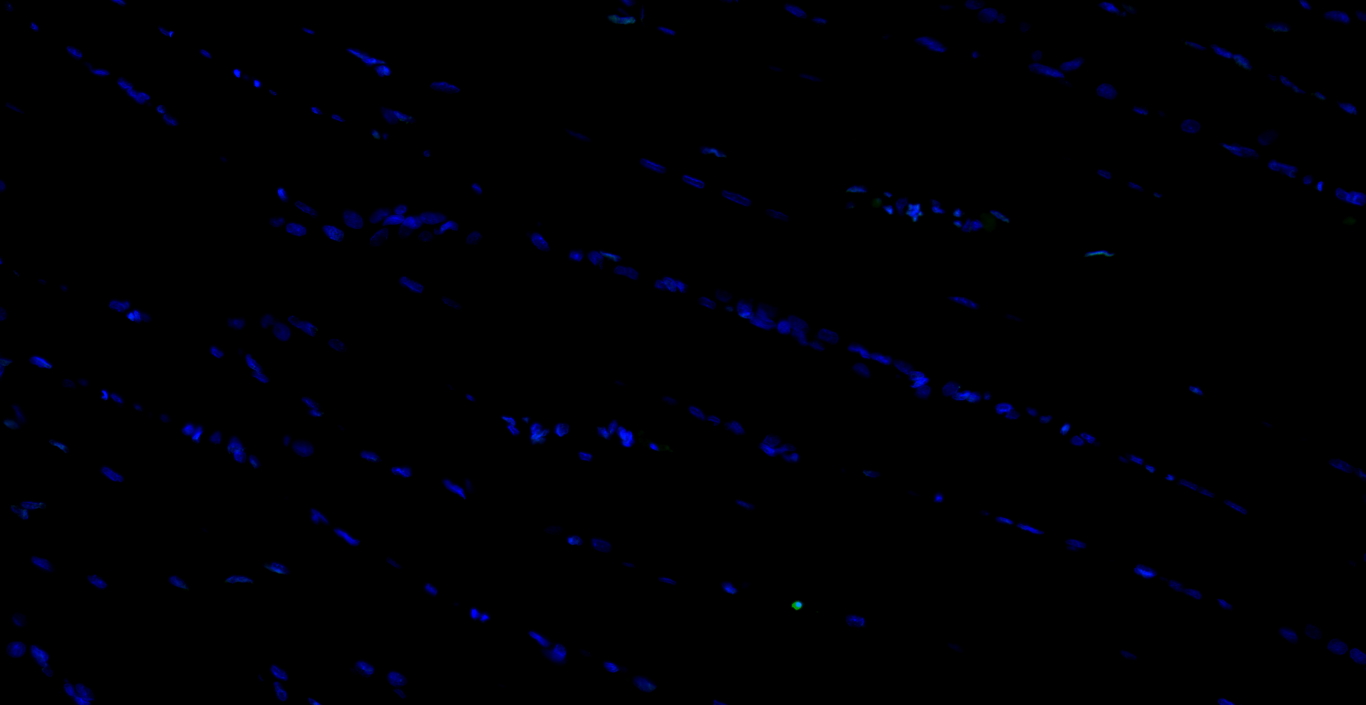

Supplement: Supplementary file 2 [file DataSheet4.ZIP › Supplemental materials 1/TUNEL/IR/2-2 merge.jpg]

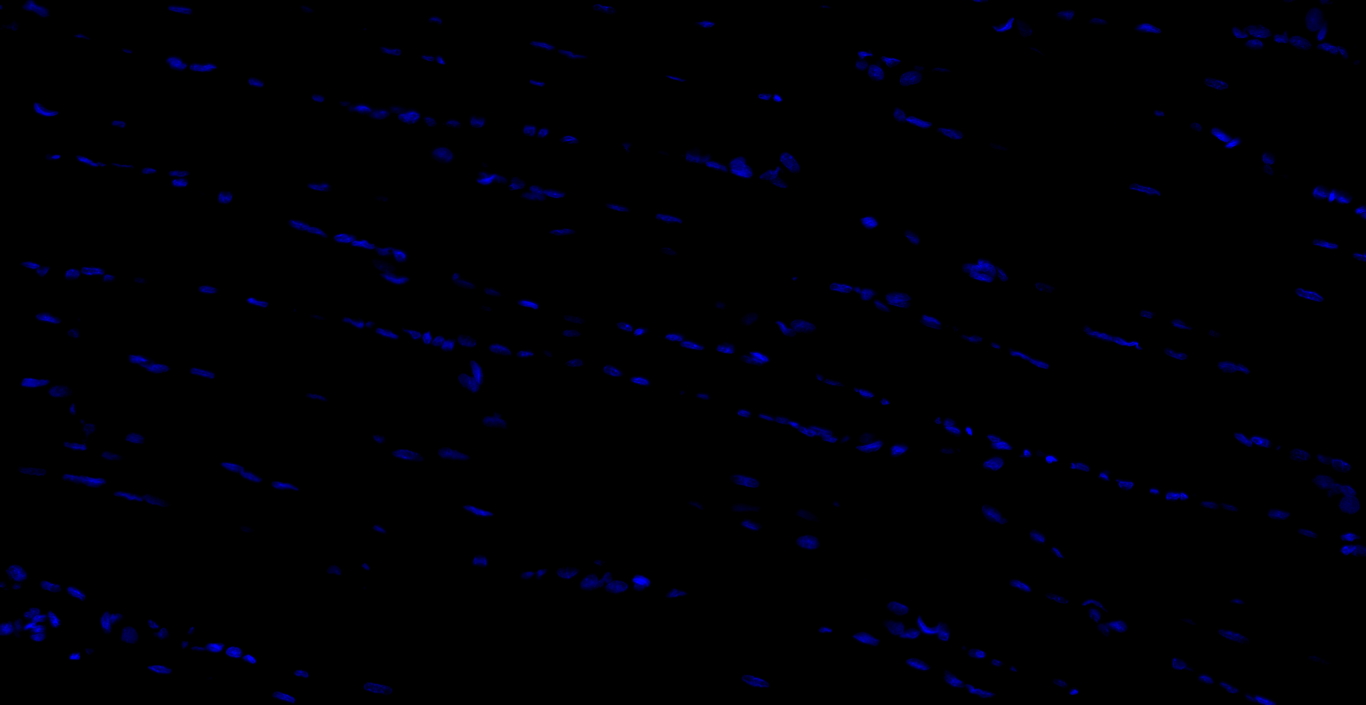

Supplement: Supplementary file 2 [file DataSheet4.ZIP › Supplemental materials 1/TUNEL/IR/2-3 DAPI.jpg]

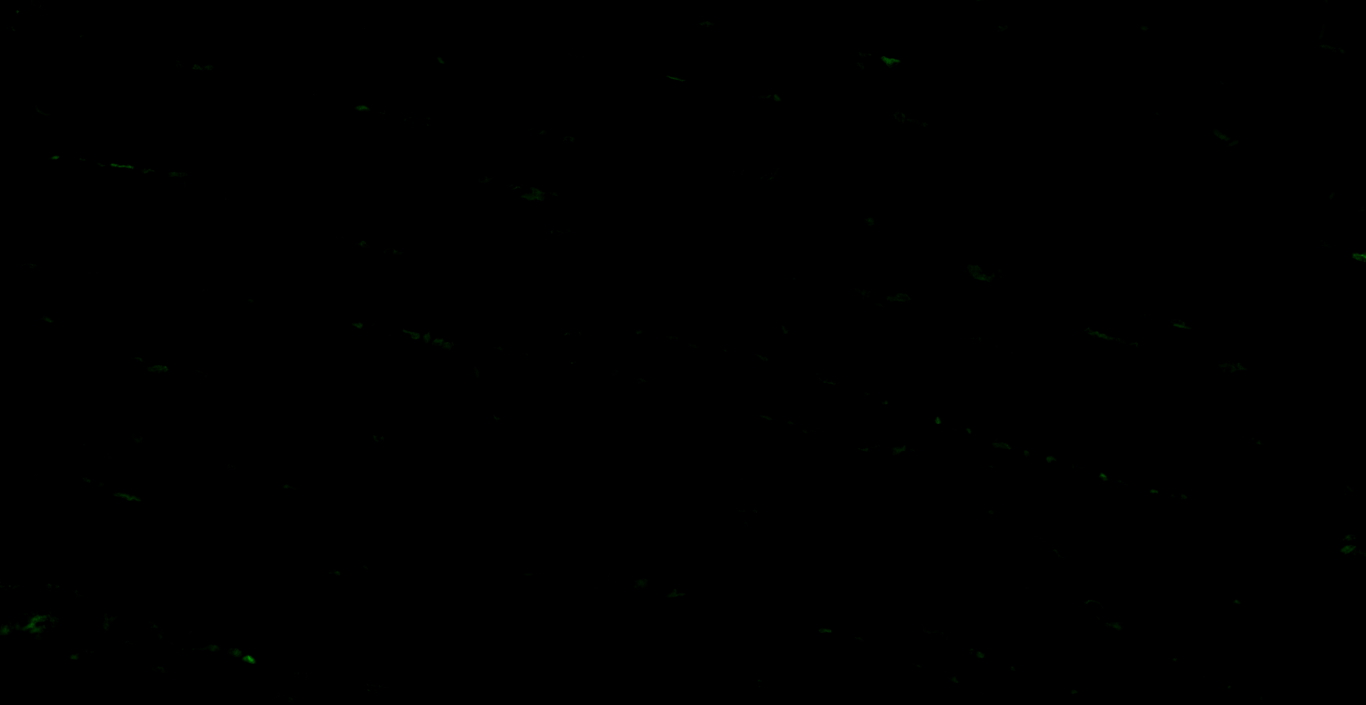

Supplement: Supplementary file 2 [file DataSheet4.ZIP › Supplemental materials 1/TUNEL/IR/2-3 TUNEL.jpg]

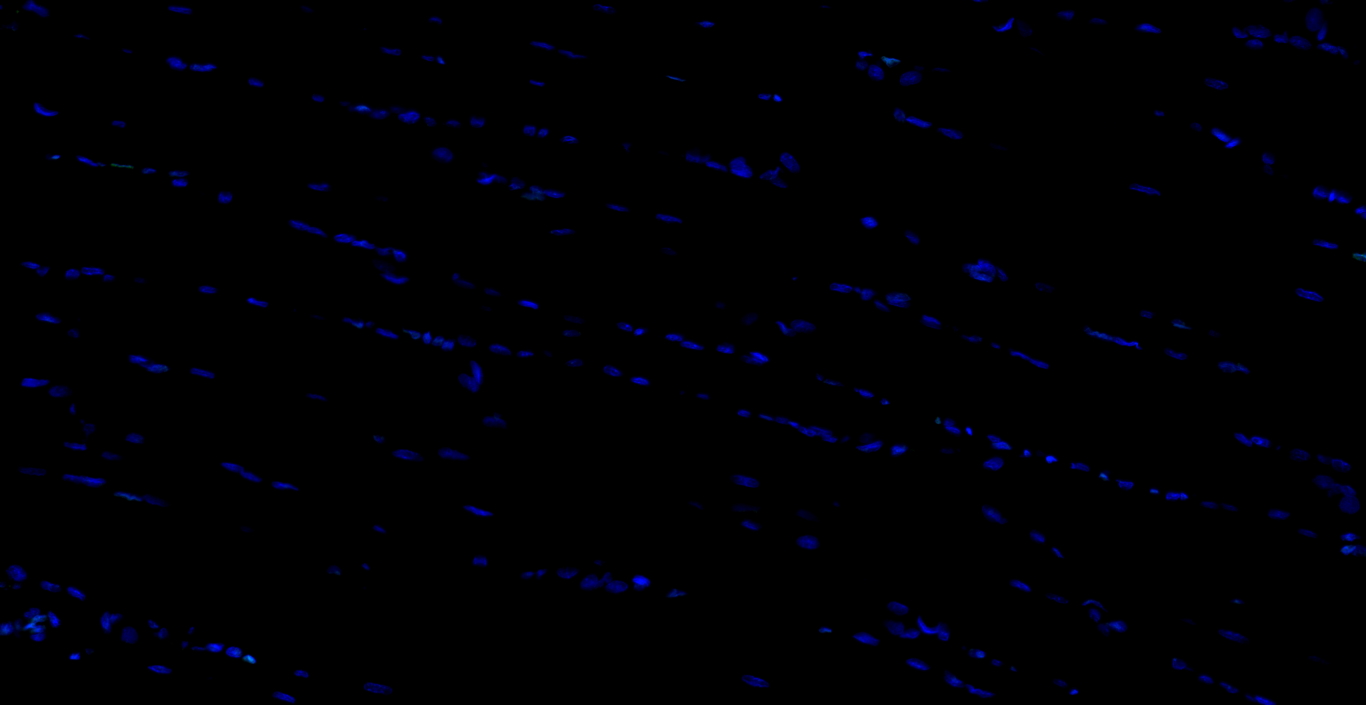

Supplement: Supplementary file 2 [file DataSheet4.ZIP › Supplemental materials 1/TUNEL/IR/2-3 merge.jpg]

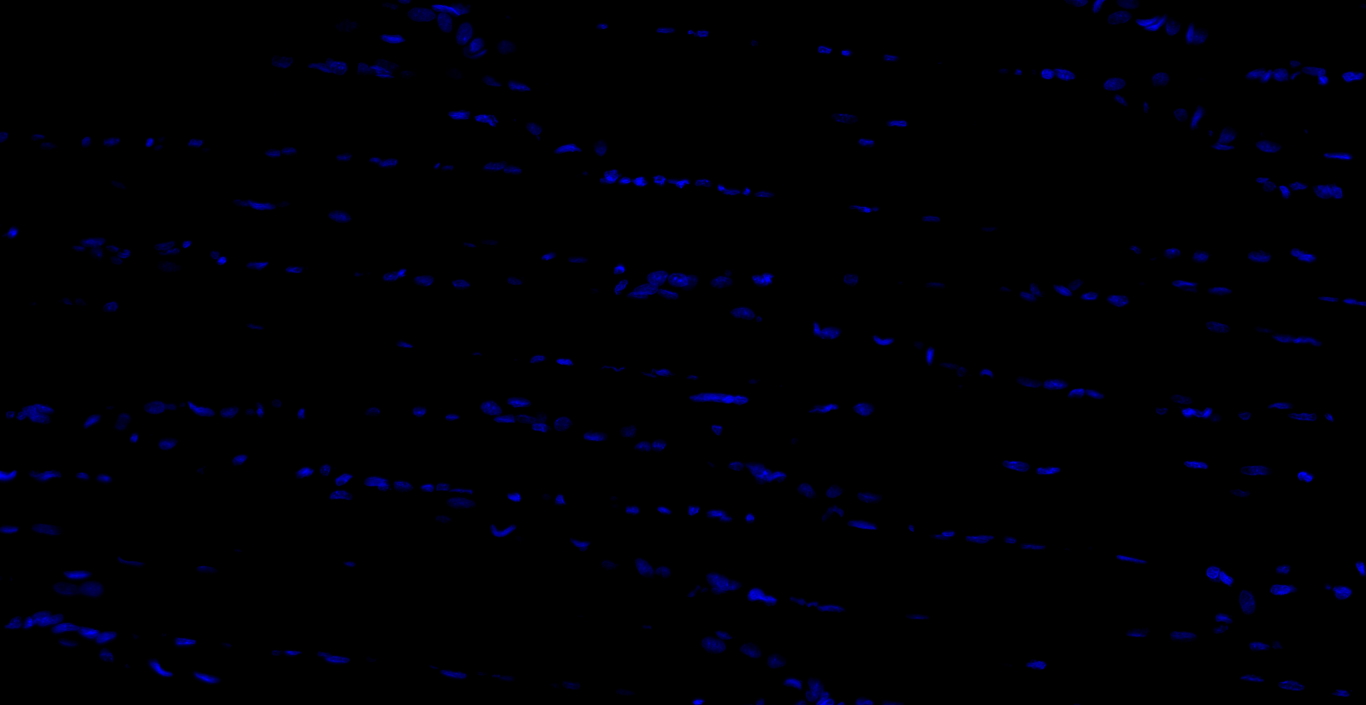

Supplement: Supplementary file 2 [file DataSheet4.ZIP › Supplemental materials 1/TUNEL/IR/3-1 DAPI.jpg]

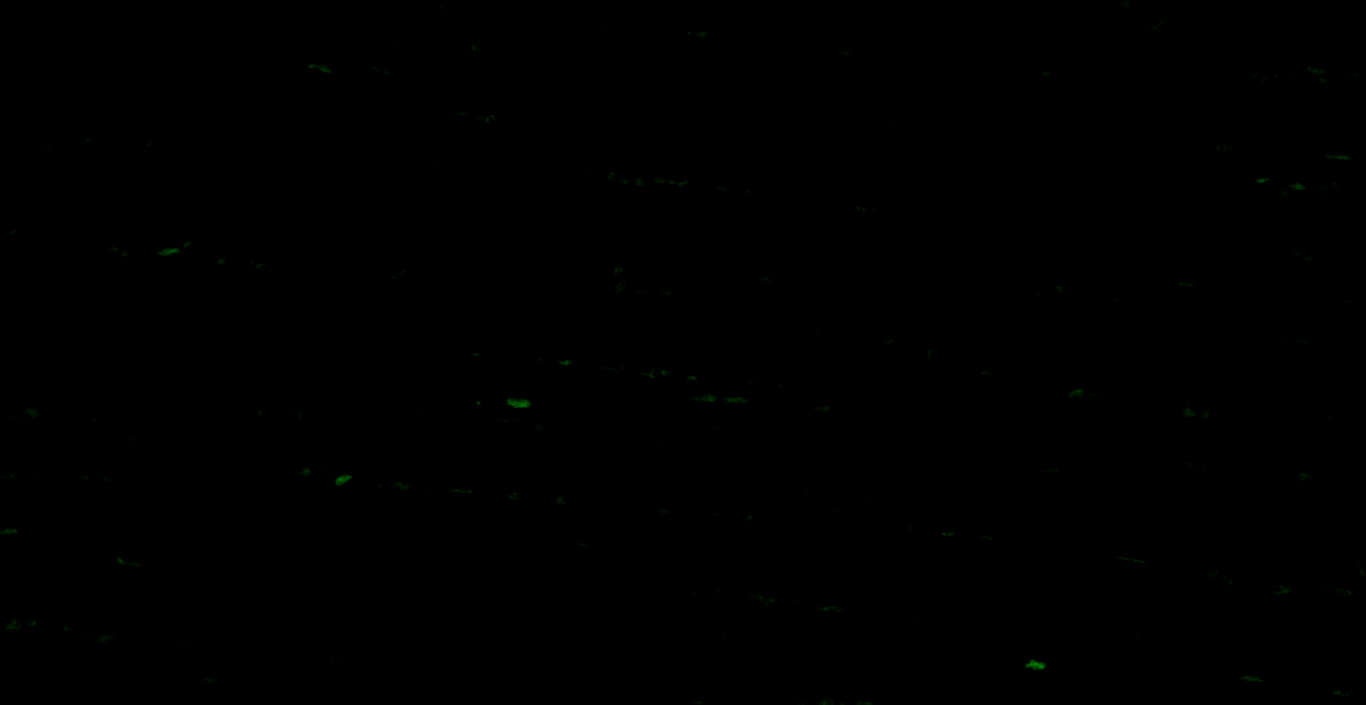

Supplement: Supplementary file 2 [file DataSheet4.ZIP › Supplemental materials 1/TUNEL/IR/3-1 TUNEL.jpg]

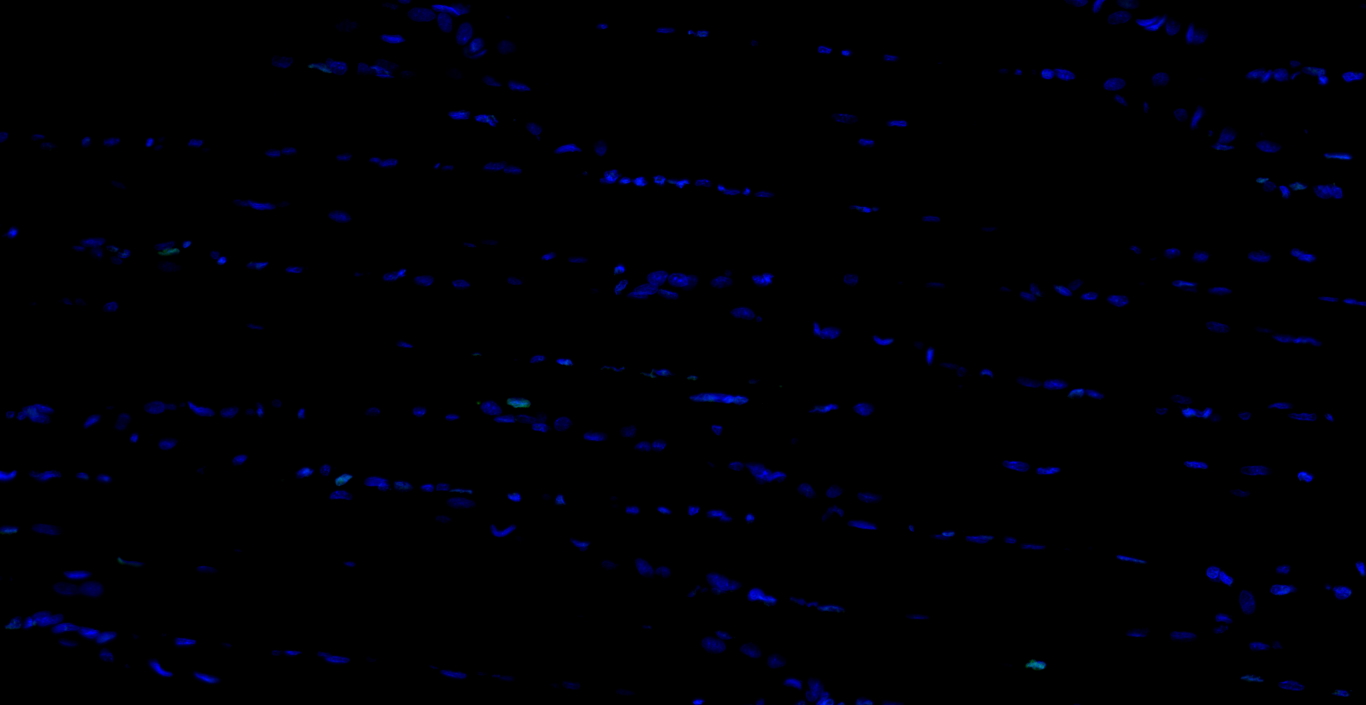

Supplement: Supplementary file 2 [file DataSheet4.ZIP › Supplemental materials 1/TUNEL/IR/3-1 merge.jpg]

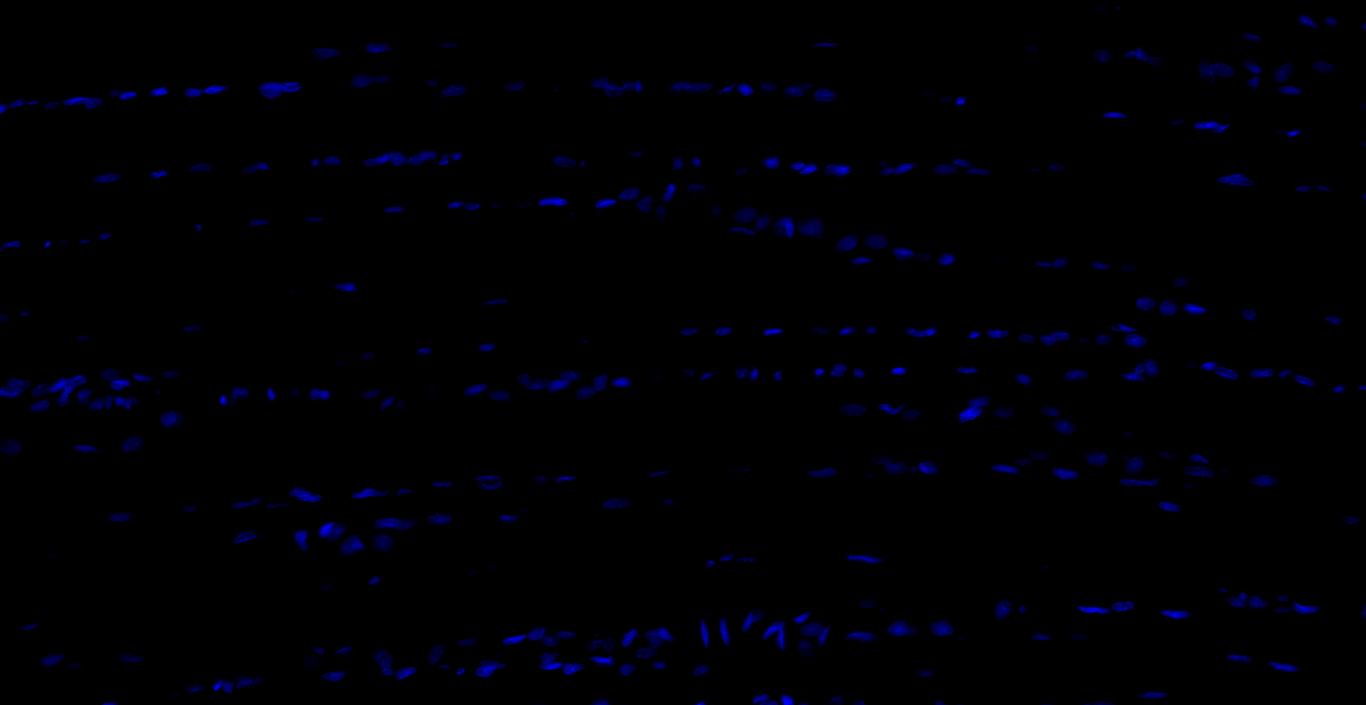

Supplement: Supplementary file 2 [file DataSheet4.ZIP › Supplemental materials 1/TUNEL/IR/3-2 DAPI.jpg]

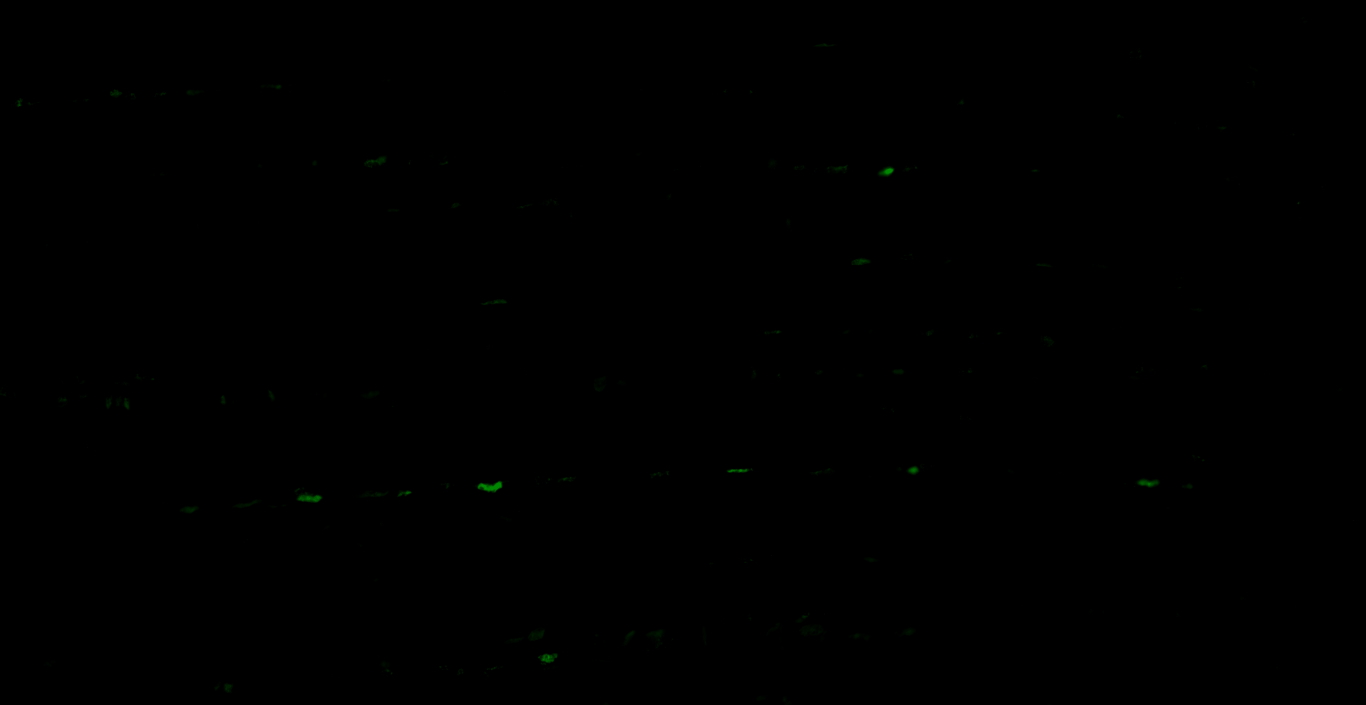

Supplement: Supplementary file 2 [file DataSheet4.ZIP › Supplemental materials 1/TUNEL/IR/3-2 TUNEL.jpg]

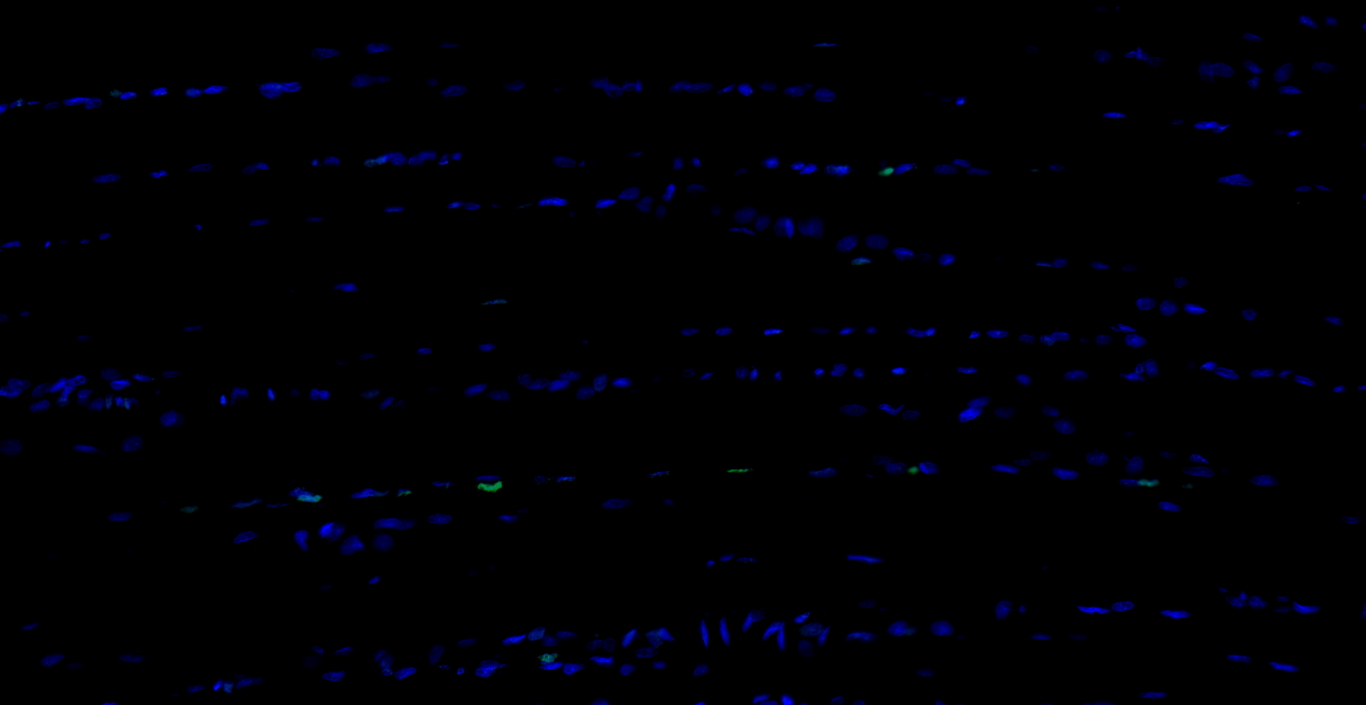

Supplement: Supplementary file 2 [file DataSheet4.ZIP › Supplemental materials 1/TUNEL/IR/3-2 merge.jpg]

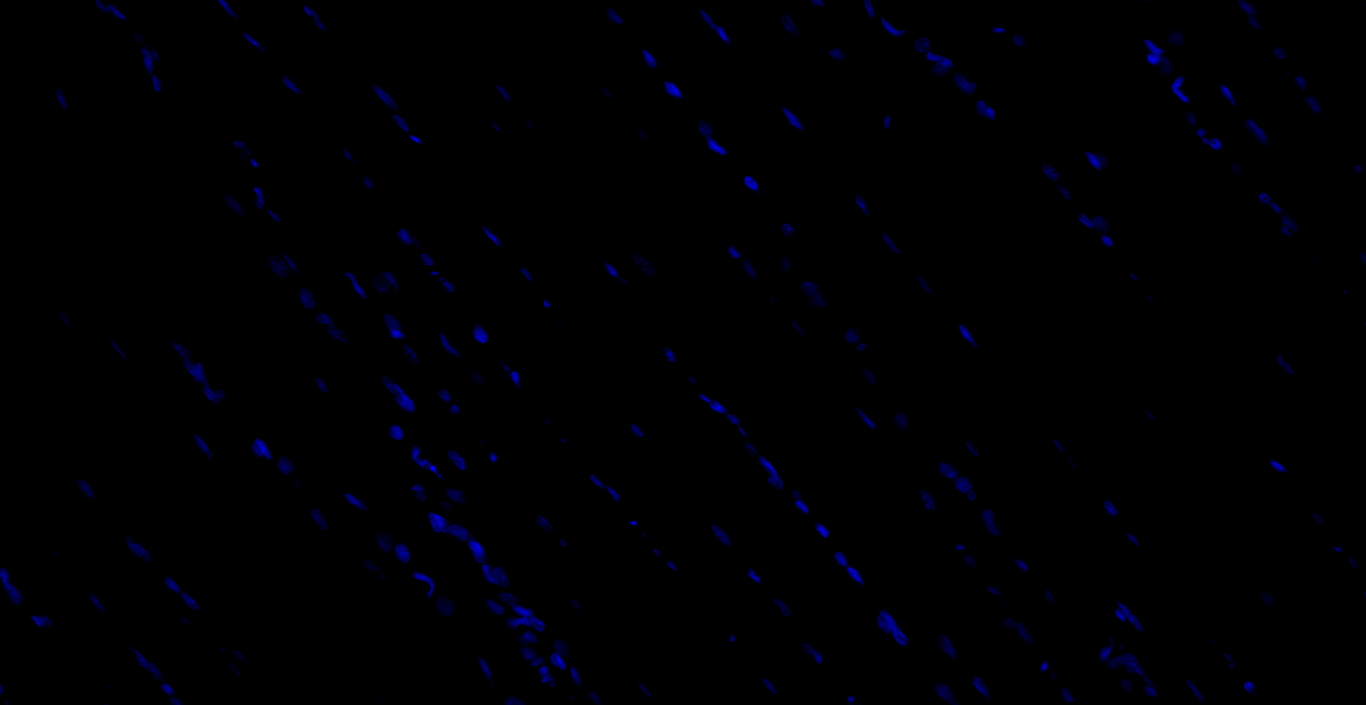

Supplement: Supplementary file 2 [file DataSheet4.ZIP › Supplemental materials 1/TUNEL/IR/3-3 DAPI.jpg]

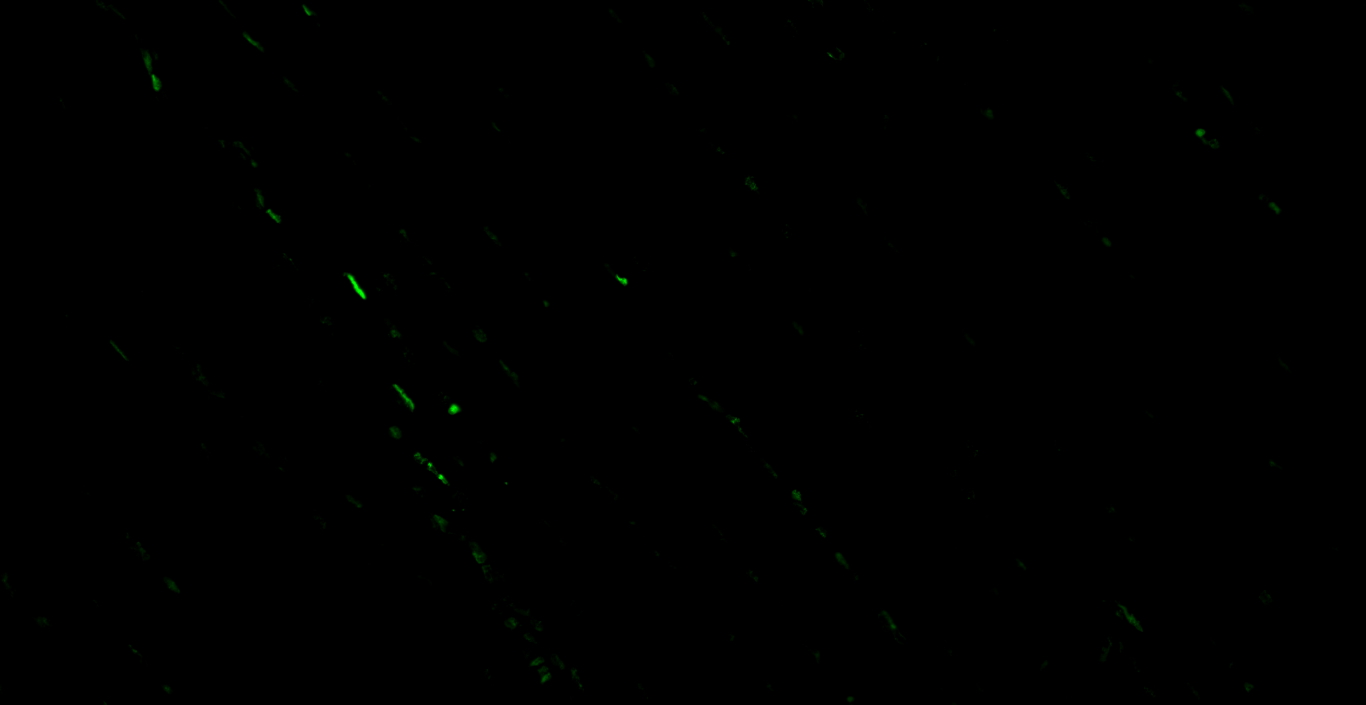

Supplement: Supplementary file 2 [file DataSheet4.ZIP › Supplemental materials 1/TUNEL/IR/3-3 TUNEL.jpg]

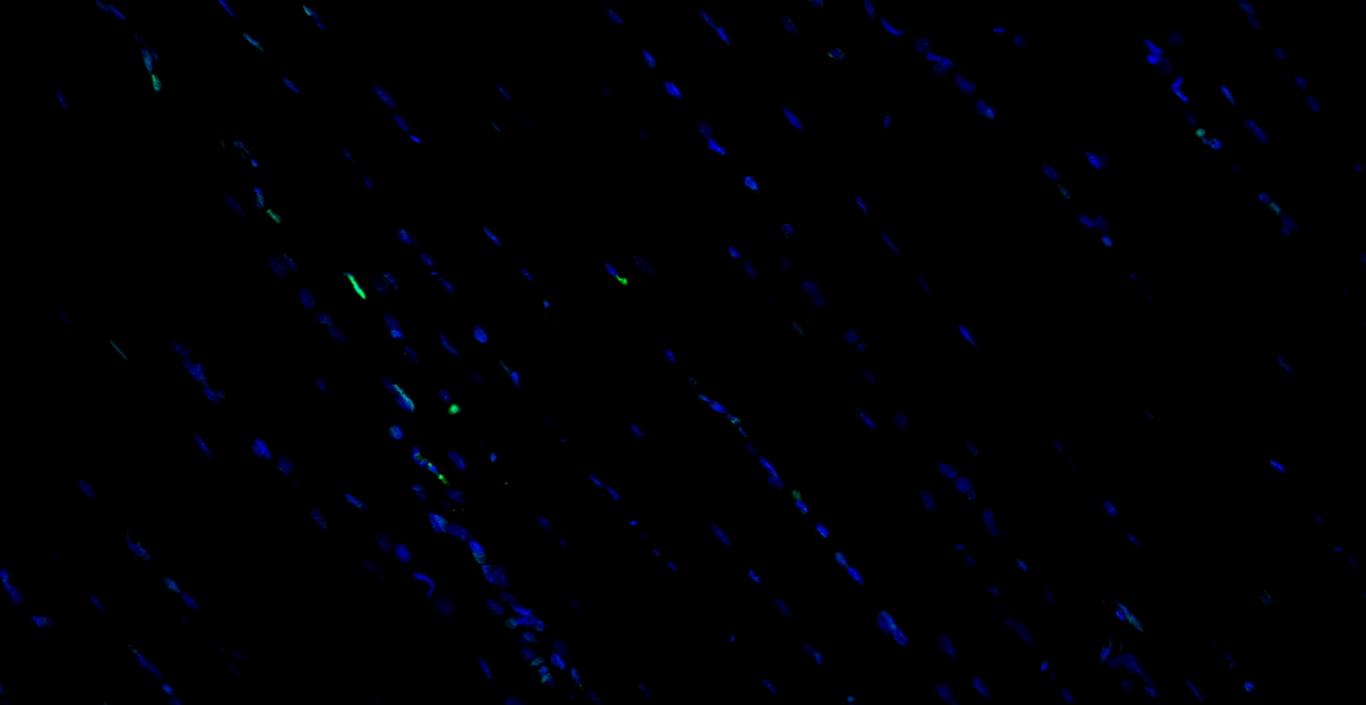

Supplement: Supplementary file 2 [file DataSheet4.ZIP › Supplemental materials 1/TUNEL/IR/3-3 merge.jpg]

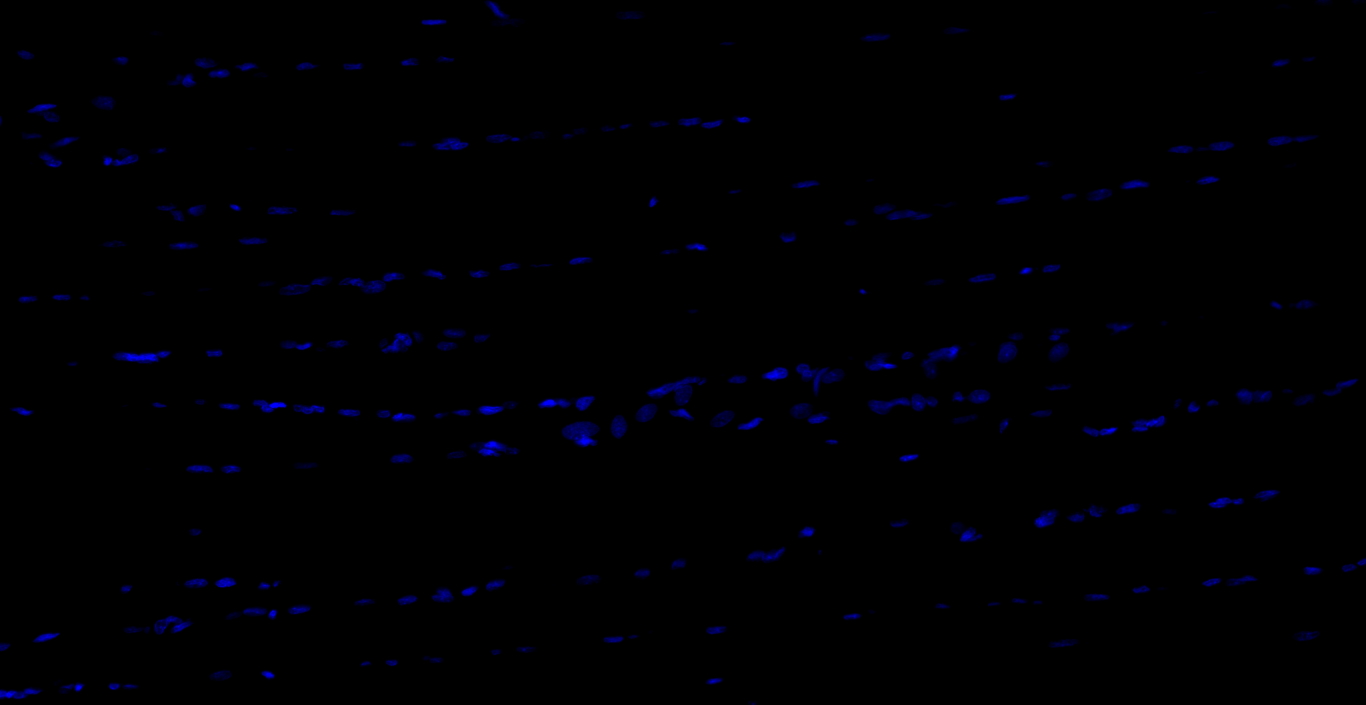

Supplement: Supplementary file 2 [file DataSheet4.ZIP › Supplemental materials 1/TUNEL/IR/4-1 DAPI.jpg]

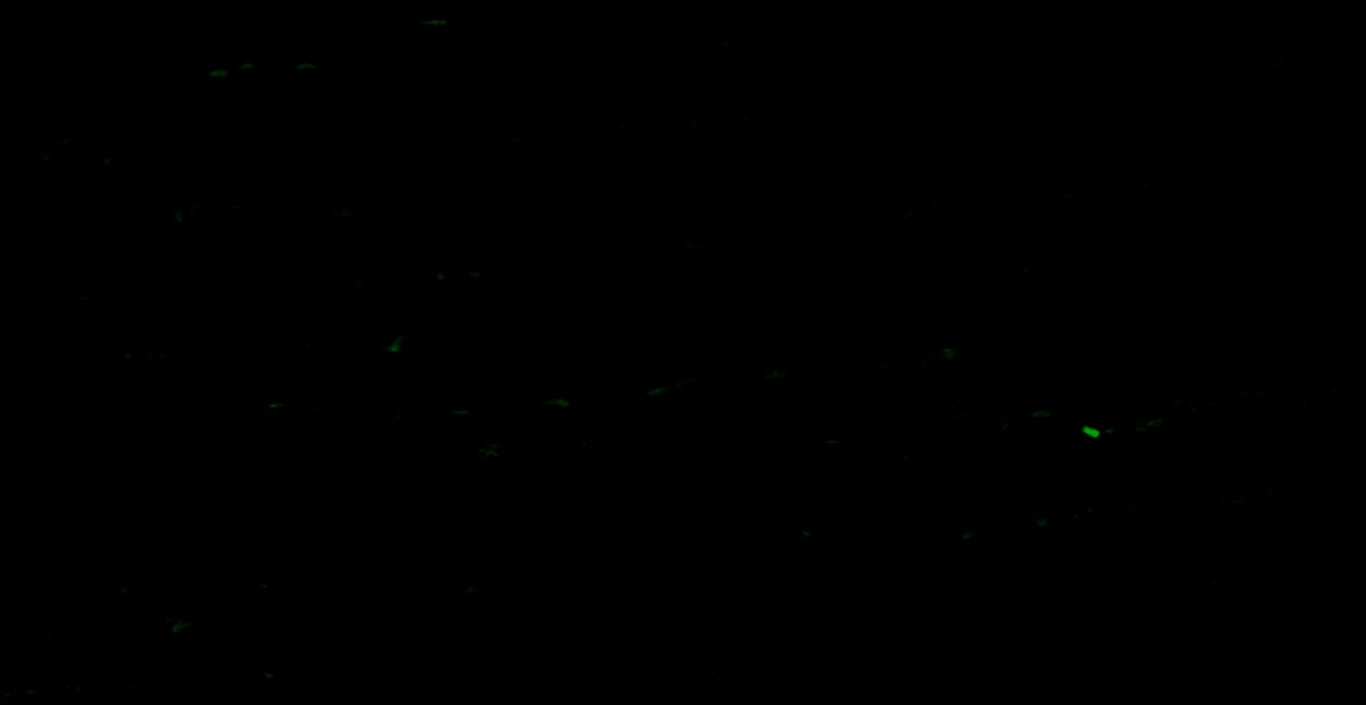

Supplement: Supplementary file 2 [file DataSheet4.ZIP › Supplemental materials 1/TUNEL/IR/4-1 TUNEL.jpg]

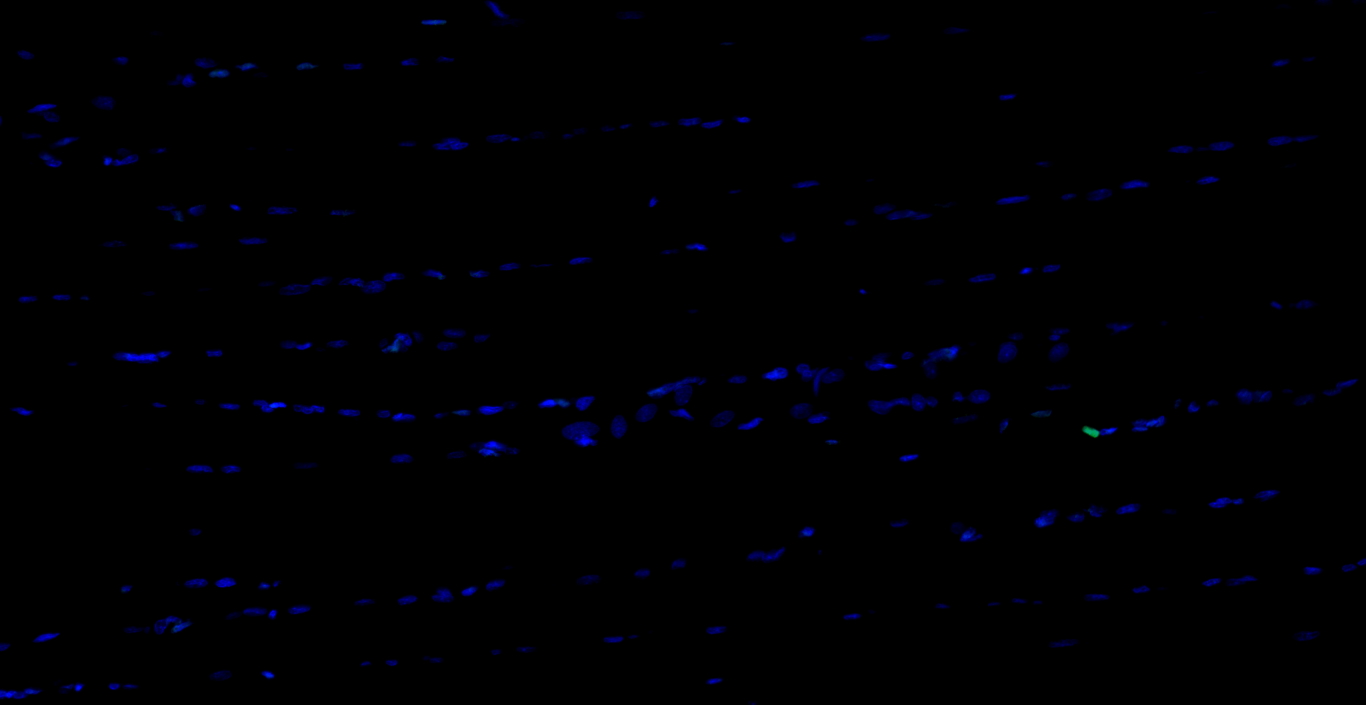

Supplement: Supplementary file 2 [file DataSheet4.ZIP › Supplemental materials 1/TUNEL/IR/4-1 merge.jpg]

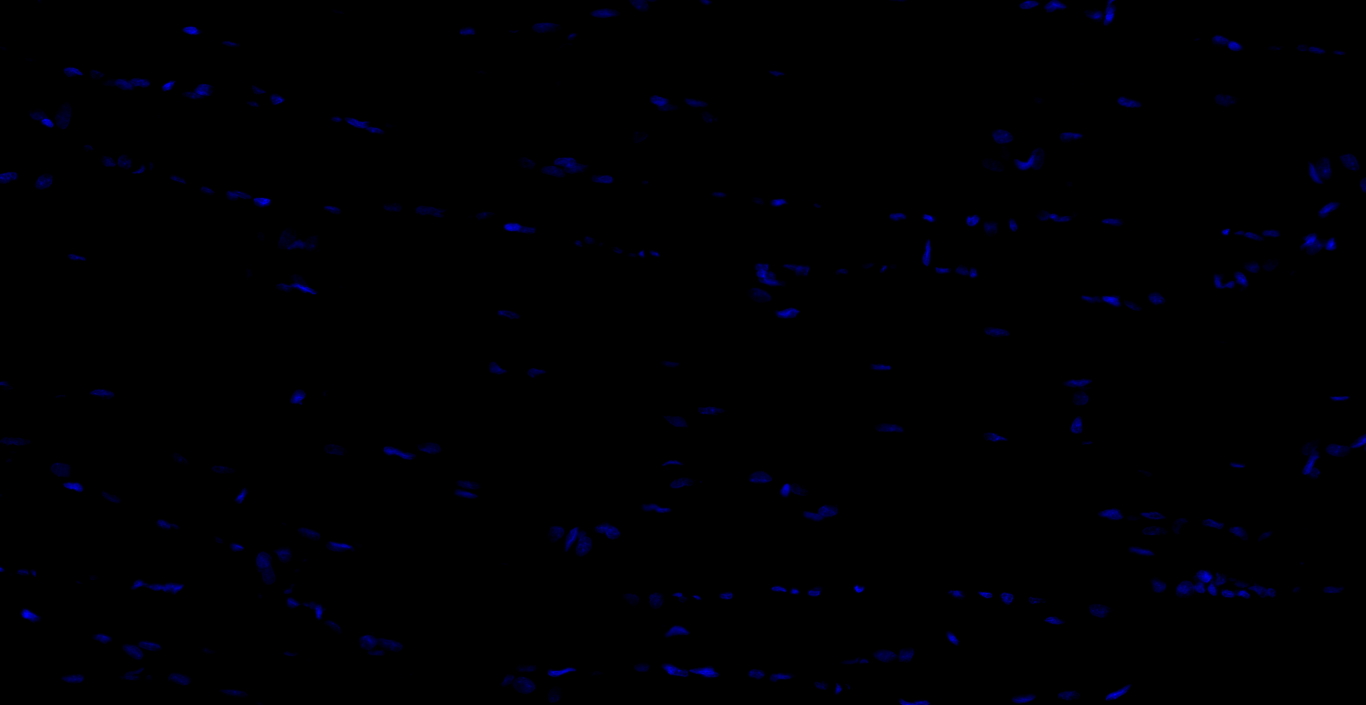

Supplement: Supplementary file 2 [file DataSheet4.ZIP › Supplemental materials 1/TUNEL/IR/4-2 DAPI.jpg]

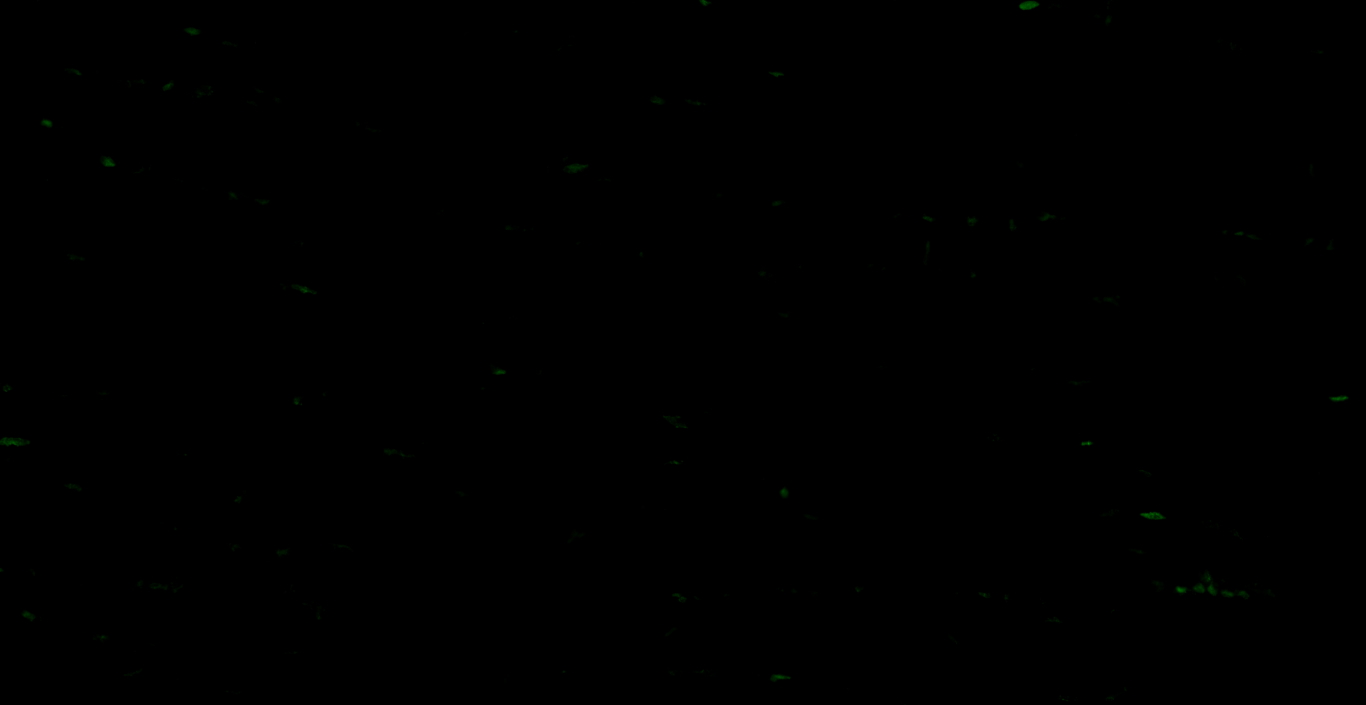

Supplement: Supplementary file 2 [file DataSheet4.ZIP › Supplemental materials 1/TUNEL/IR/4-2 TUNEL.jpg]

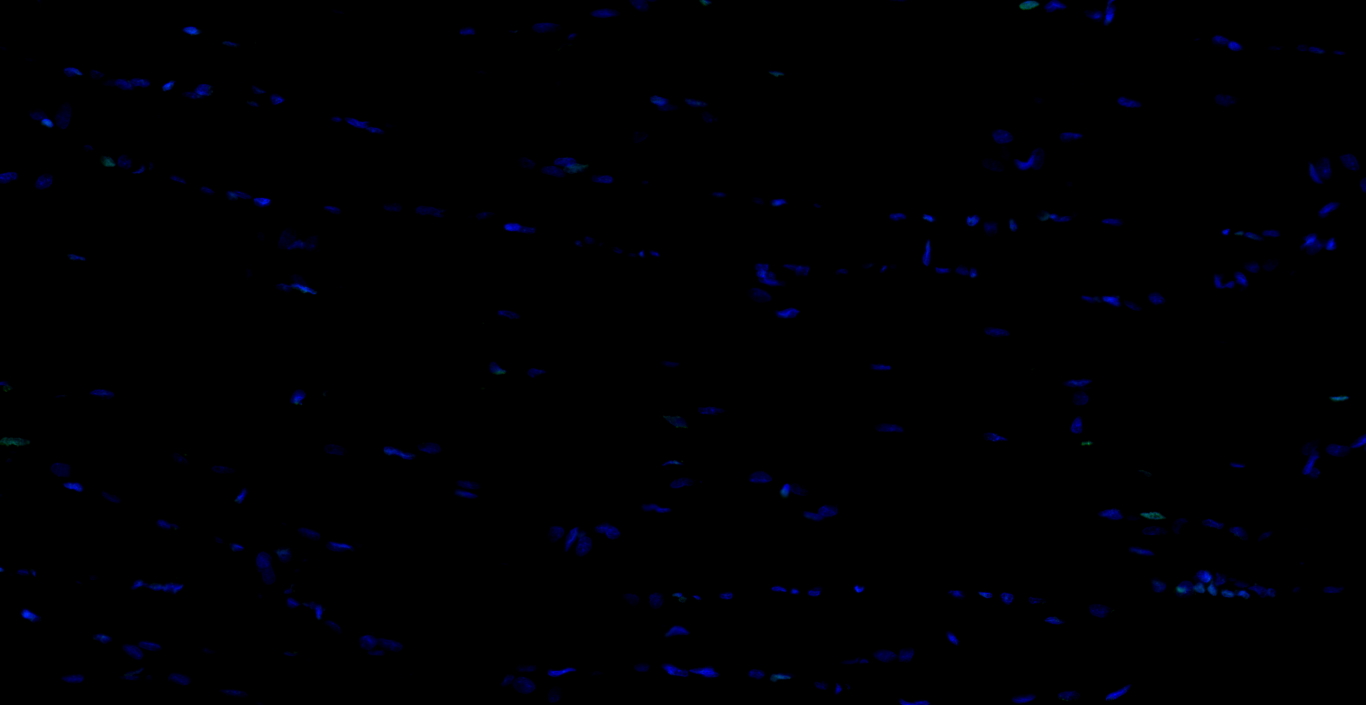

Supplement: Supplementary file 2 [file DataSheet4.ZIP › Supplemental materials 1/TUNEL/IR/4-2 merge.jpg]

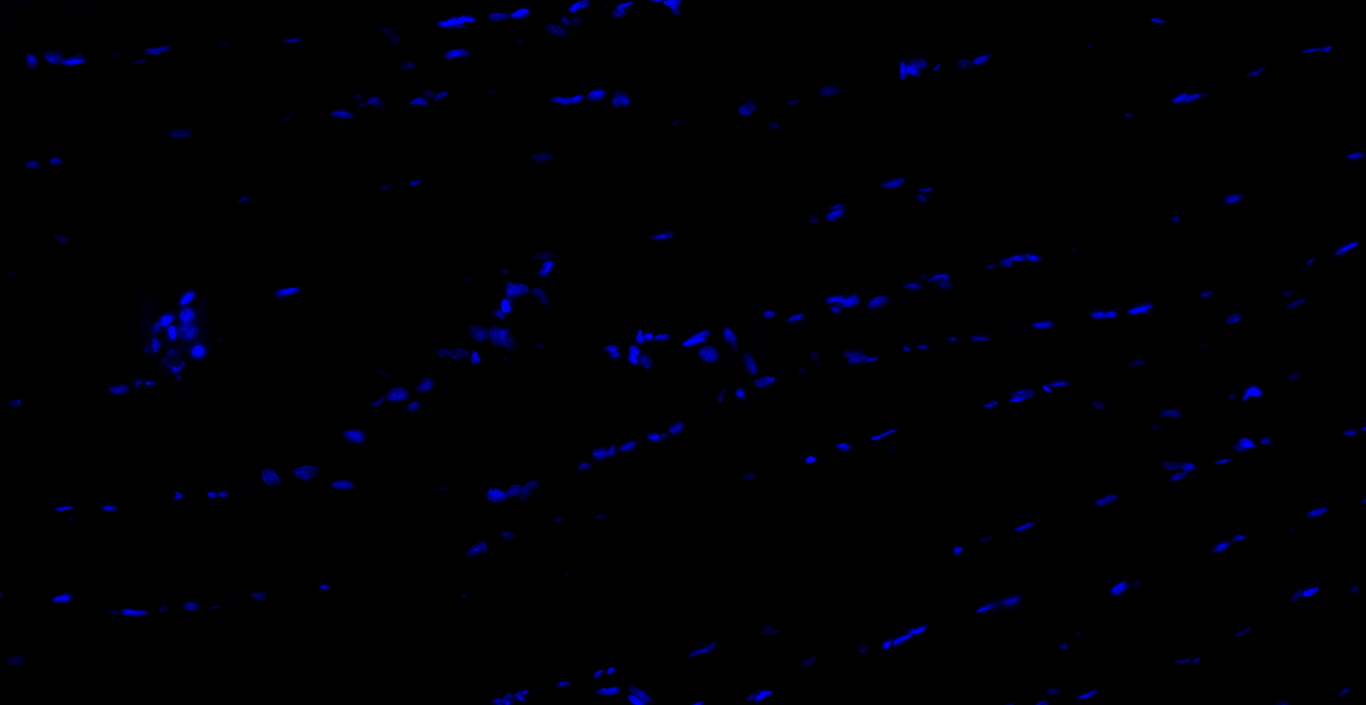

Supplement: Supplementary file 2 [file DataSheet4.ZIP › Supplemental materials 1/TUNEL/IR/4-3 DAPI.jpg]

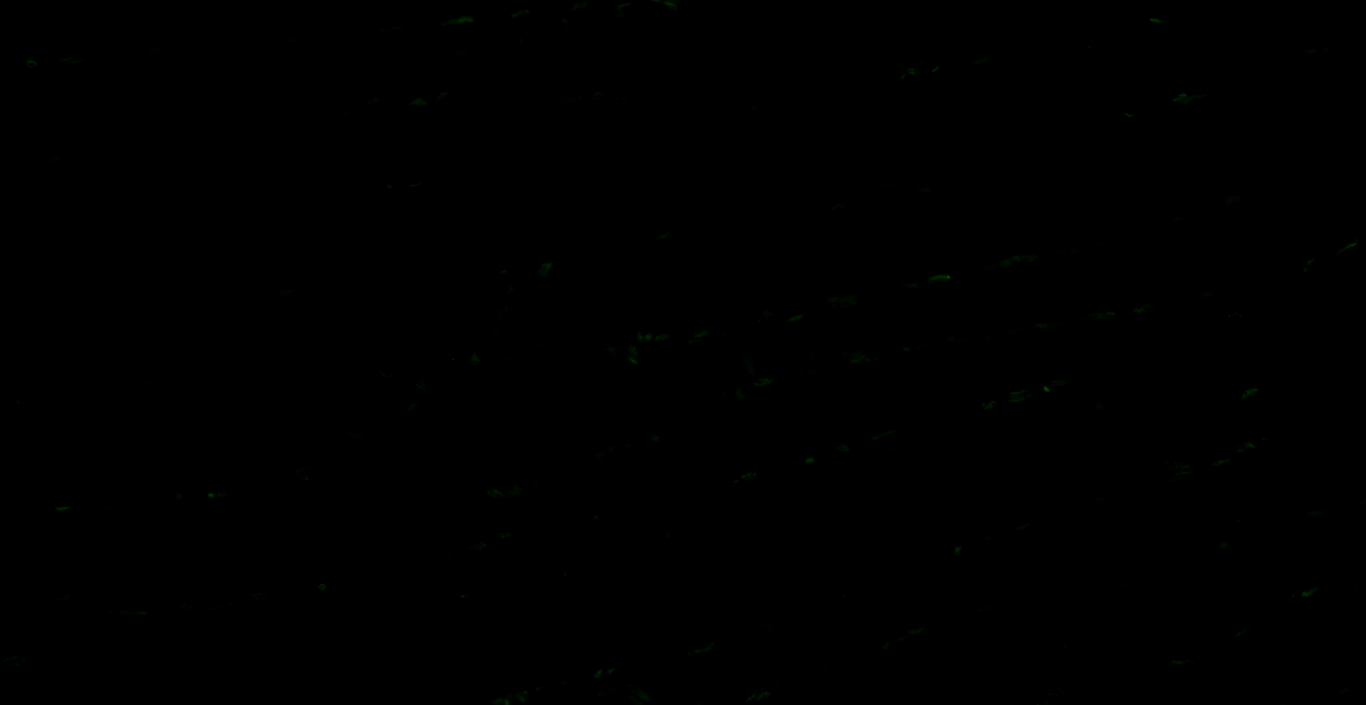

Supplement: Supplementary file 2 [file DataSheet4.ZIP › Supplemental materials 1/TUNEL/IR/4-3 TUNEL.jpg]

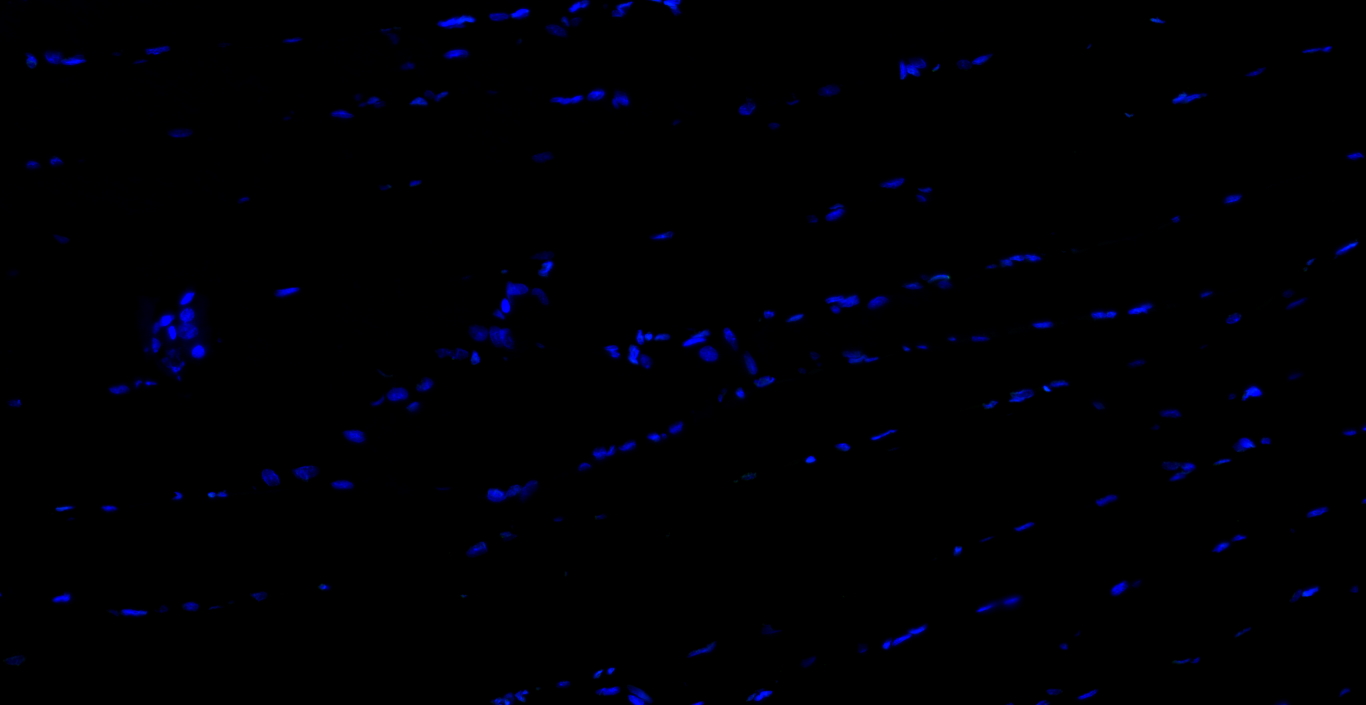

Supplement: Supplementary file 2 [file DataSheet4.ZIP › Supplemental materials 1/TUNEL/IR/4-3 merge.jpg]

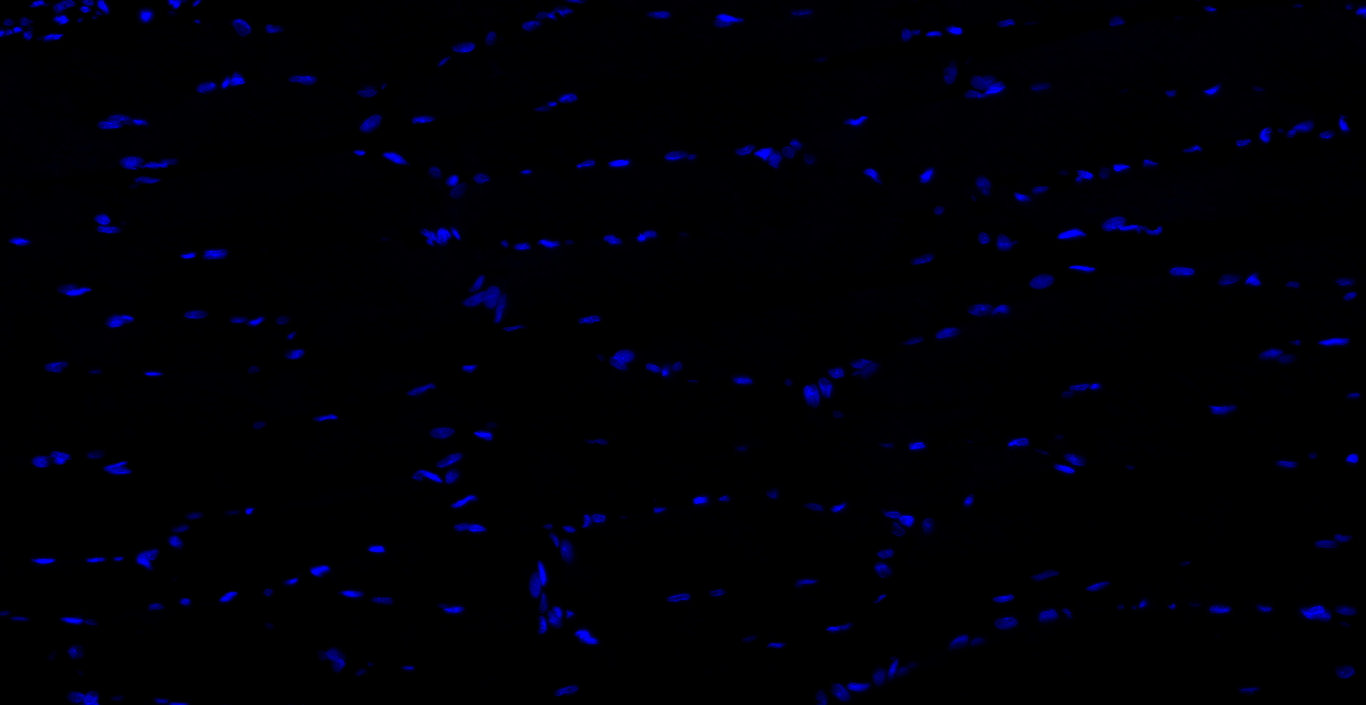

Supplement: Supplementary file 2 [file DataSheet4.ZIP › Supplemental materials 1/TUNEL/IR/5-1 DAPI.jpg]

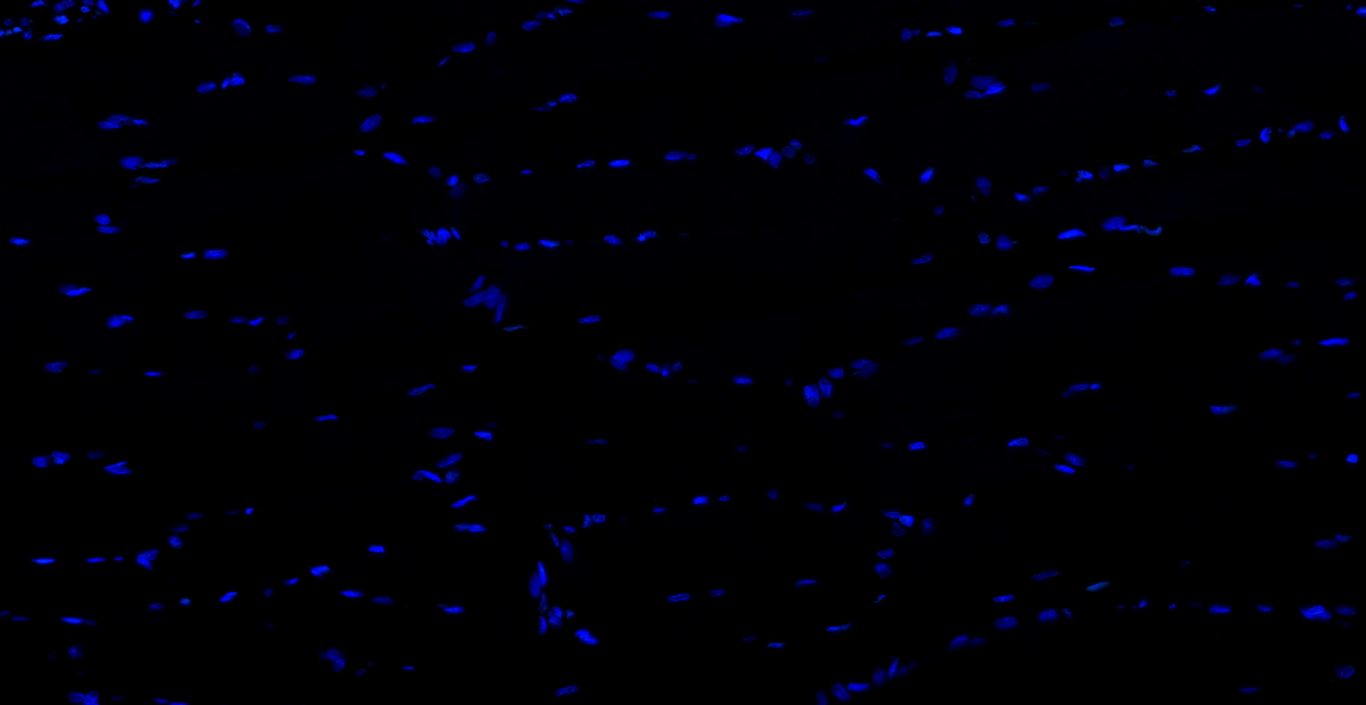

Supplement: Supplementary file 2 [file DataSheet4.ZIP › Supplemental materials 1/TUNEL/IR/5-1 merge.jpg]

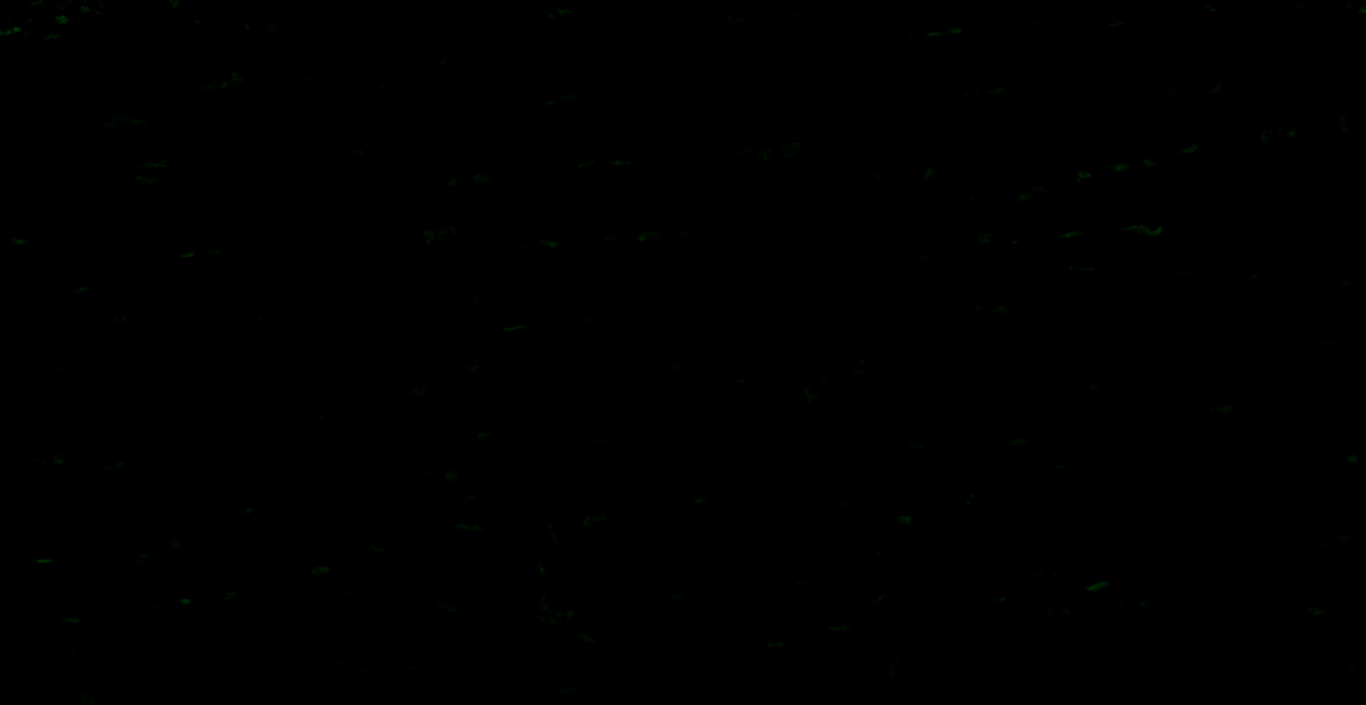

Supplement: Supplementary file 2 [file DataSheet4.ZIP › Supplemental materials 1/TUNEL/IR/5-1TUNEL.jpg]

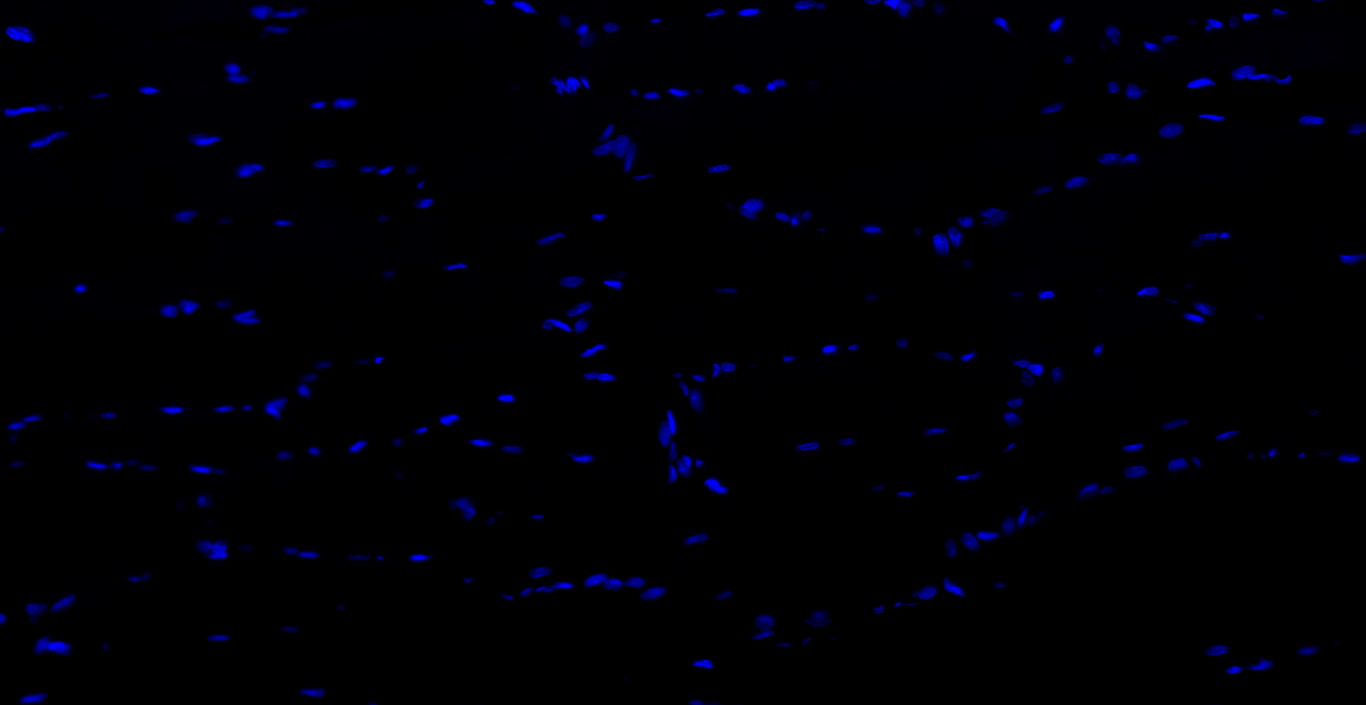

Supplement: Supplementary file 2 [file DataSheet4.ZIP › Supplemental materials 1/TUNEL/IR/5-2 DAPI.jpg]

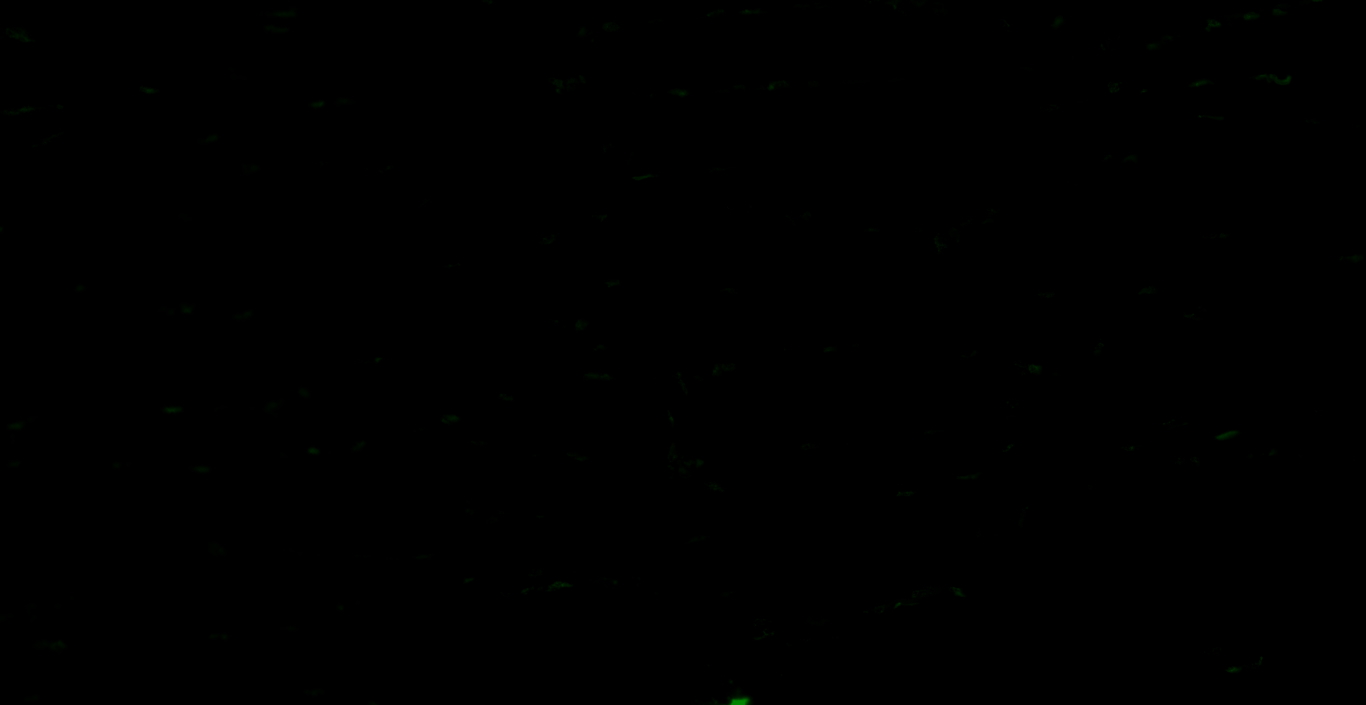

Supplement: Supplementary file 2 [file DataSheet4.ZIP › Supplemental materials 1/TUNEL/IR/5-2 TUNEL.jpg]

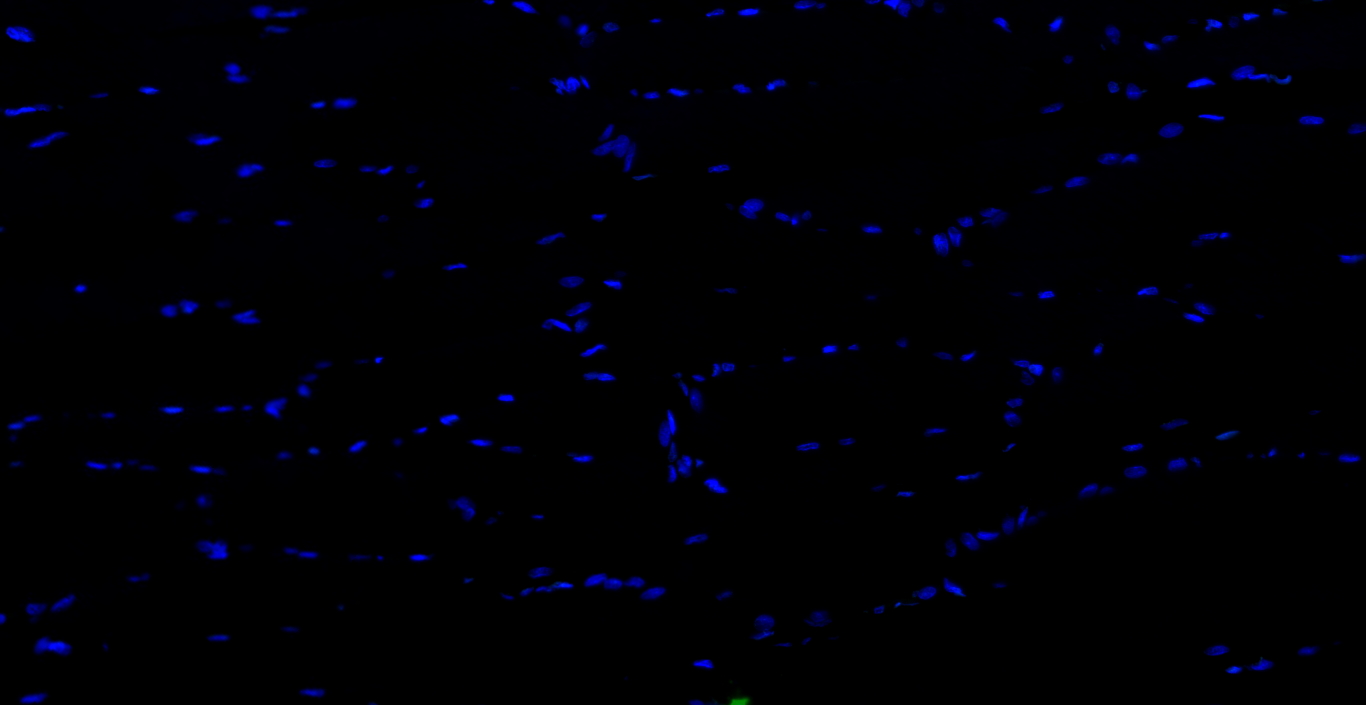

Supplement: Supplementary file 2 [file DataSheet4.ZIP › Supplemental materials 1/TUNEL/IR/5-2 merge.jpg]

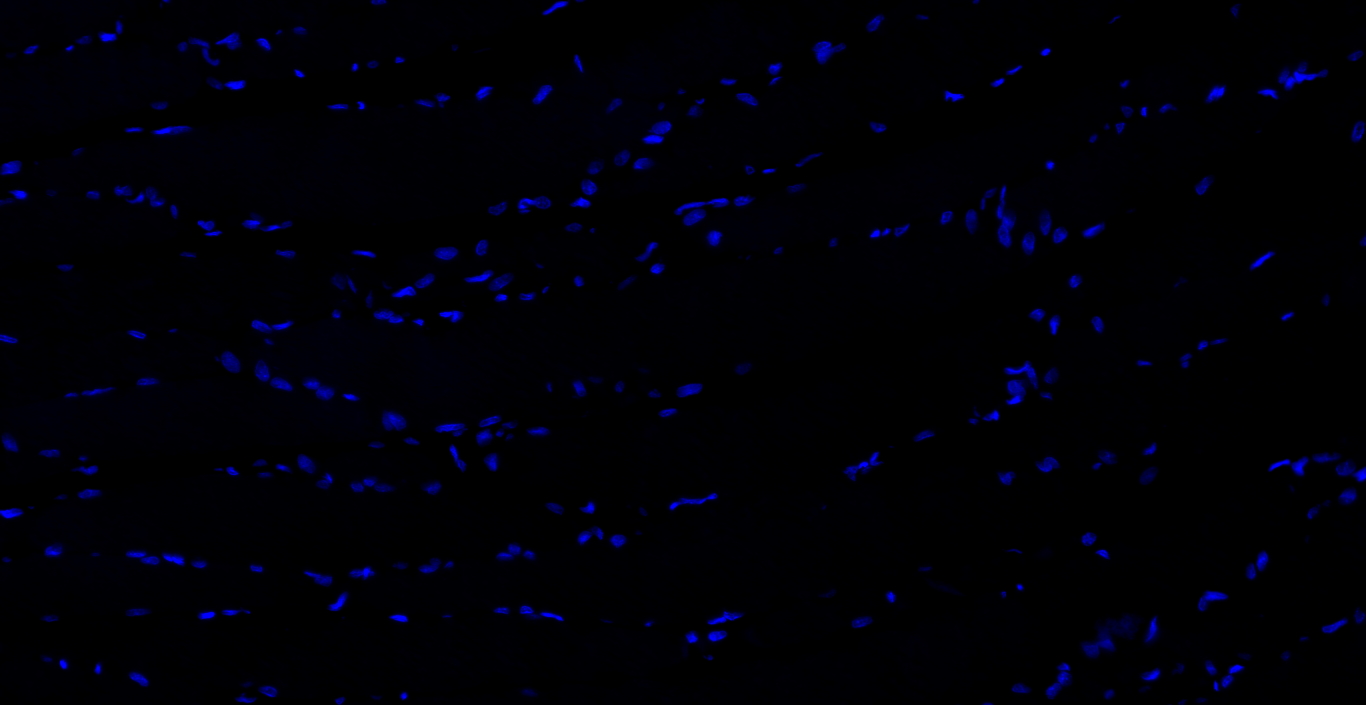

Supplement: Supplementary file 2 [file DataSheet4.ZIP › Supplemental materials 1/TUNEL/IR/5-3 DAPI.jpg]

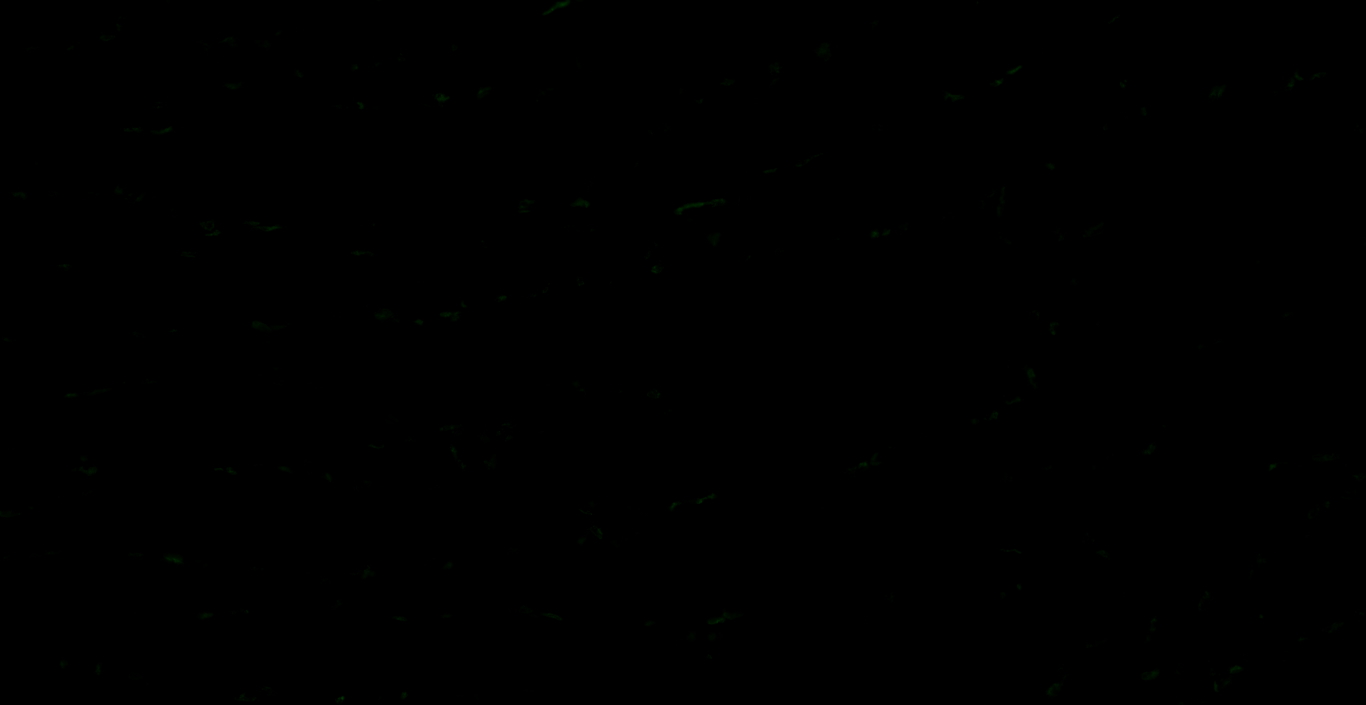

Supplement: Supplementary file 2 [file DataSheet4.ZIP › Supplemental materials 1/TUNEL/IR/5-3 TUNEL.jpg]

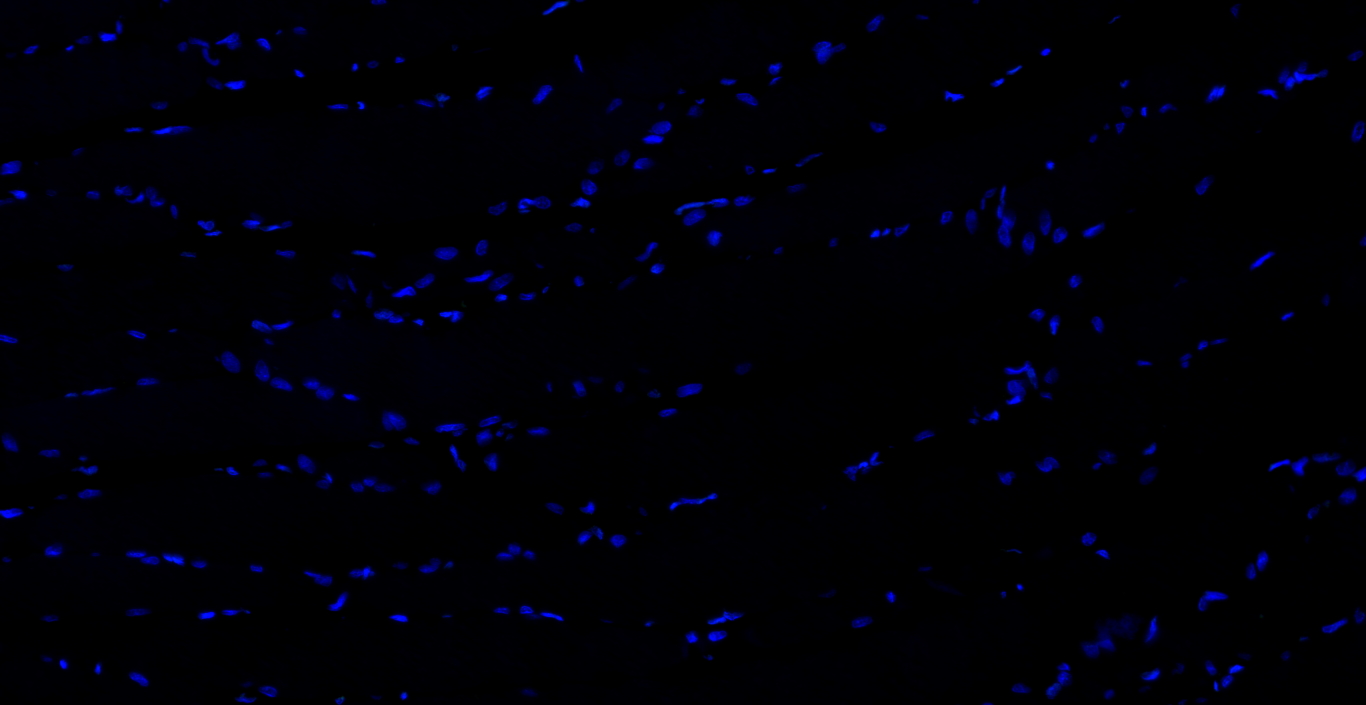

Supplement: Supplementary file 2 [file DataSheet4.ZIP › Supplemental materials 1/TUNEL/IR/5-3merge.jpg]

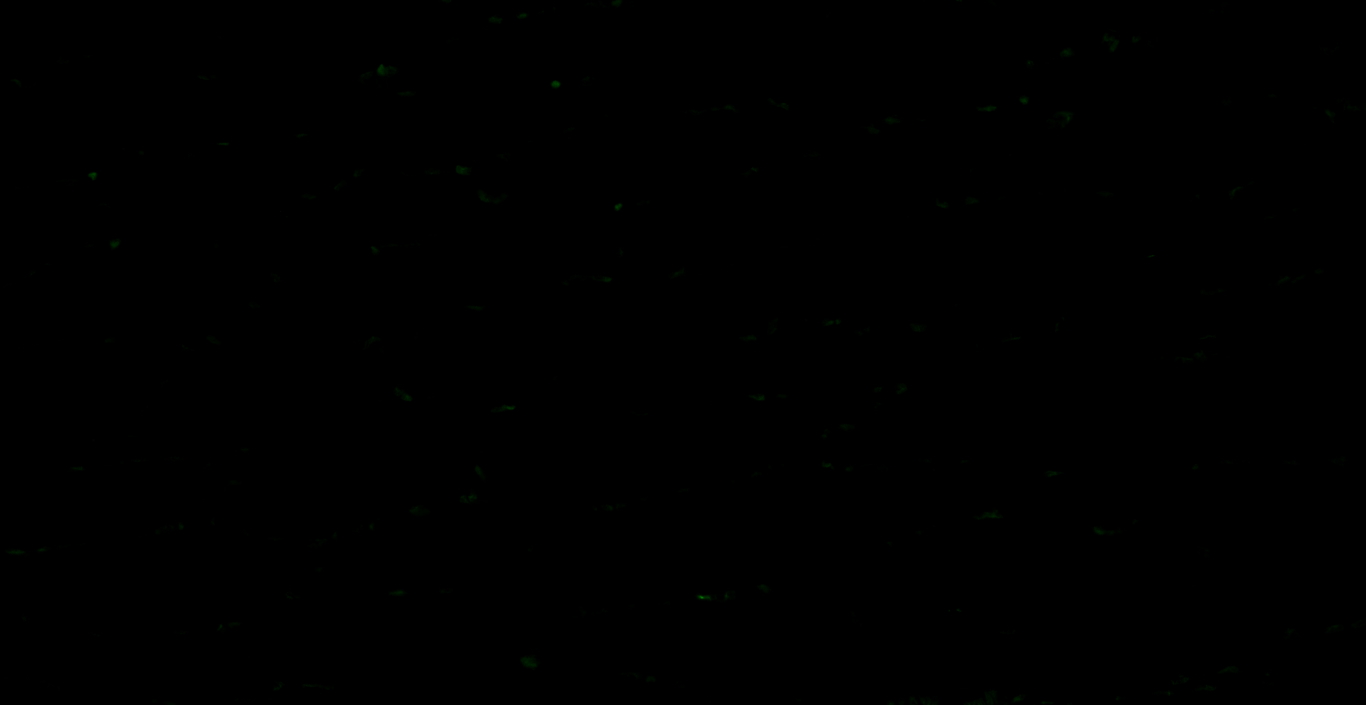

Supplement: Supplementary file 2 [file DataSheet4.ZIP › Supplemental materials 1/TUNEL/IR/6-1 DAPI.jpg]

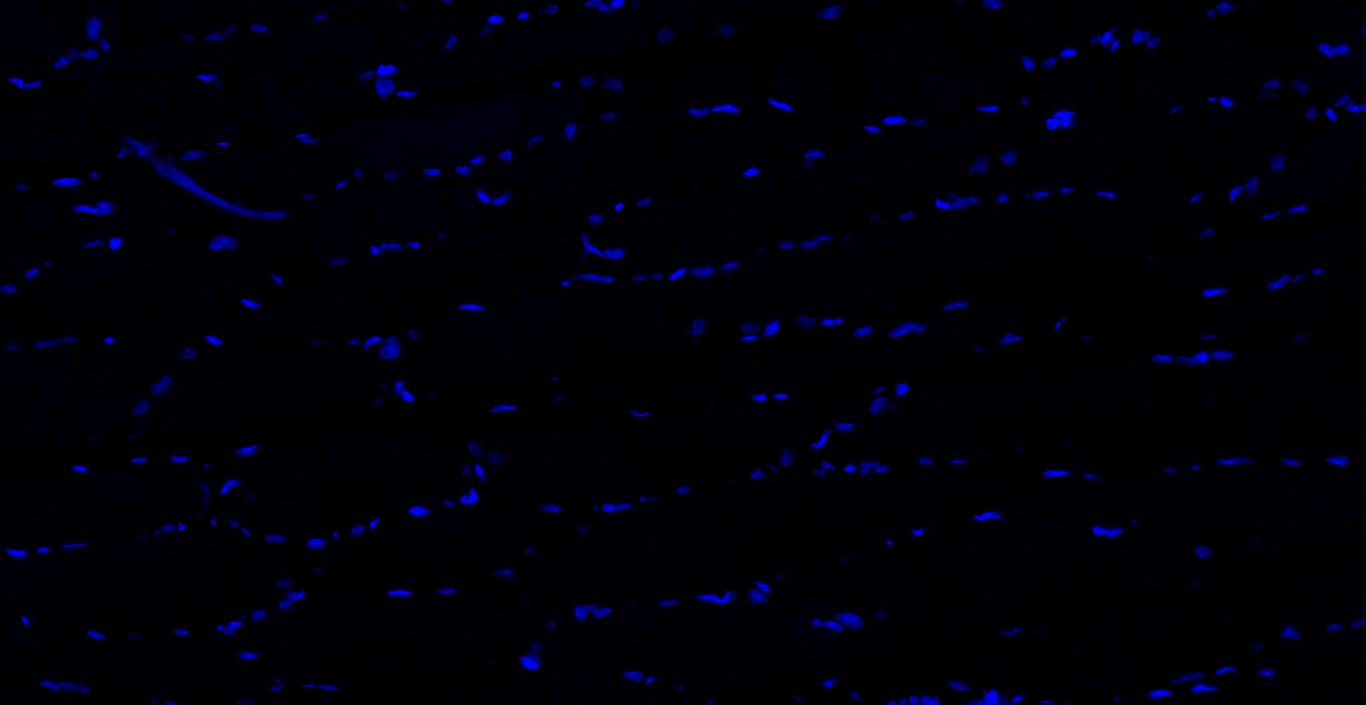

Supplement: Supplementary file 2 [file DataSheet4.ZIP › Supplemental materials 1/TUNEL/IR/6-1 TUNEL.jpg]

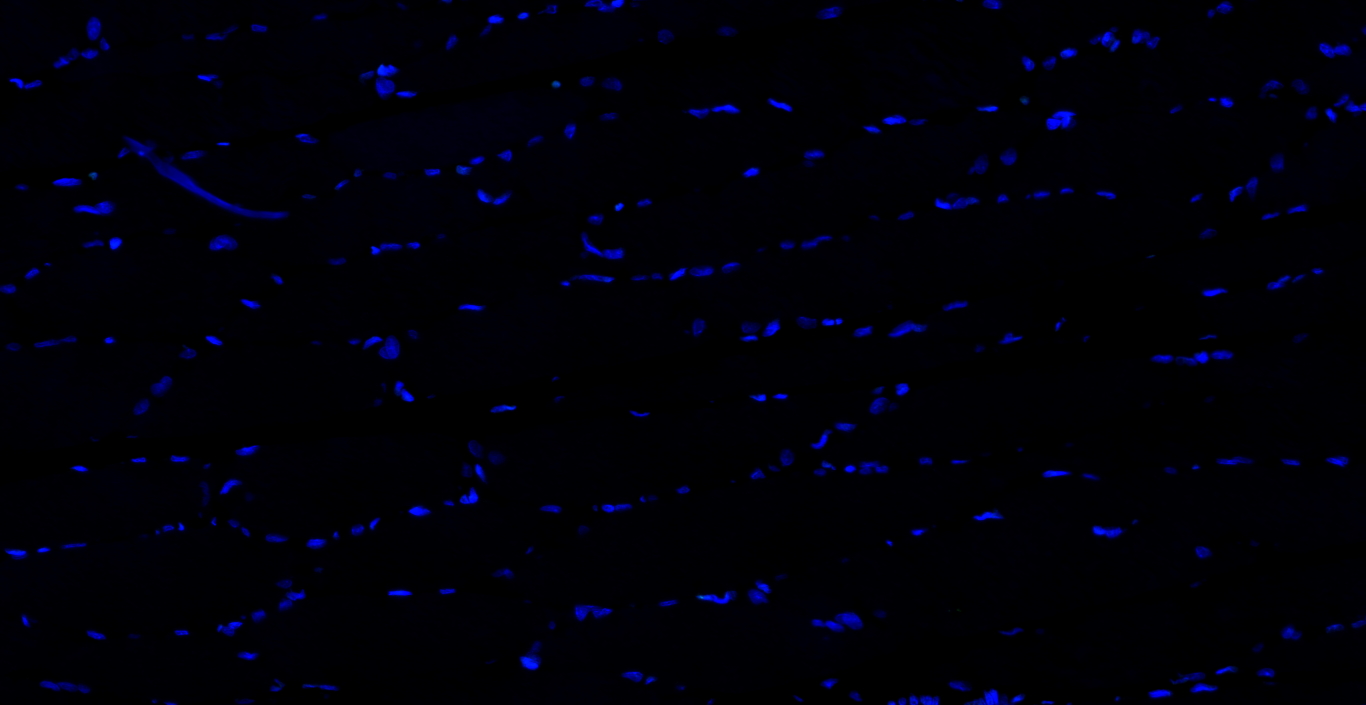

Supplement: Supplementary file 2 [file DataSheet4.ZIP › Supplemental materials 1/TUNEL/IR/6-1 merge.jpg]

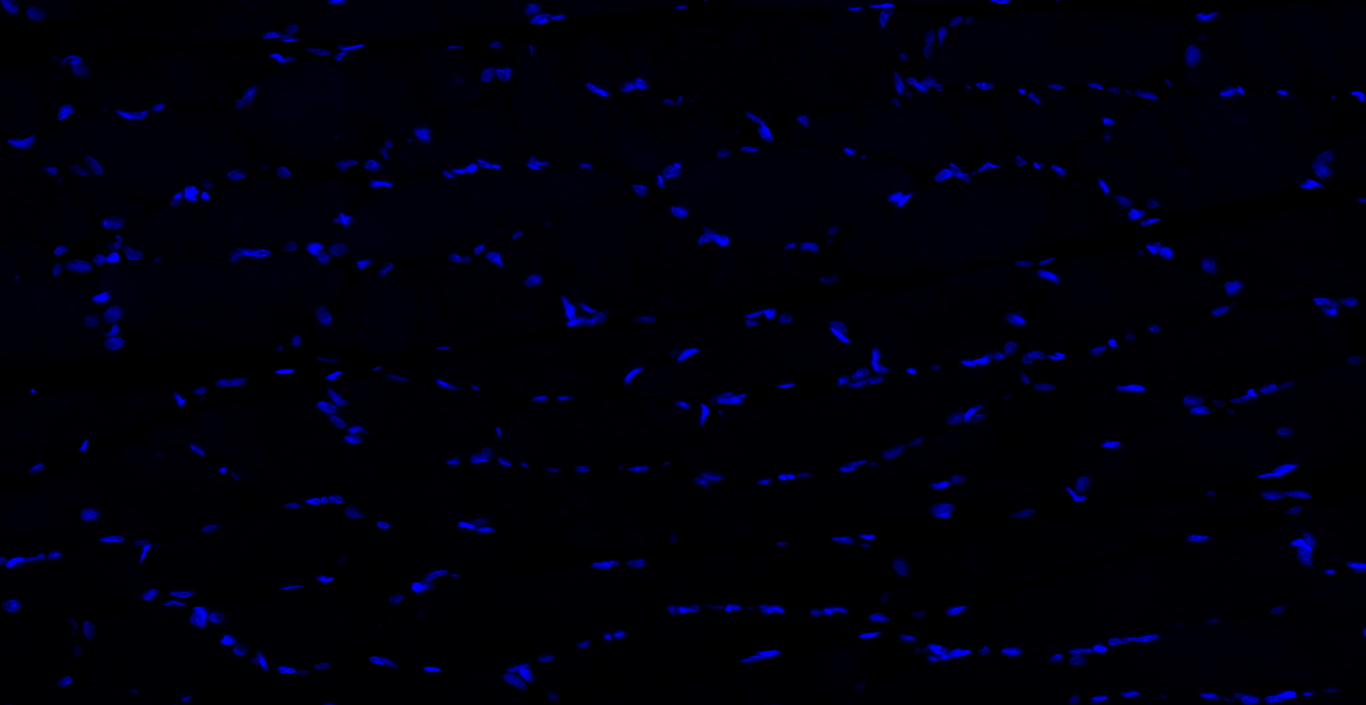

Supplement: Supplementary file 2 [file DataSheet4.ZIP › Supplemental materials 1/TUNEL/IR/6-2 DAPI.jpg]

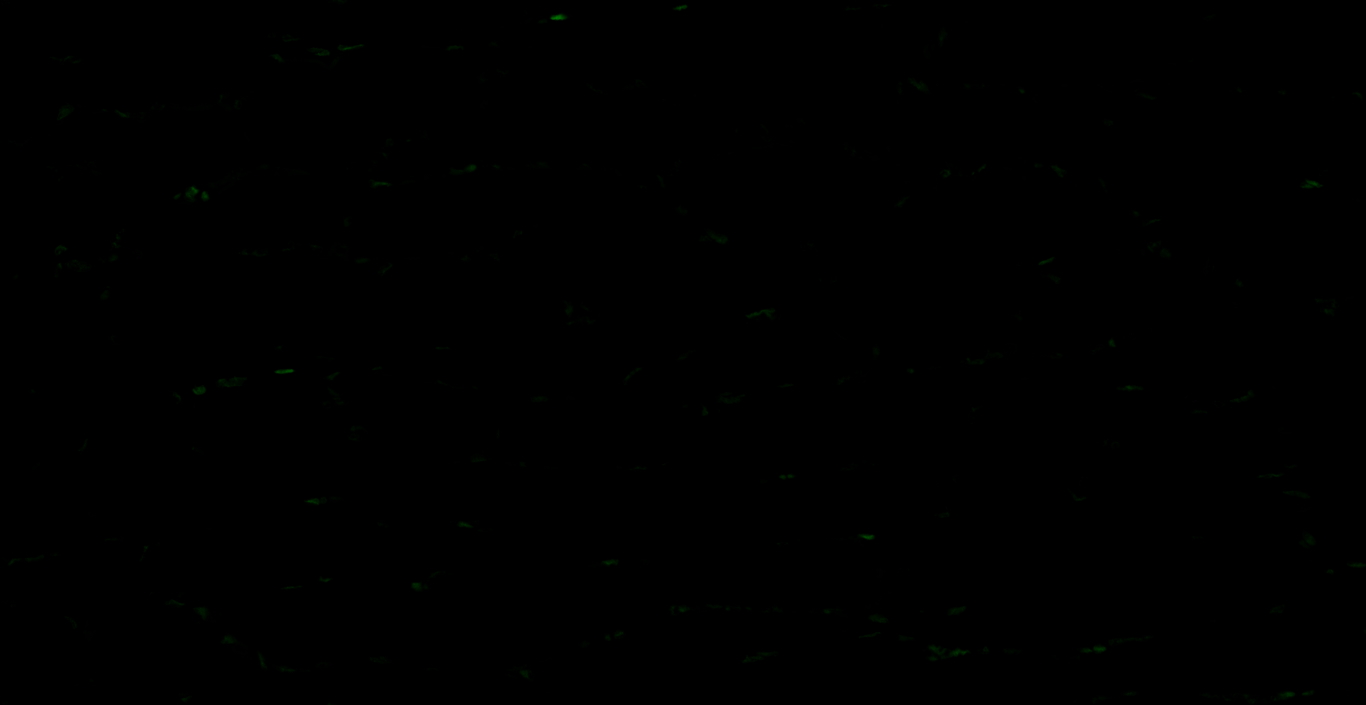

Supplement: Supplementary file 2 [file DataSheet4.ZIP › Supplemental materials 1/TUNEL/IR/6-2 TUNEL.jpg]

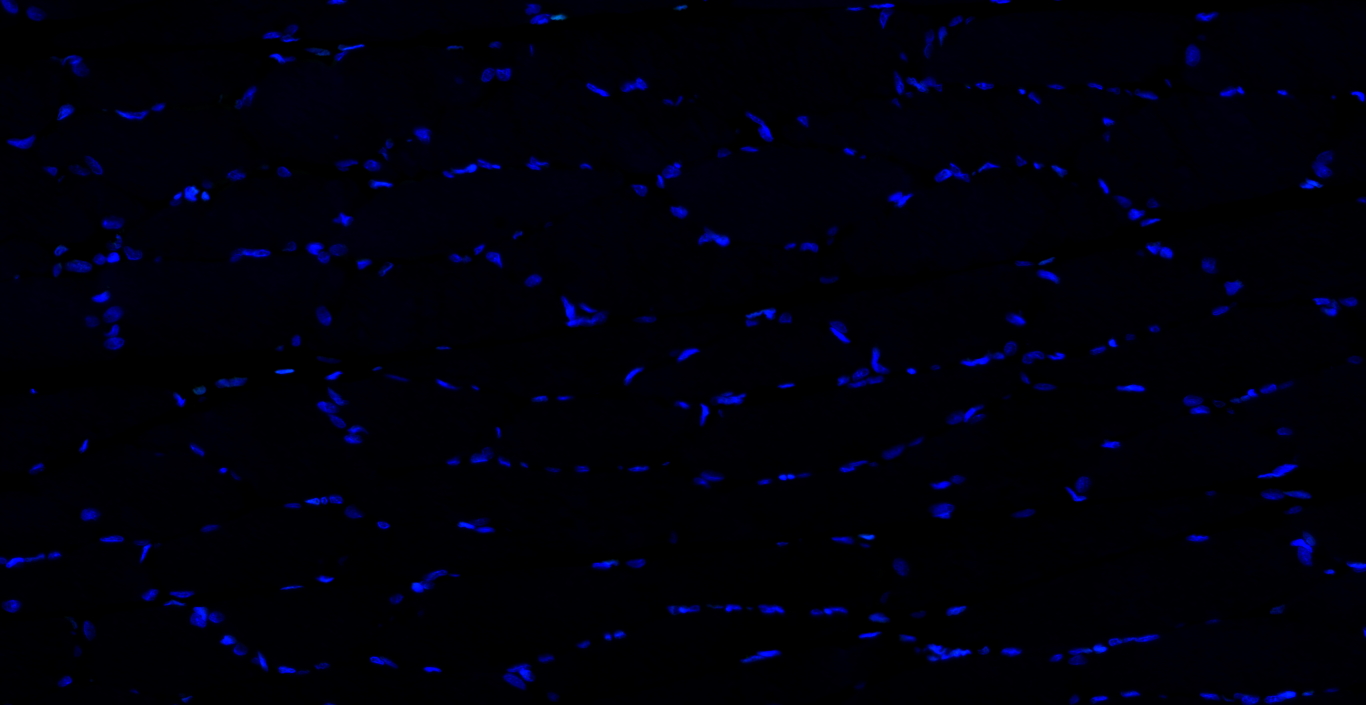

Supplement: Supplementary file 2 [file DataSheet4.ZIP › Supplemental materials 1/TUNEL/IR/6-2 merge.jpg]

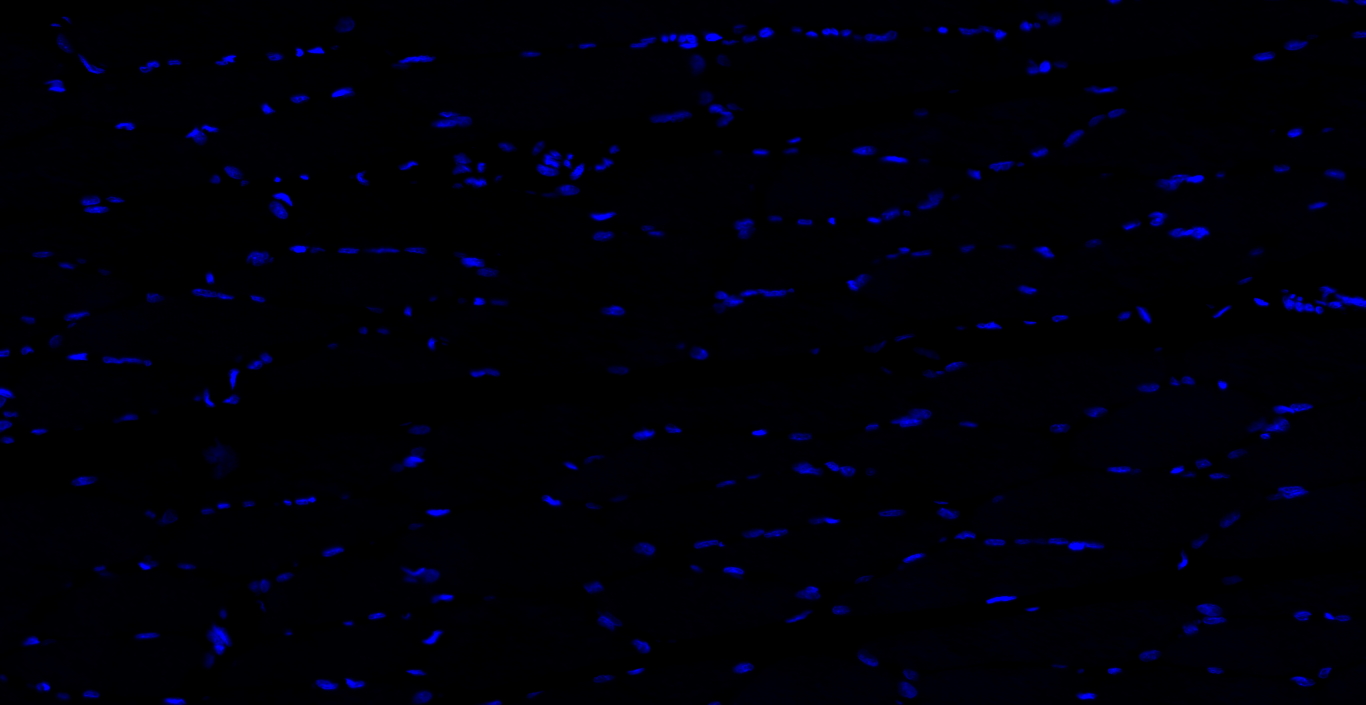

Supplement: Supplementary file 2 [file DataSheet4.ZIP › Supplemental materials 1/TUNEL/IR/6-3 DAPI.jpg]

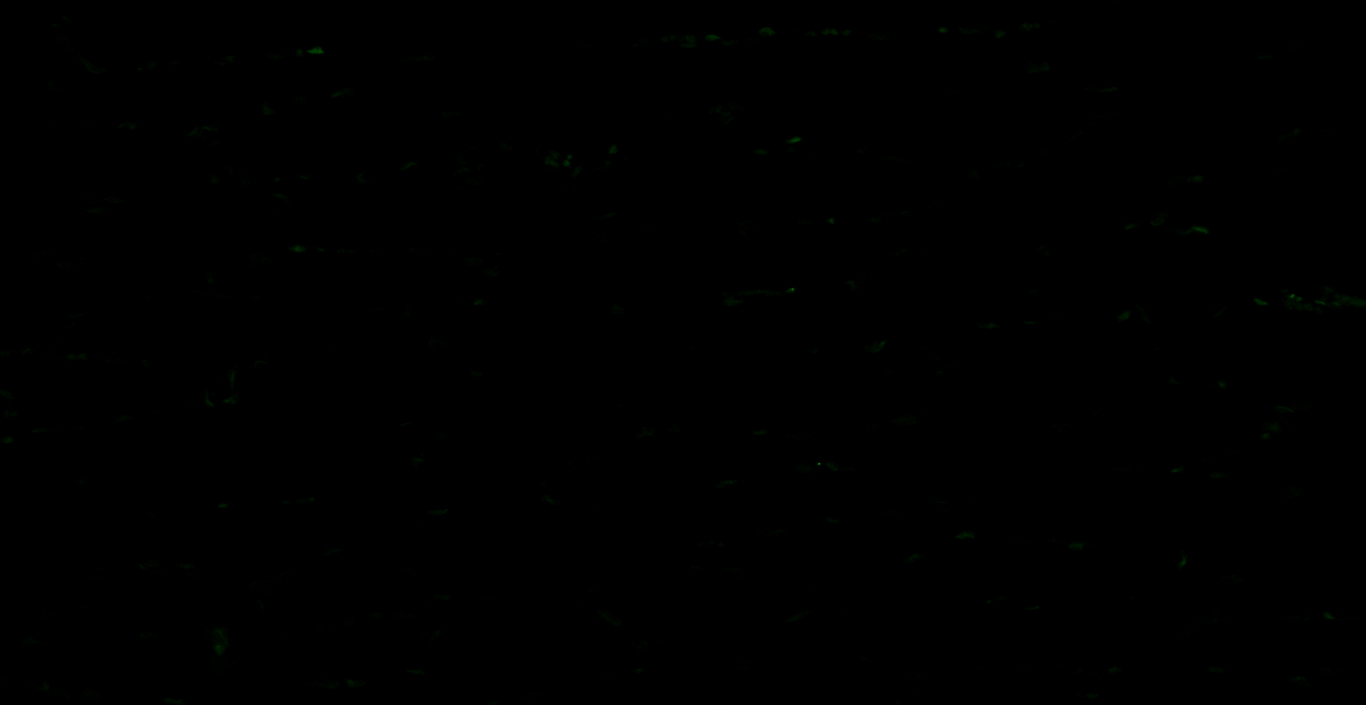

Supplement: Supplementary file 2 [file DataSheet4.ZIP › Supplemental materials 1/TUNEL/IR/6-3 TUNEL.jpg]

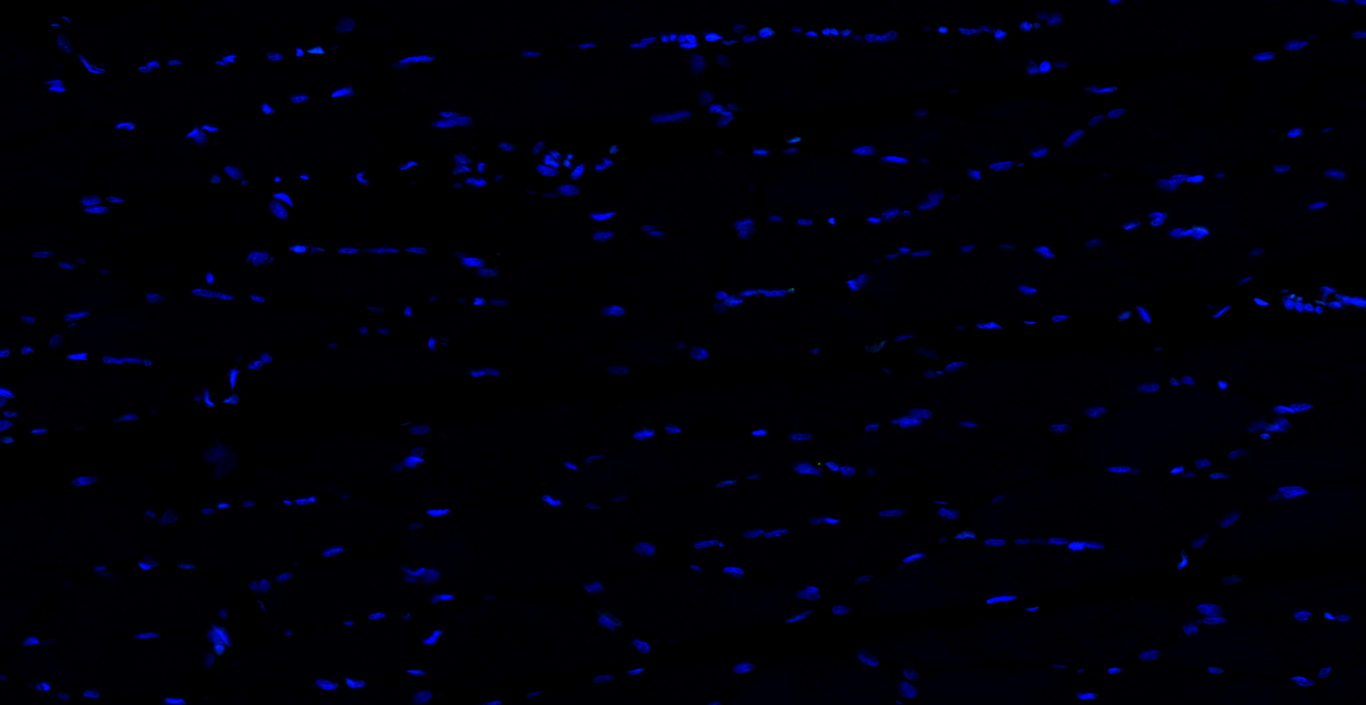

Supplement: Supplementary file 2 [file DataSheet4.ZIP › Supplemental materials 1/TUNEL/IR/6-3 merge.jpg]

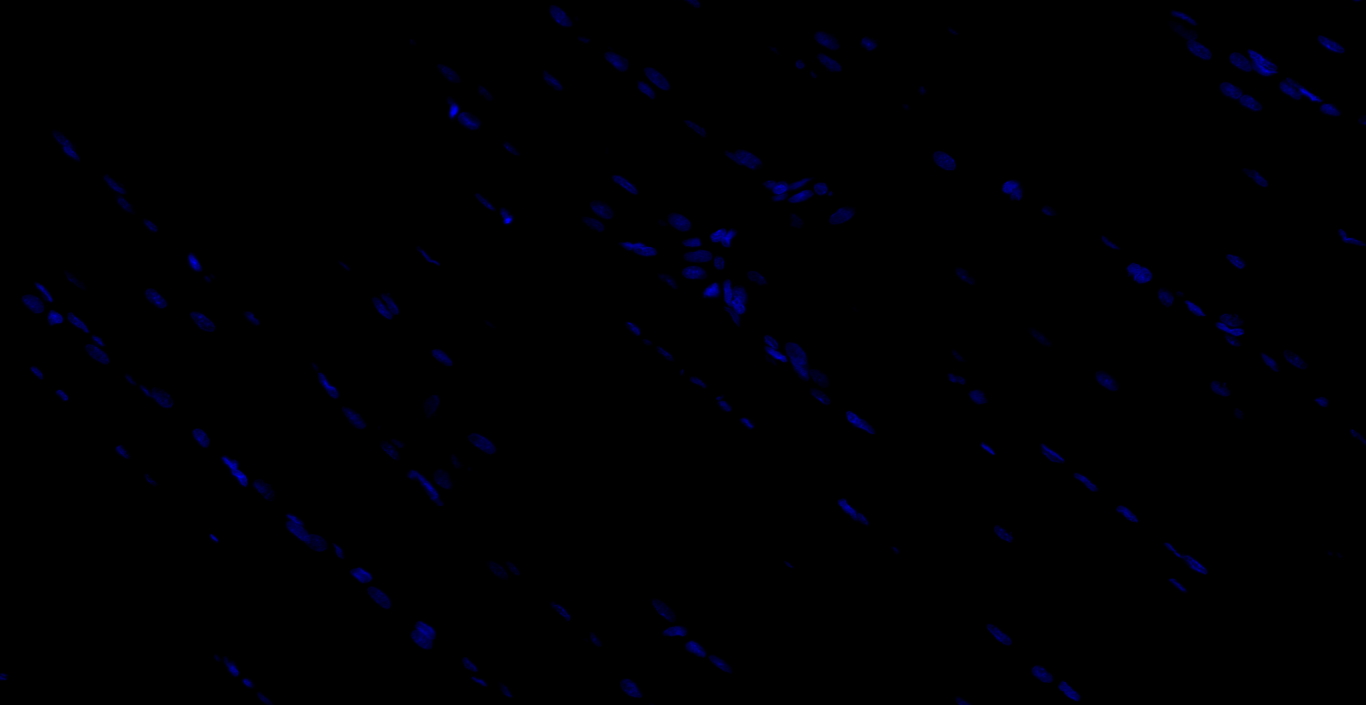

Supplement: Supplementary file 2 [file DataSheet4.ZIP › Supplemental materials 1/TUNEL/Sham/1-1 DAPI.jpg]

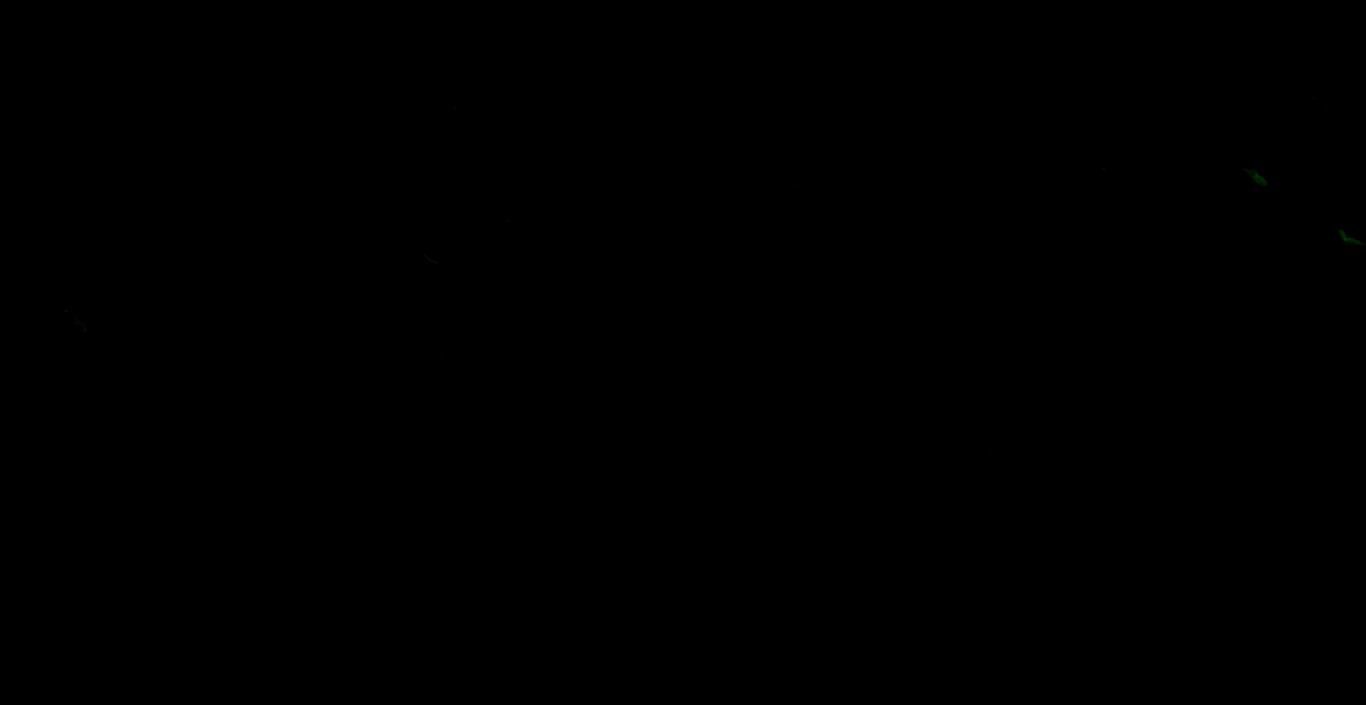

Supplement: Supplementary file 2 [file DataSheet4.ZIP › Supplemental materials 1/TUNEL/Sham/1-1 TUNEL.jpg]

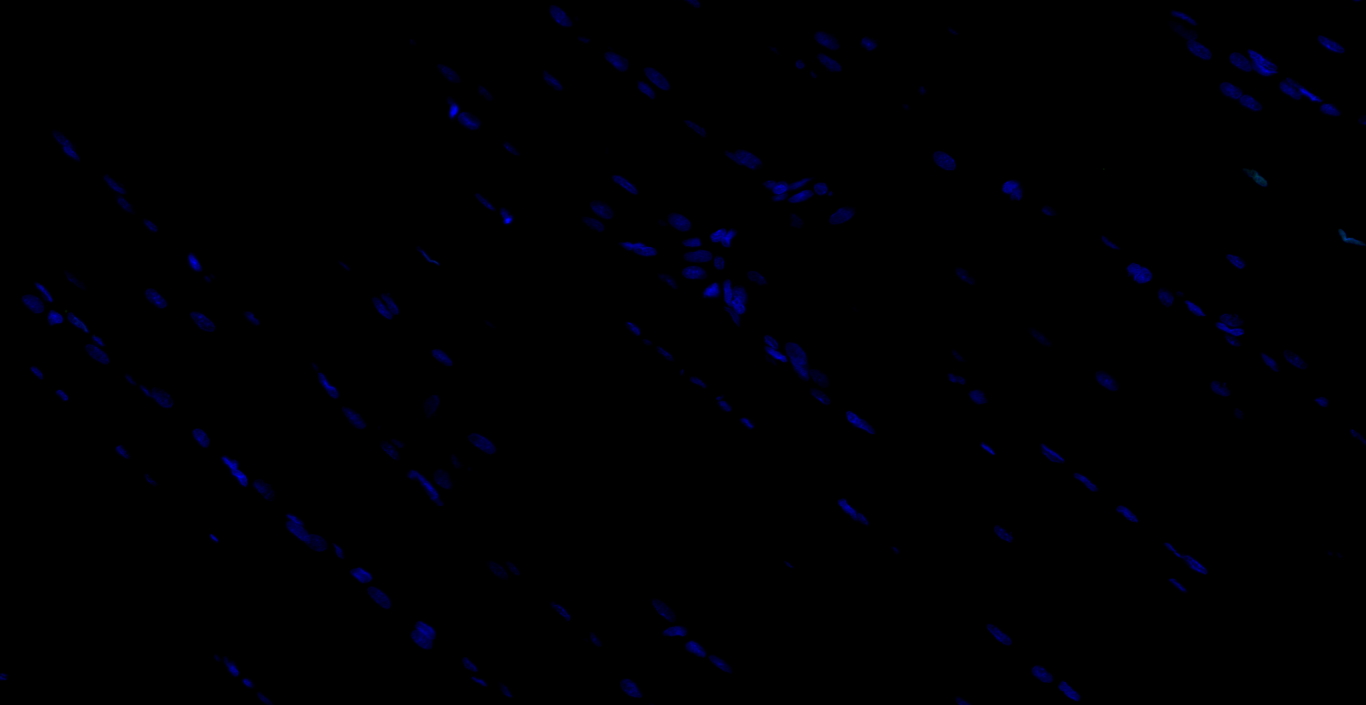

Supplement: Supplementary file 2 [file DataSheet4.ZIP › Supplemental materials 1/TUNEL/Sham/1-1 merge.jpg]

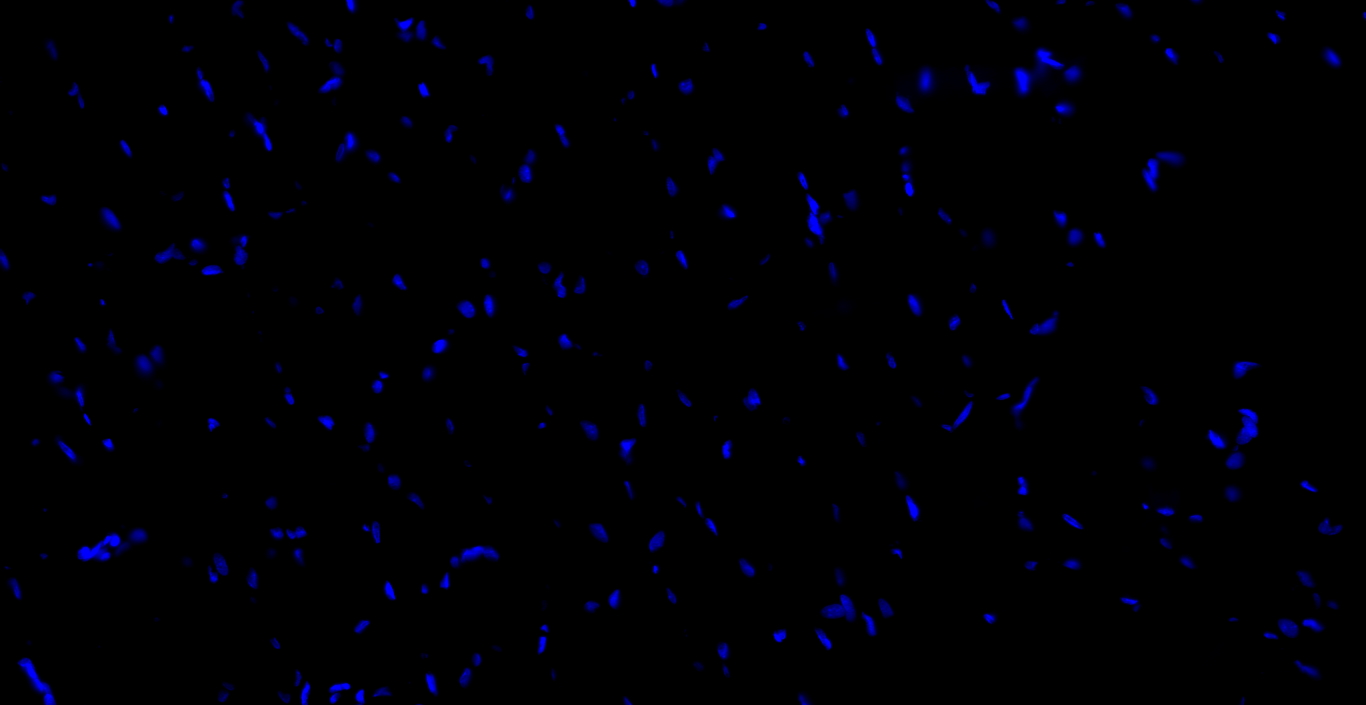

Supplement: Supplementary file 2 [file DataSheet4.ZIP › Supplemental materials 1/TUNEL/Sham/1-2 DAPI.jpg]

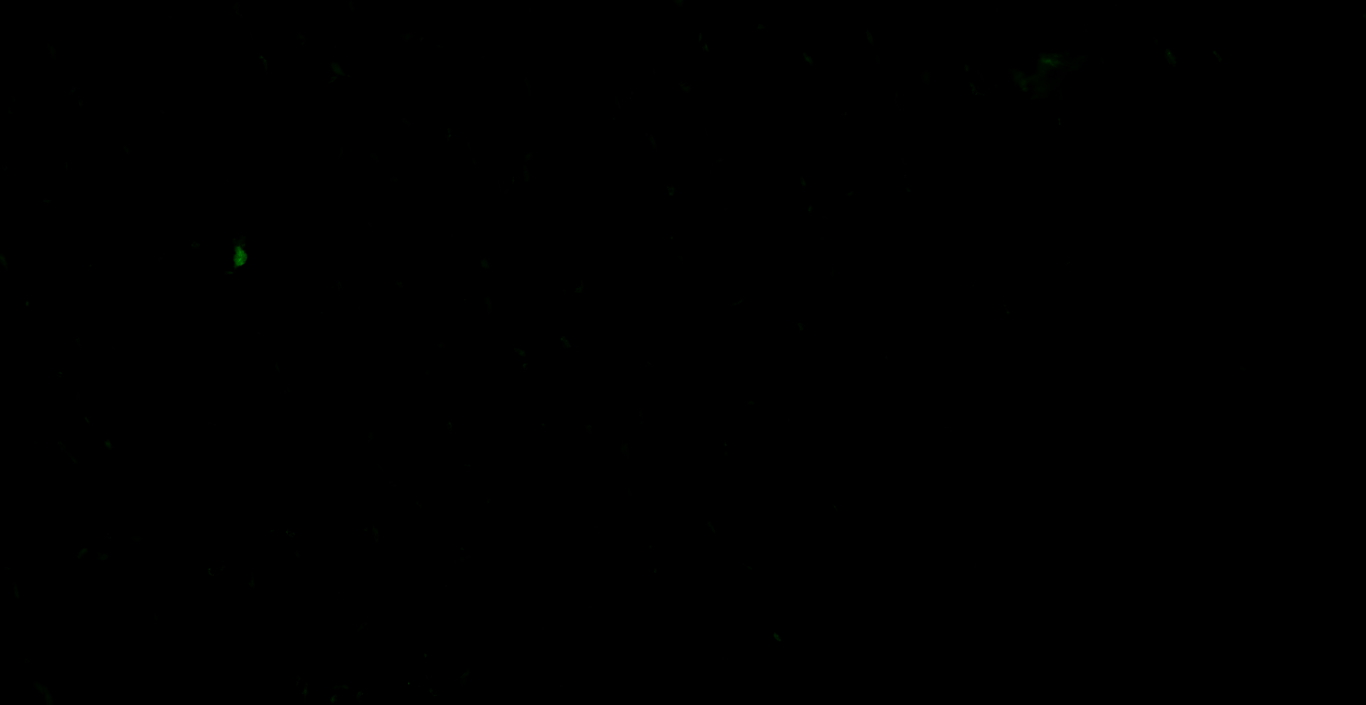

Supplement: Supplementary file 2 [file DataSheet4.ZIP › Supplemental materials 1/TUNEL/Sham/1-2 TUNEL.jpg]

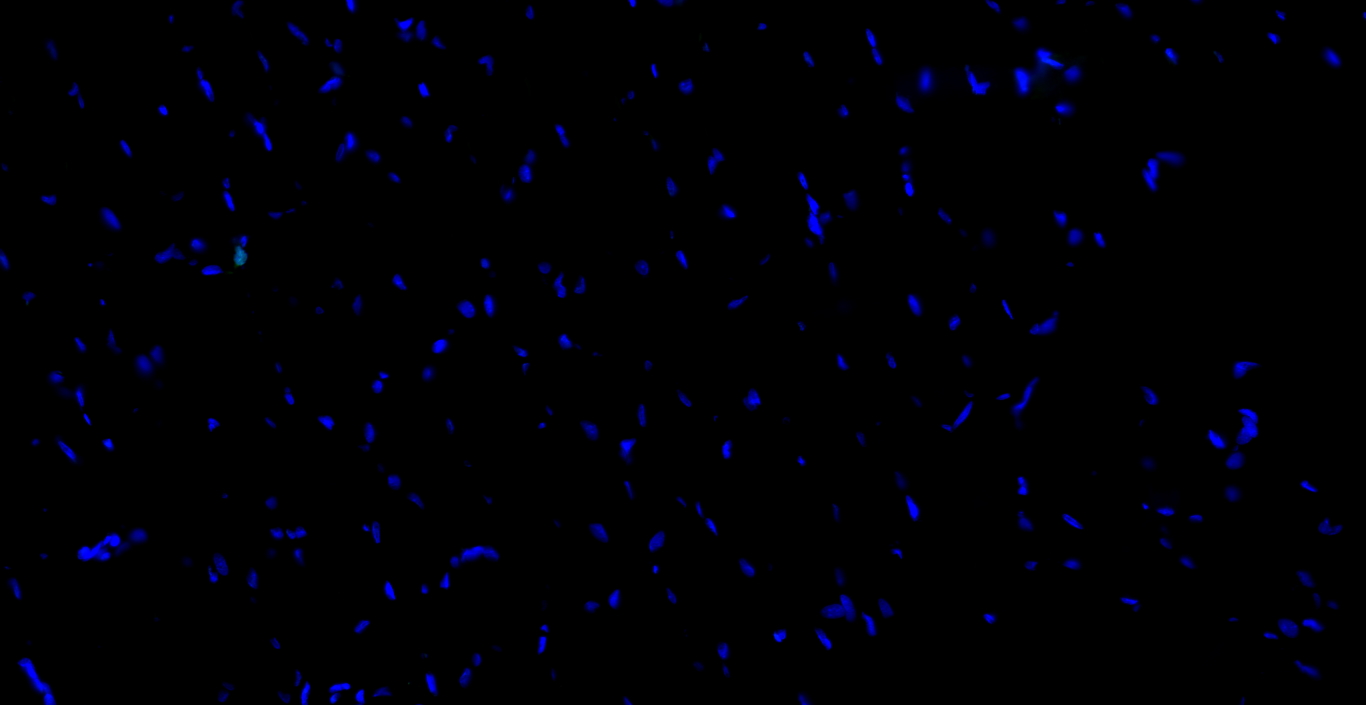

Supplement: Supplementary file 2 [file DataSheet4.ZIP › Supplemental materials 1/TUNEL/Sham/1-2 merge.jpg]

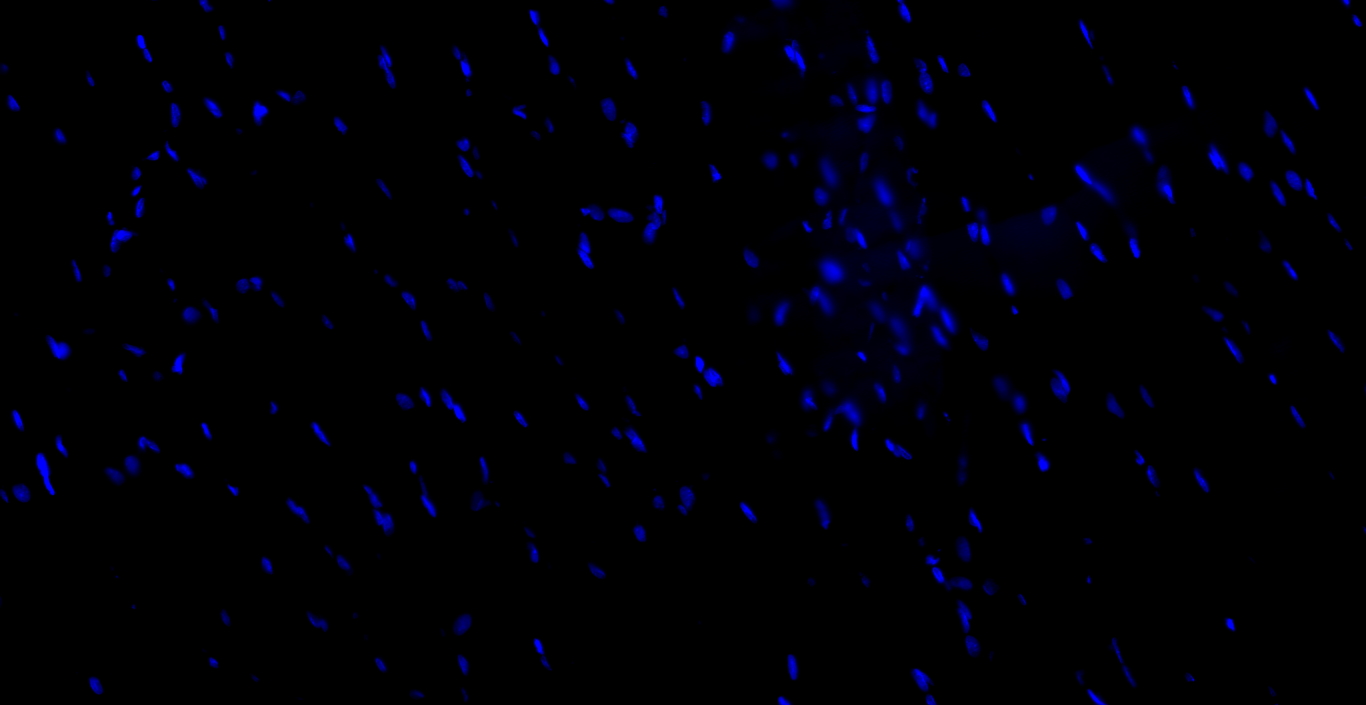

Supplement: Supplementary file 2 [file DataSheet4.ZIP › Supplemental materials 1/TUNEL/Sham/1-3 DAPI.jpg]

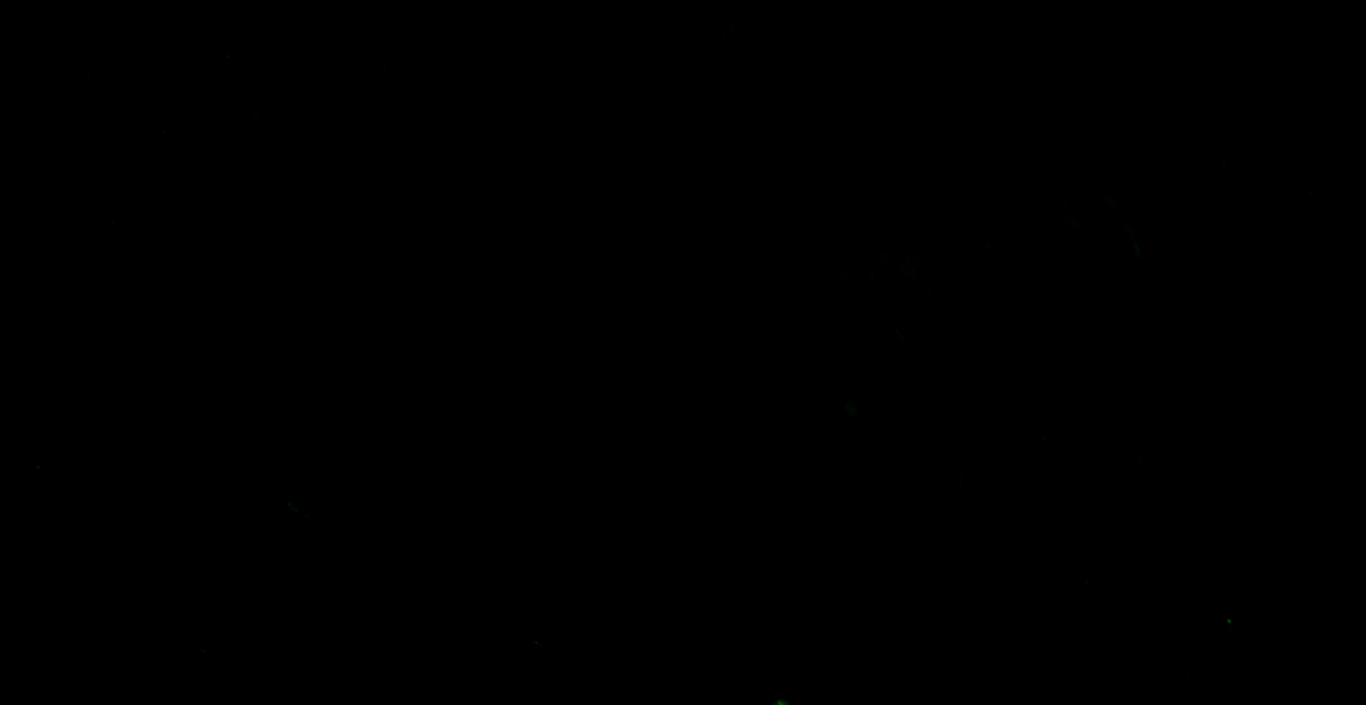

Supplement: Supplementary file 2 [file DataSheet4.ZIP › Supplemental materials 1/TUNEL/Sham/1-3 TUNEL.jpg]

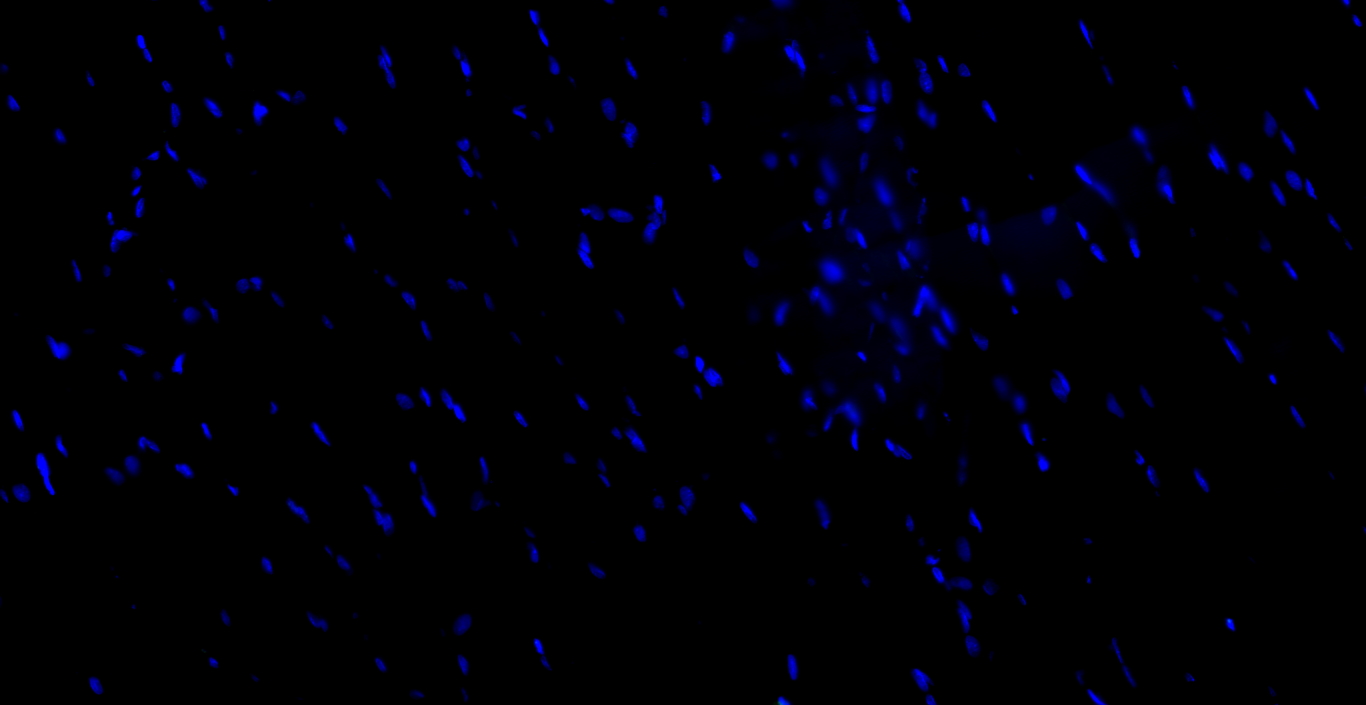

Supplement: Supplementary file 2 [file DataSheet4.ZIP › Supplemental materials 1/TUNEL/Sham/1-3 merge.jpg]

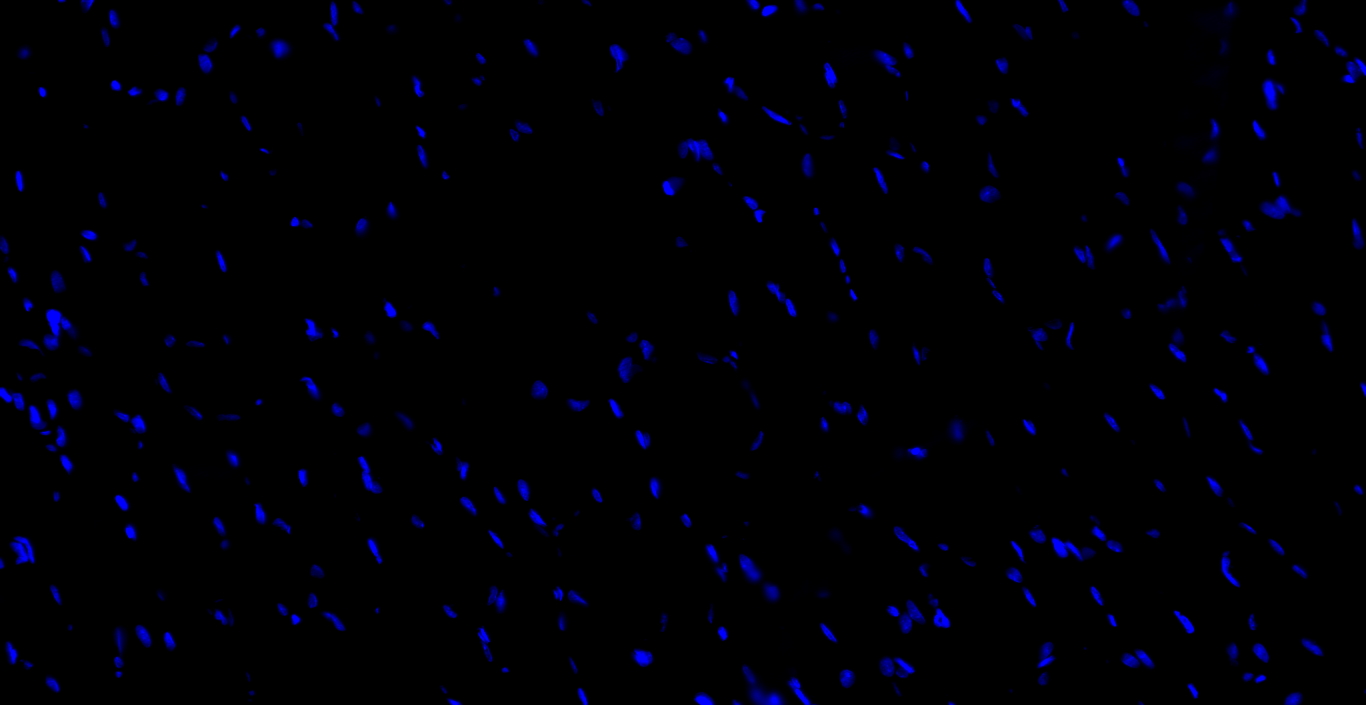

Supplement: Supplementary file 2 [file DataSheet4.ZIP › Supplemental materials 1/TUNEL/Sham/2-1 DAPI.jpg]

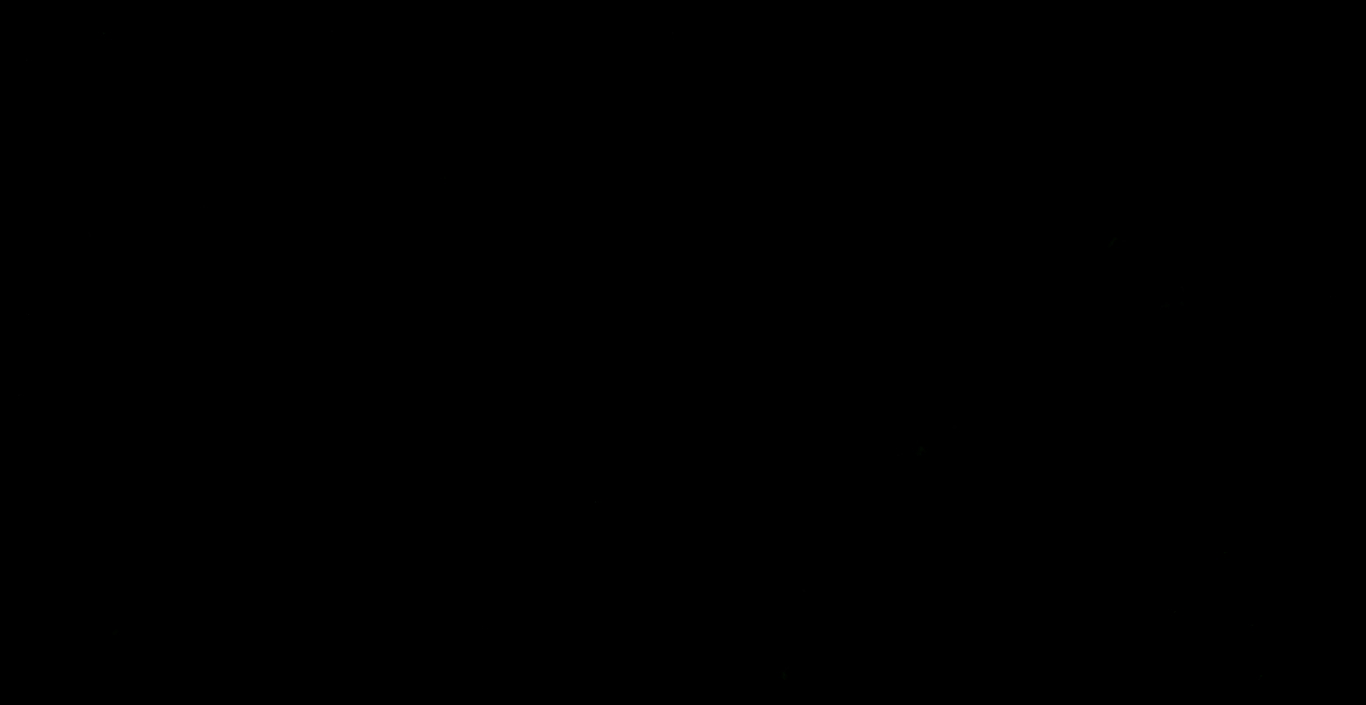

Supplement: Supplementary file 2 [file DataSheet4.ZIP › Supplemental materials 1/TUNEL/Sham/2-1 TUNEL.jpg]

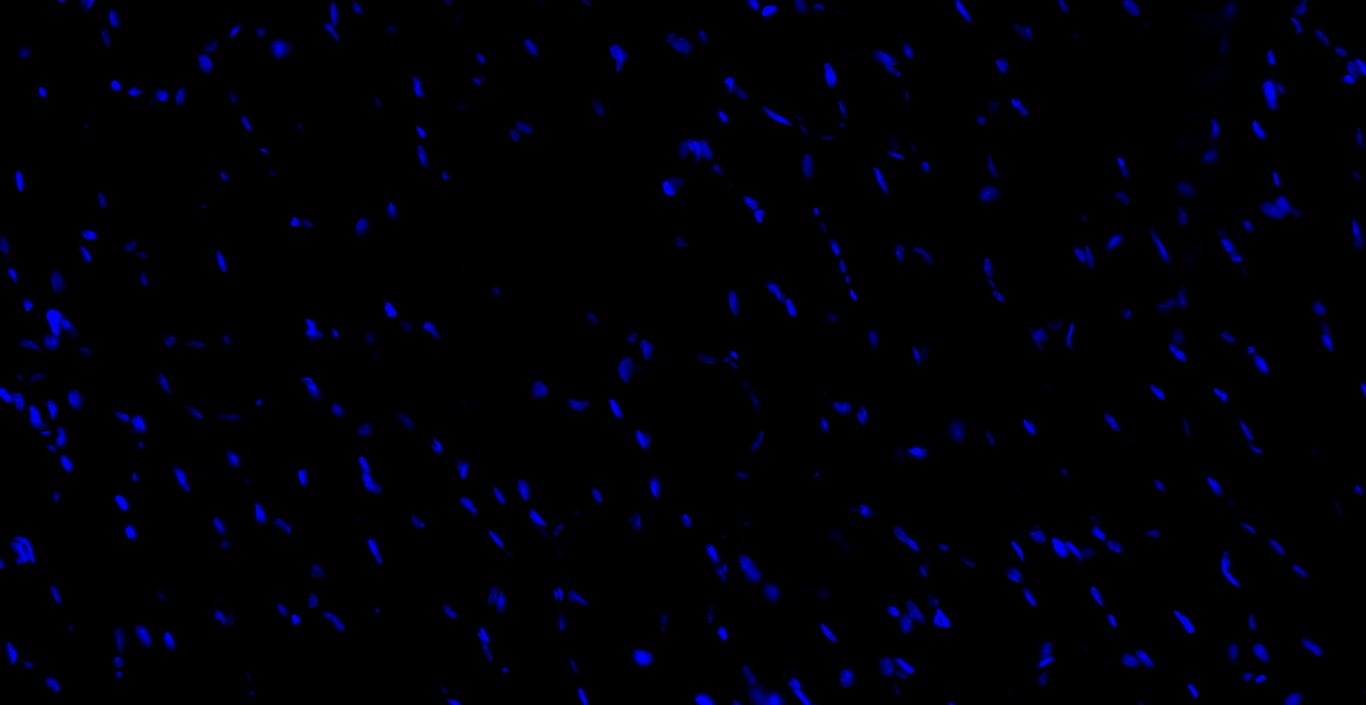

Supplement: Supplementary file 2 [file DataSheet4.ZIP › Supplemental materials 1/TUNEL/Sham/2-1 merge.jpg]

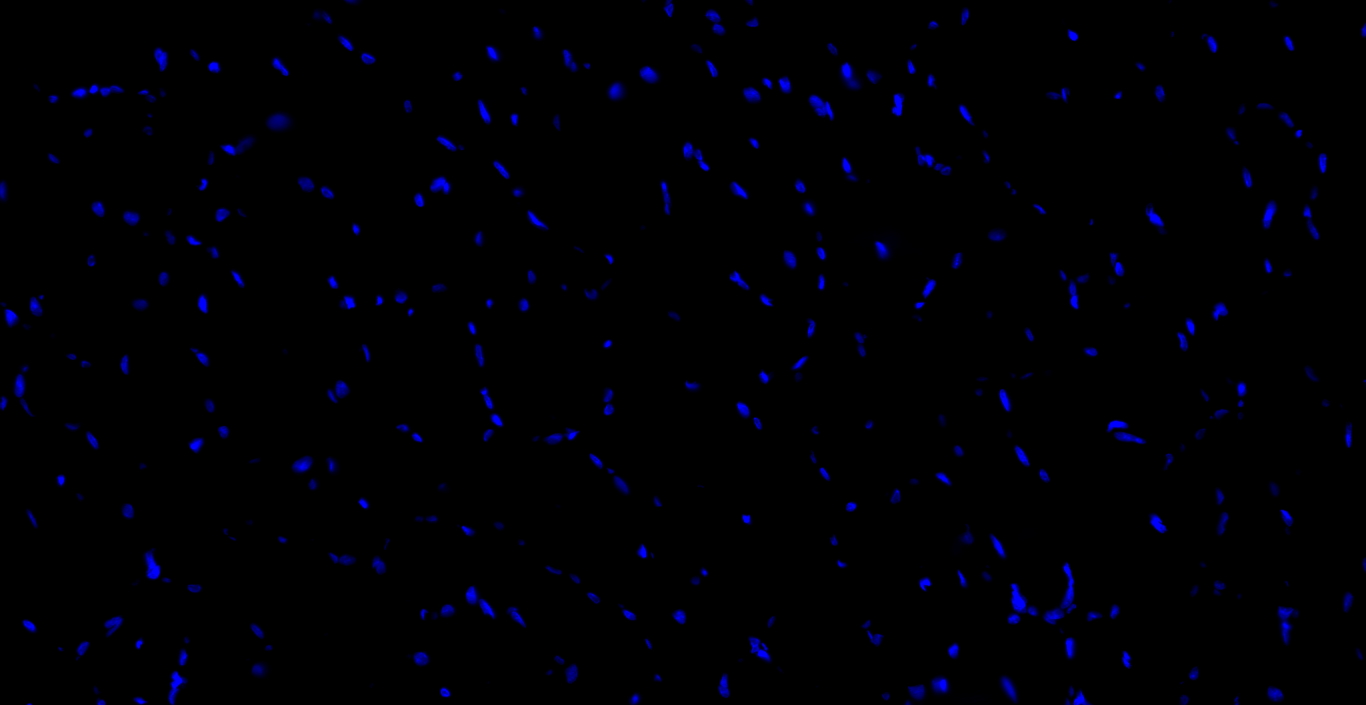

Supplement: Supplementary file 2 [file DataSheet4.ZIP › Supplemental materials 1/TUNEL/Sham/2-2 DAPI.jpg]

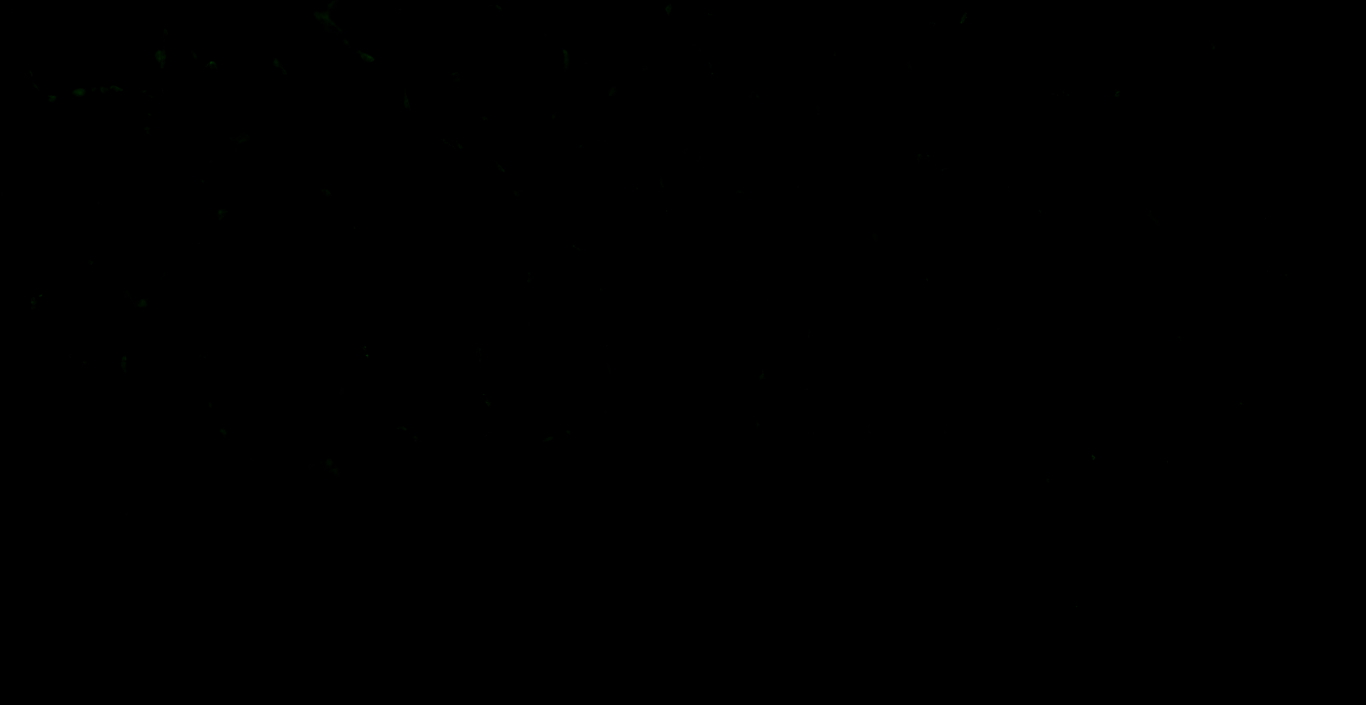

Supplement: Supplementary file 2 [file DataSheet4.ZIP › Supplemental materials 1/TUNEL/Sham/2-2 TUNEL.jpg]

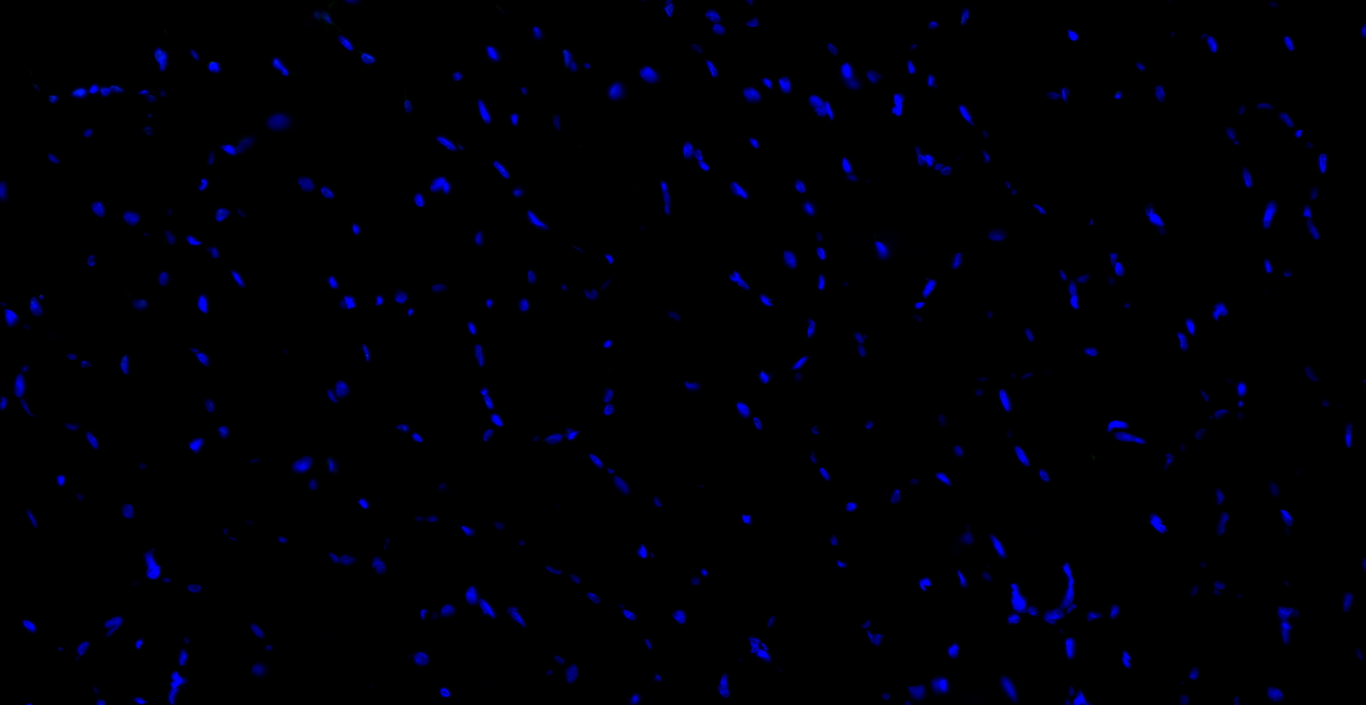

Supplement: Supplementary file 2 [file DataSheet4.ZIP › Supplemental materials 1/TUNEL/Sham/2-2 merge.jpg]

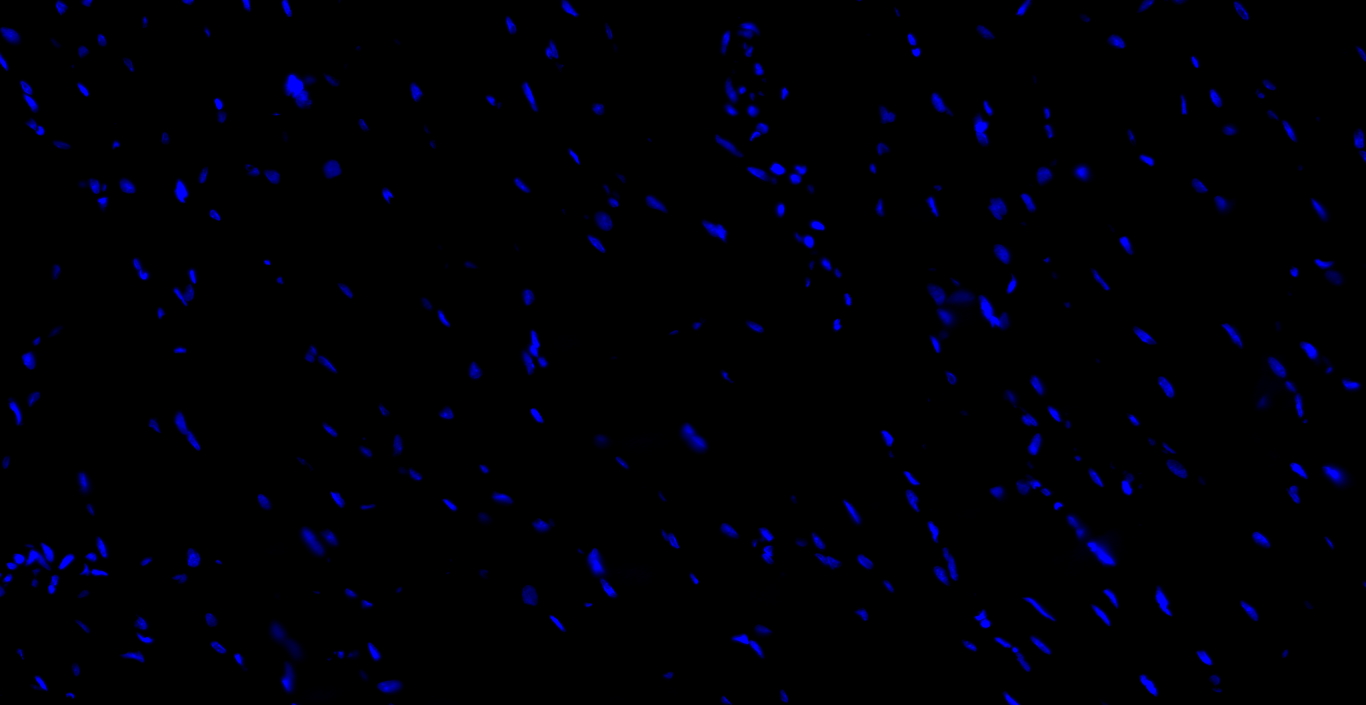

Supplement: Supplementary file 2 [file DataSheet4.ZIP › Supplemental materials 1/TUNEL/Sham/2-3 DAPI.jpg]

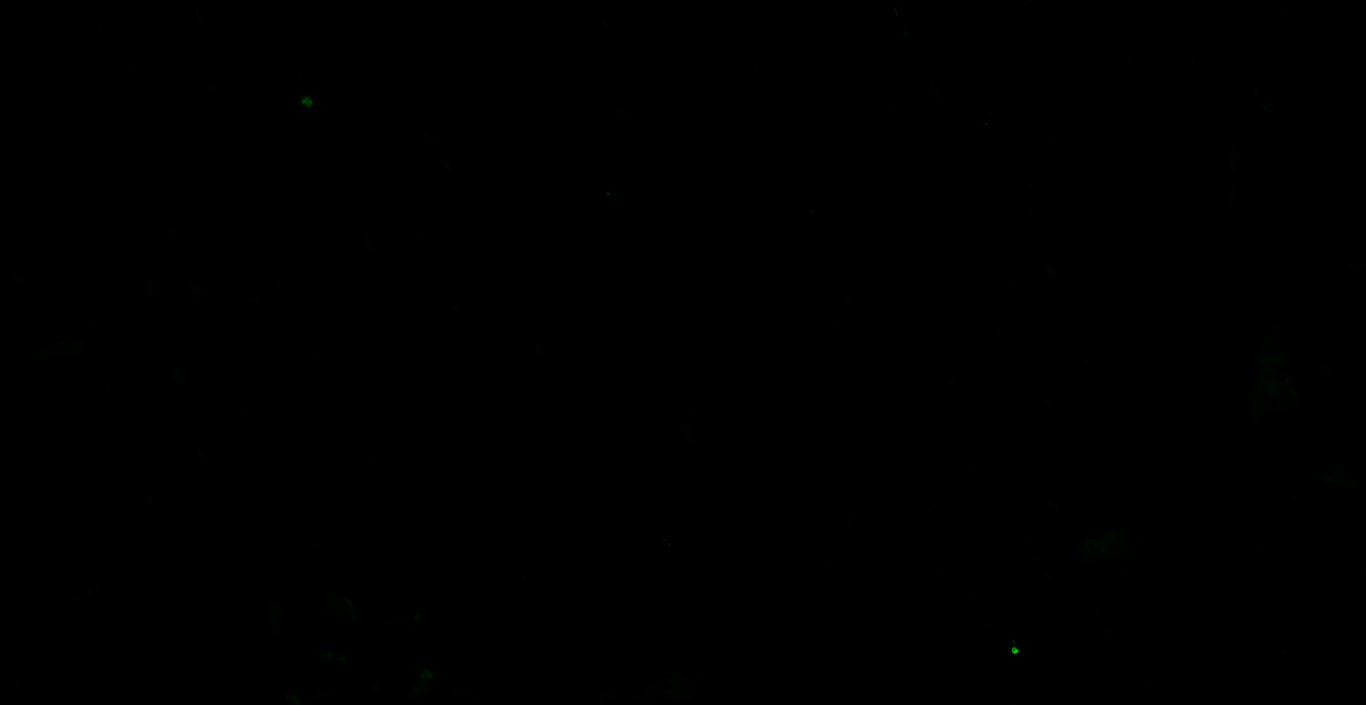

Supplement: Supplementary file 2 [file DataSheet4.ZIP › Supplemental materials 1/TUNEL/Sham/2-3 TUNEL.jpg]

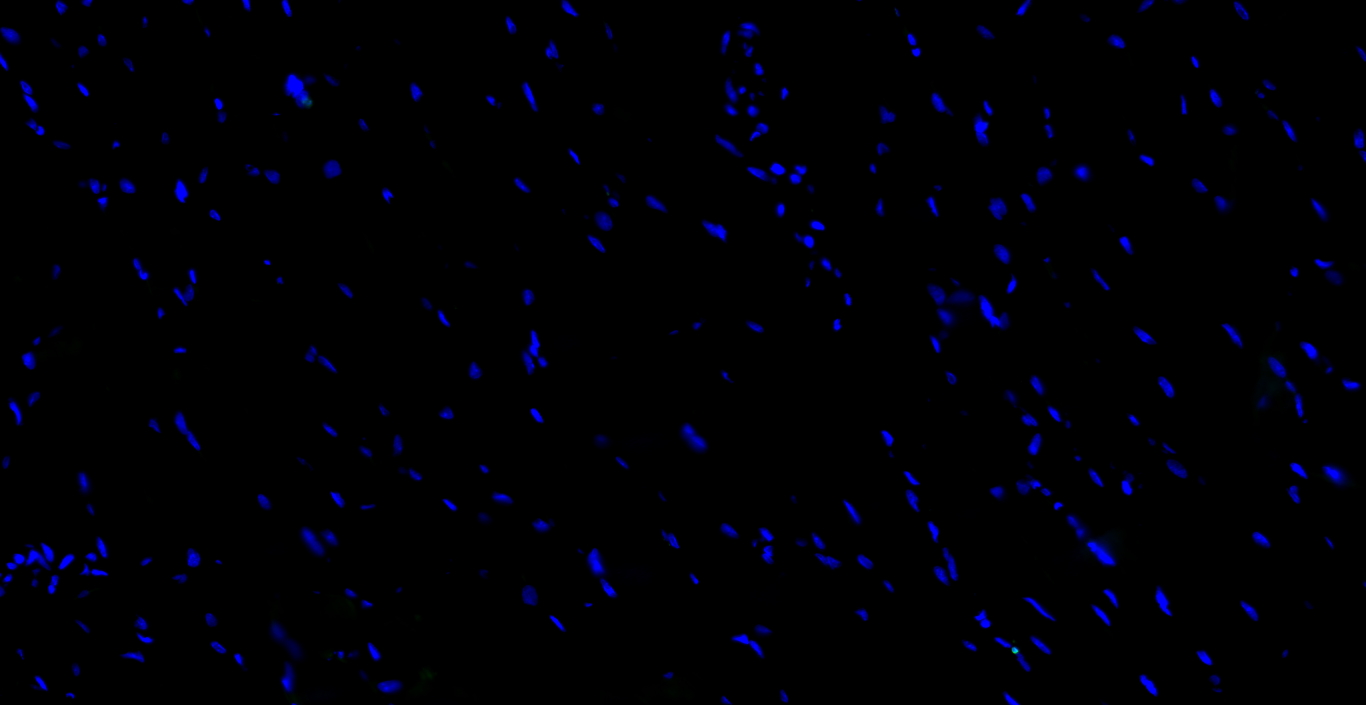

Supplement: Supplementary file 2 [file DataSheet4.ZIP › Supplemental materials 1/TUNEL/Sham/2-3 merge.jpg]

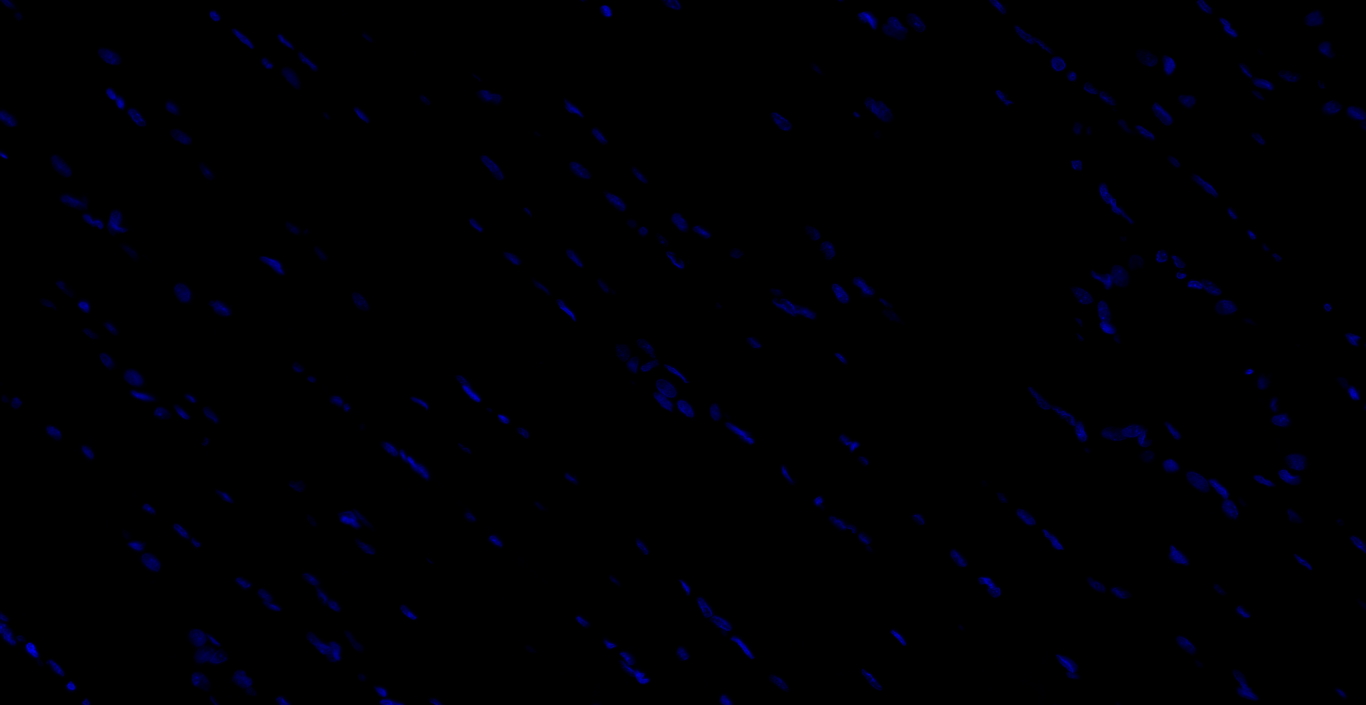

Supplement: Supplementary file 2 [file DataSheet4.ZIP › Supplemental materials 1/TUNEL/Sham/3-1 DAPI.jpg]

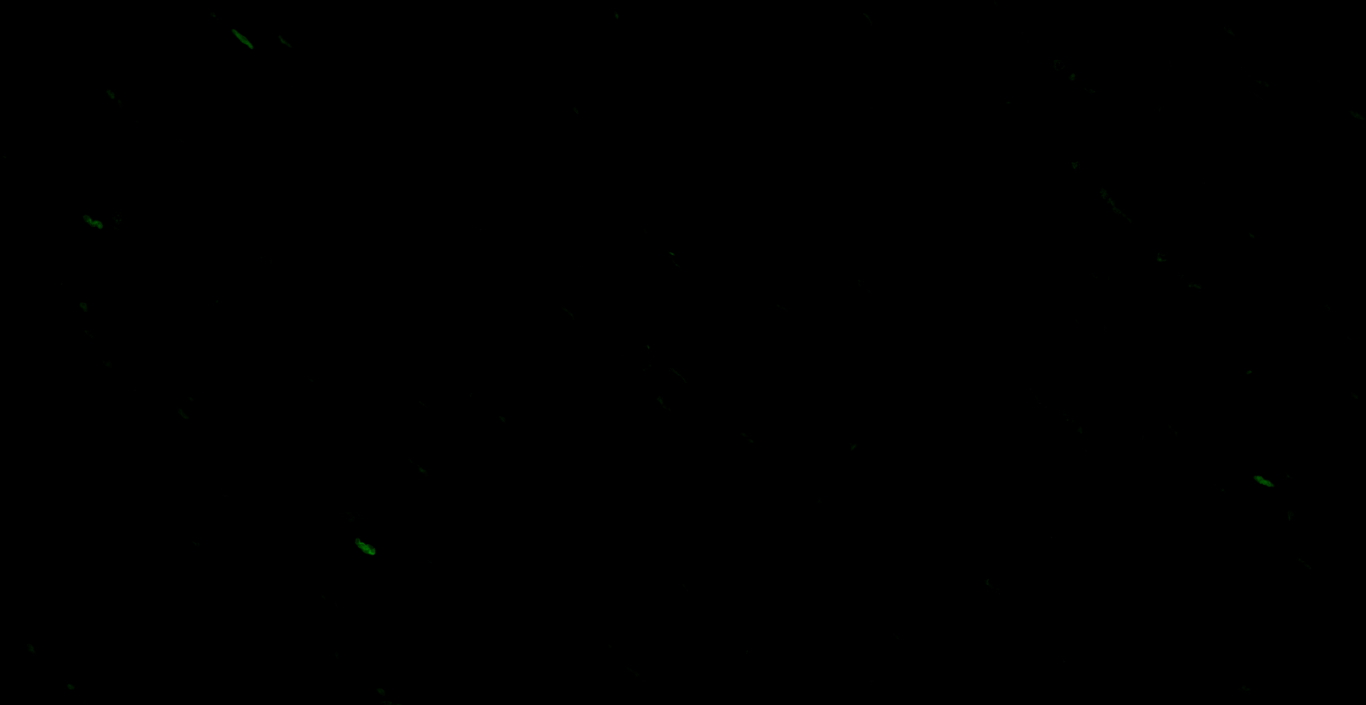

Supplement: Supplementary file 2 [file DataSheet4.ZIP › Supplemental materials 1/TUNEL/Sham/3-1 TUNEL.jpg]

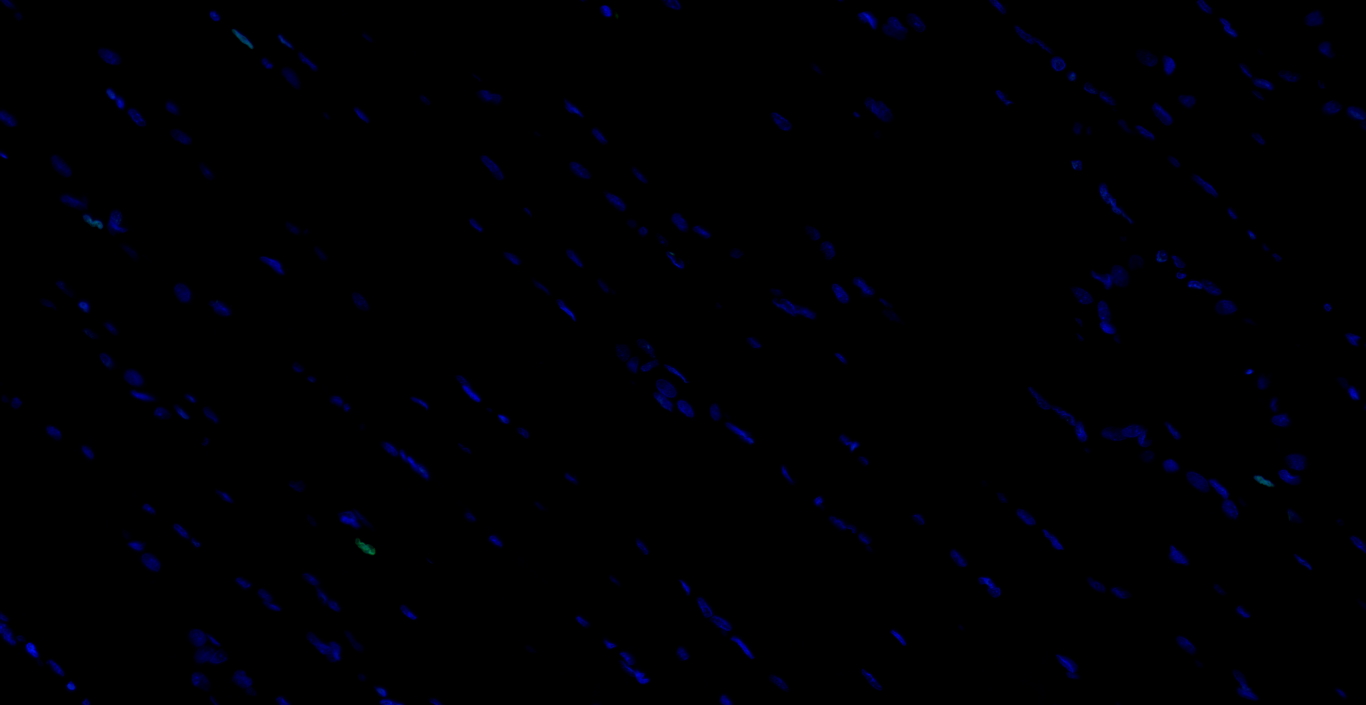

Supplement: Supplementary file 2 [file DataSheet4.ZIP › Supplemental materials 1/TUNEL/Sham/3-1 merge.jpg]

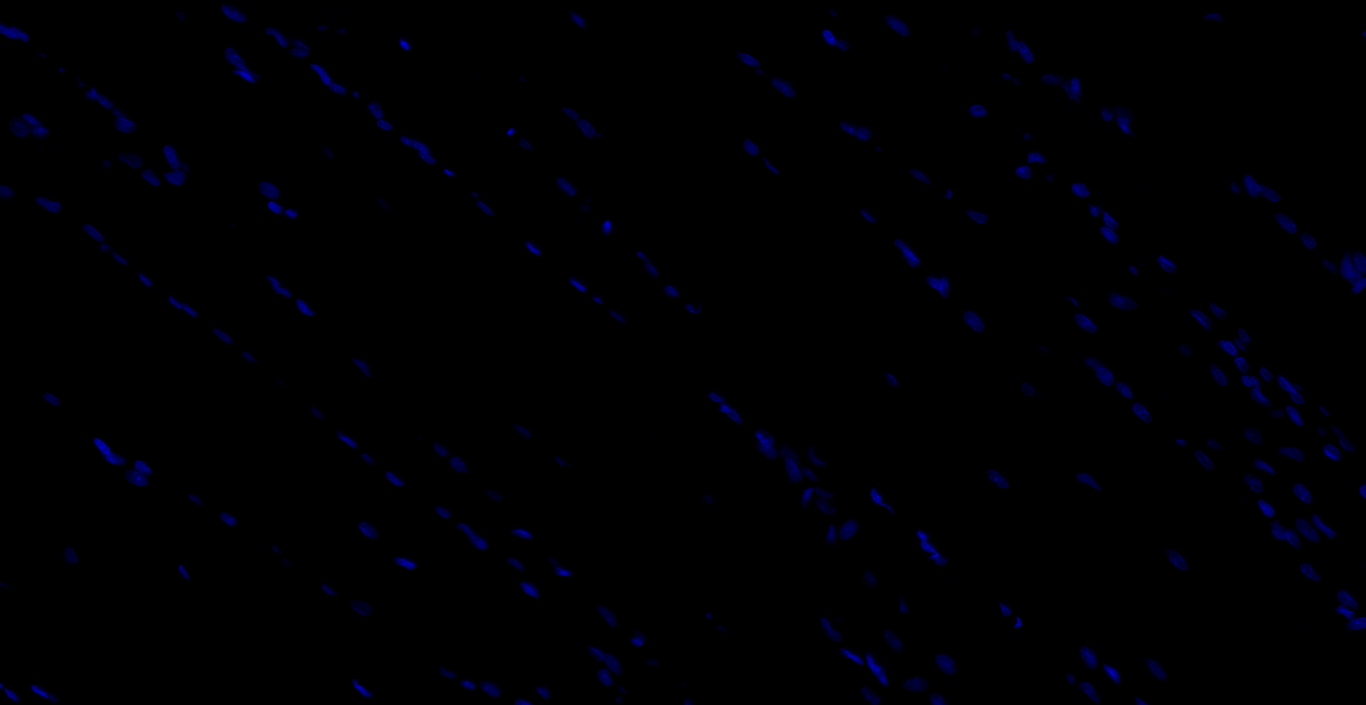

Supplement: Supplementary file 2 [file DataSheet4.ZIP › Supplemental materials 1/TUNEL/Sham/3-2 DAPI.jpg]

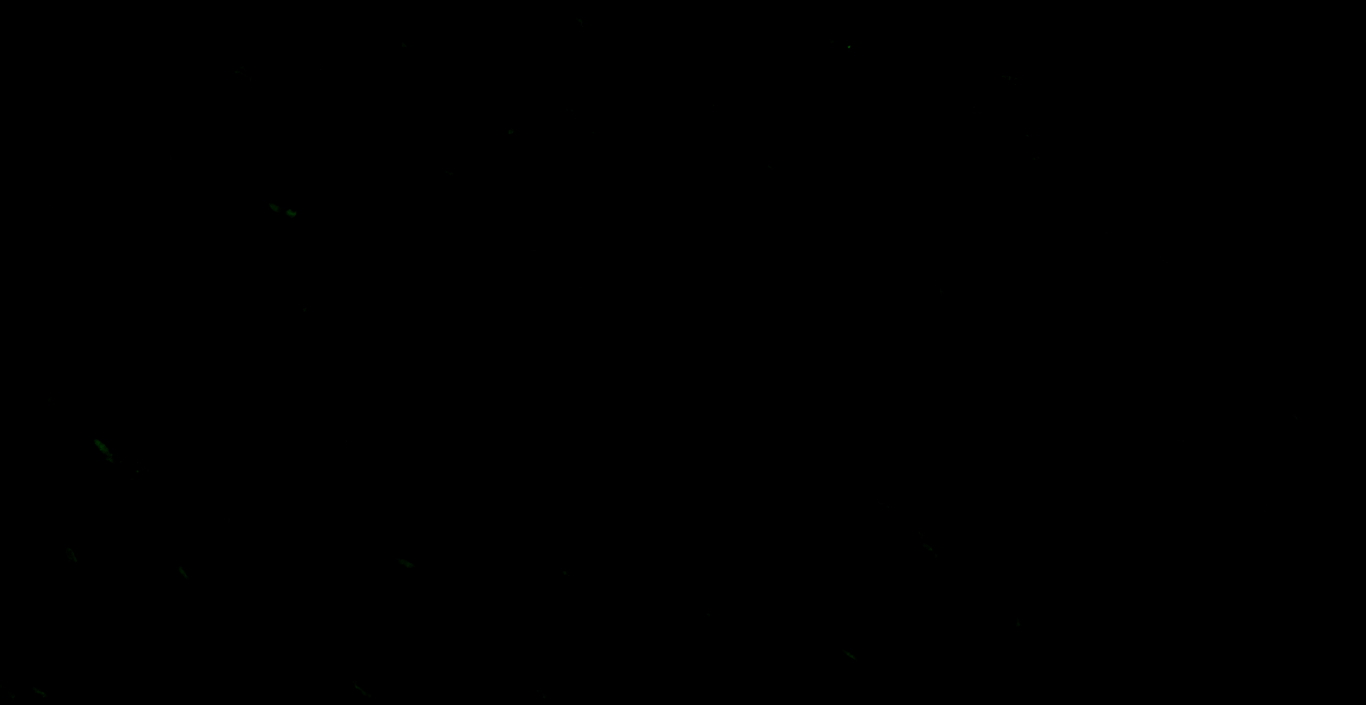

Supplement: Supplementary file 2 [file DataSheet4.ZIP › Supplemental materials 1/TUNEL/Sham/3-2 TUNEL.jpg]

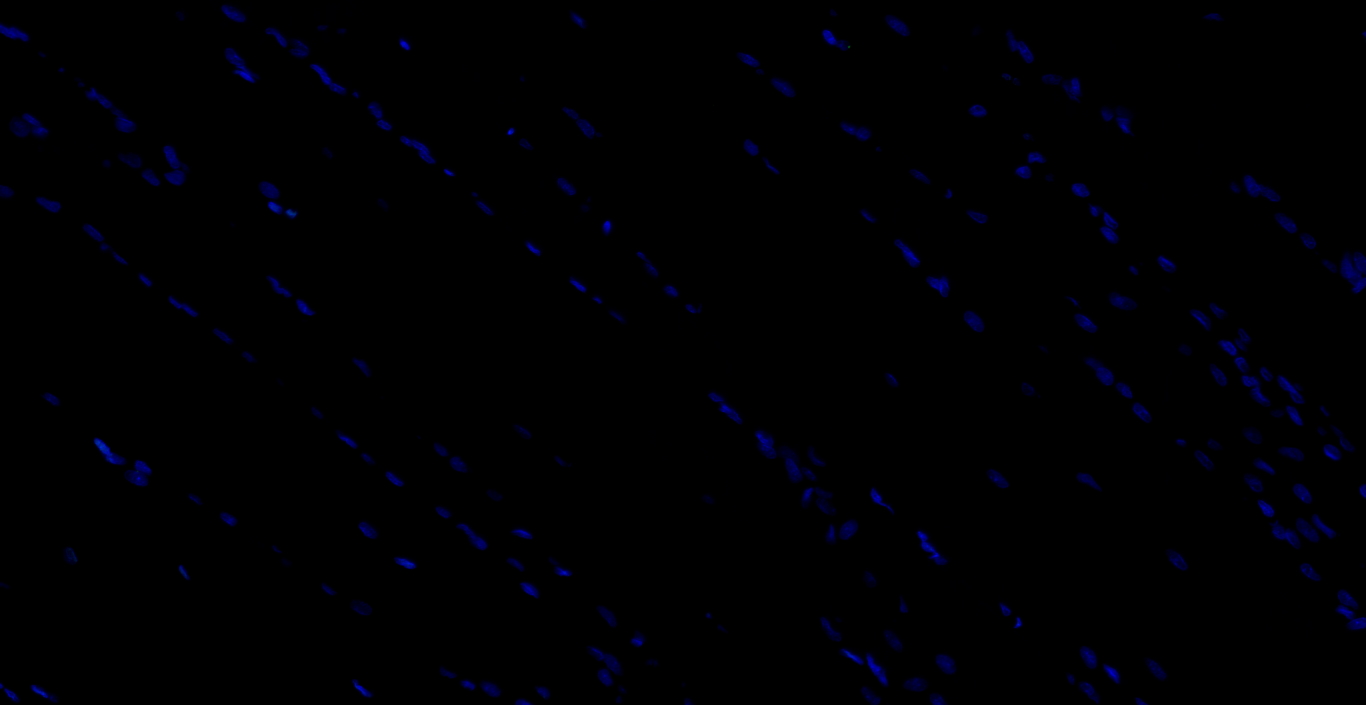

Supplement: Supplementary file 2 [file DataSheet4.ZIP › Supplemental materials 1/TUNEL/Sham/3-2 merge.jpg]

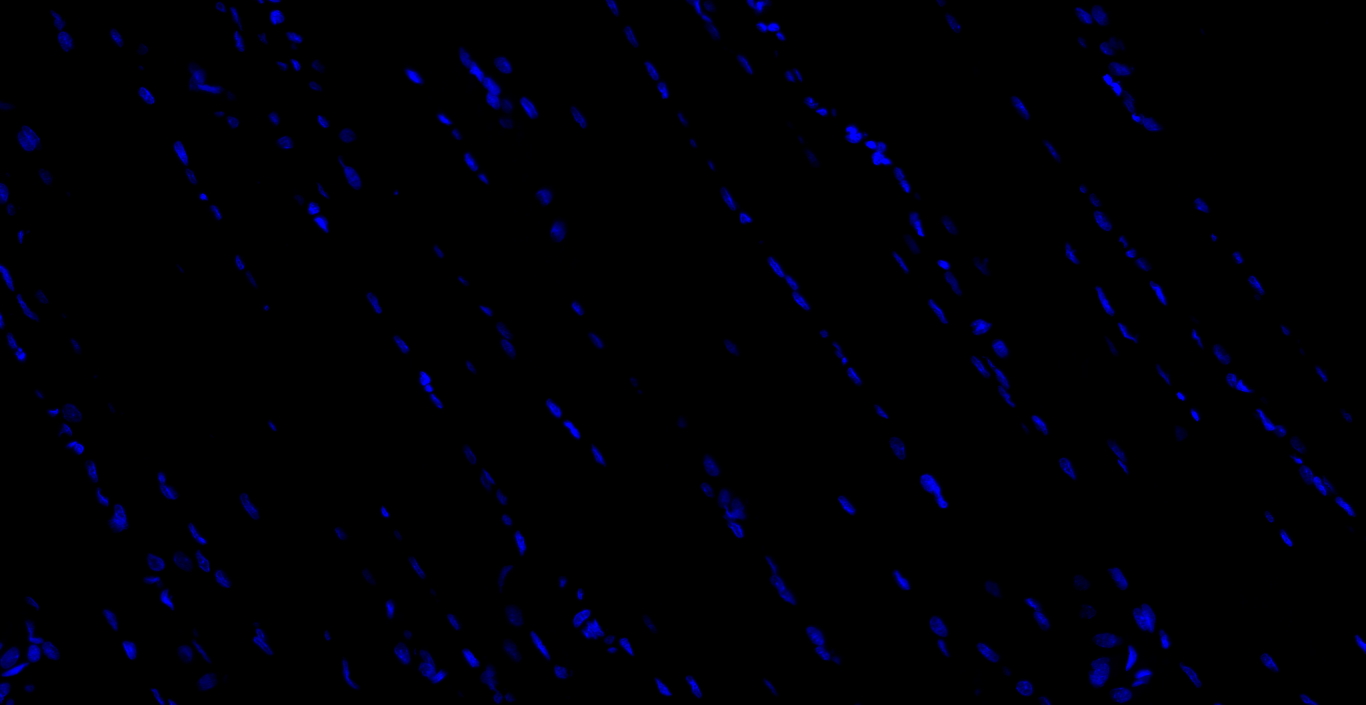

Supplement: Supplementary file 2 [file DataSheet4.ZIP › Supplemental materials 1/TUNEL/Sham/3-3 DAPI.jpg]

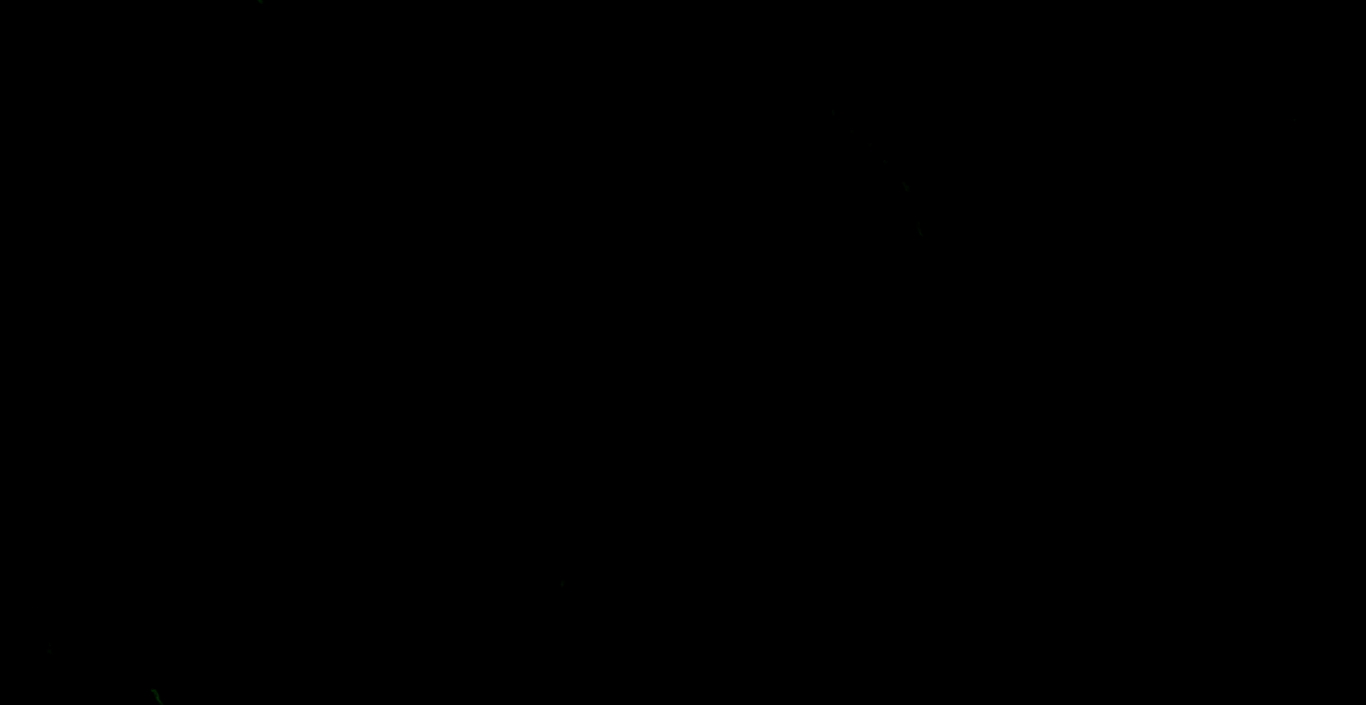

Supplement: Supplementary file 2 [file DataSheet4.ZIP › Supplemental materials 1/TUNEL/Sham/3-3 TUNEL.jpg]

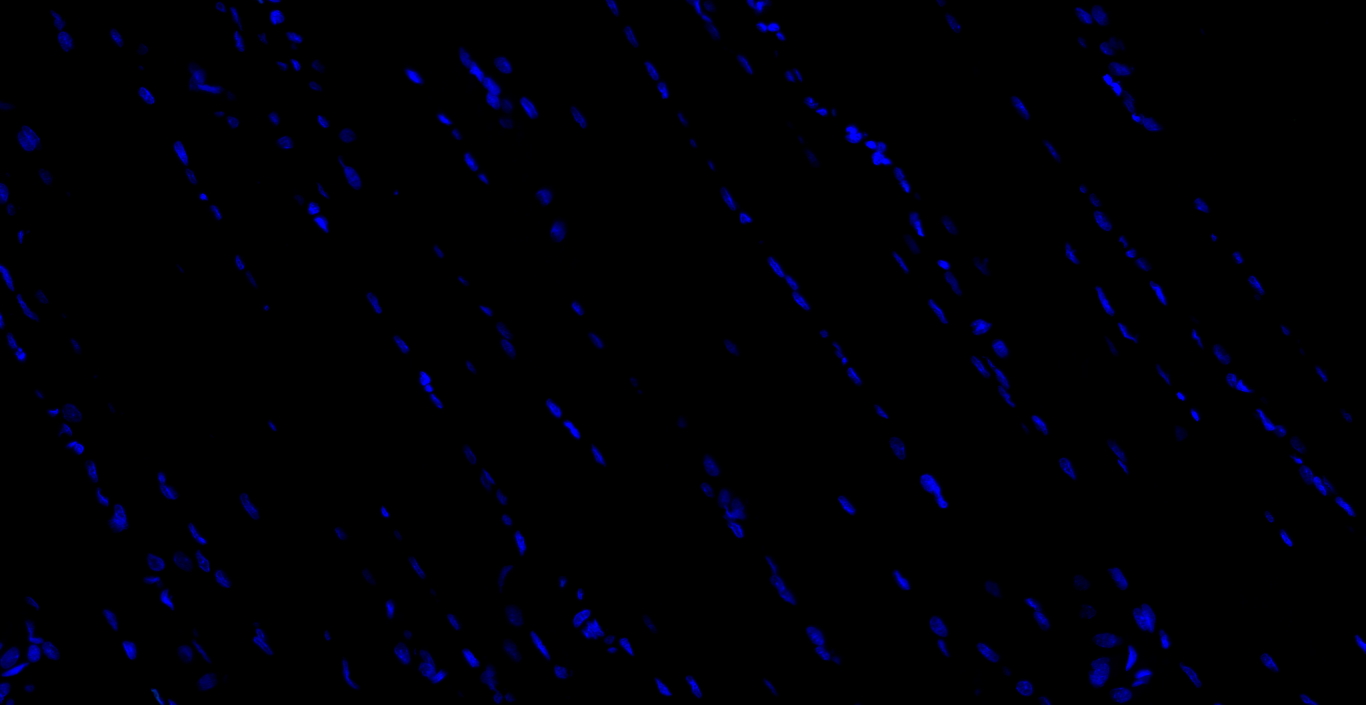

Supplement: Supplementary file 2 [file DataSheet4.ZIP › Supplemental materials 1/TUNEL/Sham/3-3 merge.jpg]

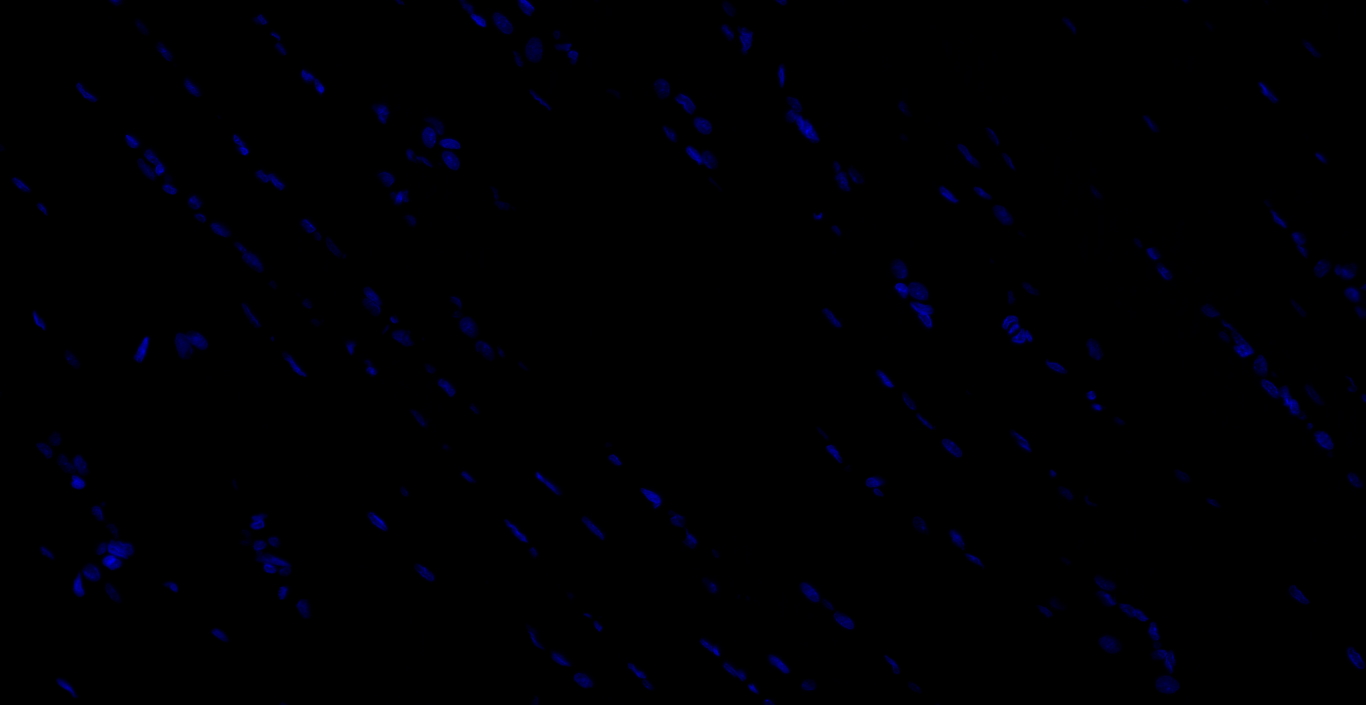

Supplement: Supplementary file 2 [file DataSheet4.ZIP › Supplemental materials 1/TUNEL/Sham/4-1 DAPI.jpg]

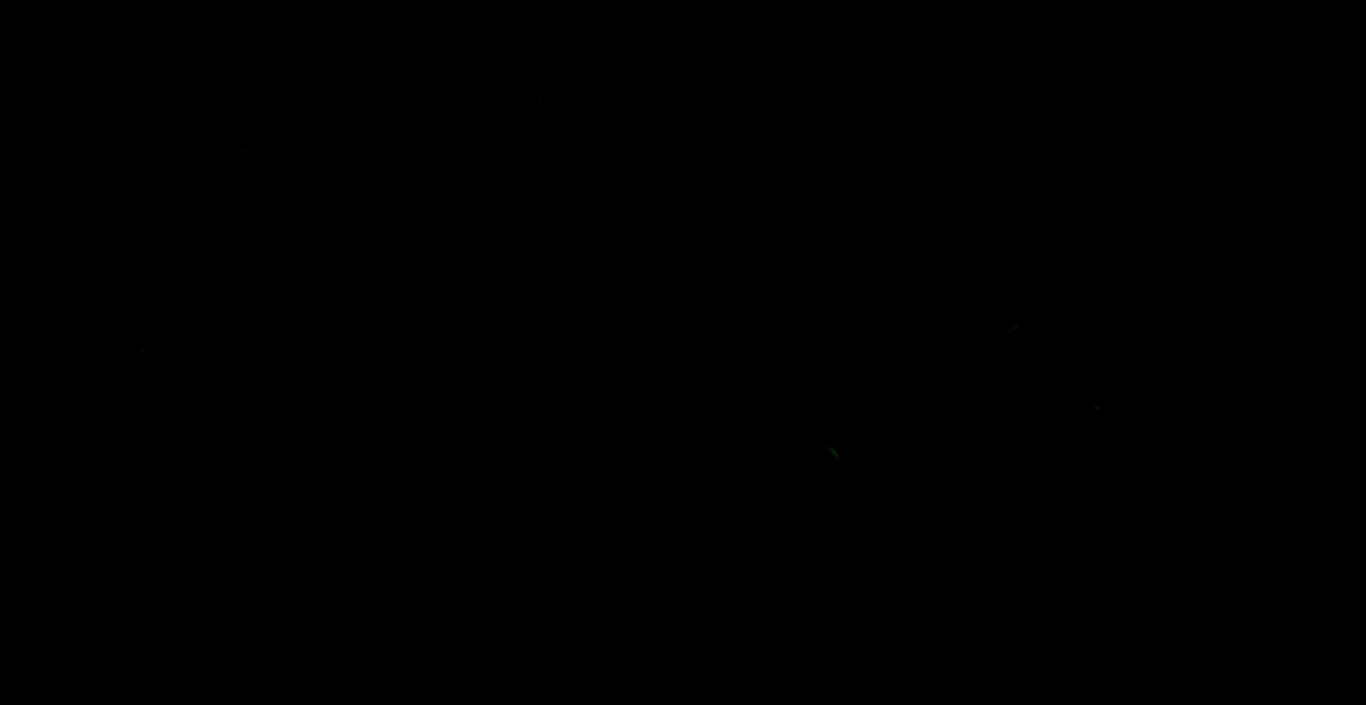

Supplement: Supplementary file 2 [file DataSheet4.ZIP › Supplemental materials 1/TUNEL/Sham/4-1 TUNEL.jpg]

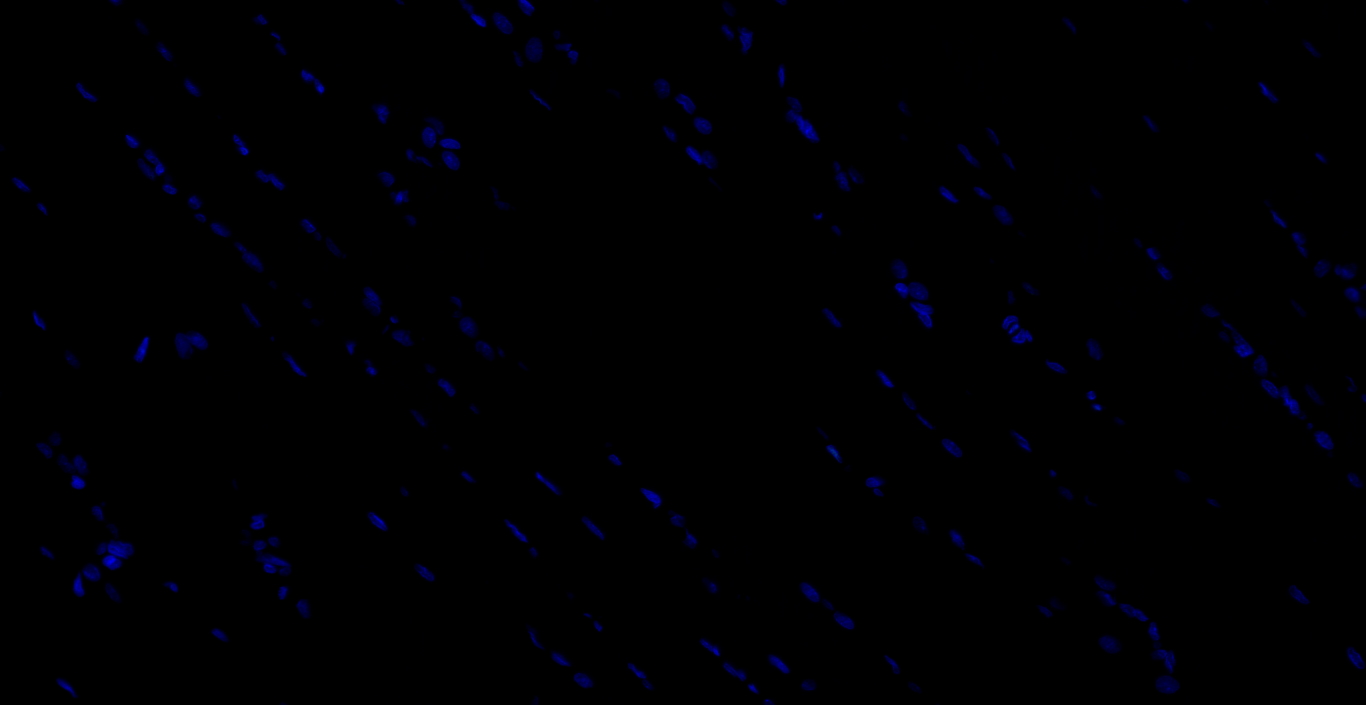

Supplement: Supplementary file 2 [file DataSheet4.ZIP › Supplemental materials 1/TUNEL/Sham/4-1merge.jpg]

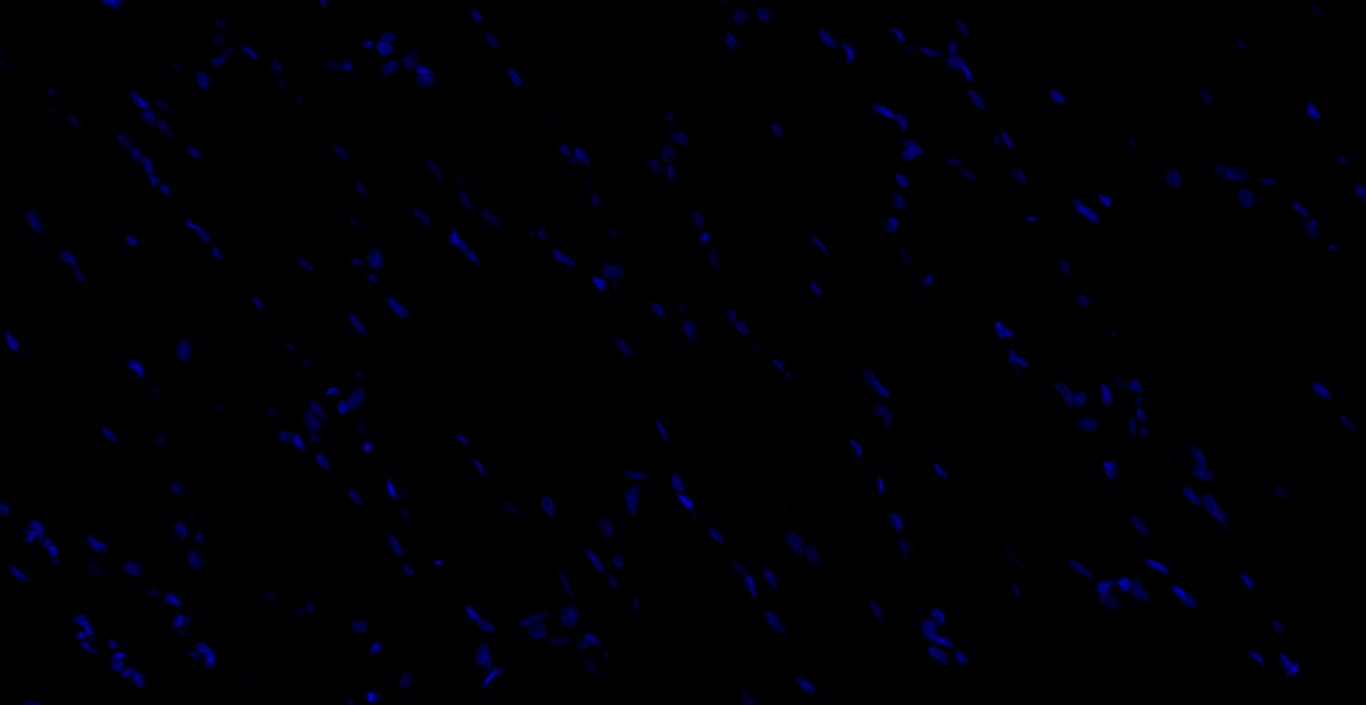

Supplement: Supplementary file 2 [file DataSheet4.ZIP › Supplemental materials 1/TUNEL/Sham/4-2 DAPI.jpg]

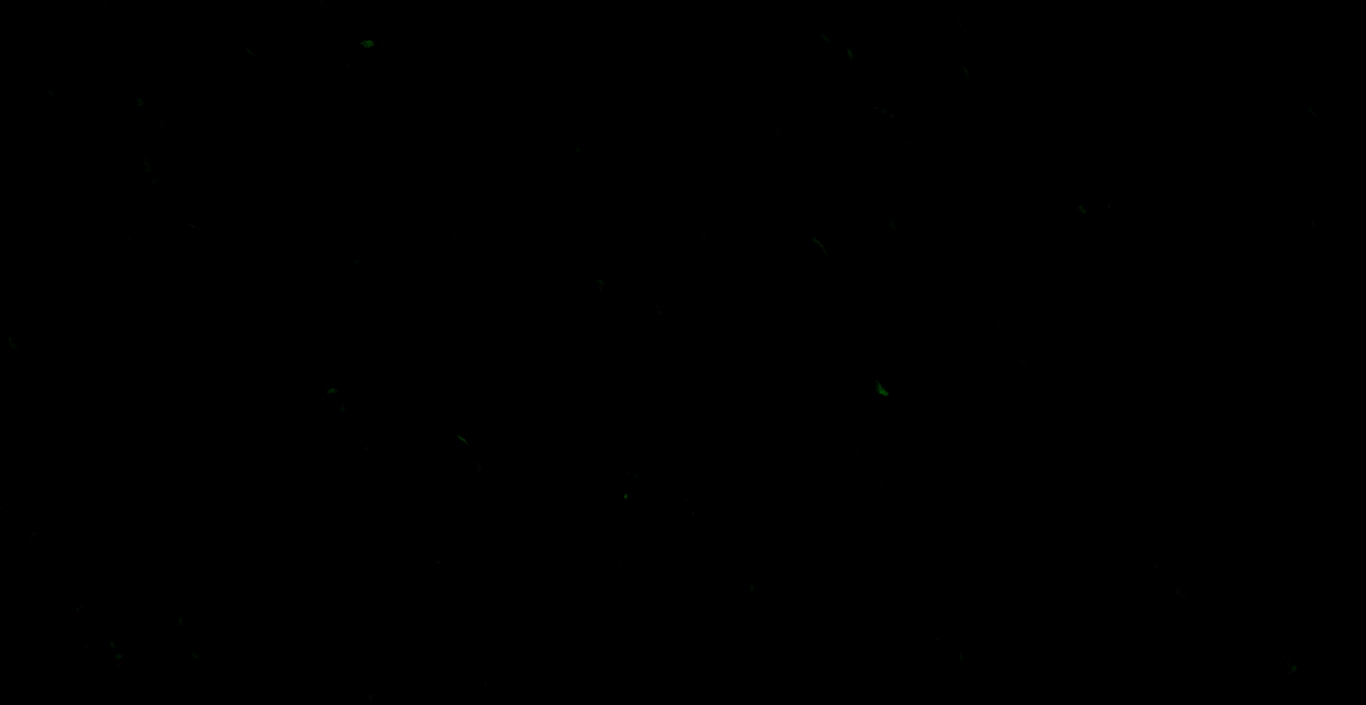

Supplement: Supplementary file 2 [file DataSheet4.ZIP › Supplemental materials 1/TUNEL/Sham/4-2 TUNEL.jpg]

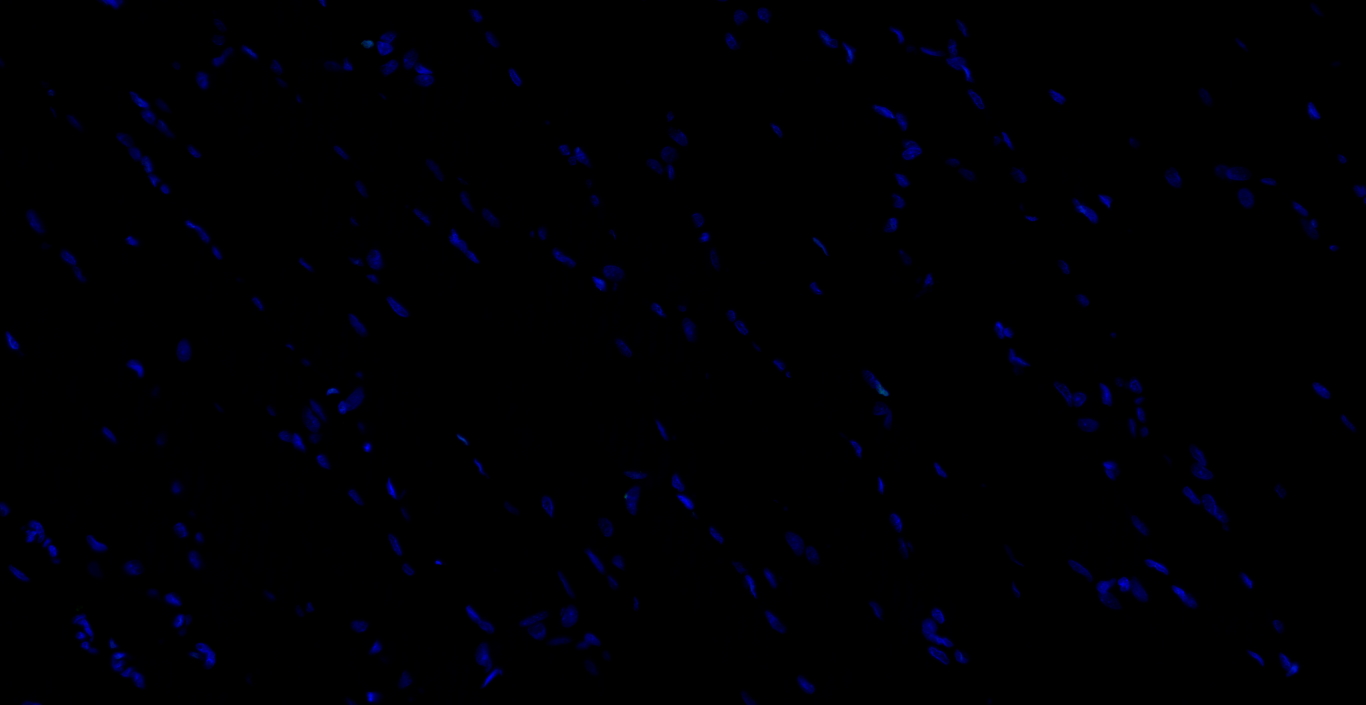

Supplement: Supplementary file 2 [file DataSheet4.ZIP › Supplemental materials 1/TUNEL/Sham/4-2merge.jpg]
