# Supplementary material for: Vagus Nerve Stimulation Attenuates Acute Skeletal Muscle Injury Induced by Hepatic Ischemia/Reperfusion Injury in Rats
Source: Front Pharmacol. 2022 Jan 3;12:756997. doi: 10.3389/fphar.2021.756997 (PMC8762262; doi:10.3389/fphar.2021.756997)
Supplement: Supplementary file 2 [file DataSheet4.ZIP › Supplemental materials 1/Figures PPT.pptx]

## Slide 1
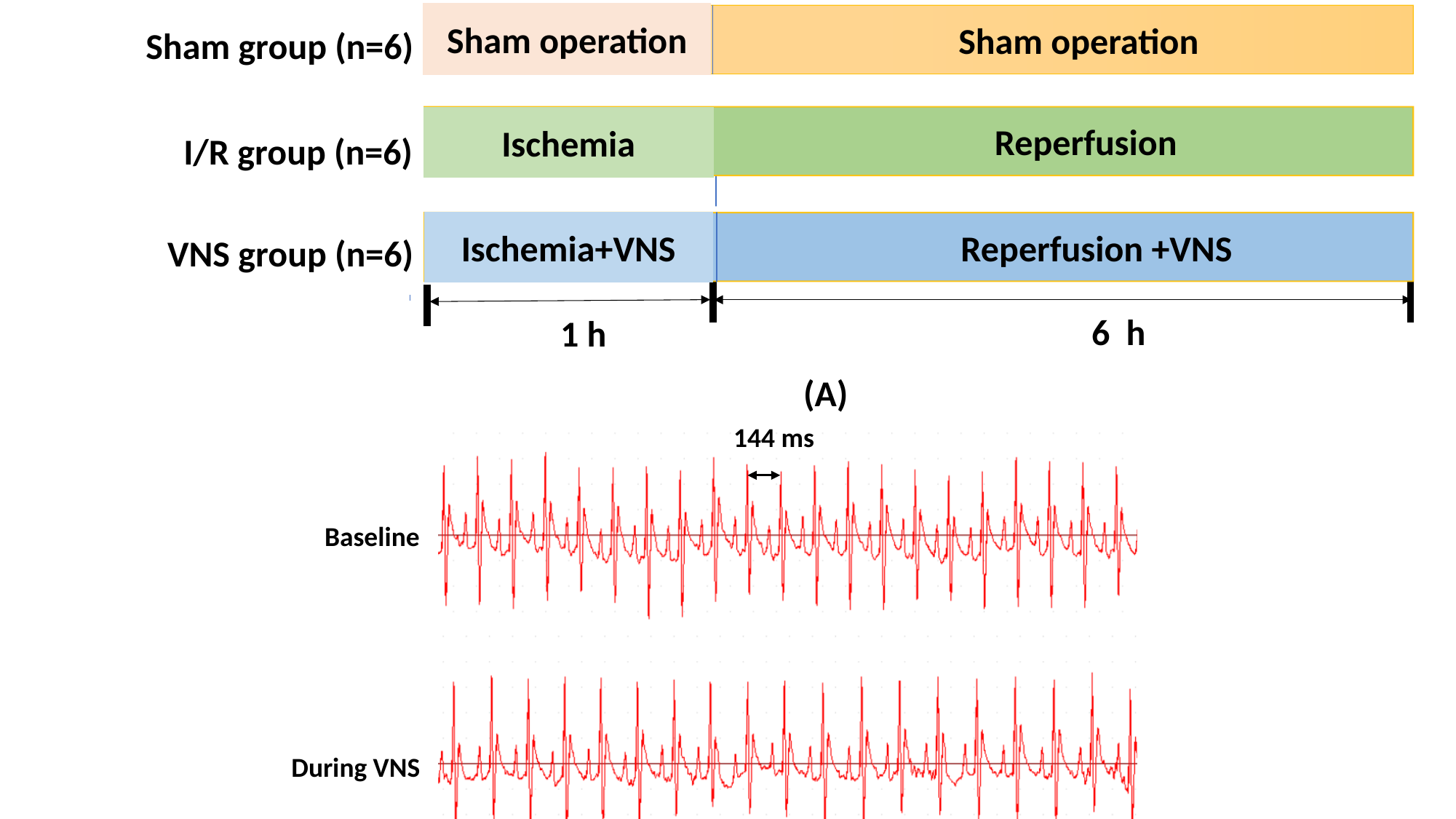

Sham operation
 Sham operation Sham operation
Sham group (n=6)
 Ischemia Reperfusion
Ischemia
I/R group (n=6)
 Ischemia + VNS Reperfusion +VNS
VNS group (n=6)
Ischemia+VNS
6 h
1 h
(A)
144 ms
Baseline
During VNS
Electrical stimulus signal
164 ms
(B)

## Slide 2
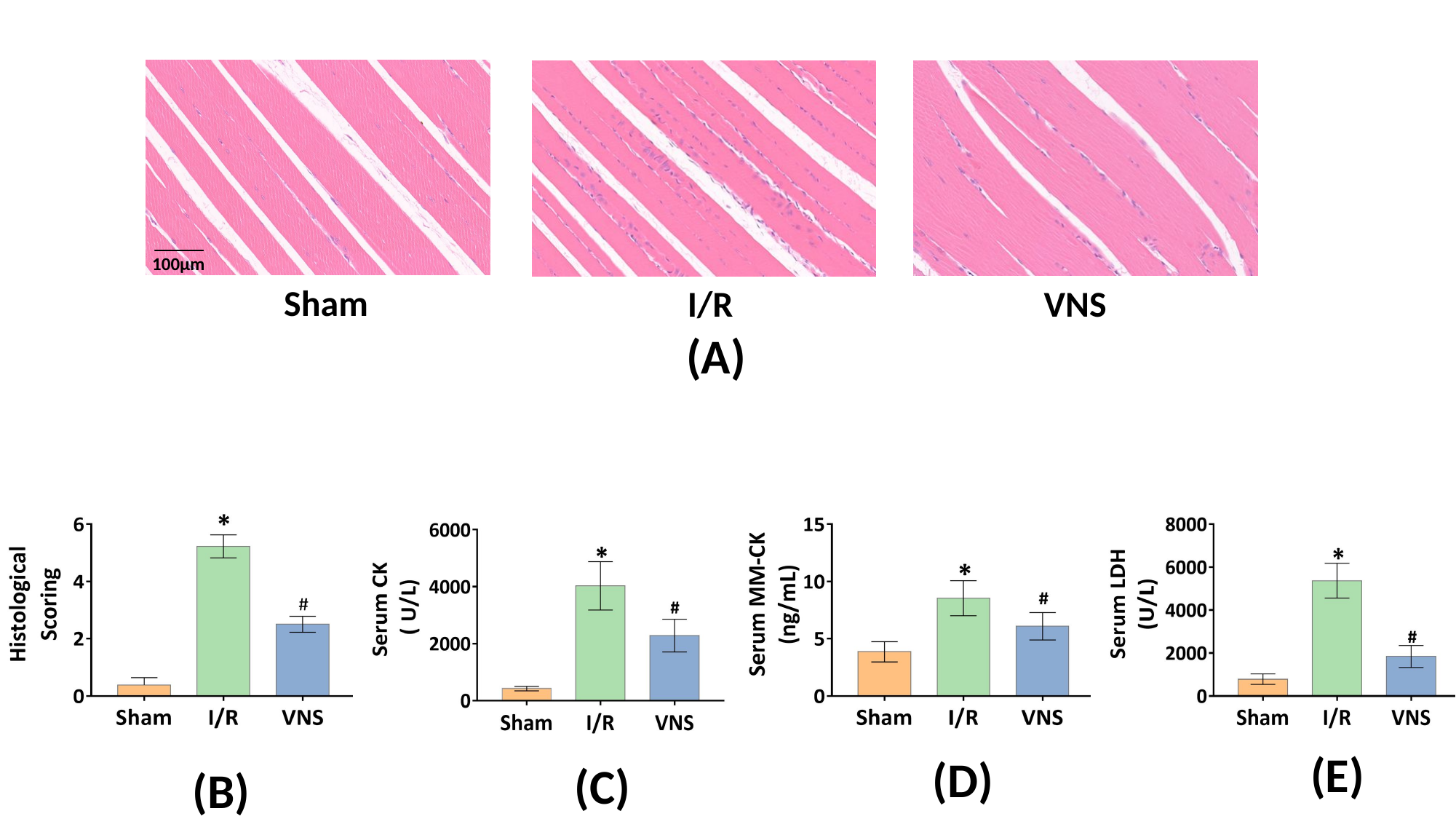

100μm
Sham
I/R
VNS
100μm
(A)
(B)
(D)
(E)
(C)

## Slide 3
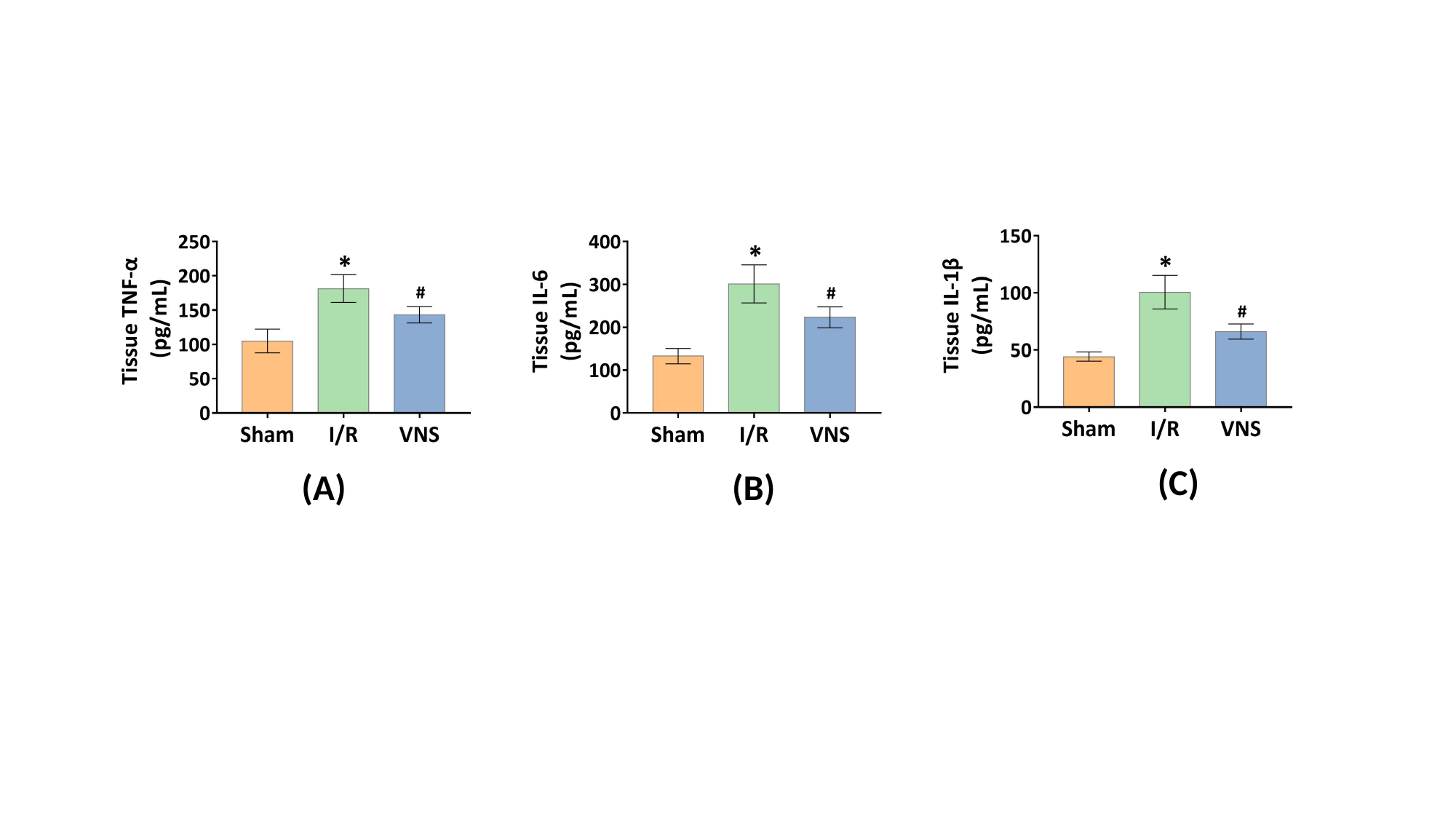

(C)
(A)
(B)

## Slide 4
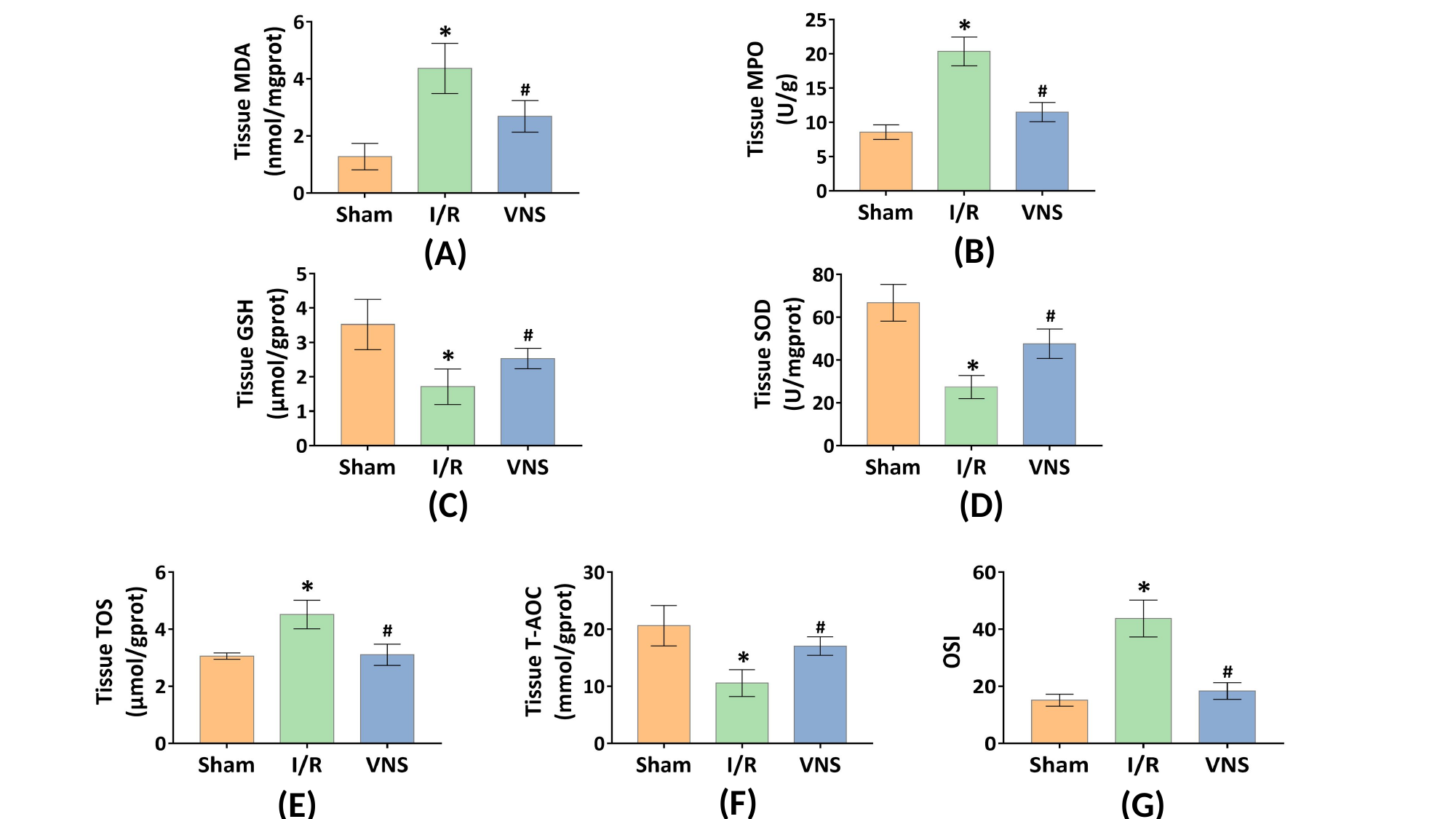

(B)
(A)
(C)
(D)
(E)
(F)
(G)

## Slide 5
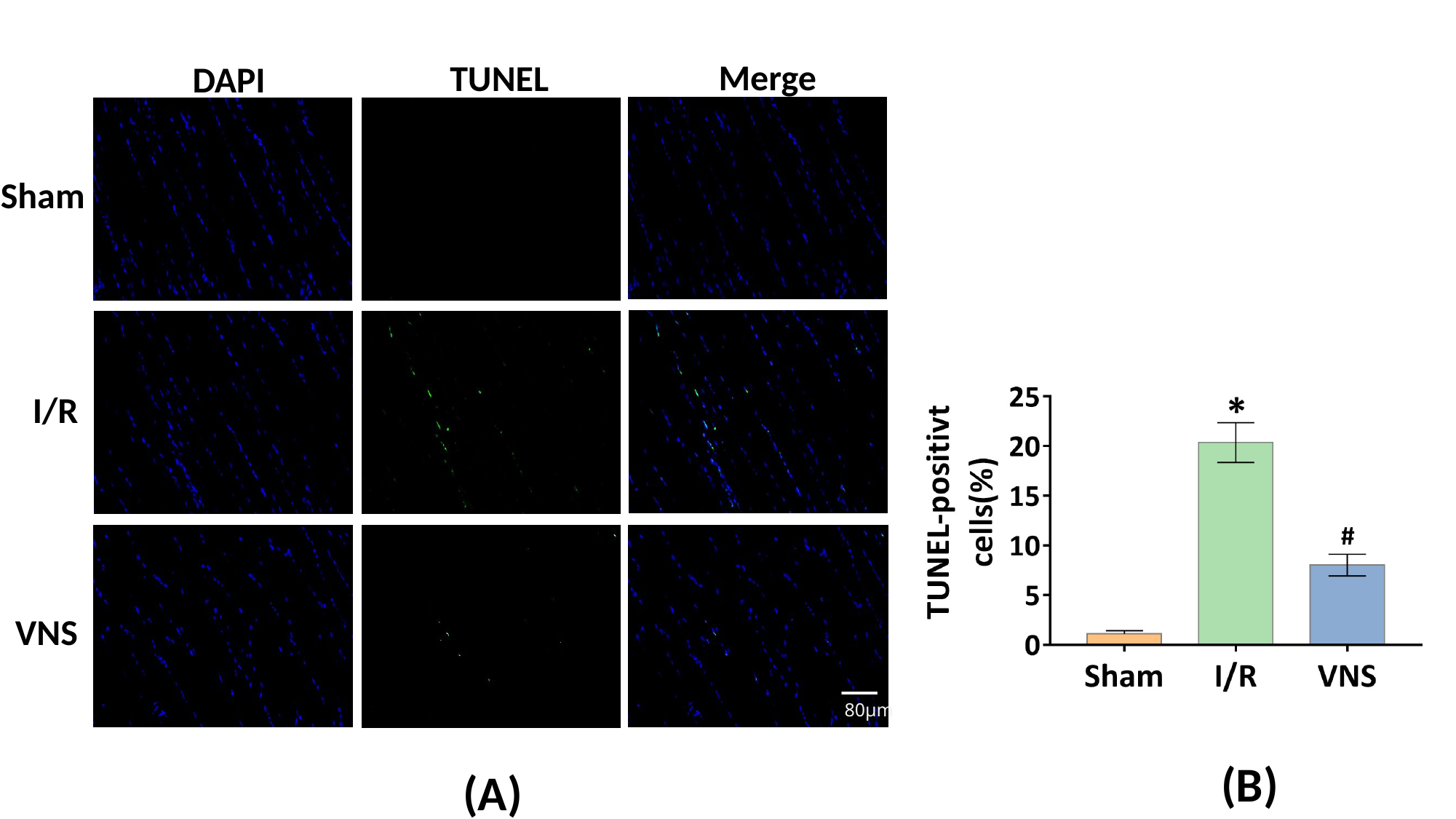

Merge
TUNEL
DAPI
80μm
Sham
I/R
VNS
(B)
(A)

## Slide 6
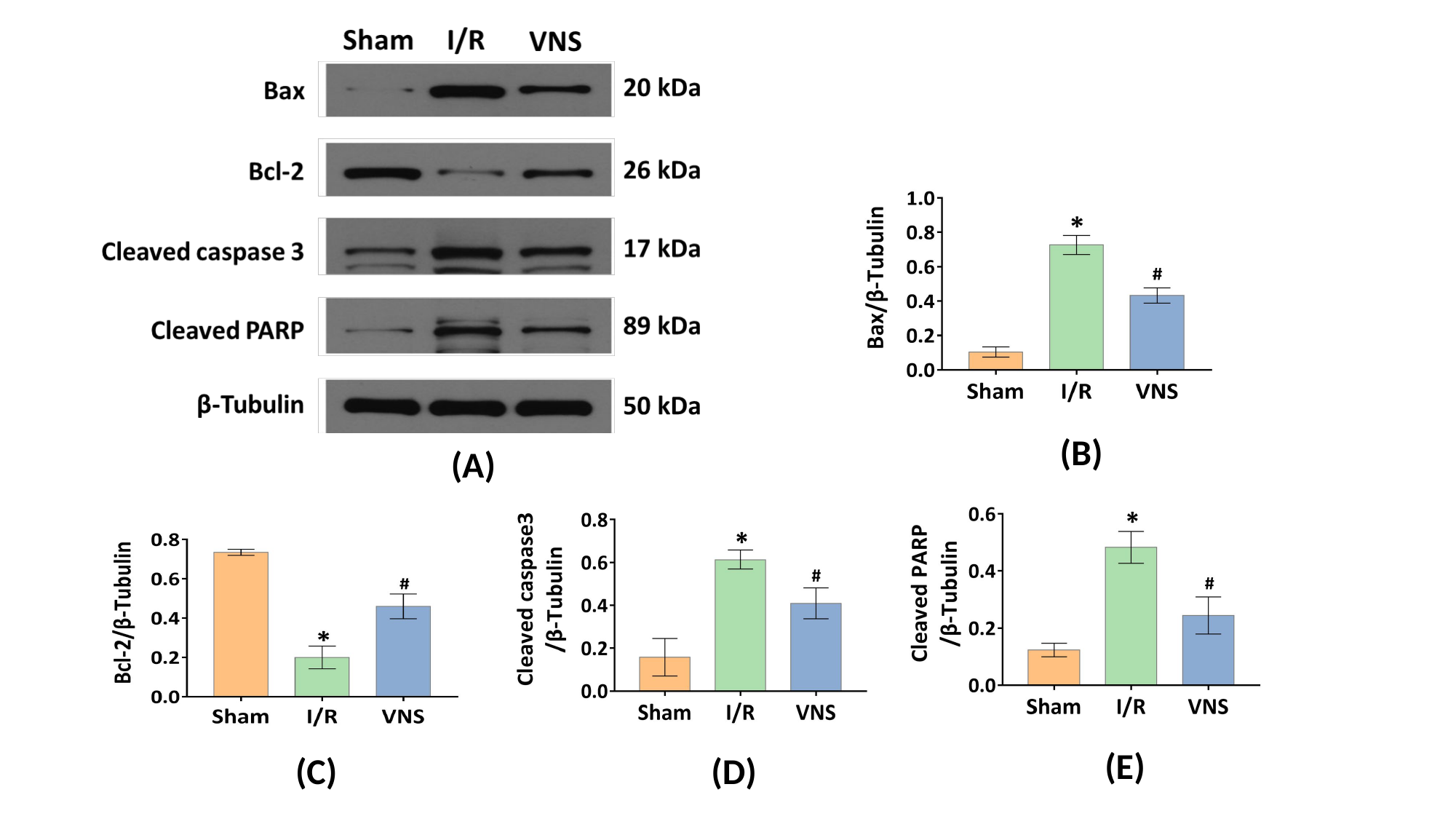

(A)
(B)
(E)
(D)
(C)

## Slide 7
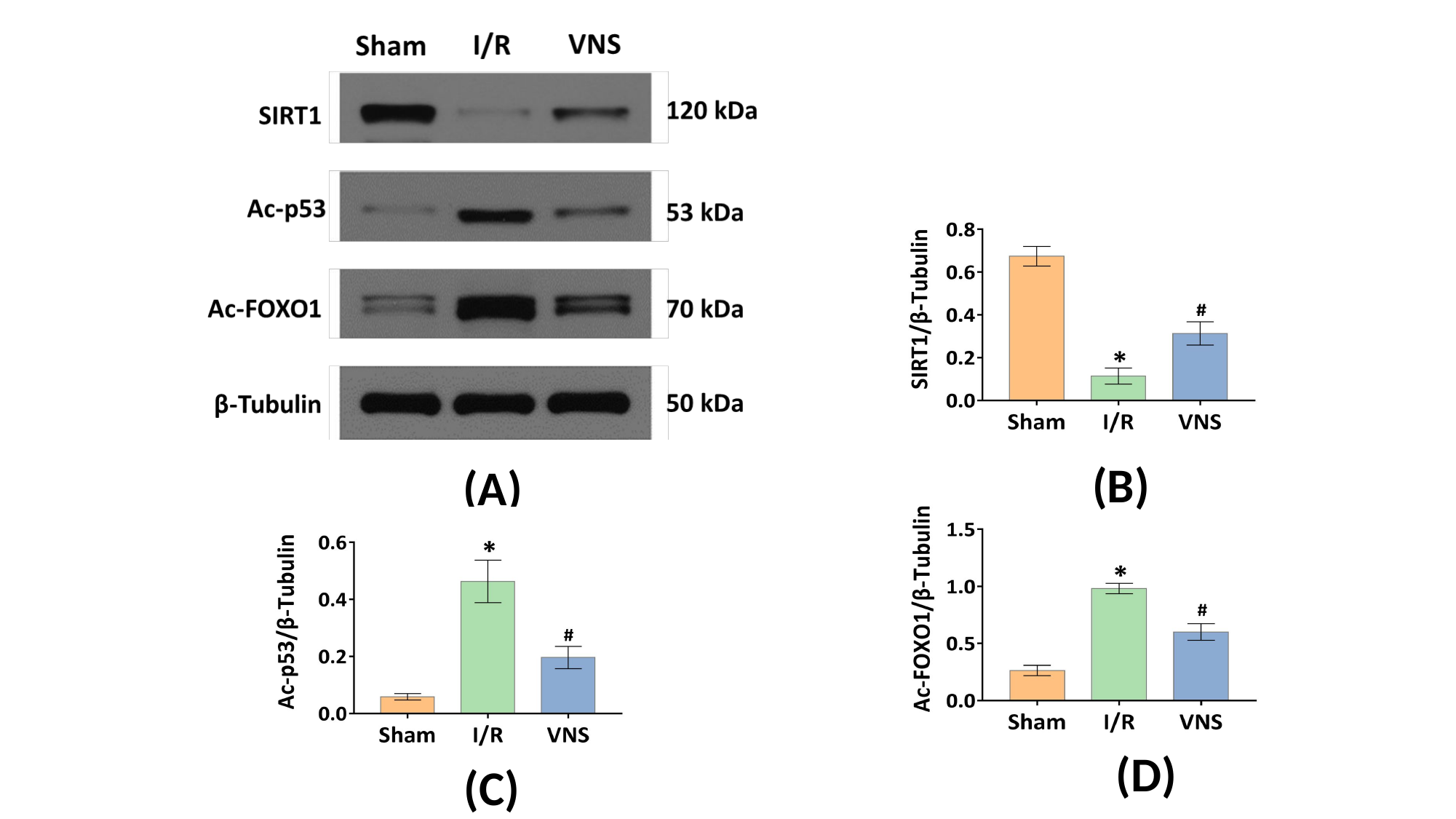

(A)
(B)
(D)
(C)

## Slide 8
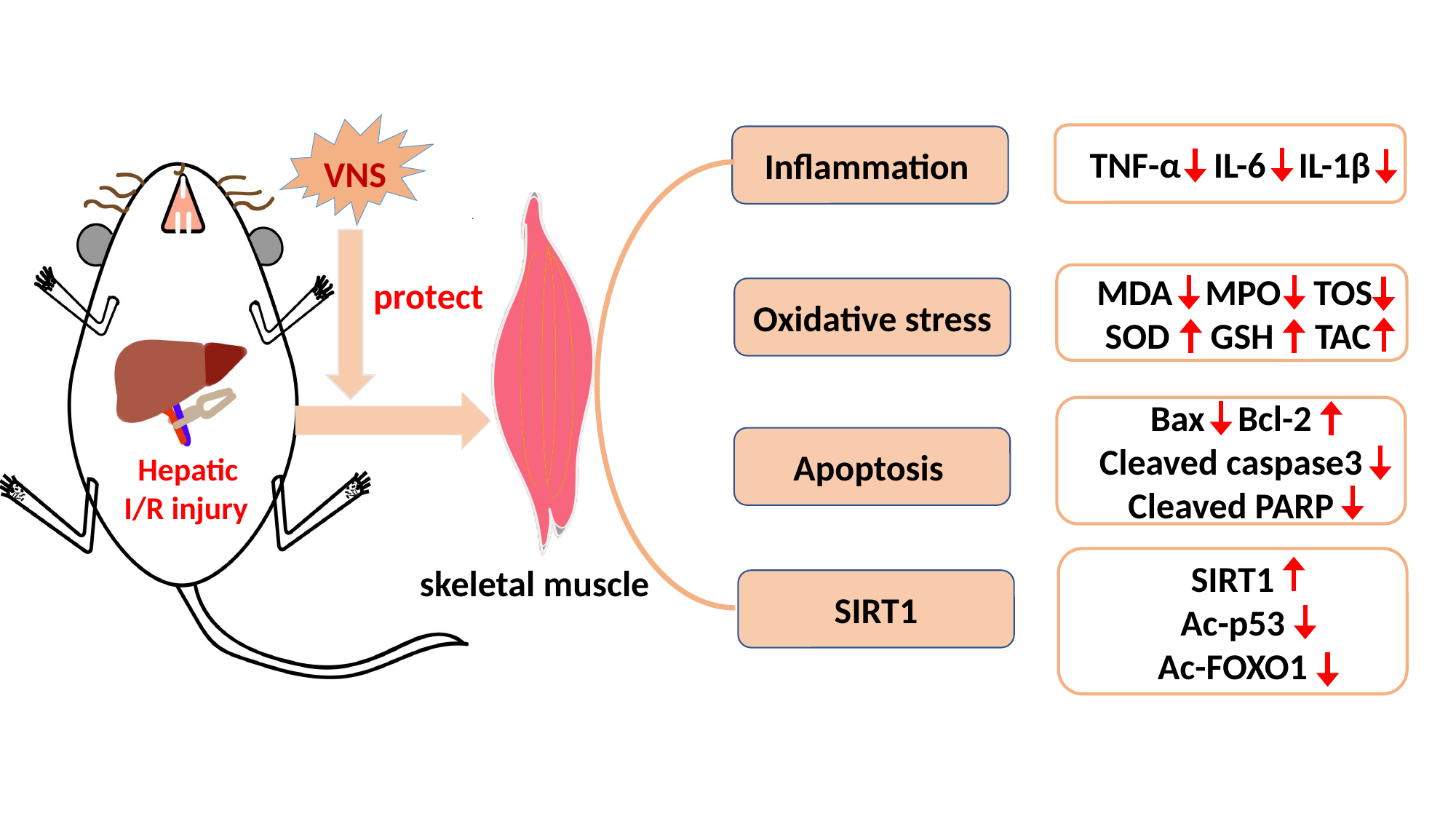

VNS
protect
TNF-α IL-6 IL-1β
Inflammation
Oxidative stress
Apoptosis
SIRT1
 Hepatic
I/R injury
 MDA MPO TOS
 SOD GSH TAC
Bax Bcl-2
Cleaved caspase3
Cleaved PARP
SIRT1
Ac-p53
Ac-FOXO1
skeletal muscle
