# Supplementary figures and images for: Vagus Nerve Stimulation Attenuates Acute Skeletal Muscle Injury Induced by Hepatic Ischemia/Reperfusion Injury in Rats (part 2 of 3)
Source: Front Pharmacol. 2022 Jan 3;12:756997. doi: 10.3389/fphar.2021.756997 (PMC8762262; doi:10.3389/fphar.2021.756997)

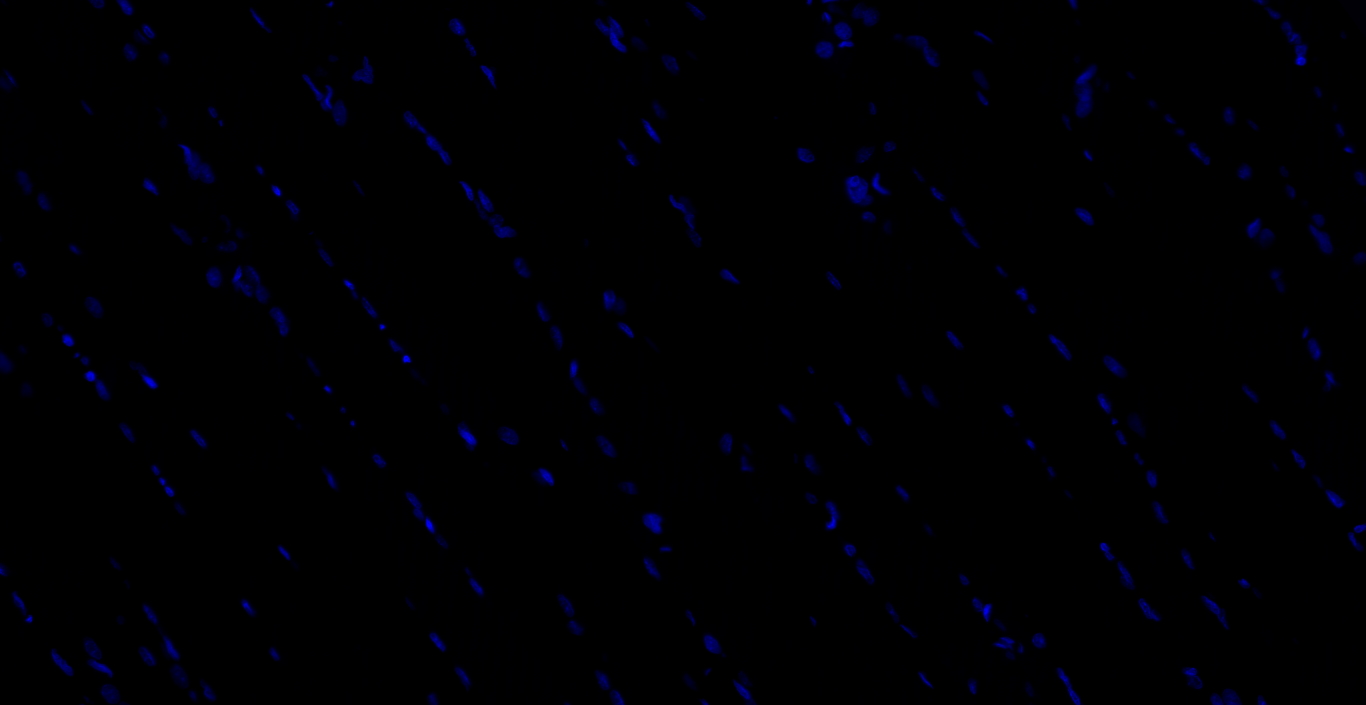

Supplement: Supplementary file 2 [file DataSheet4.ZIP › Supplemental materials 1/TUNEL/Sham/4-3 DAPI.jpg]

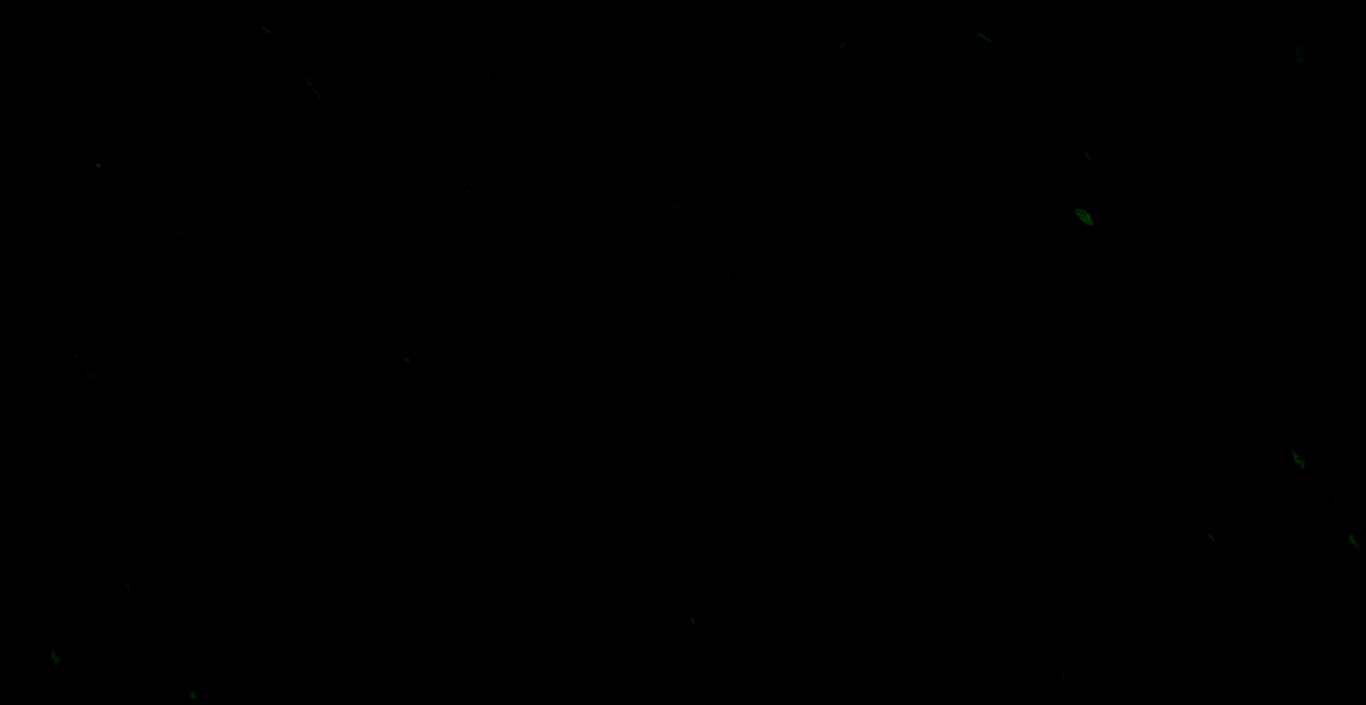

Supplement: Supplementary file 2 [file DataSheet4.ZIP › Supplemental materials 1/TUNEL/Sham/4-3 TUNEL.jpg]

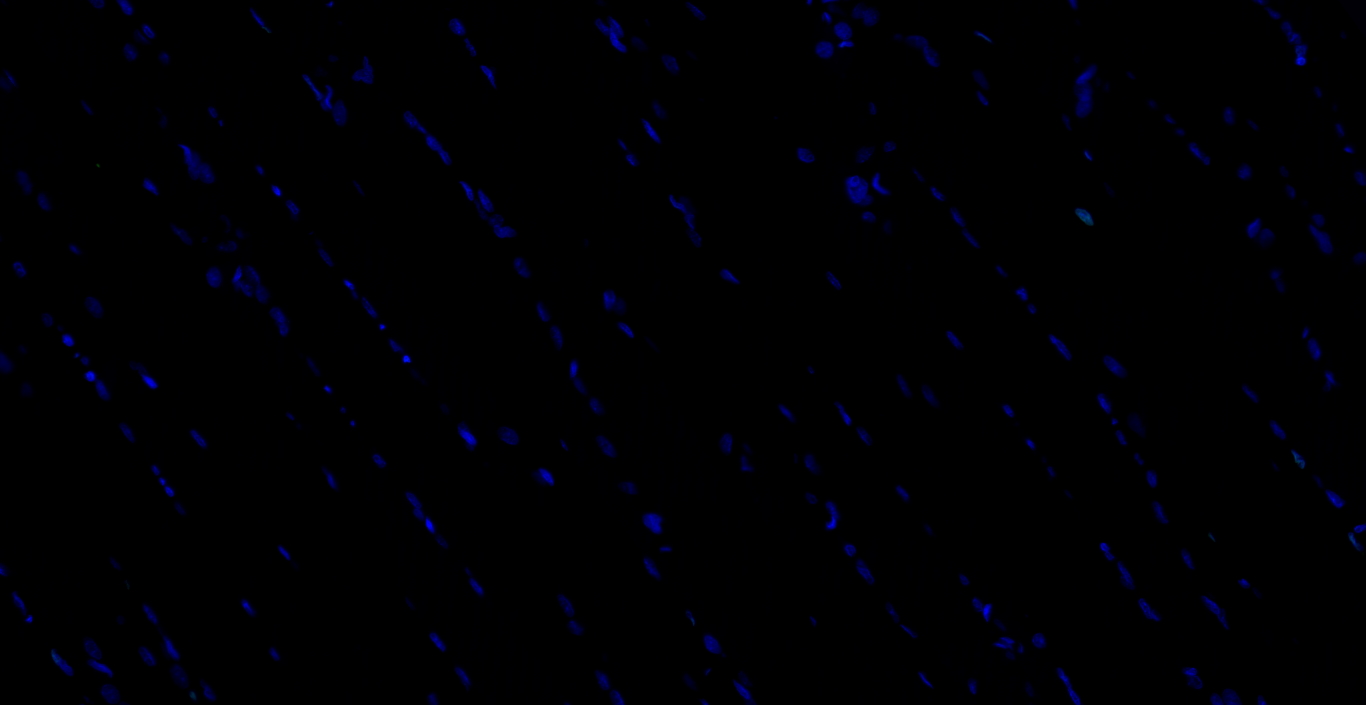

Supplement: Supplementary file 2 [file DataSheet4.ZIP › Supplemental materials 1/TUNEL/Sham/4-3 merge.jpg]

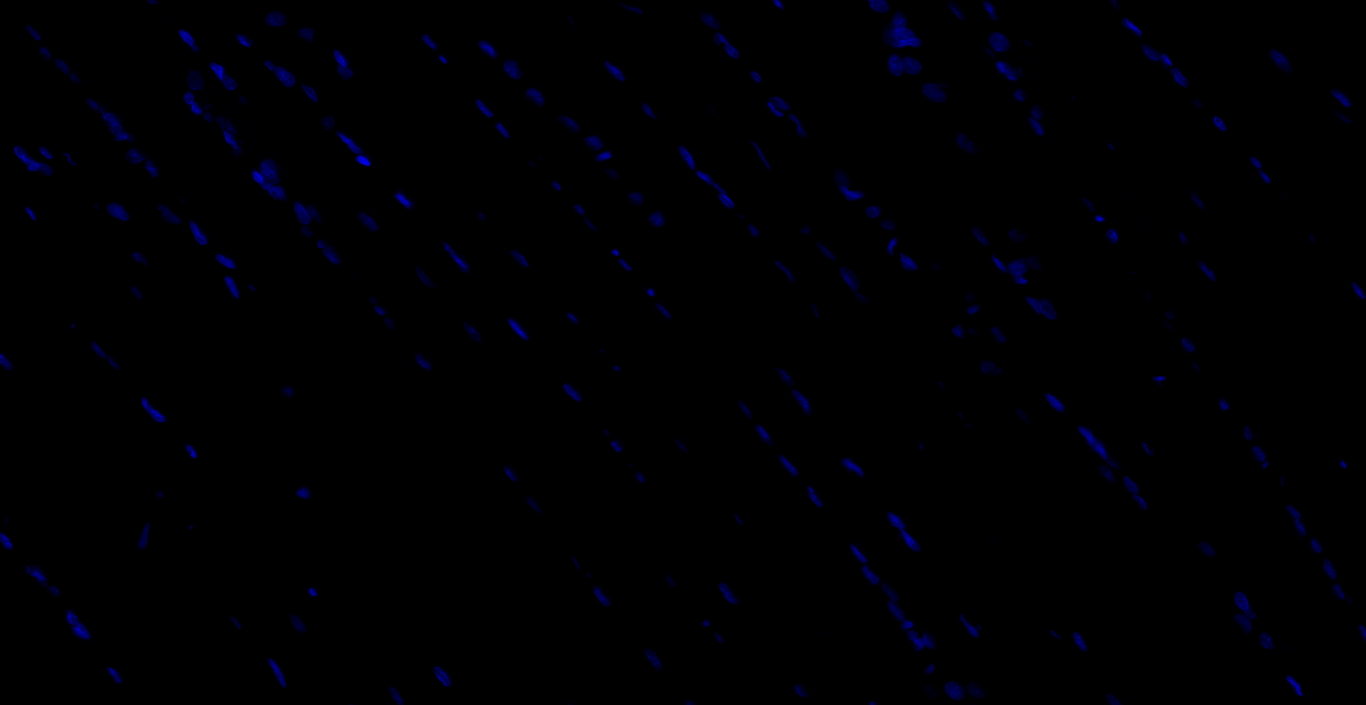

Supplement: Supplementary file 2 [file DataSheet4.ZIP › Supplemental materials 1/TUNEL/Sham/5-1 DAPI.jpg]

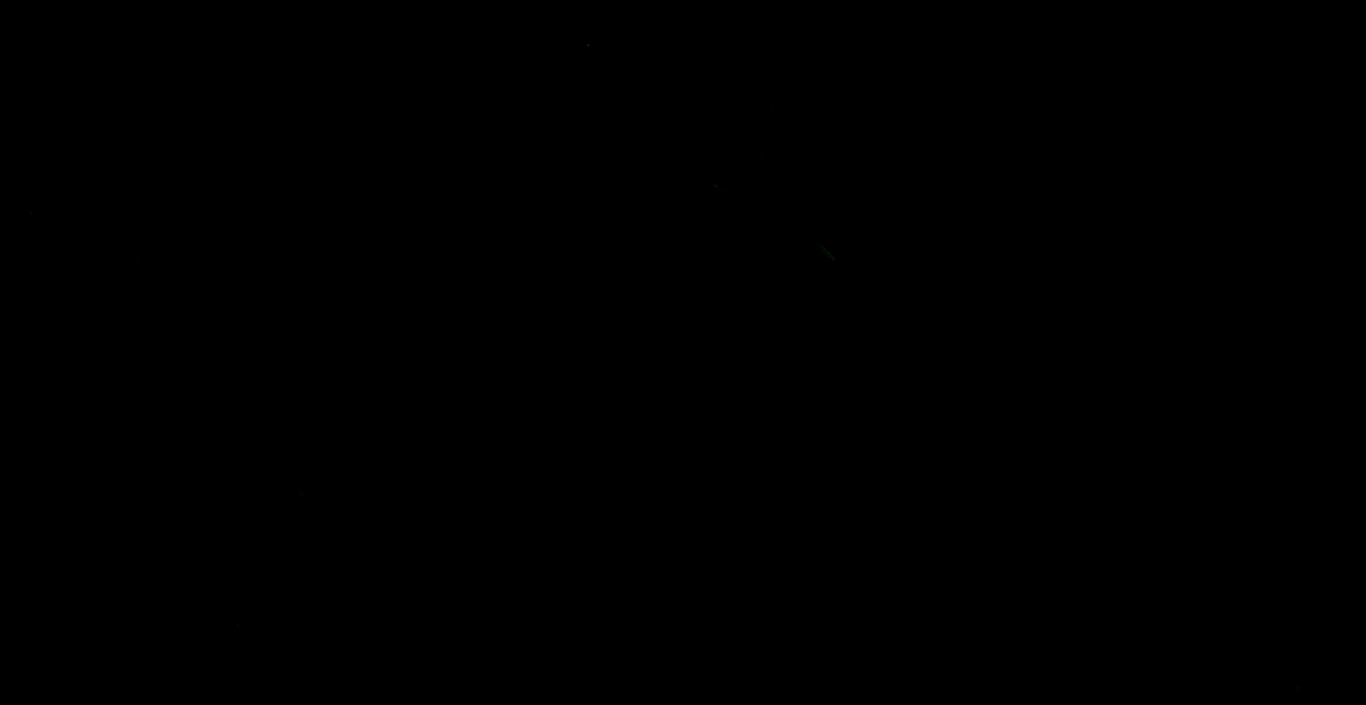

Supplement: Supplementary file 2 [file DataSheet4.ZIP › Supplemental materials 1/TUNEL/Sham/5-1 TUNEL.jpg]

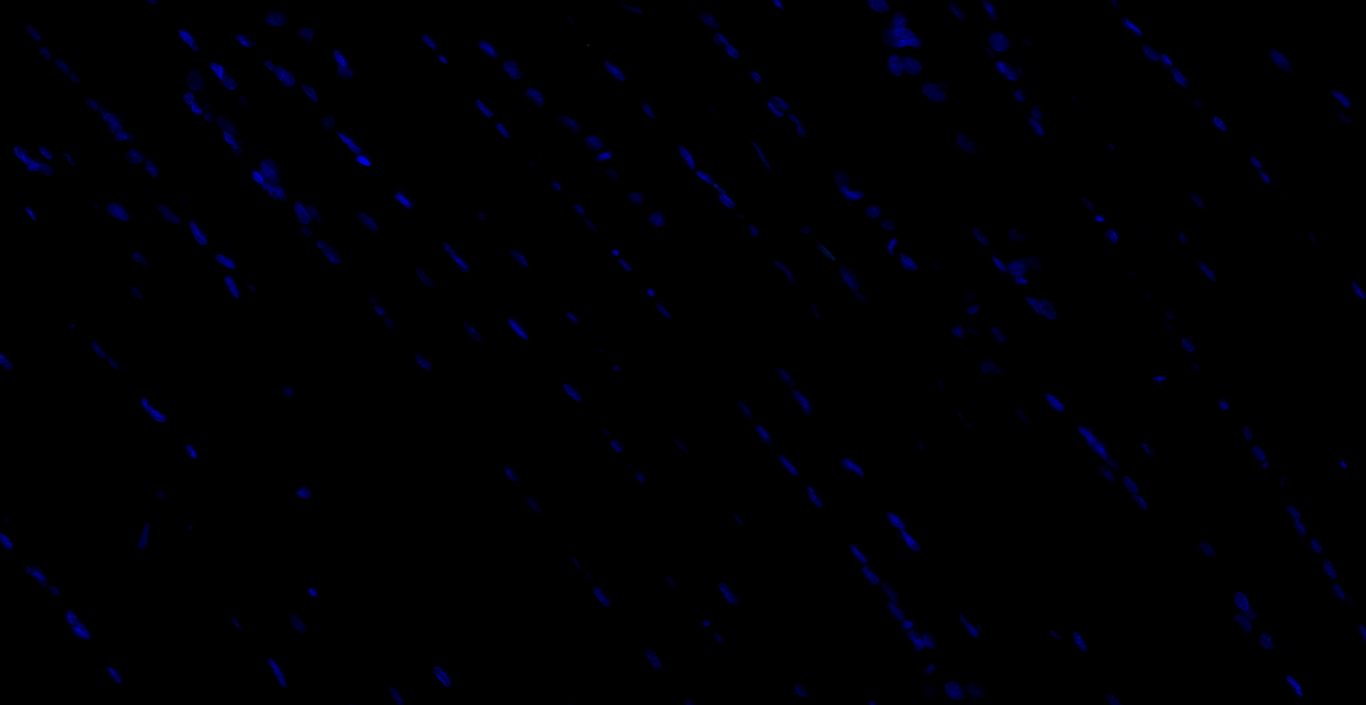

Supplement: Supplementary file 2 [file DataSheet4.ZIP › Supplemental materials 1/TUNEL/Sham/5-1 merge.jpg]

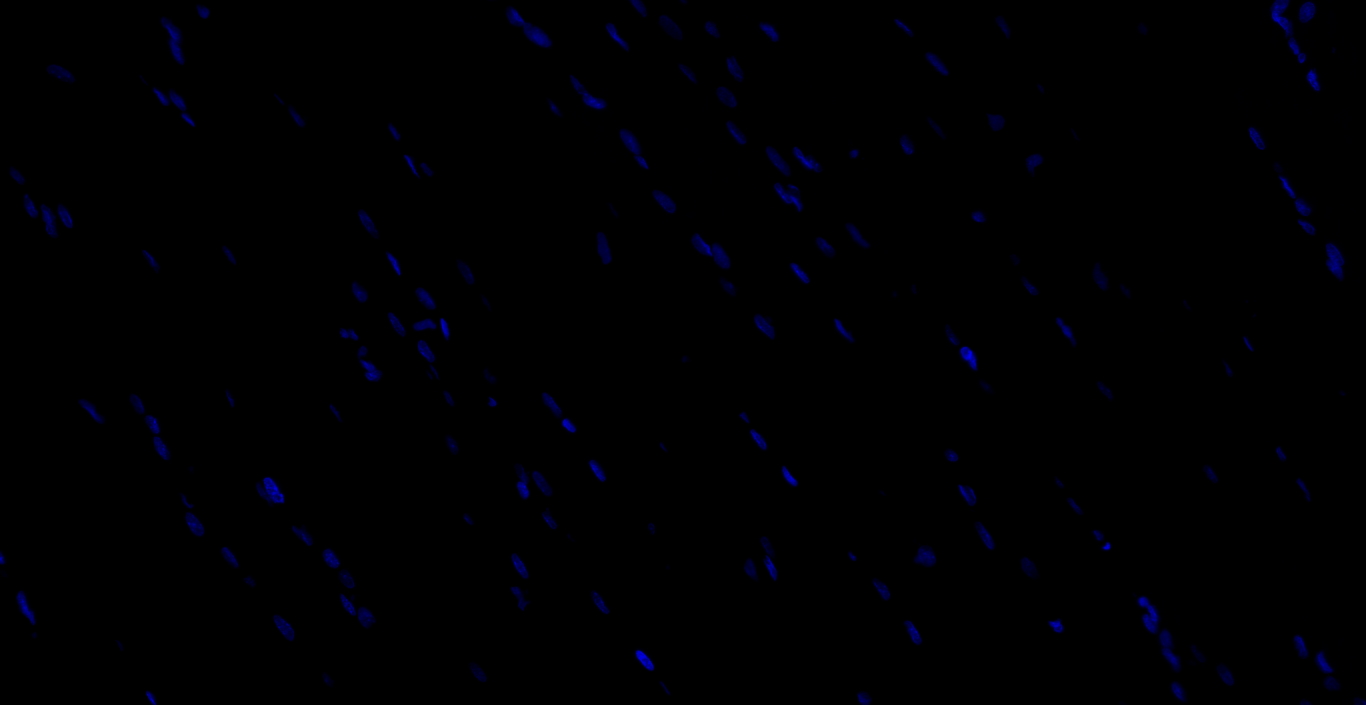

Supplement: Supplementary file 2 [file DataSheet4.ZIP › Supplemental materials 1/TUNEL/Sham/5-2 DAPI.jpg]

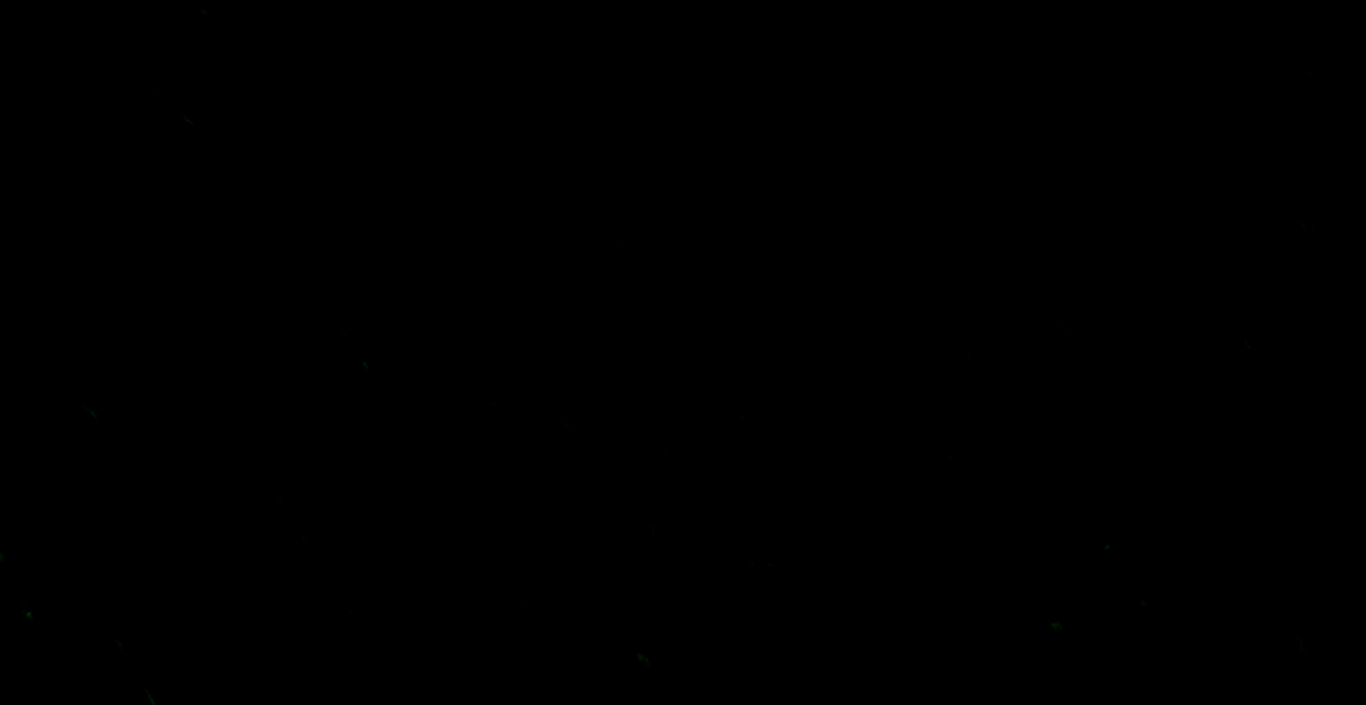

Supplement: Supplementary file 2 [file DataSheet4.ZIP › Supplemental materials 1/TUNEL/Sham/5-2 TUNEL.jpg]

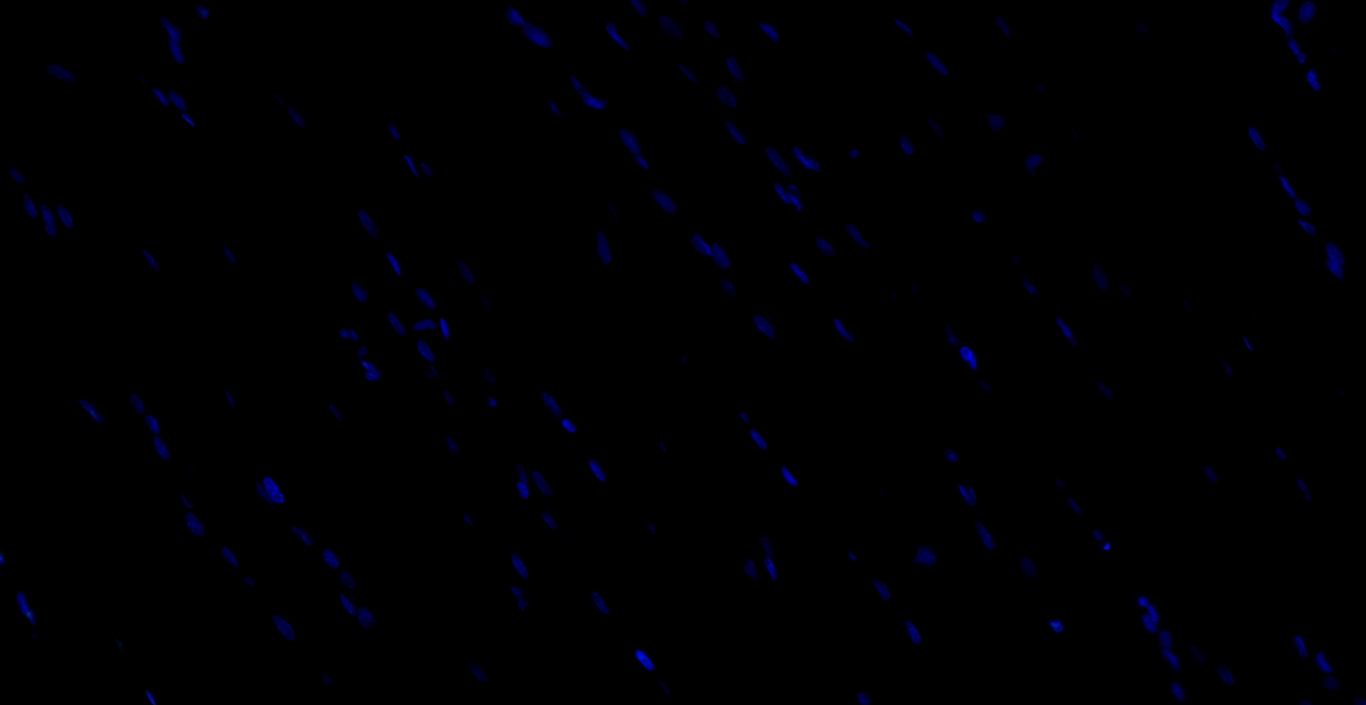

Supplement: Supplementary file 2 [file DataSheet4.ZIP › Supplemental materials 1/TUNEL/Sham/5-2 merge.jpg]

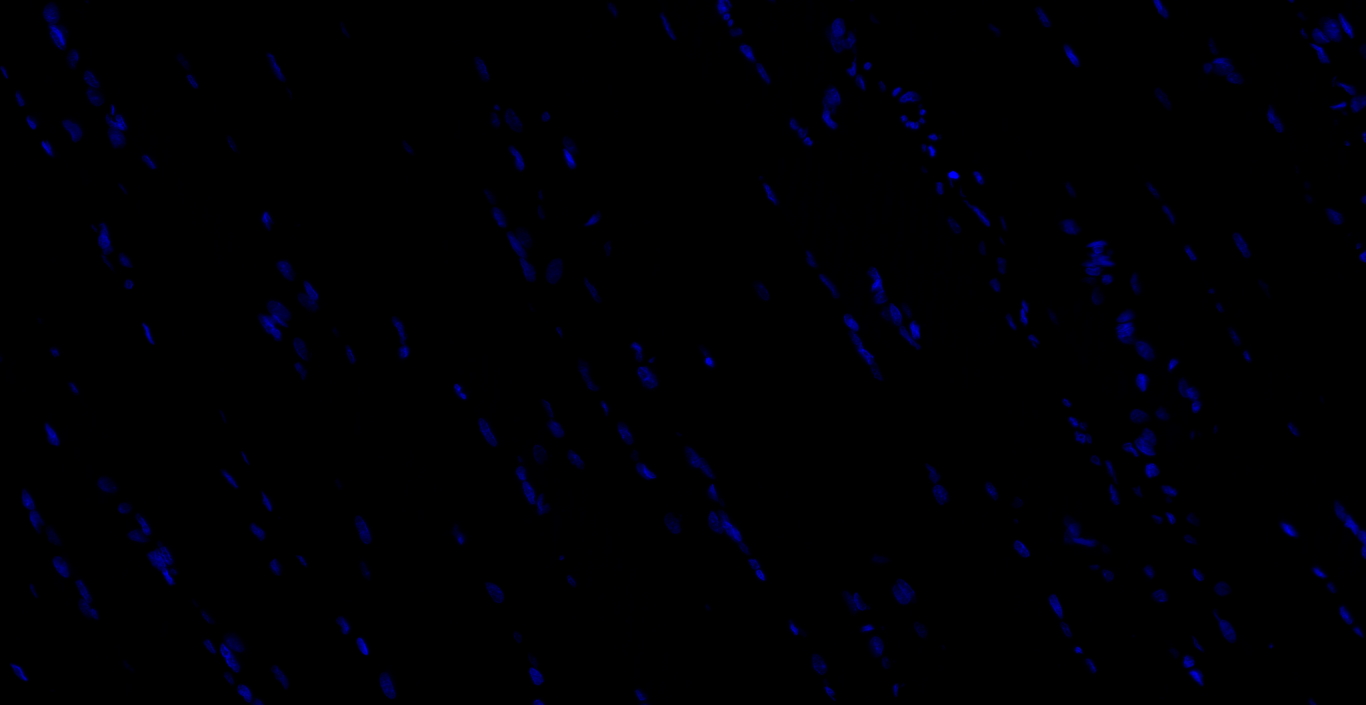

Supplement: Supplementary file 2 [file DataSheet4.ZIP › Supplemental materials 1/TUNEL/Sham/5-3 DAPI.jpg]

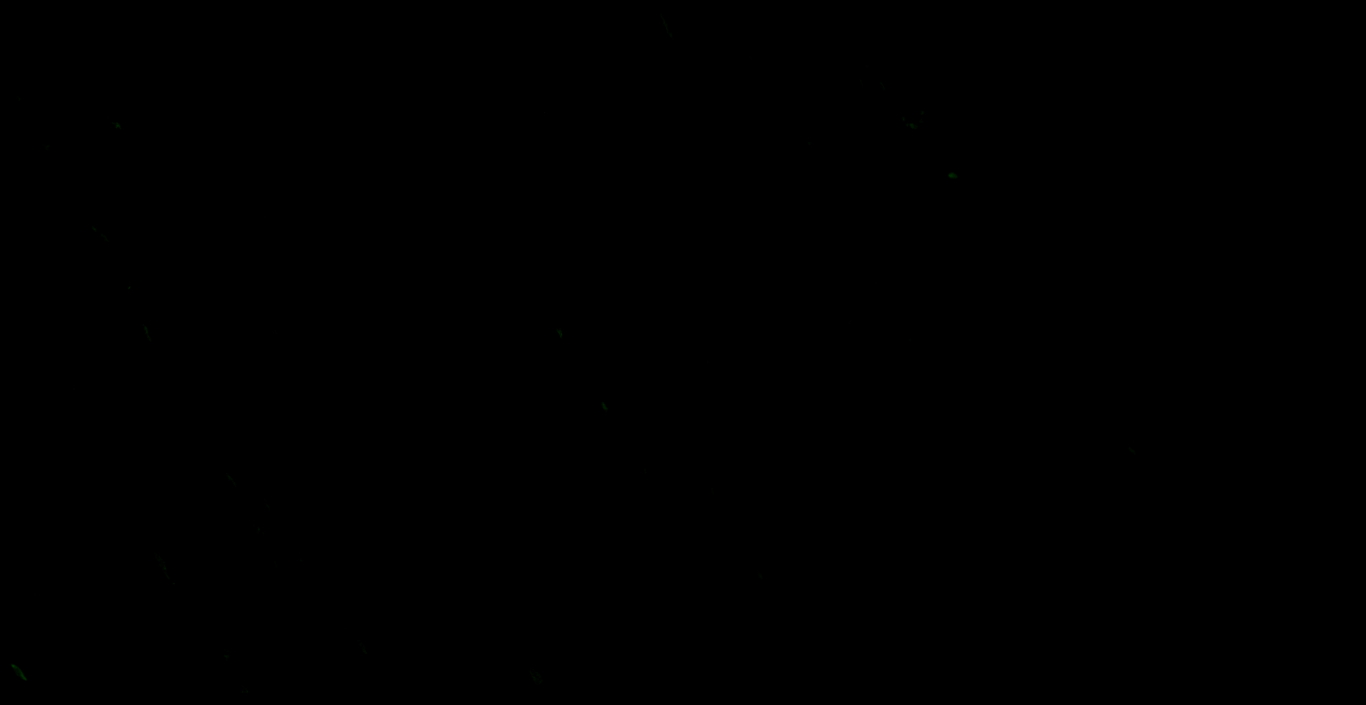

Supplement: Supplementary file 2 [file DataSheet4.ZIP › Supplemental materials 1/TUNEL/Sham/5-3 TUNEL.jpg]

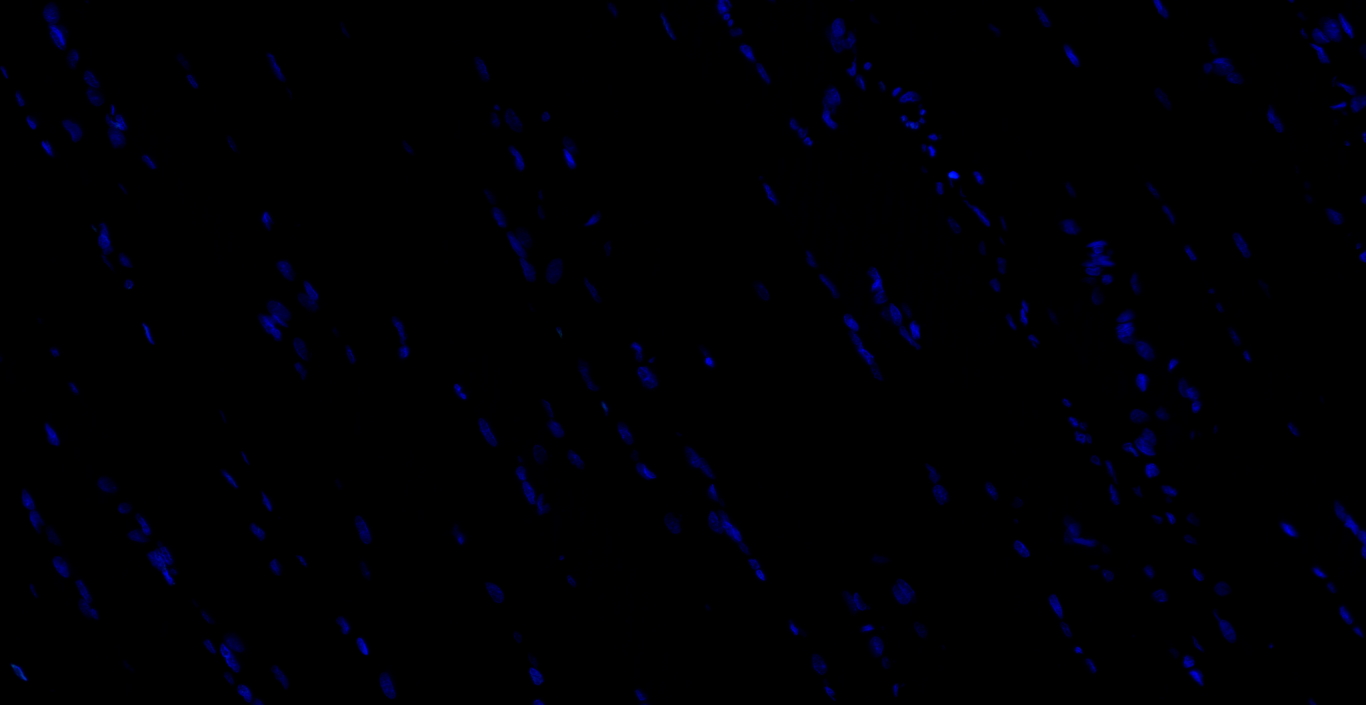

Supplement: Supplementary file 2 [file DataSheet4.ZIP › Supplemental materials 1/TUNEL/Sham/5-3 merge.jpg]

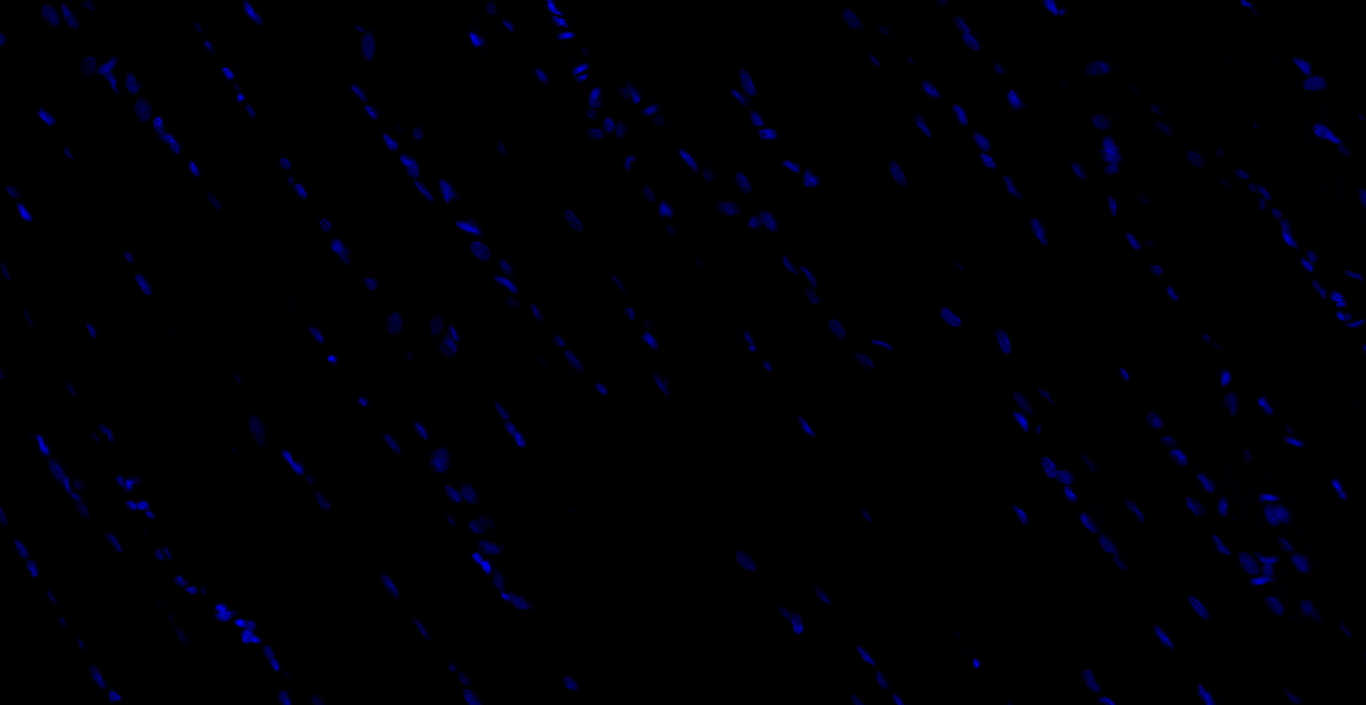

Supplement: Supplementary file 2 [file DataSheet4.ZIP › Supplemental materials 1/TUNEL/Sham/6-1 DAPI.jpg]

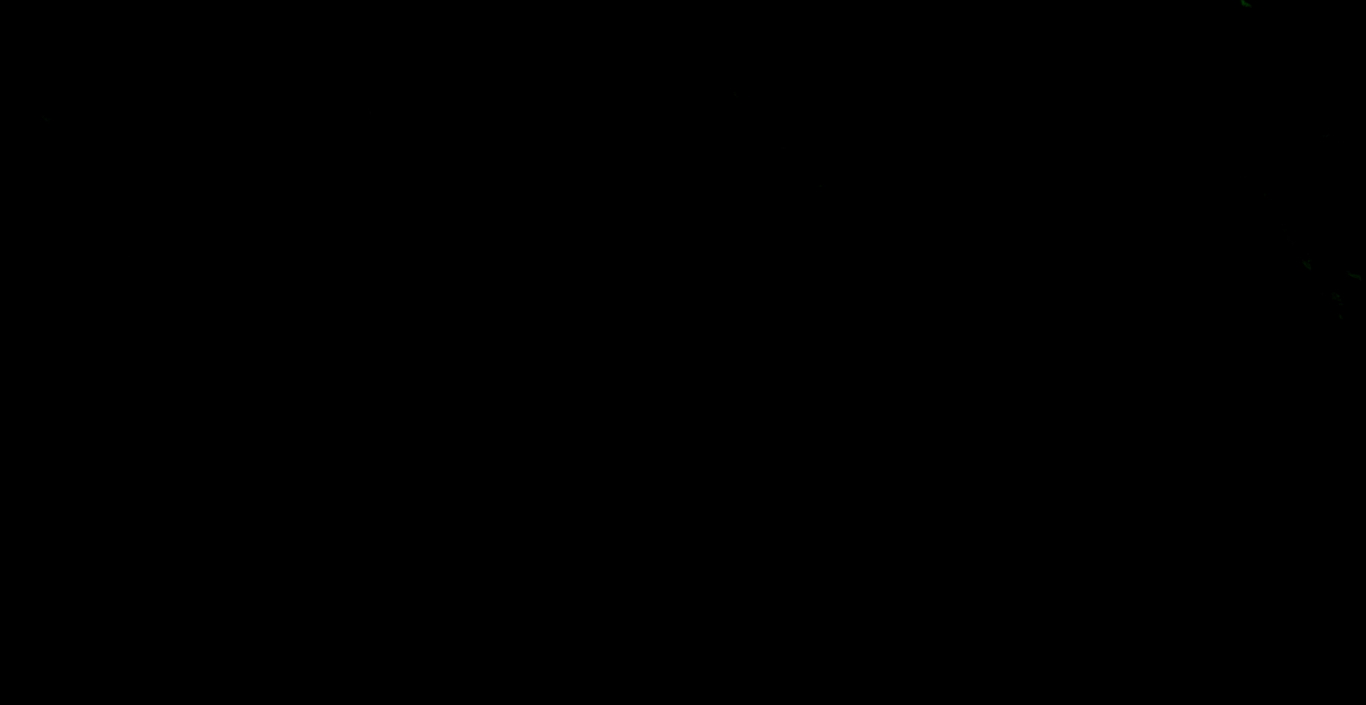

Supplement: Supplementary file 2 [file DataSheet4.ZIP › Supplemental materials 1/TUNEL/Sham/6-1 TUNEL.jpg]

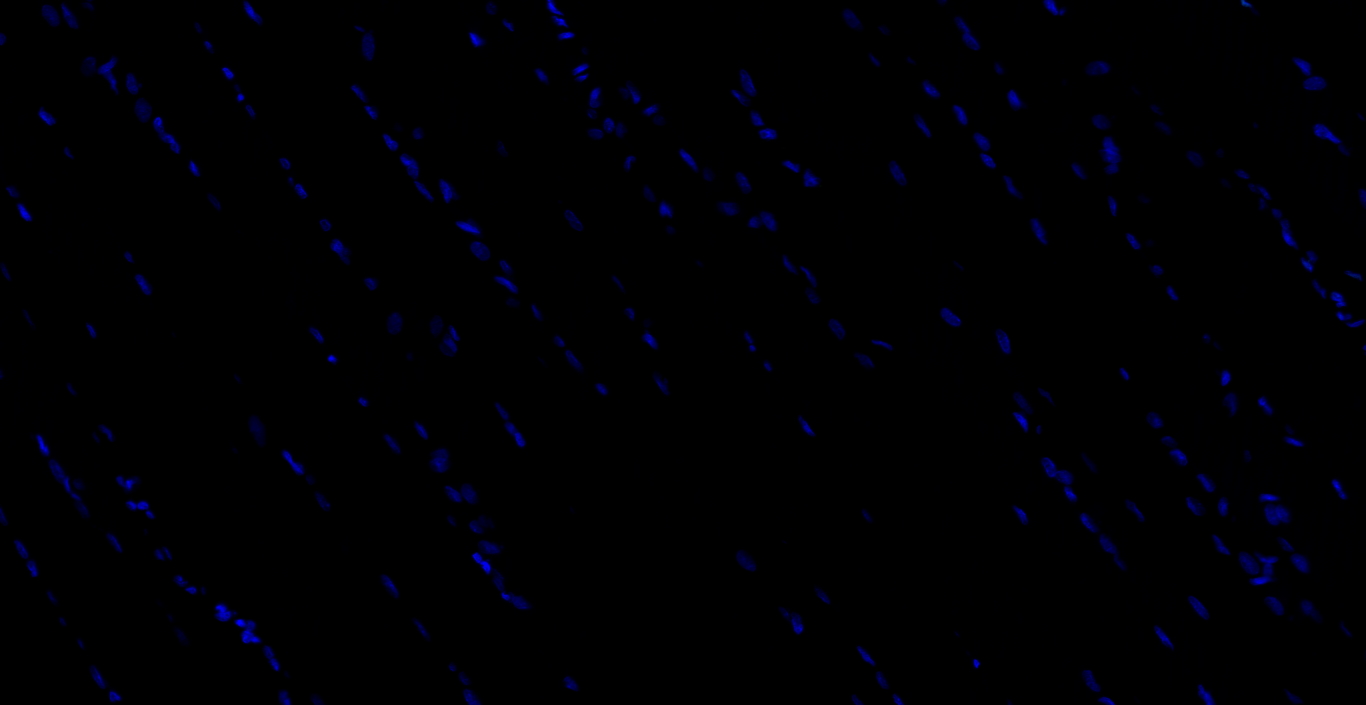

Supplement: Supplementary file 2 [file DataSheet4.ZIP › Supplemental materials 1/TUNEL/Sham/6-1 merge.jpg]

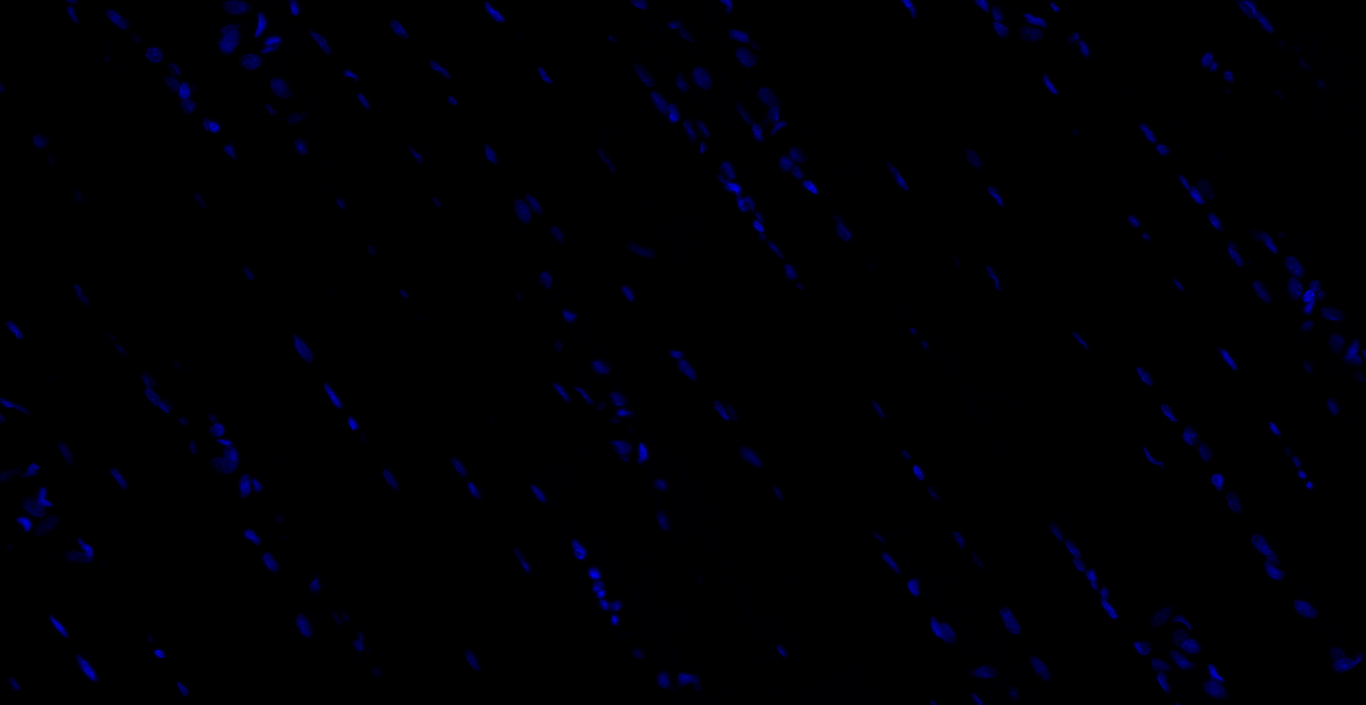

Supplement: Supplementary file 2 [file DataSheet4.ZIP › Supplemental materials 1/TUNEL/Sham/6-2 DAPI.jpg]

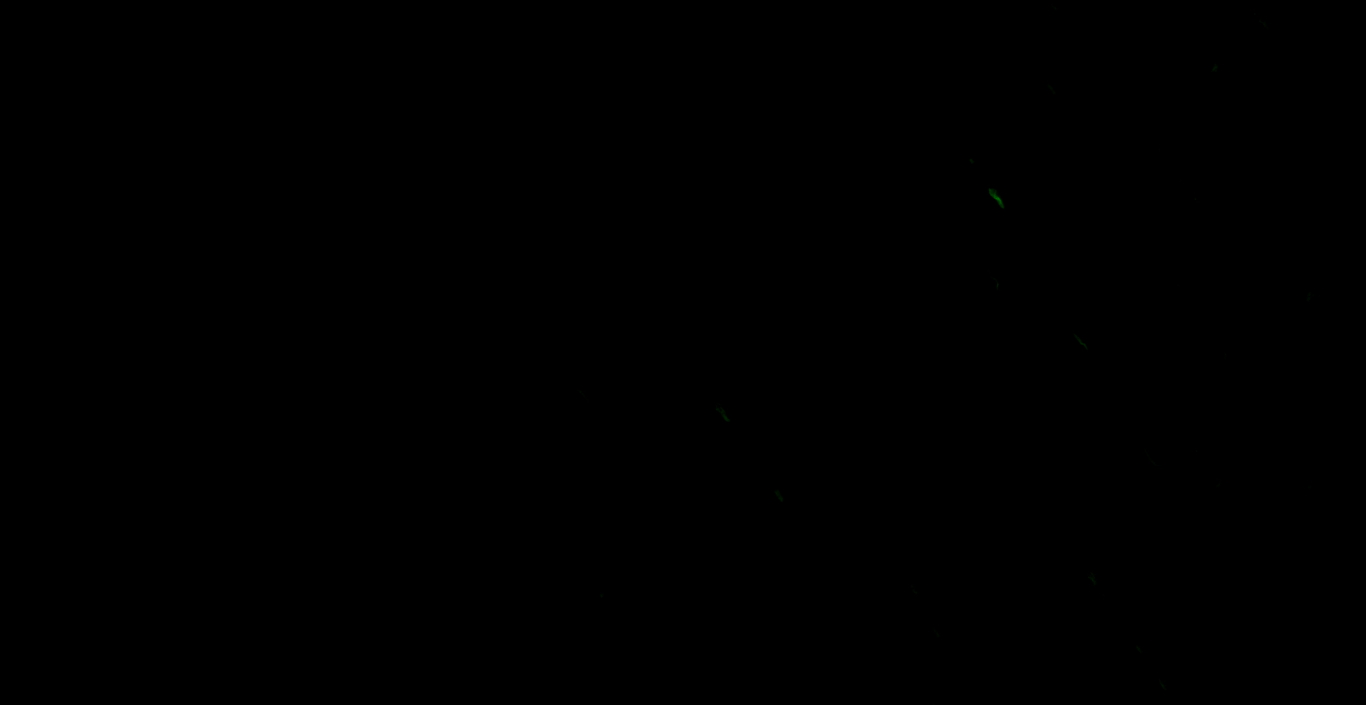

Supplement: Supplementary file 2 [file DataSheet4.ZIP › Supplemental materials 1/TUNEL/Sham/6-2 TUNEL.jpg]

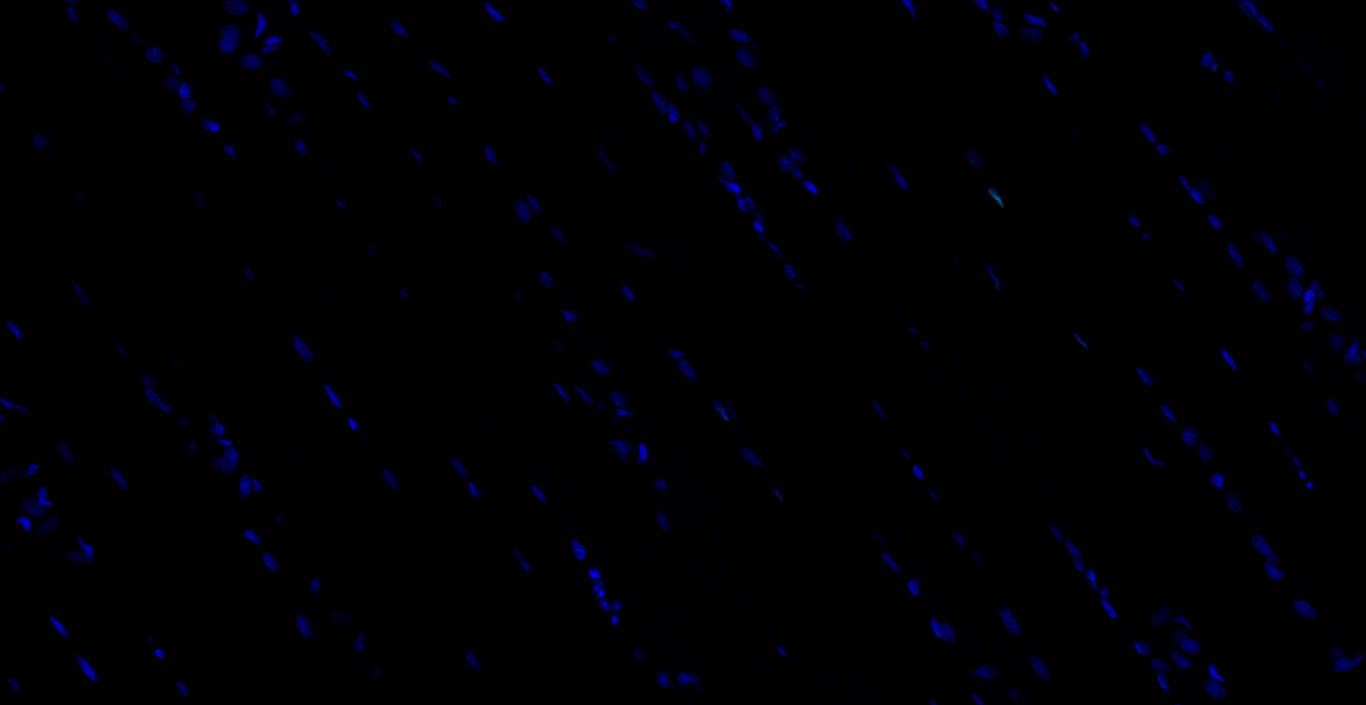

Supplement: Supplementary file 2 [file DataSheet4.ZIP › Supplemental materials 1/TUNEL/Sham/6-2 merge.jpg]

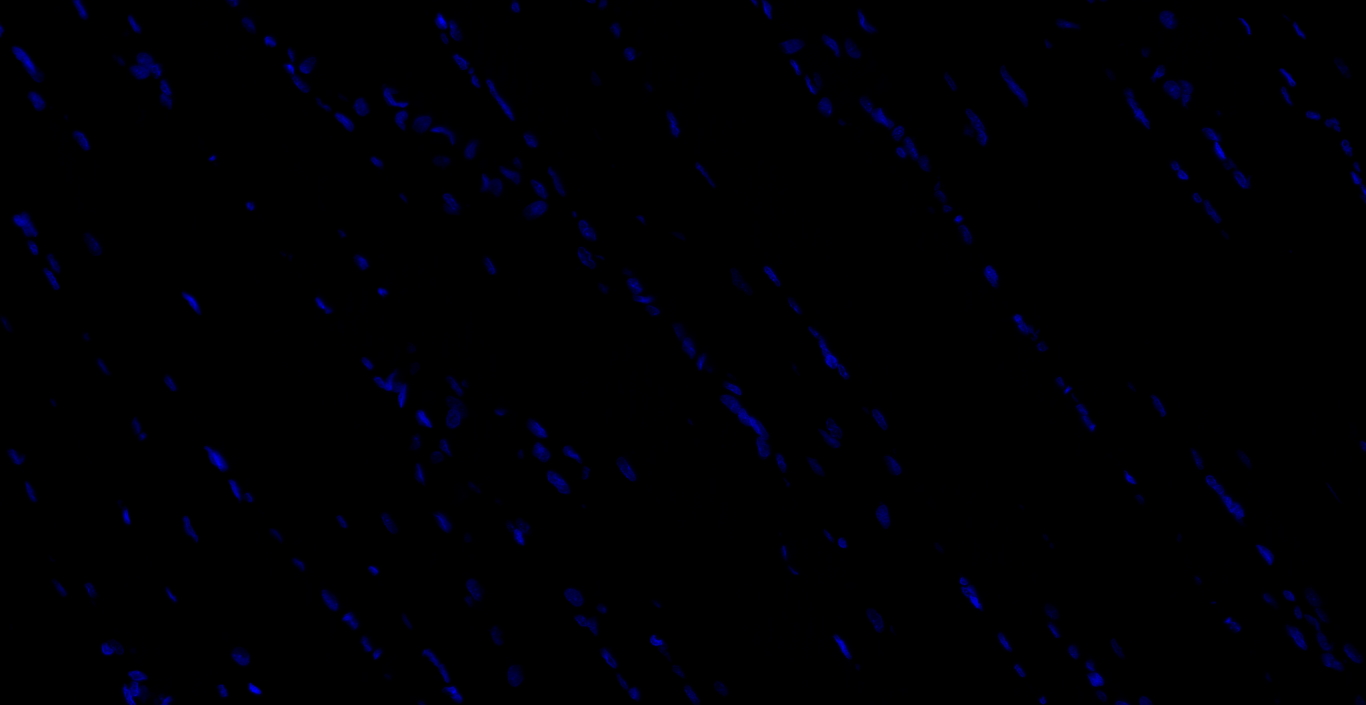

Supplement: Supplementary file 2 [file DataSheet4.ZIP › Supplemental materials 1/TUNEL/Sham/6-3 DAPI.jpg]

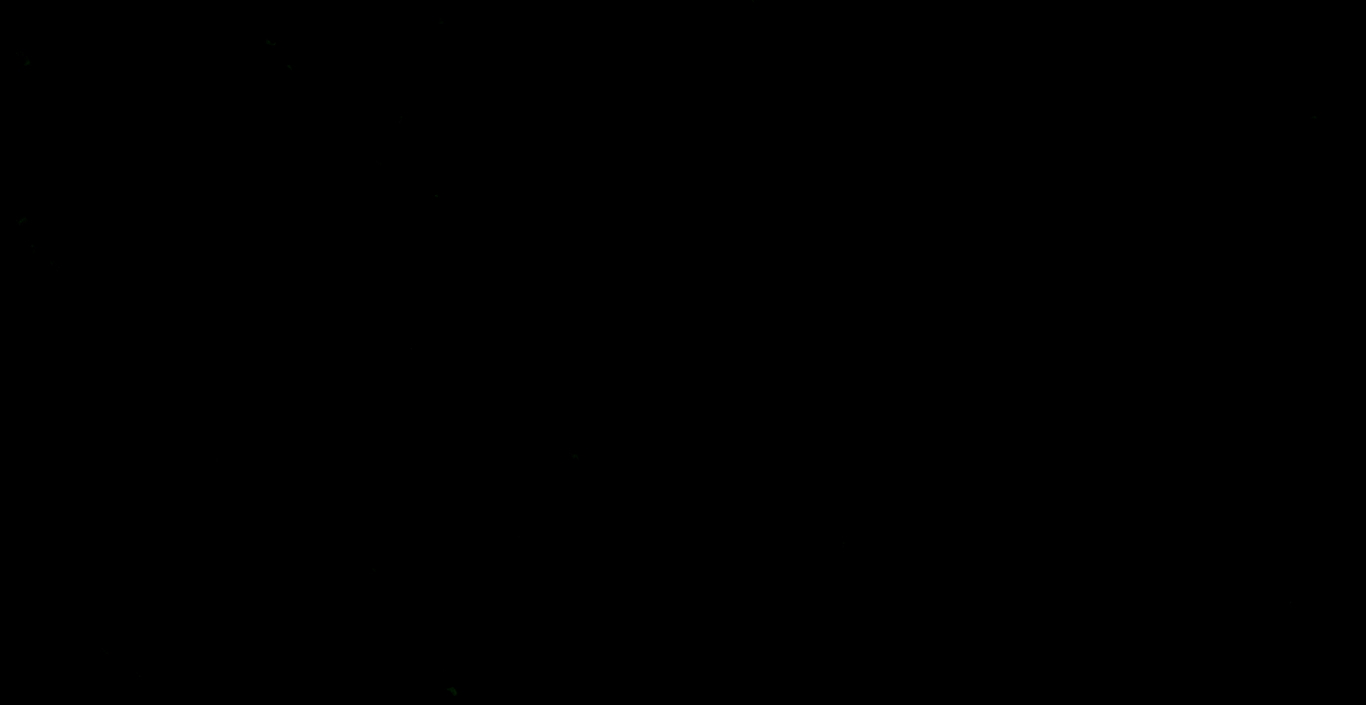

Supplement: Supplementary file 2 [file DataSheet4.ZIP › Supplemental materials 1/TUNEL/Sham/6-3 TUNEL.jpg]

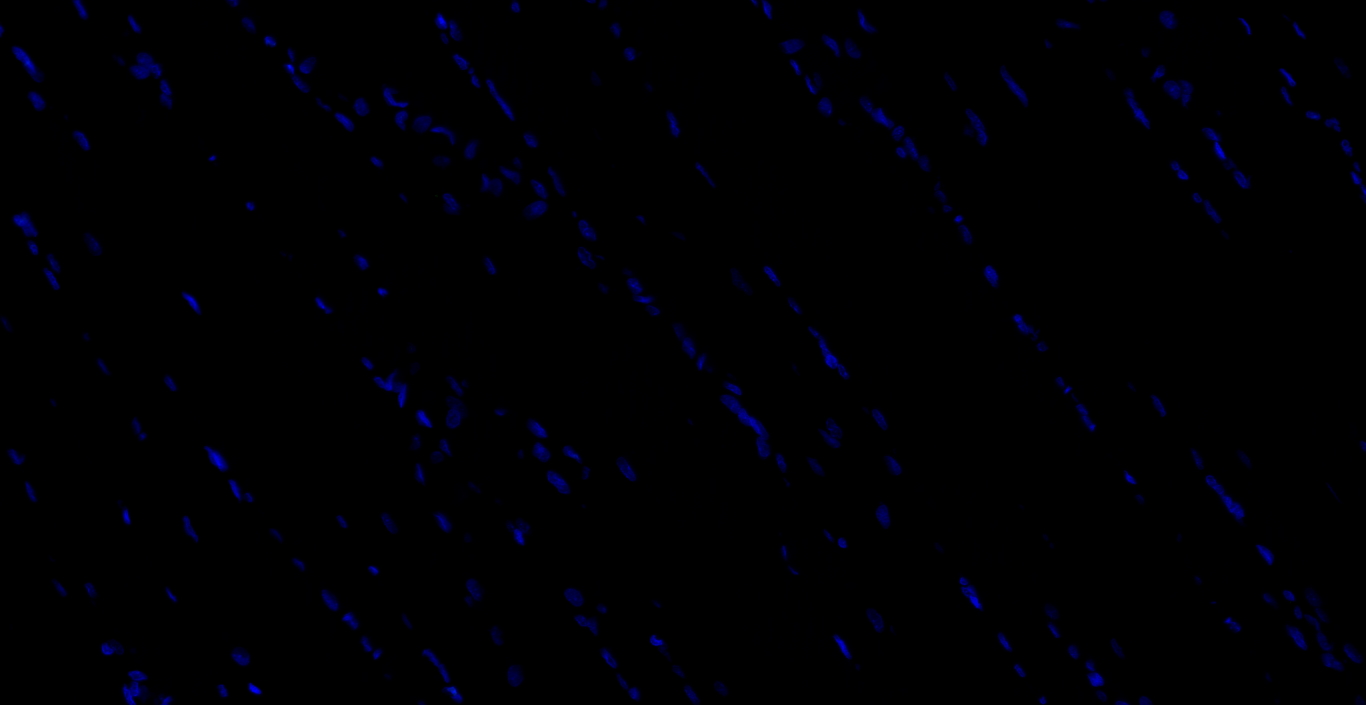

Supplement: Supplementary file 2 [file DataSheet4.ZIP › Supplemental materials 1/TUNEL/Sham/6-3 merge.jpg]

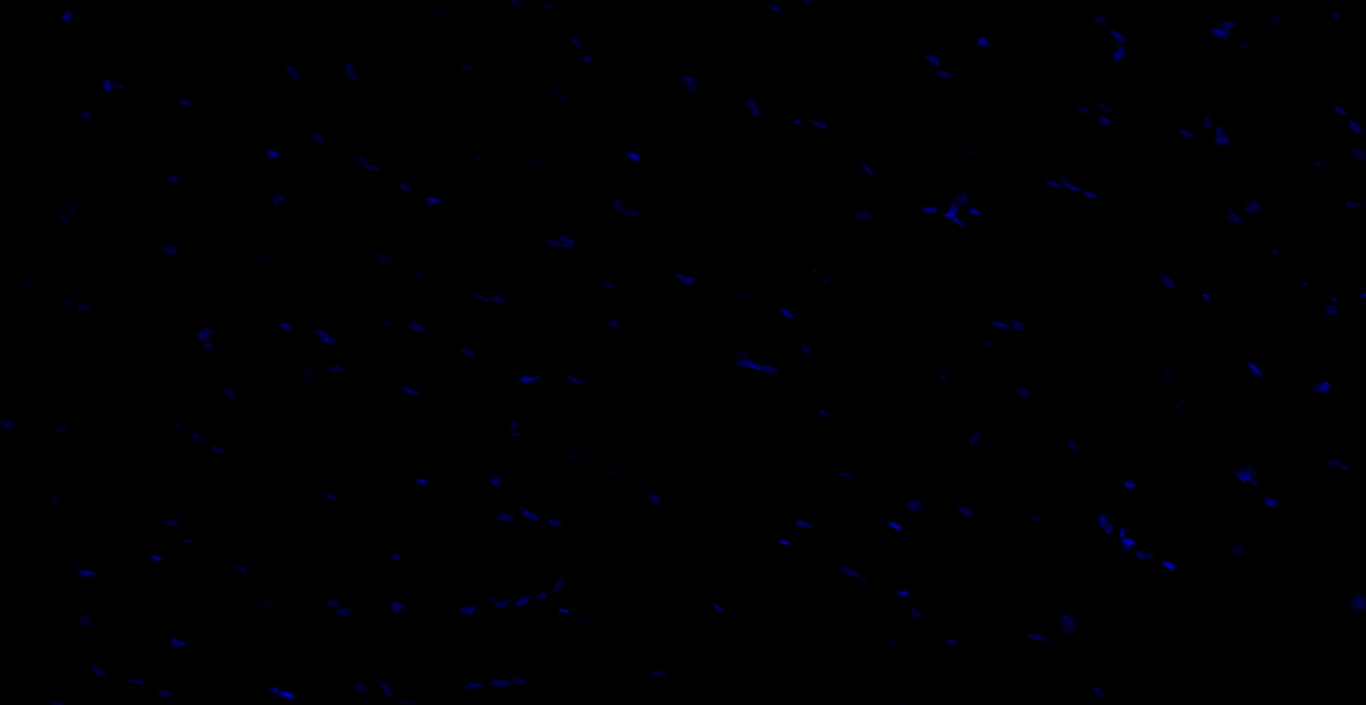

Supplement: Supplementary file 2 [file DataSheet4.ZIP › Supplemental materials 1/TUNEL/VNS/1-1 DAPI.jpg]

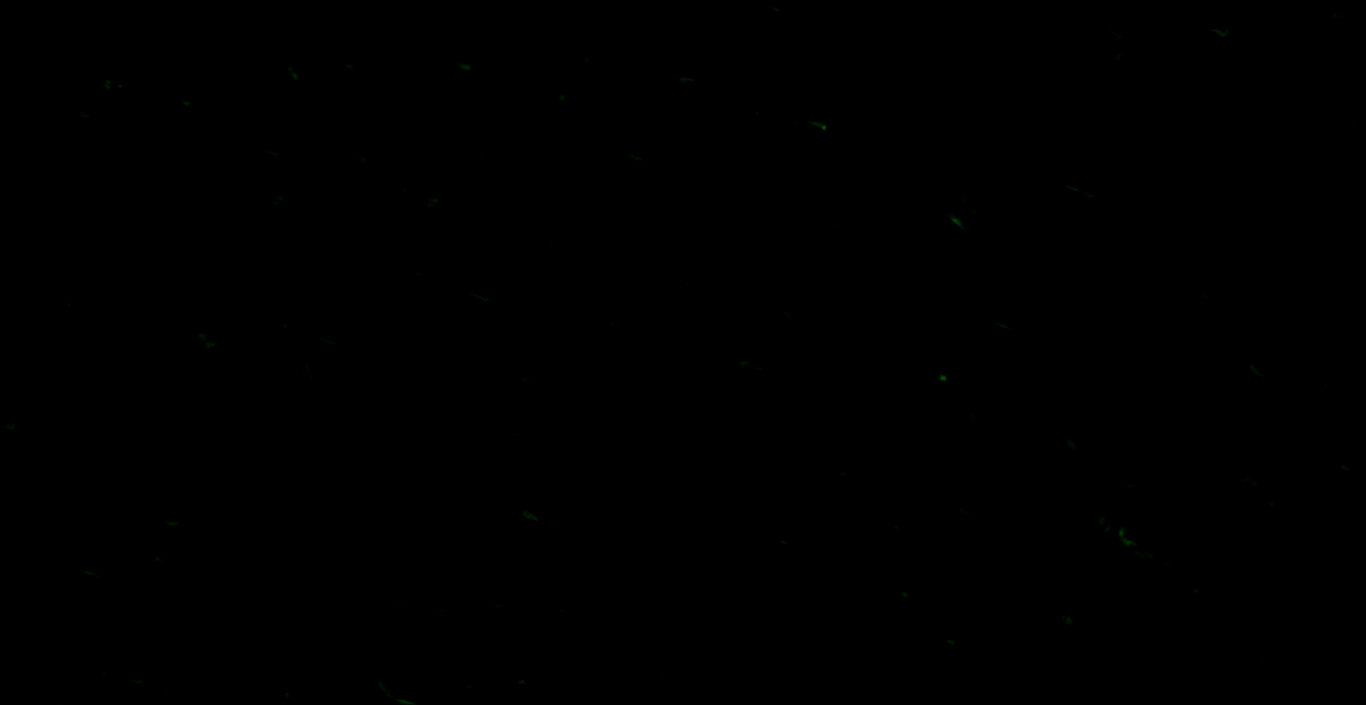

Supplement: Supplementary file 2 [file DataSheet4.ZIP › Supplemental materials 1/TUNEL/VNS/1-1 TUNEL.jpg]

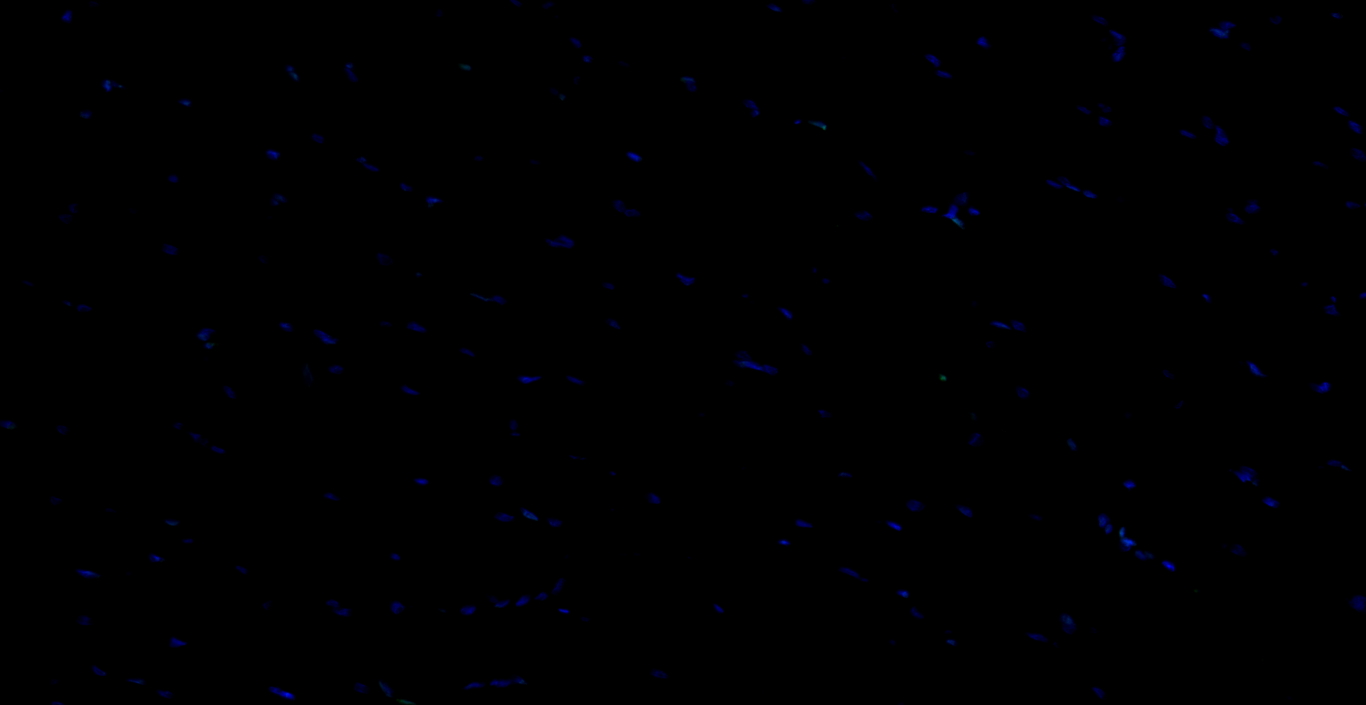

Supplement: Supplementary file 2 [file DataSheet4.ZIP › Supplemental materials 1/TUNEL/VNS/1-1 merge.jpg]

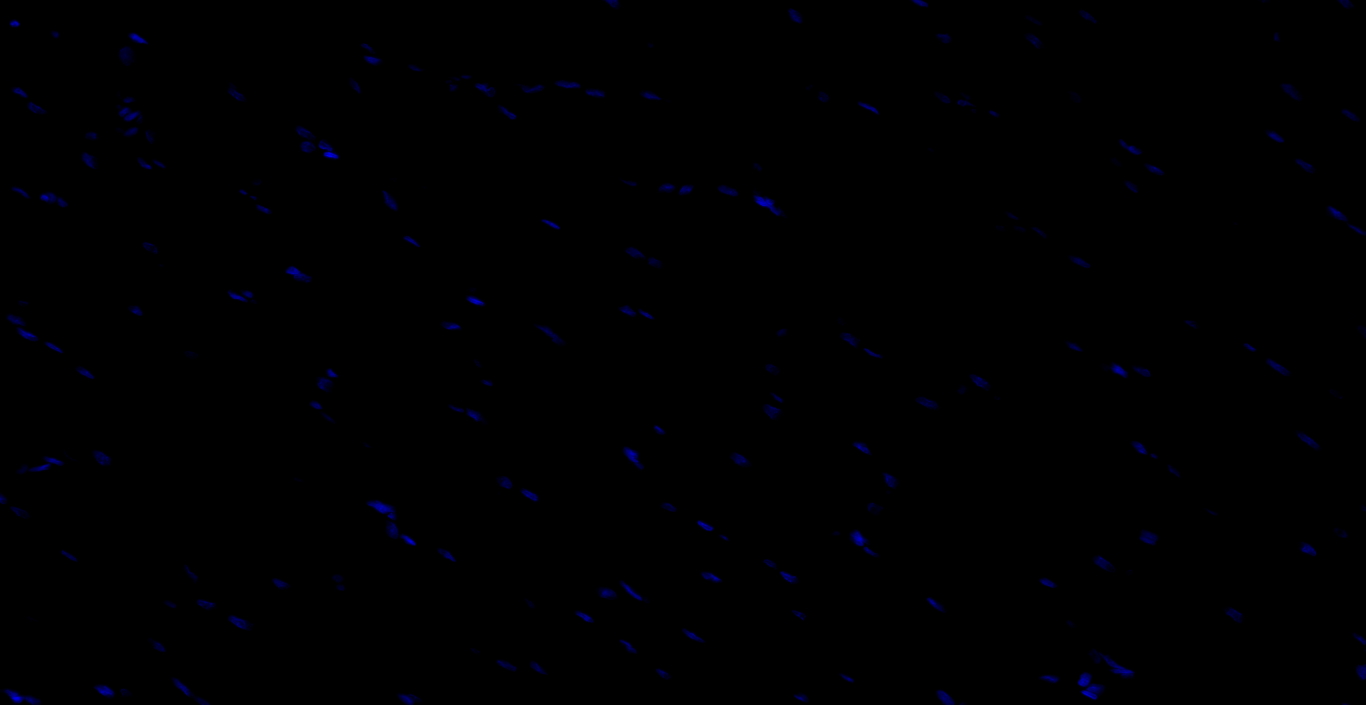

Supplement: Supplementary file 2 [file DataSheet4.ZIP › Supplemental materials 1/TUNEL/VNS/1-2 DAPI.jpg]

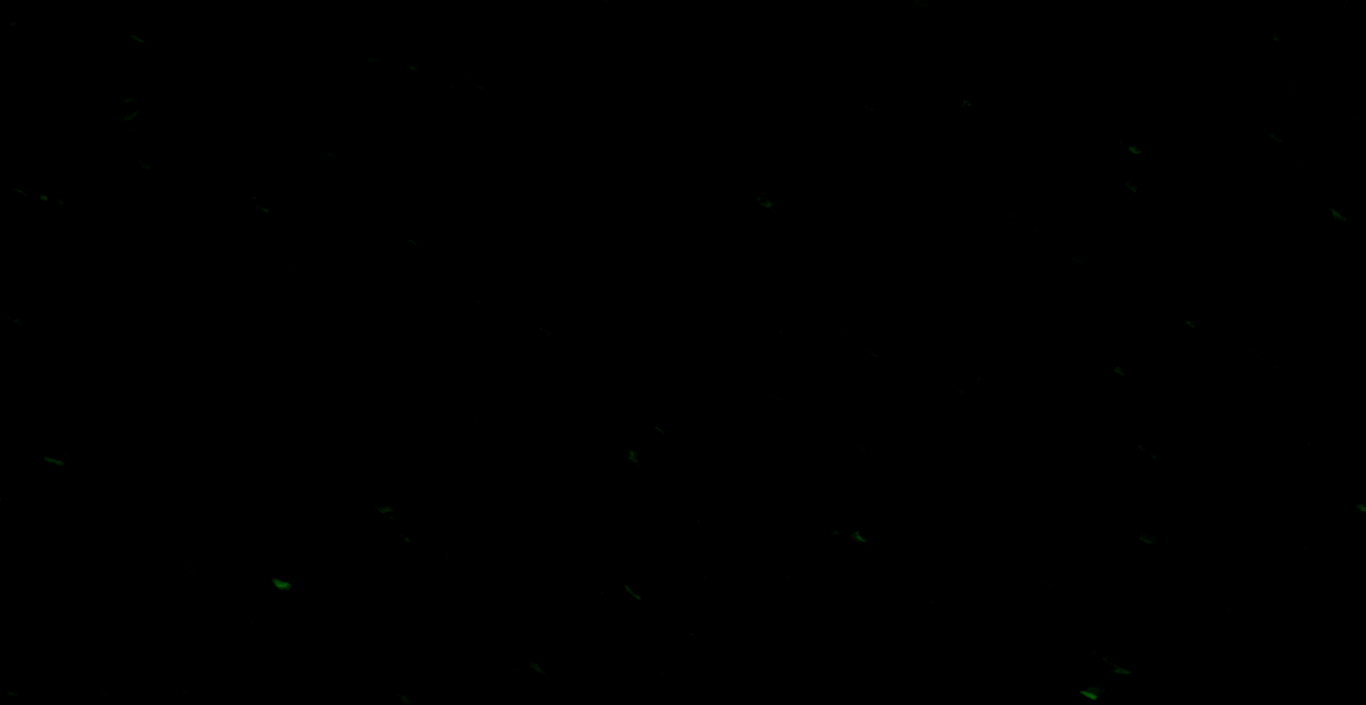

Supplement: Supplementary file 2 [file DataSheet4.ZIP › Supplemental materials 1/TUNEL/VNS/1-2 TUNEL.jpg]

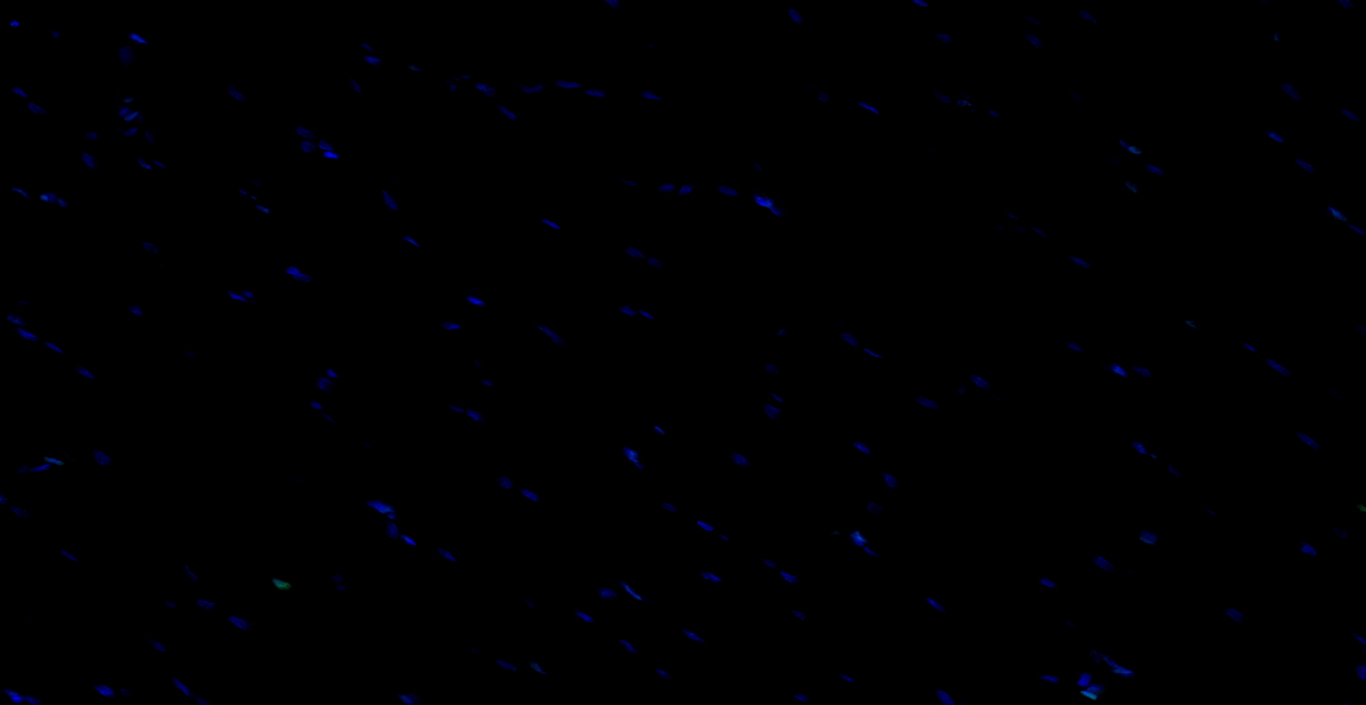

Supplement: Supplementary file 2 [file DataSheet4.ZIP › Supplemental materials 1/TUNEL/VNS/1-2 merge.jpg]

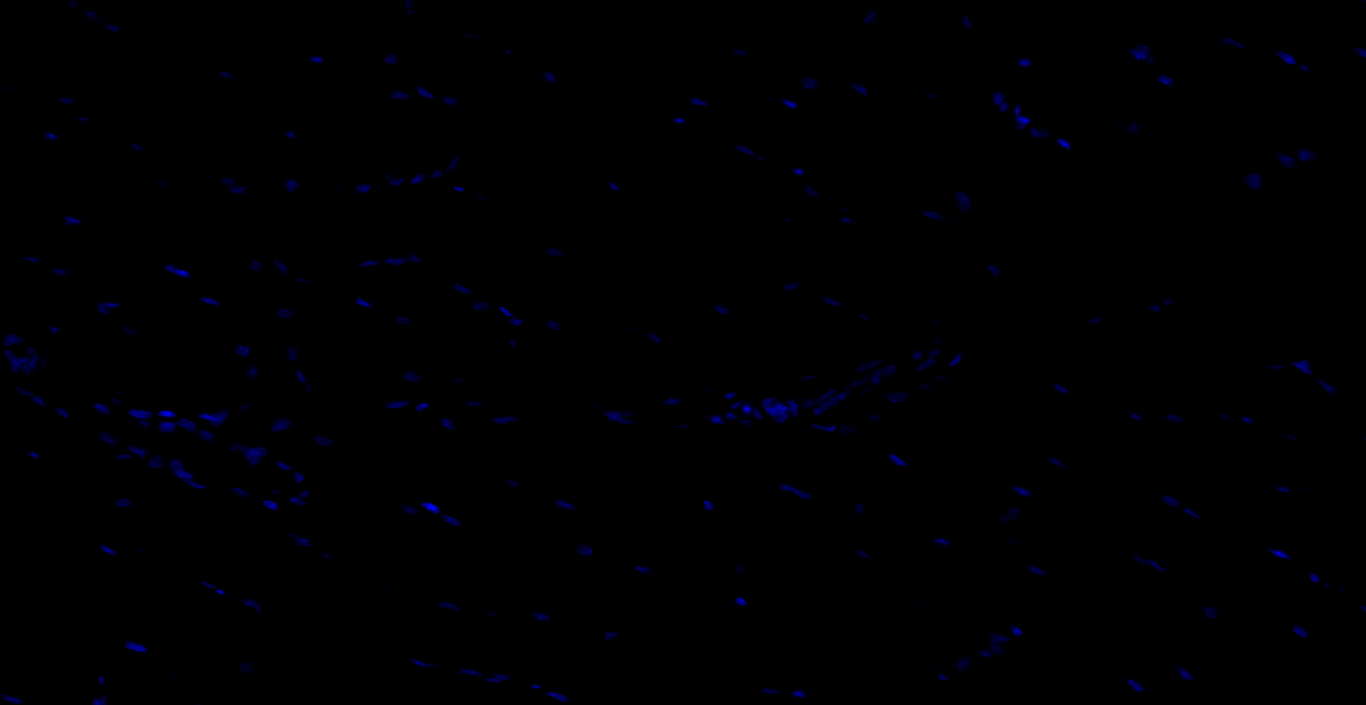

Supplement: Supplementary file 2 [file DataSheet4.ZIP › Supplemental materials 1/TUNEL/VNS/1-3 DAPI.jpg]

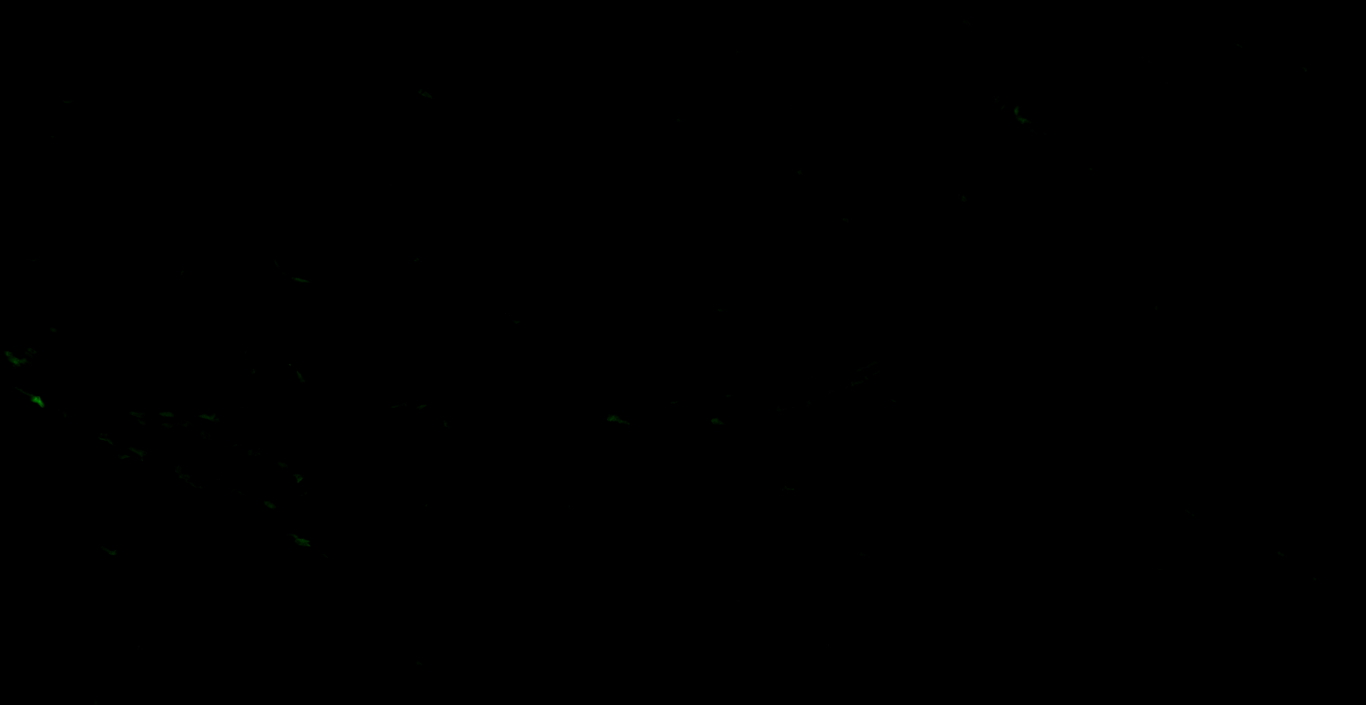

Supplement: Supplementary file 2 [file DataSheet4.ZIP › Supplemental materials 1/TUNEL/VNS/1-3 TUNEL.jpg]

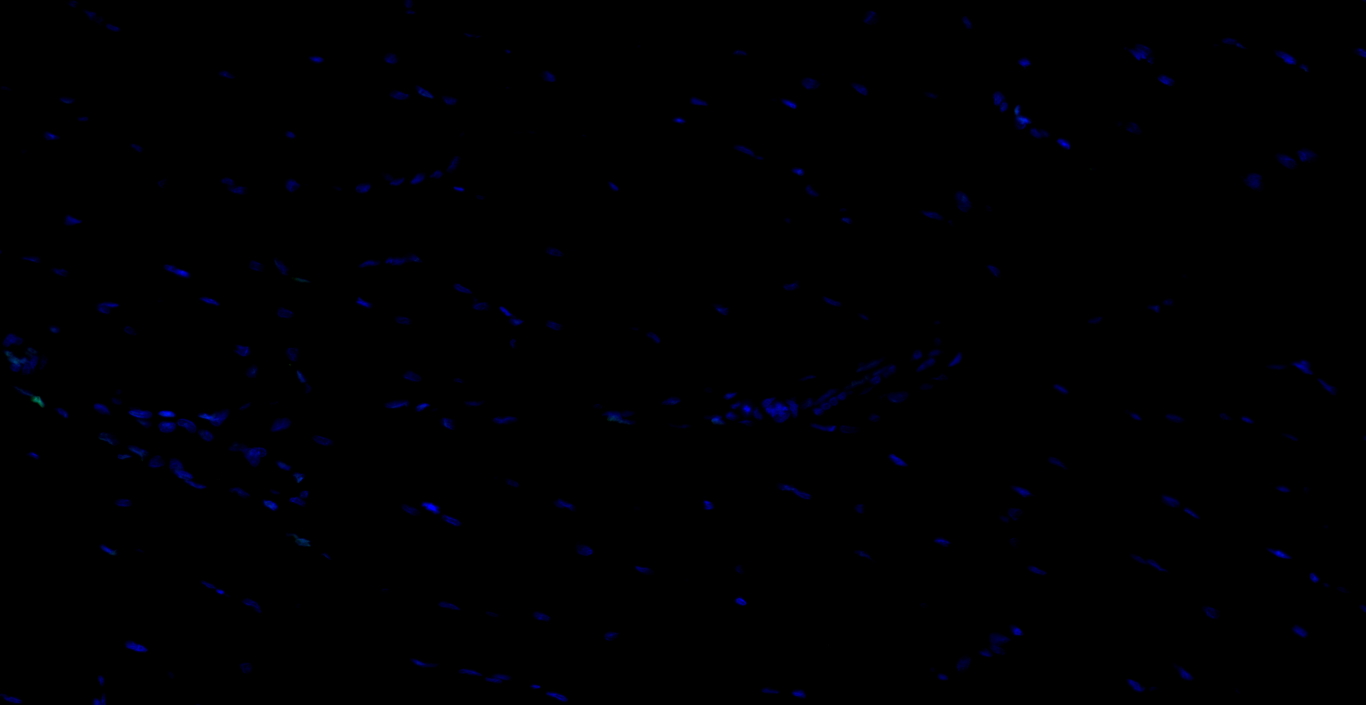

Supplement: Supplementary file 2 [file DataSheet4.ZIP › Supplemental materials 1/TUNEL/VNS/1-3 merge.jpg]

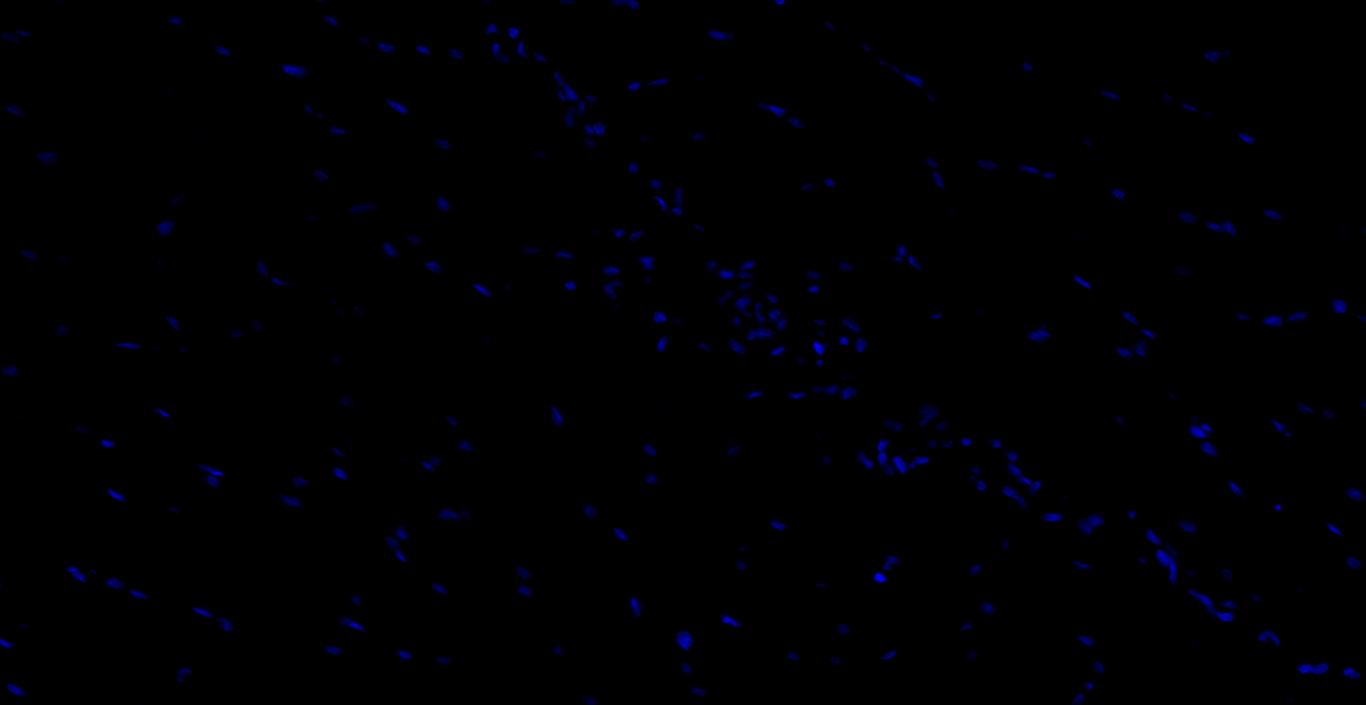

Supplement: Supplementary file 2 [file DataSheet4.ZIP › Supplemental materials 1/TUNEL/VNS/2-1 DAPI.jpg]

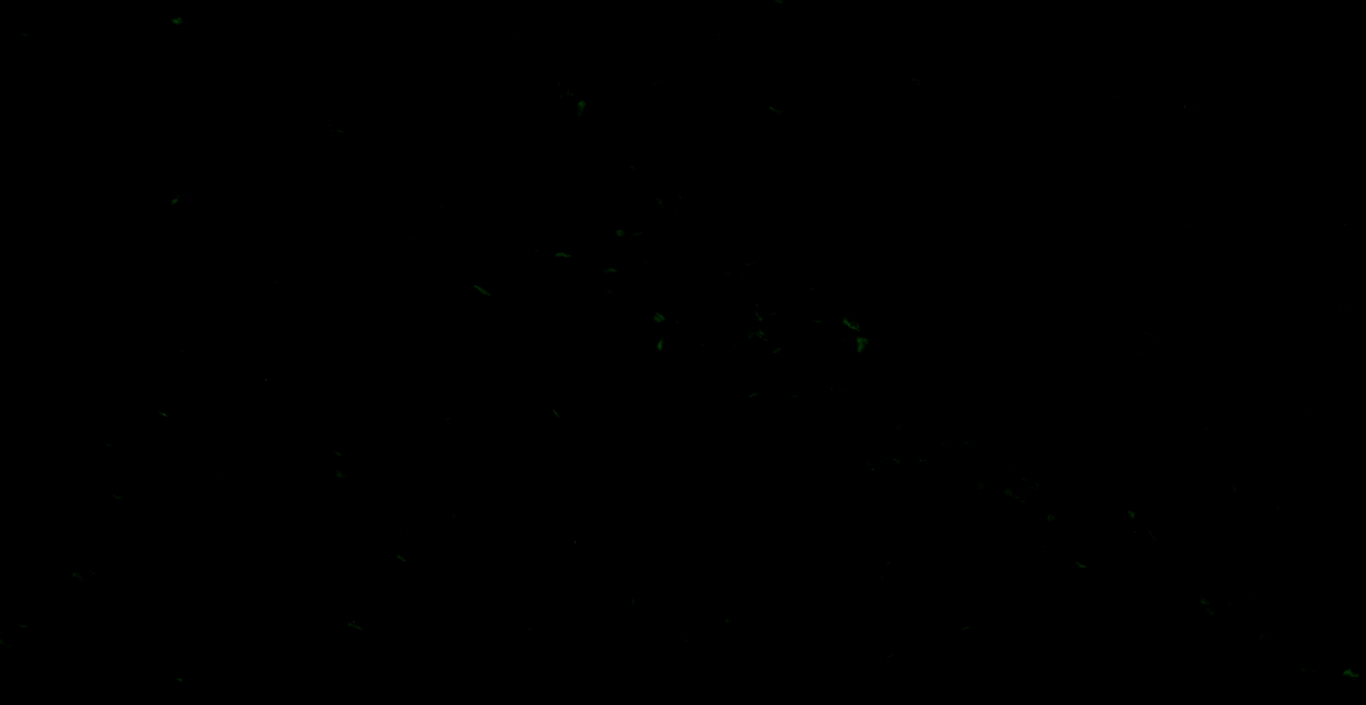

Supplement: Supplementary file 2 [file DataSheet4.ZIP › Supplemental materials 1/TUNEL/VNS/2-1 TUNEL.jpg]

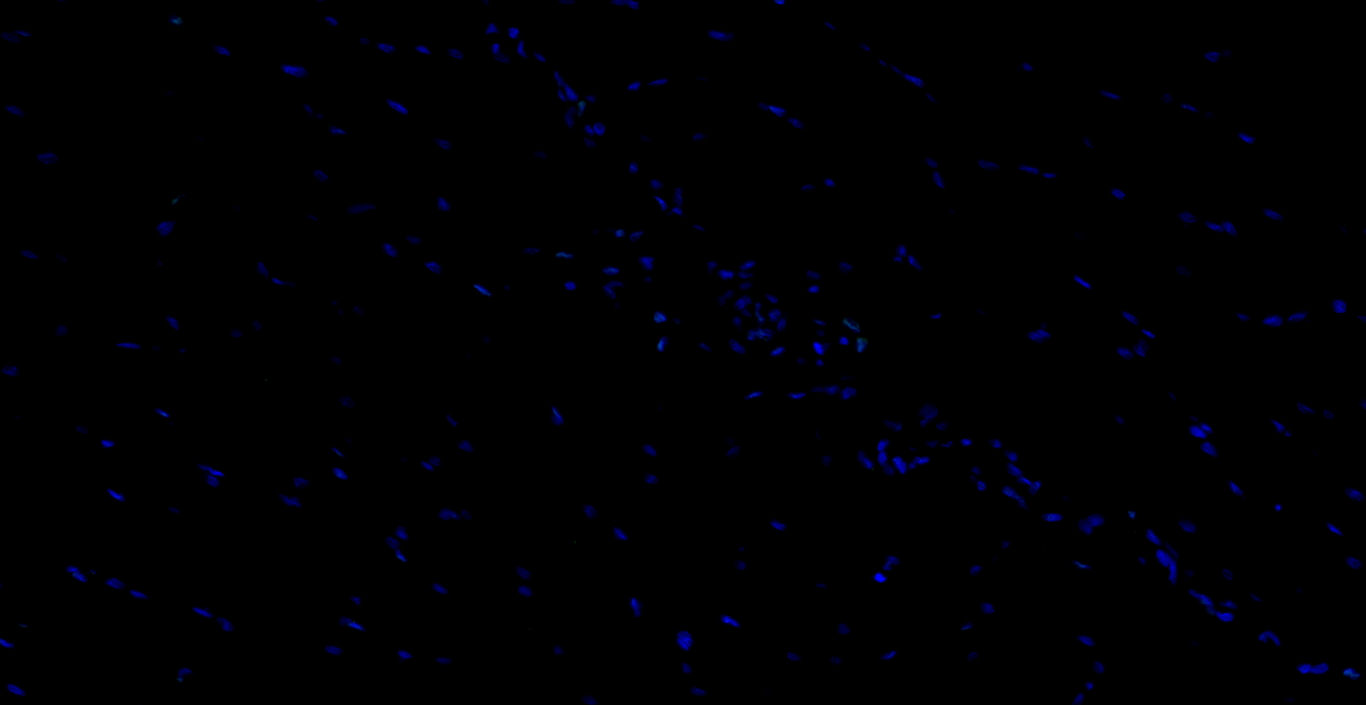

Supplement: Supplementary file 2 [file DataSheet4.ZIP › Supplemental materials 1/TUNEL/VNS/2-1 merge.jpg]

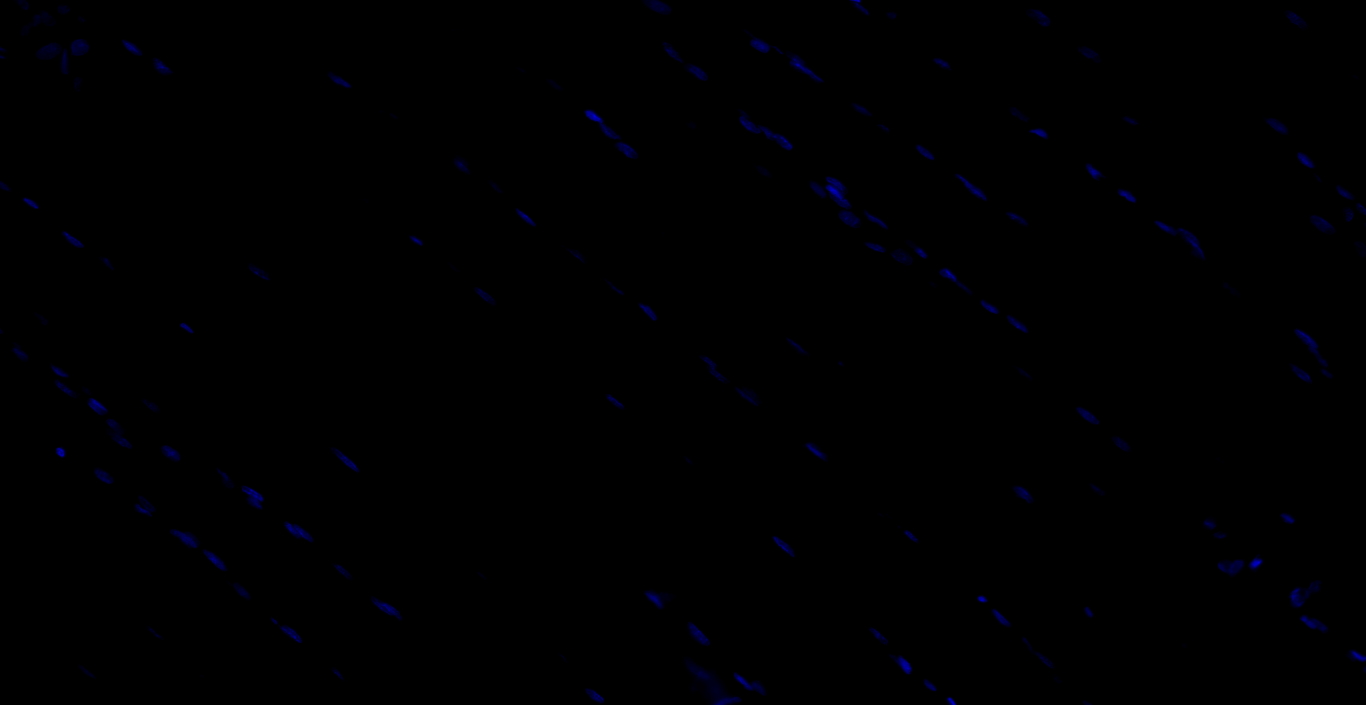

Supplement: Supplementary file 2 [file DataSheet4.ZIP › Supplemental materials 1/TUNEL/VNS/2-2 DAPI.jpg]

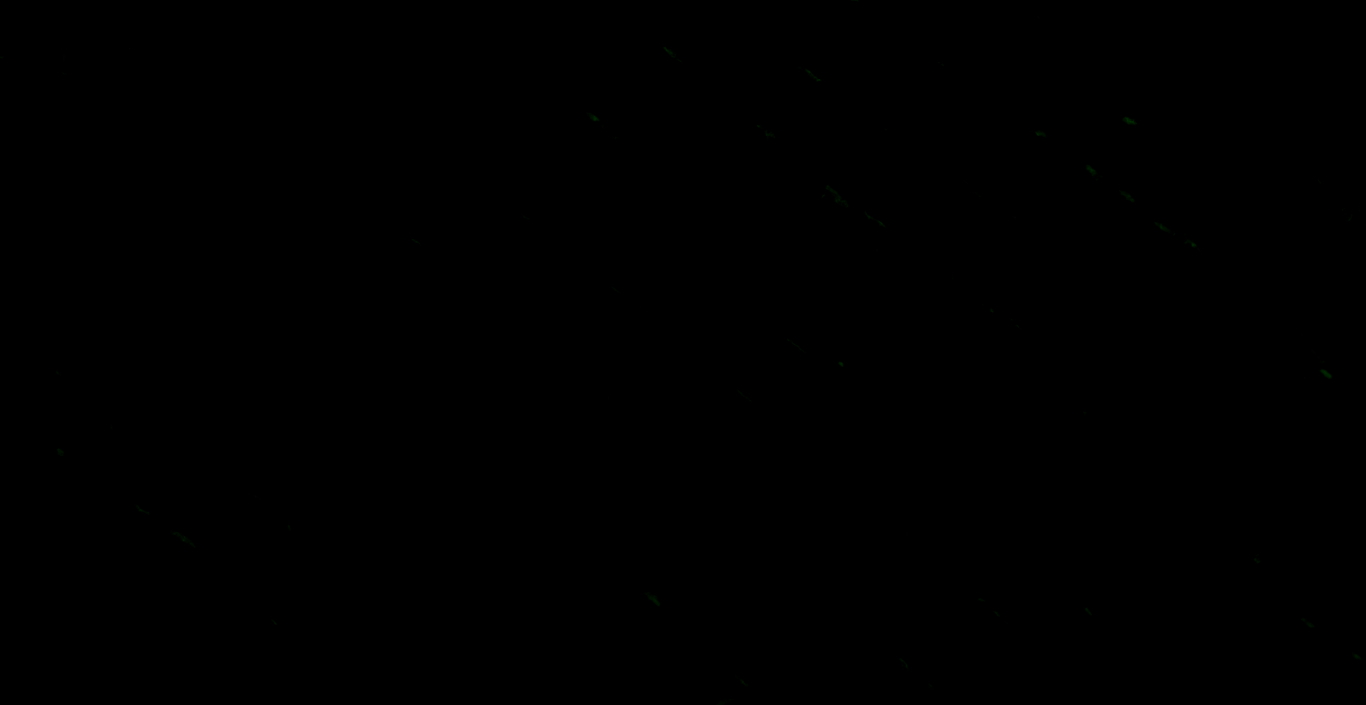

Supplement: Supplementary file 2 [file DataSheet4.ZIP › Supplemental materials 1/TUNEL/VNS/2-2 TUNEL.jpg]

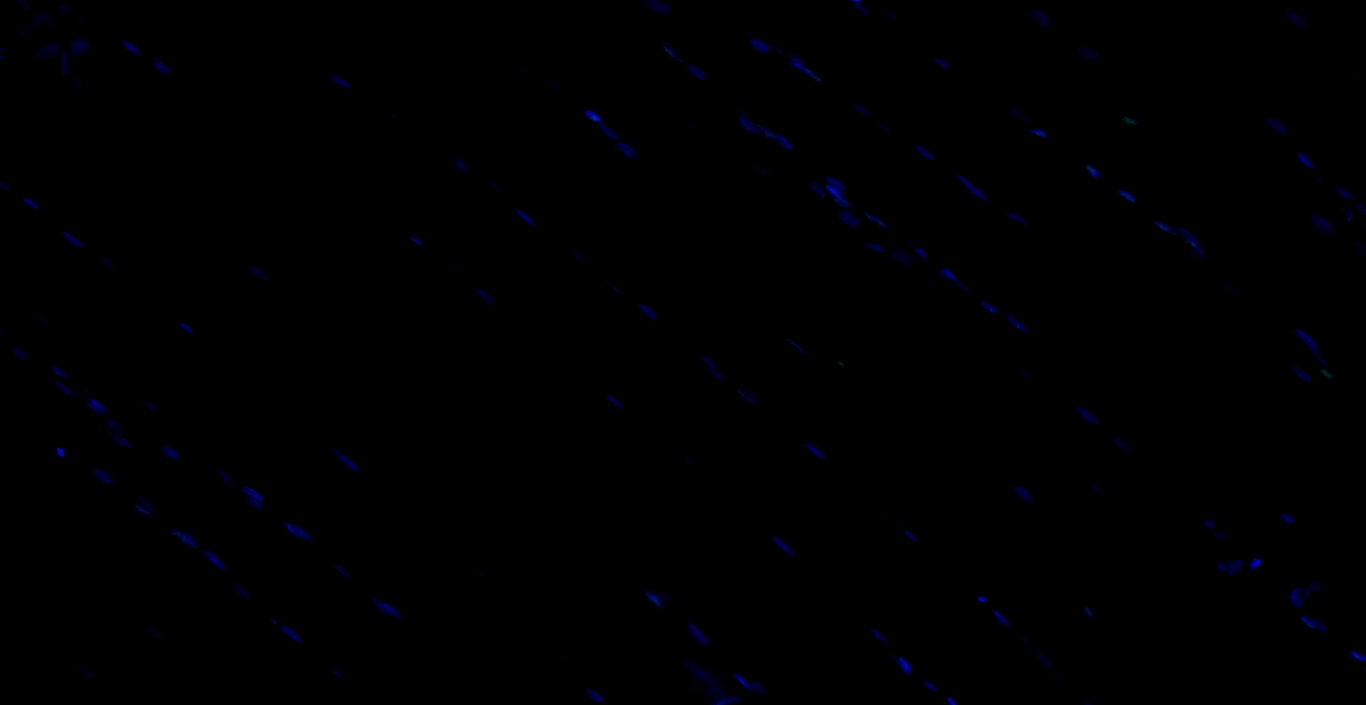

Supplement: Supplementary file 2 [file DataSheet4.ZIP › Supplemental materials 1/TUNEL/VNS/2-2 merge.jpg]

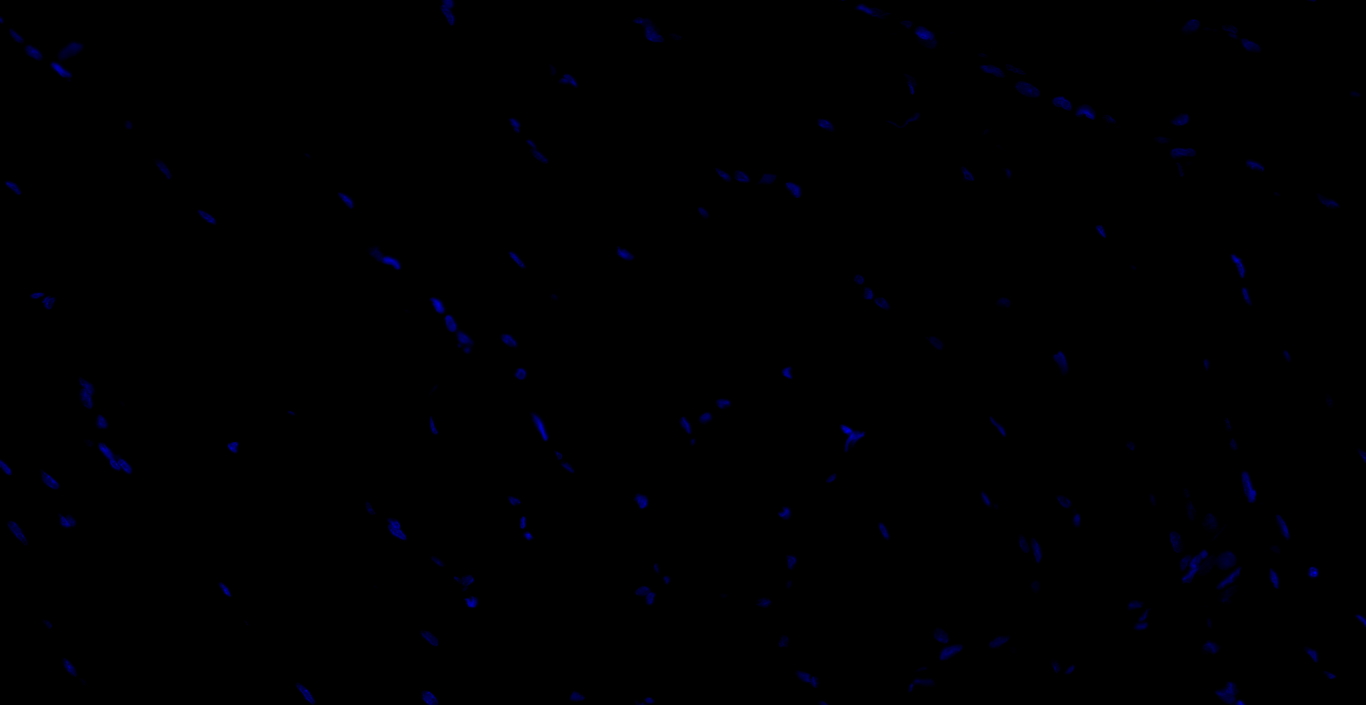

Supplement: Supplementary file 2 [file DataSheet4.ZIP › Supplemental materials 1/TUNEL/VNS/2-3 DAPI.jpg]

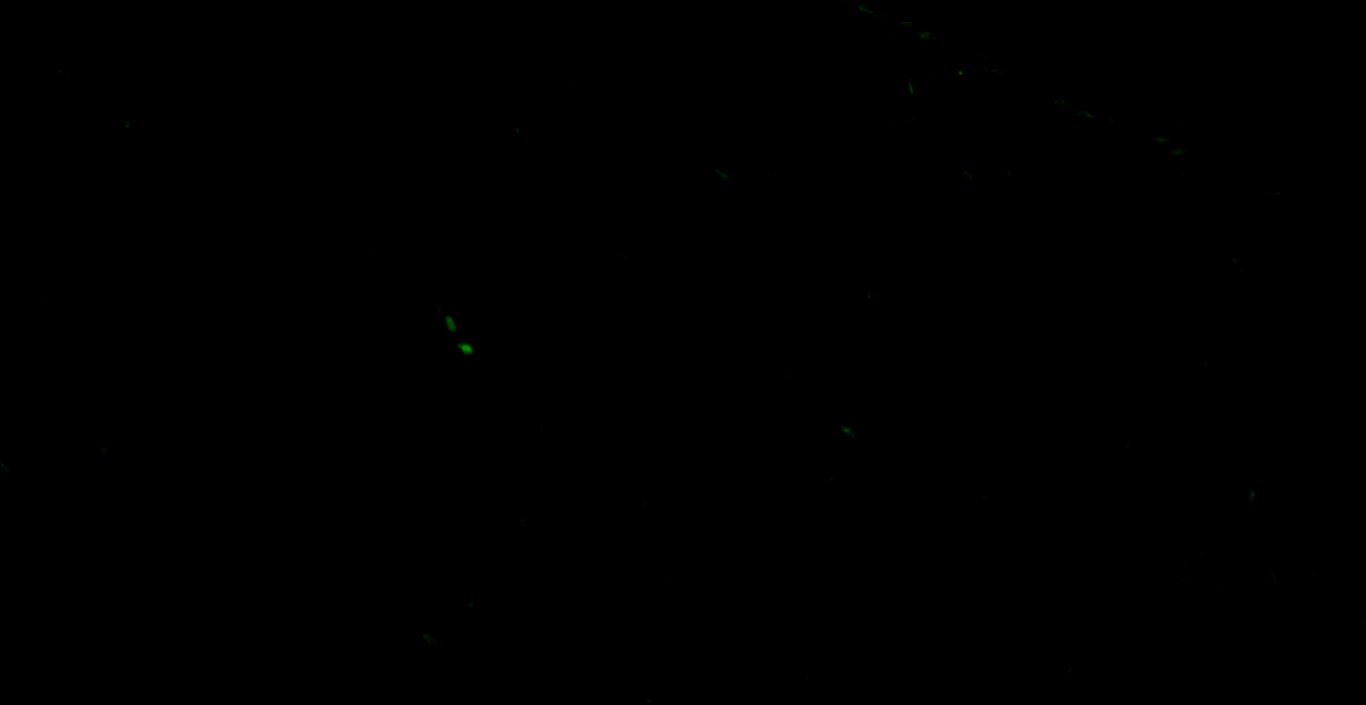

Supplement: Supplementary file 2 [file DataSheet4.ZIP › Supplemental materials 1/TUNEL/VNS/2-3 TUNEL.jpg]

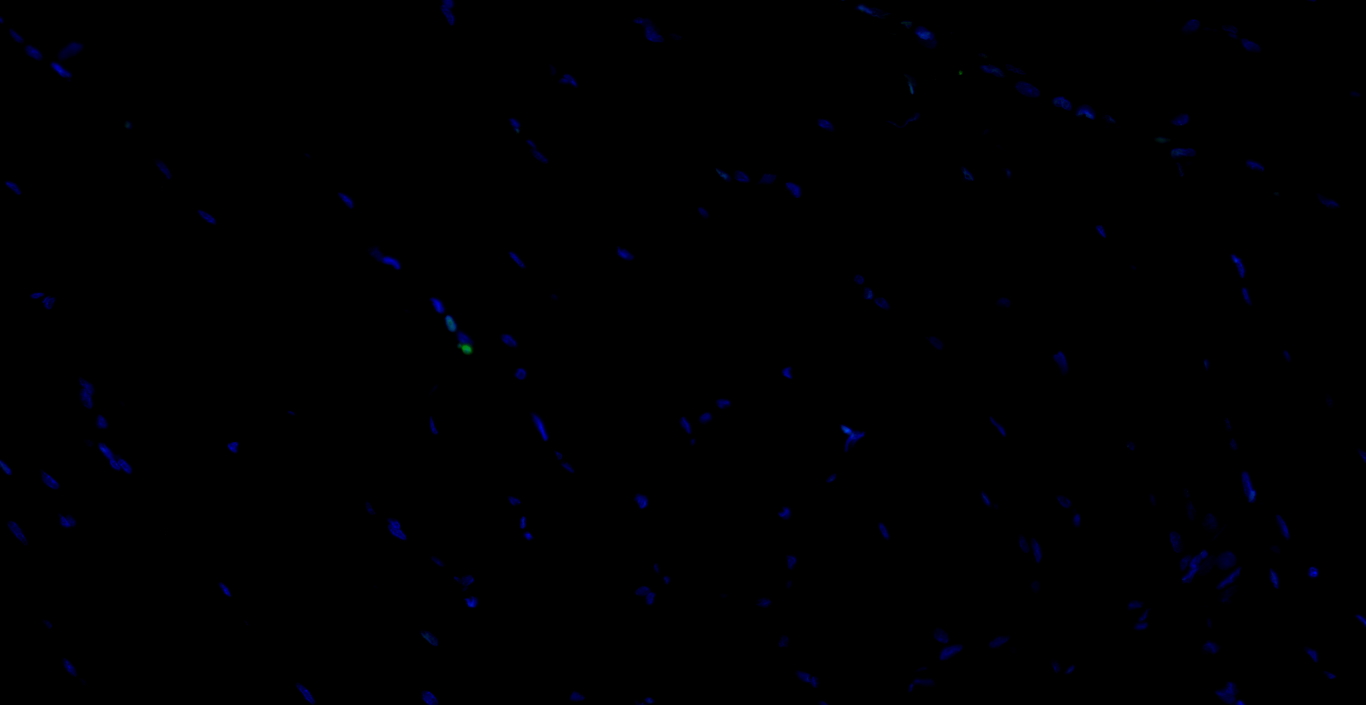

Supplement: Supplementary file 2 [file DataSheet4.ZIP › Supplemental materials 1/TUNEL/VNS/2-3 merge.jpg]

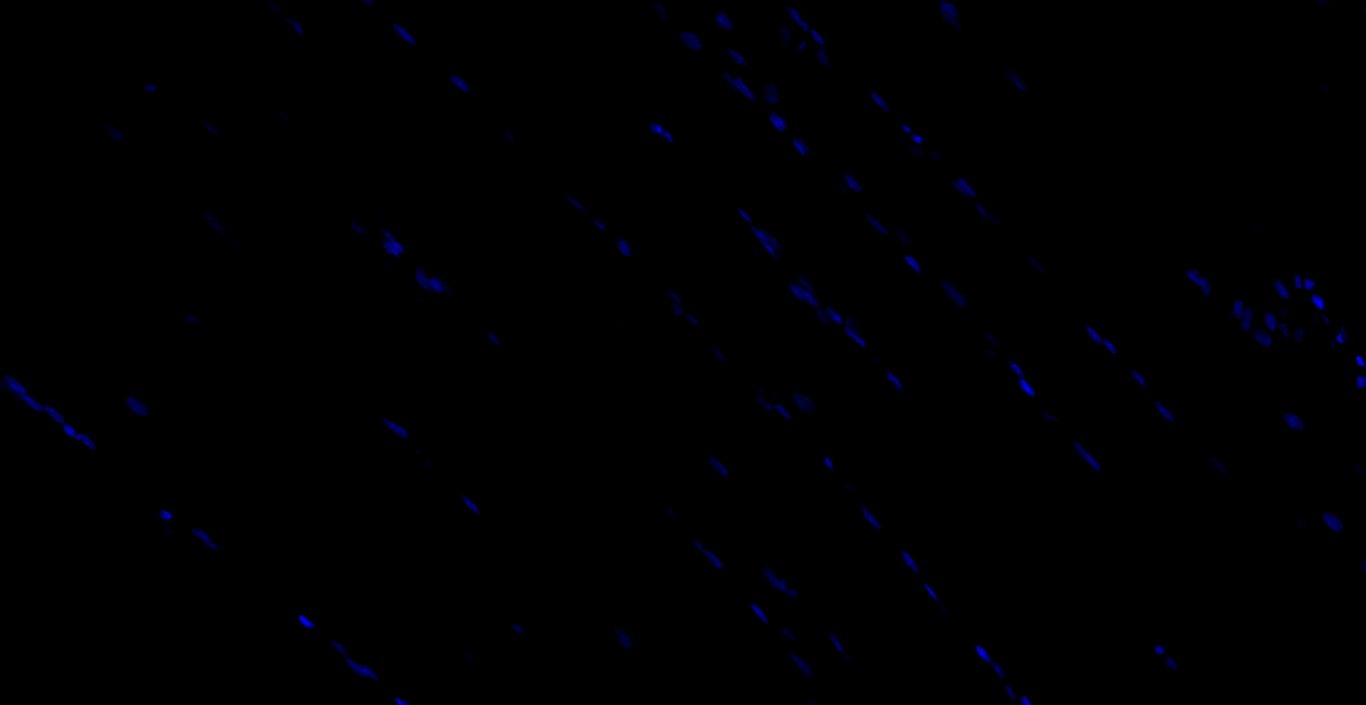

Supplement: Supplementary file 2 [file DataSheet4.ZIP › Supplemental materials 1/TUNEL/VNS/3-1 DAPI.jpg]

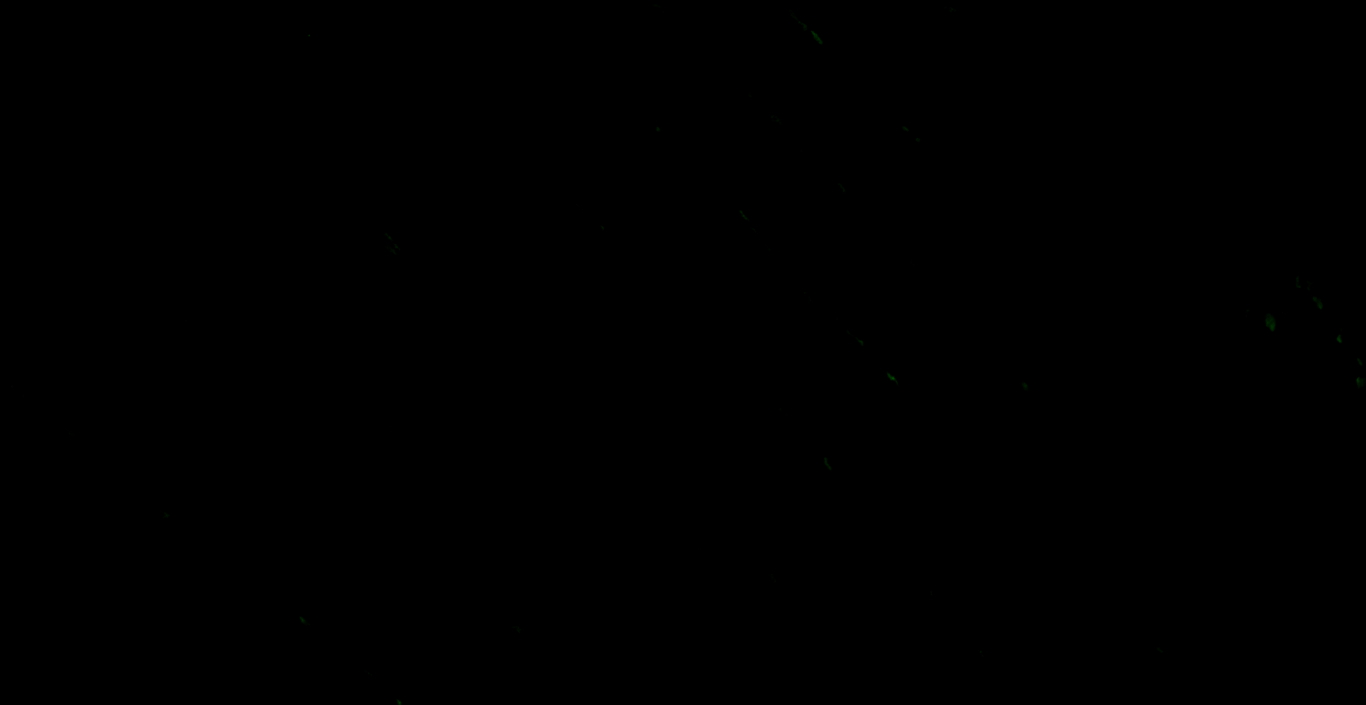

Supplement: Supplementary file 2 [file DataSheet4.ZIP › Supplemental materials 1/TUNEL/VNS/3-1 TUNEL.jpg]

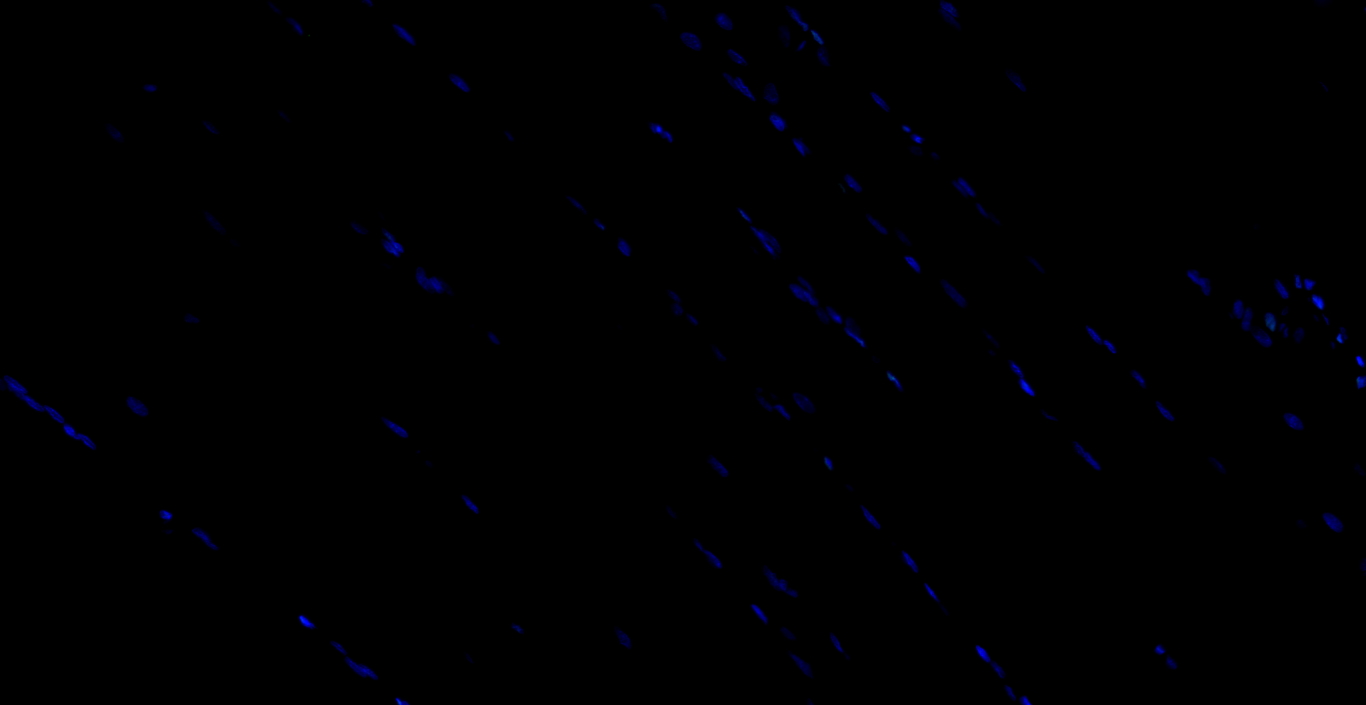

Supplement: Supplementary file 2 [file DataSheet4.ZIP › Supplemental materials 1/TUNEL/VNS/3-1 merge.jpg]

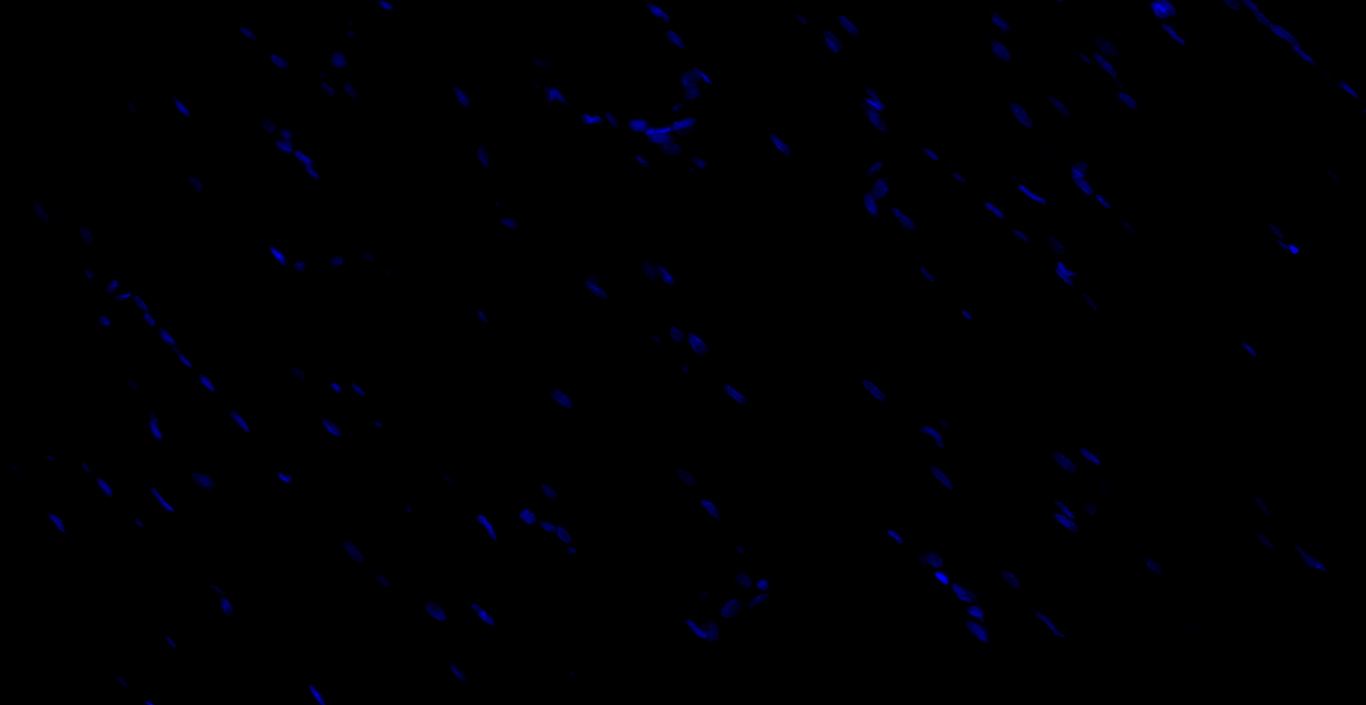

Supplement: Supplementary file 2 [file DataSheet4.ZIP › Supplemental materials 1/TUNEL/VNS/3-2 DAPI.jpg]

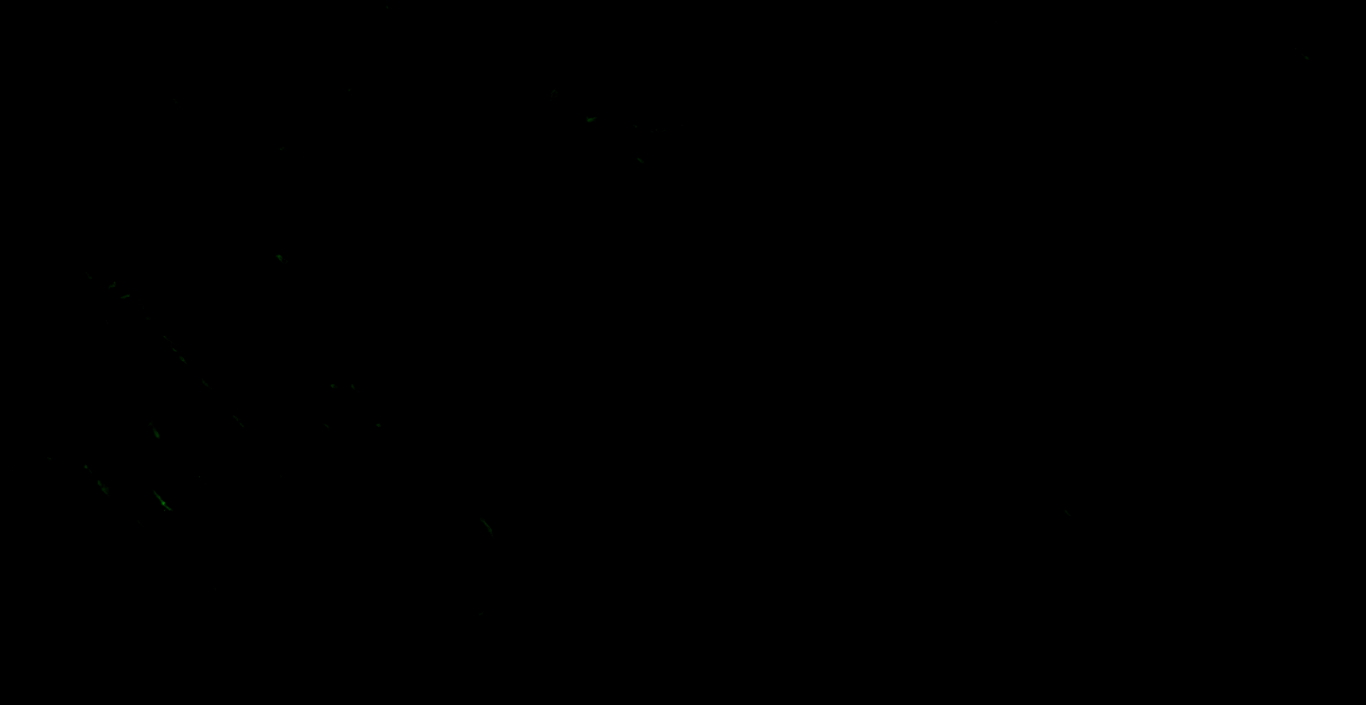

Supplement: Supplementary file 2 [file DataSheet4.ZIP › Supplemental materials 1/TUNEL/VNS/3-2 TUNEL.jpg]

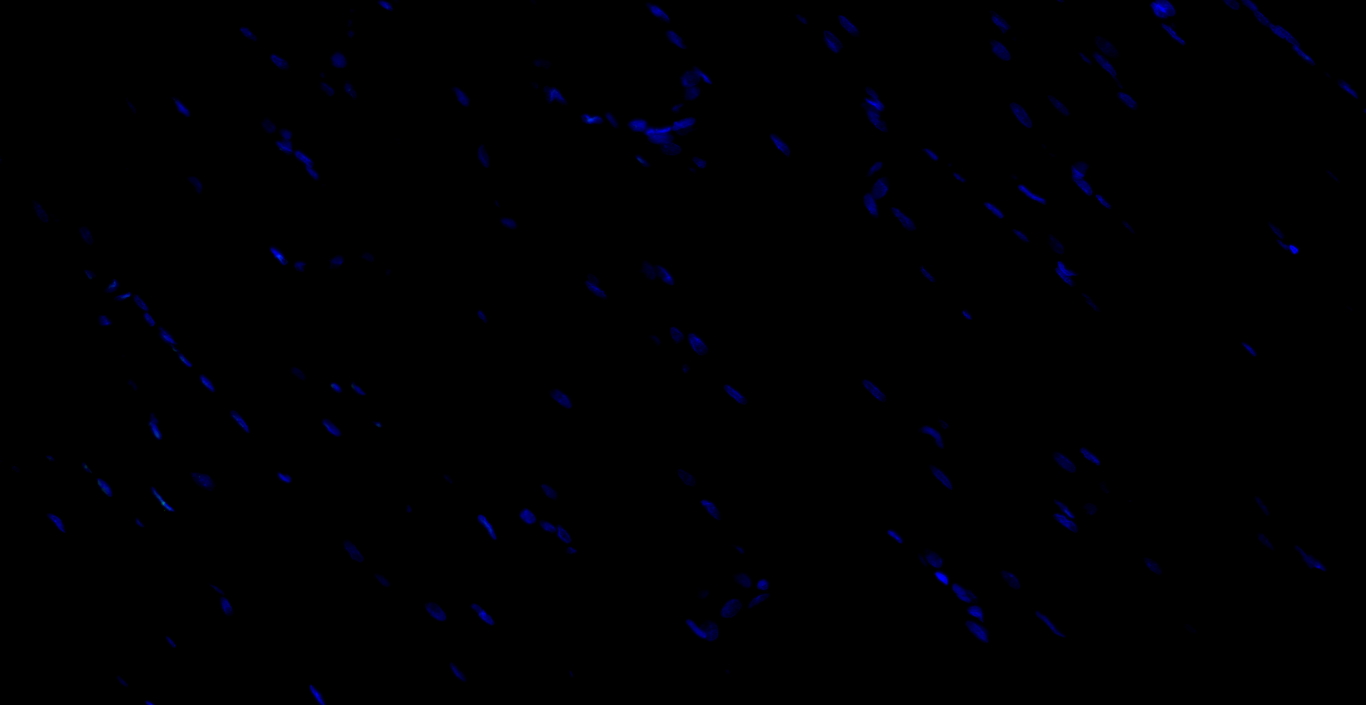

Supplement: Supplementary file 2 [file DataSheet4.ZIP › Supplemental materials 1/TUNEL/VNS/3-2 merge.jpg]

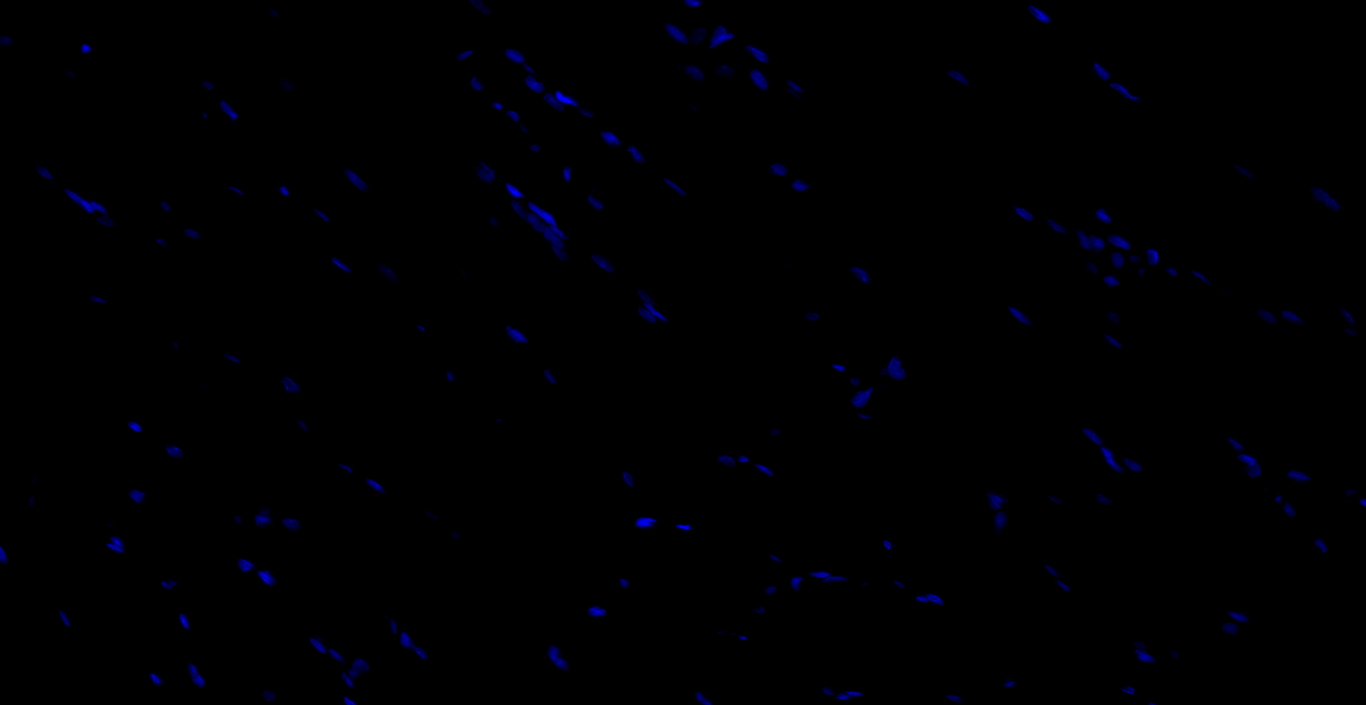

Supplement: Supplementary file 2 [file DataSheet4.ZIP › Supplemental materials 1/TUNEL/VNS/3-3 DAPI.jpg]

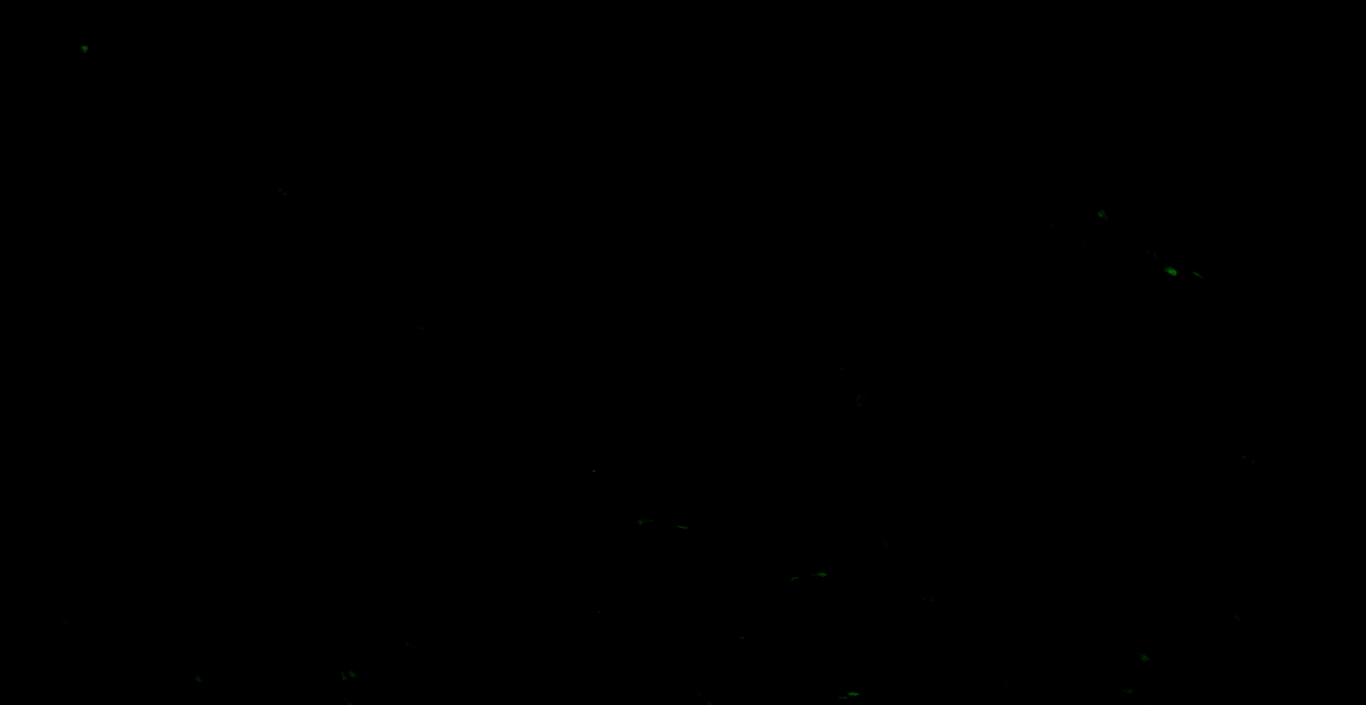

Supplement: Supplementary file 2 [file DataSheet4.ZIP › Supplemental materials 1/TUNEL/VNS/3-3 TUNEL.jpg]

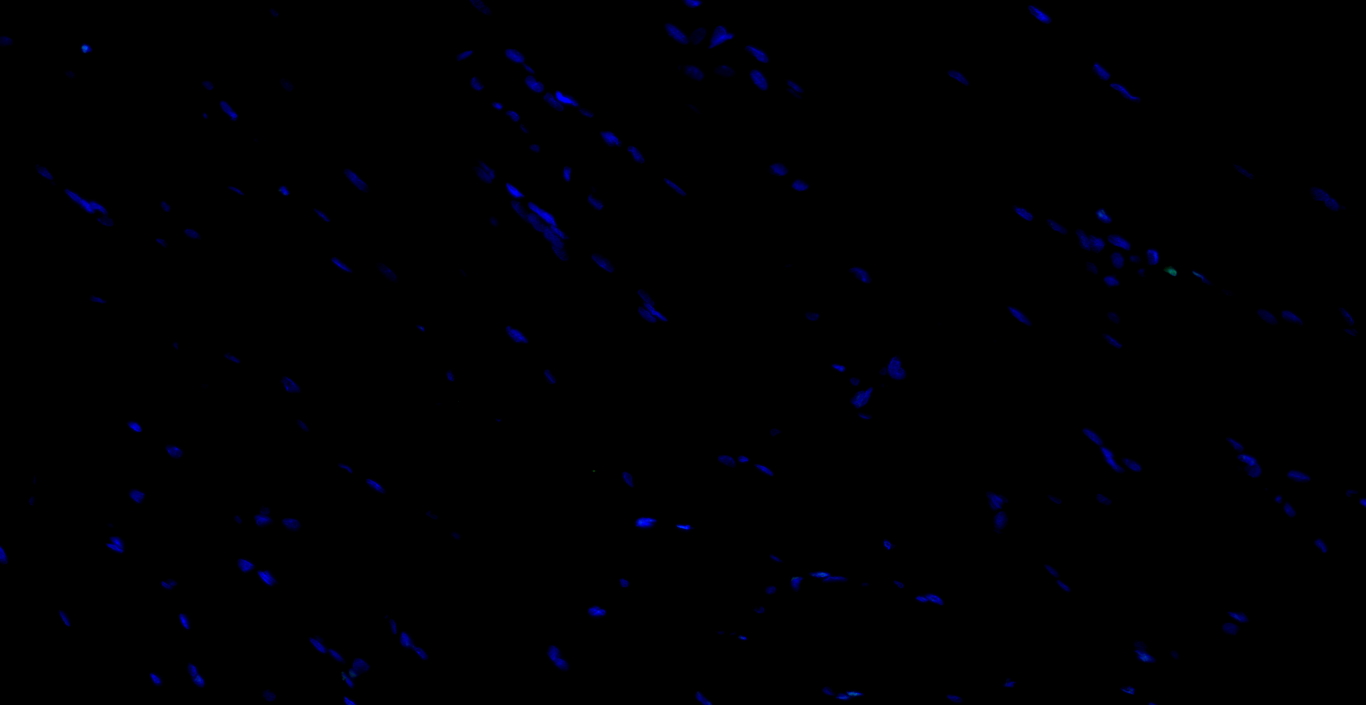

Supplement: Supplementary file 2 [file DataSheet4.ZIP › Supplemental materials 1/TUNEL/VNS/3-3 merge.jpg]

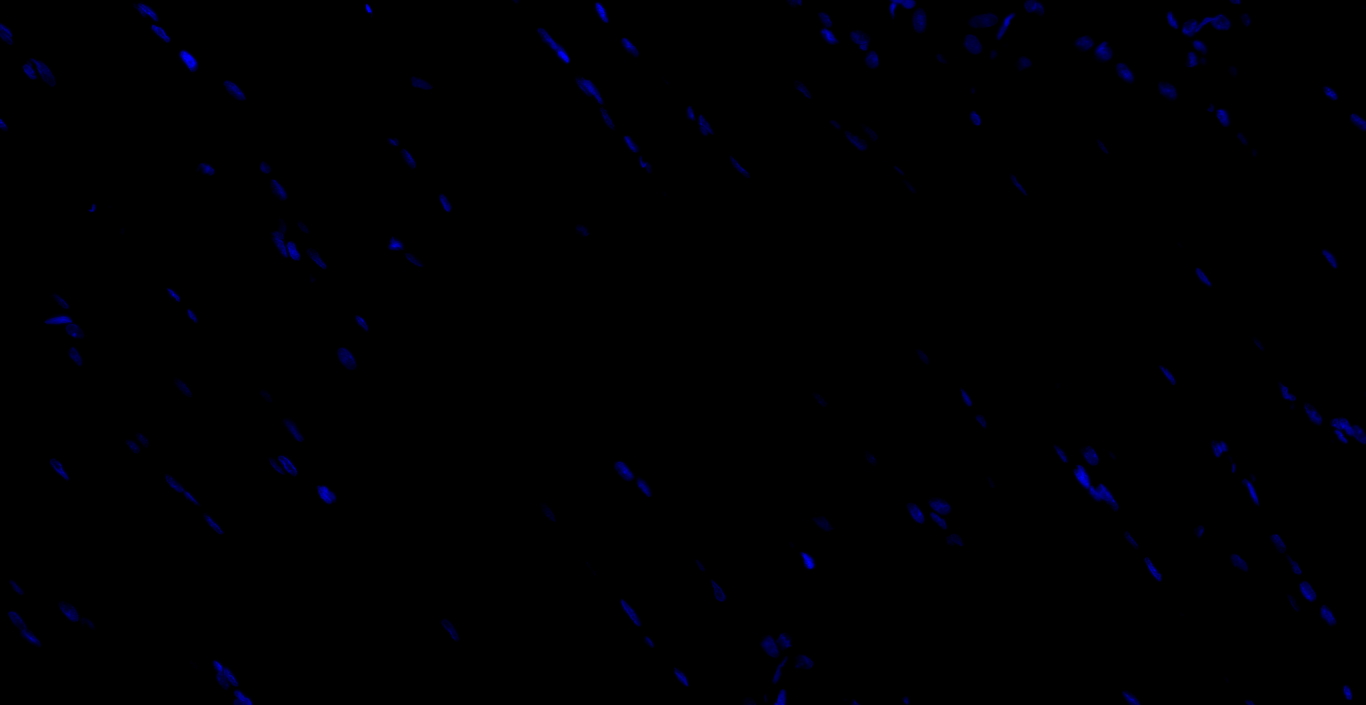

Supplement: Supplementary file 2 [file DataSheet4.ZIP › Supplemental materials 1/TUNEL/VNS/4-1 DAPI.jpg]

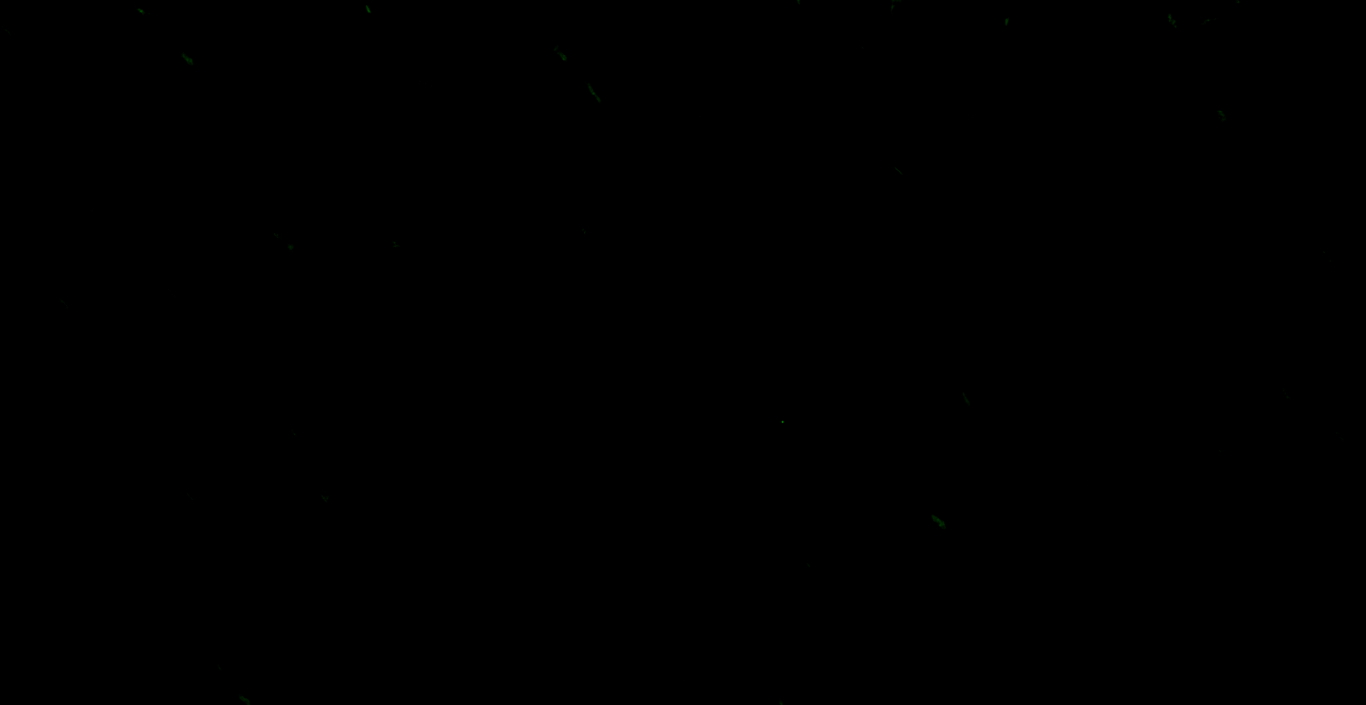

Supplement: Supplementary file 2 [file DataSheet4.ZIP › Supplemental materials 1/TUNEL/VNS/4-1 TUNEL.jpg]

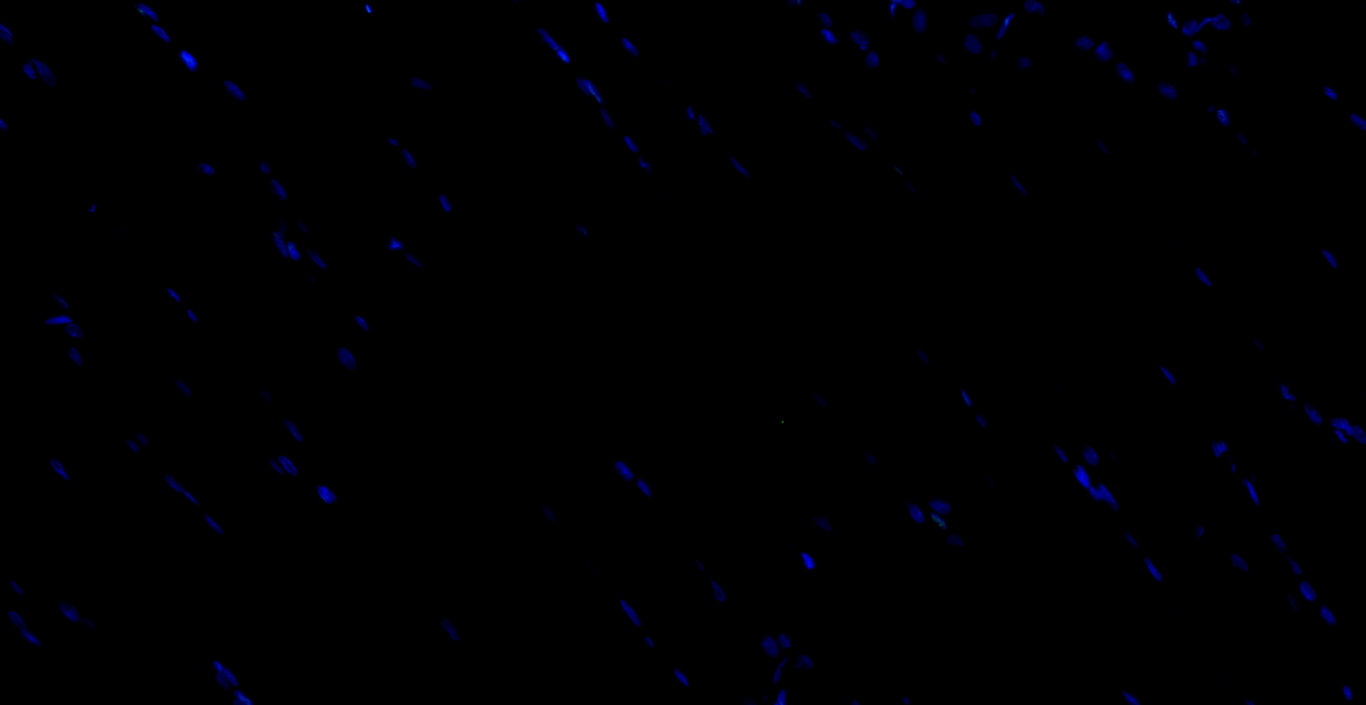

Supplement: Supplementary file 2 [file DataSheet4.ZIP › Supplemental materials 1/TUNEL/VNS/4-1 merge.jpg]

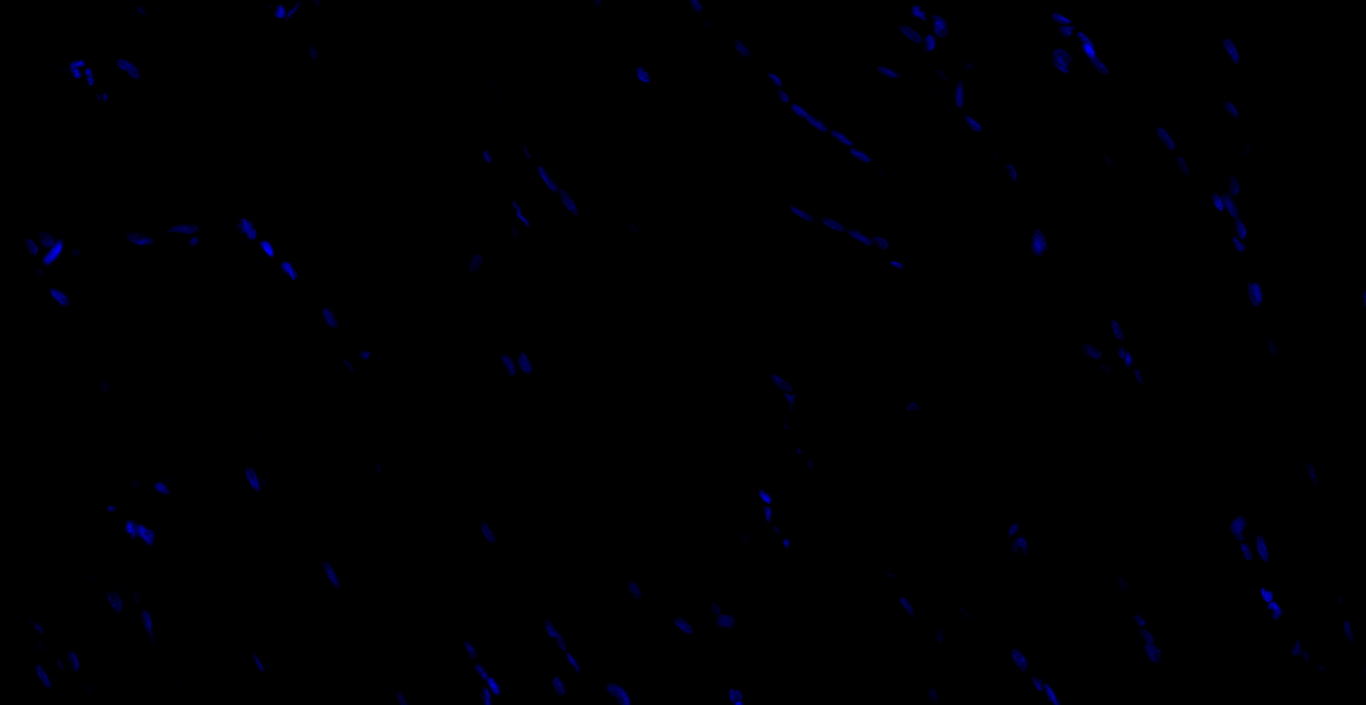

Supplement: Supplementary file 2 [file DataSheet4.ZIP › Supplemental materials 1/TUNEL/VNS/4-2 DAPI.jpg]

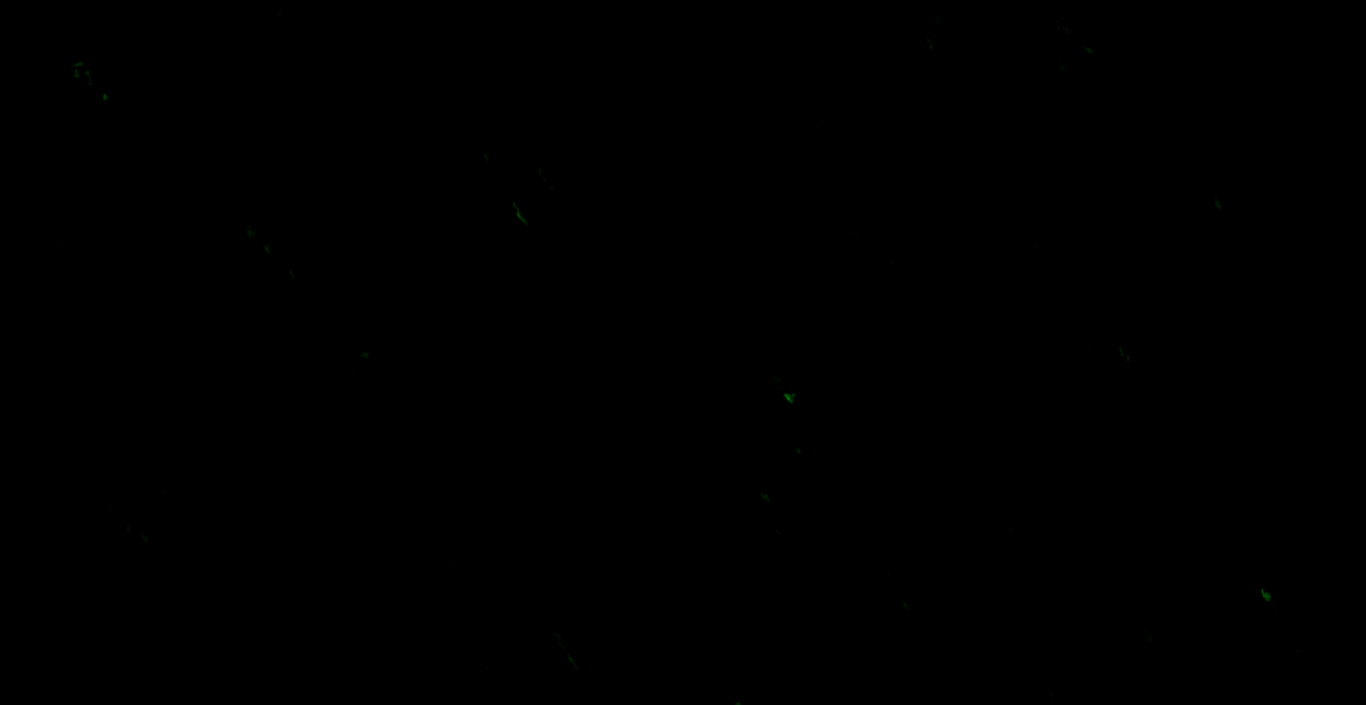

Supplement: Supplementary file 2 [file DataSheet4.ZIP › Supplemental materials 1/TUNEL/VNS/4-2 TUNEL.jpg]

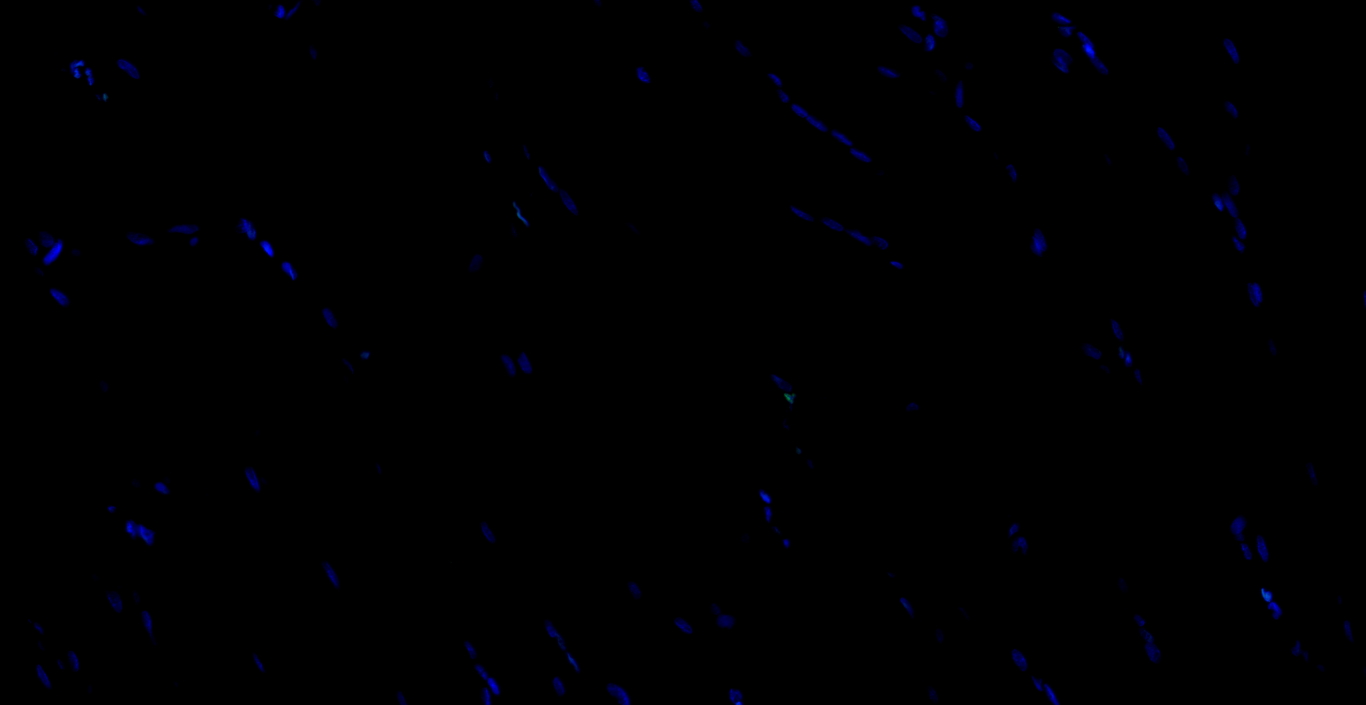

Supplement: Supplementary file 2 [file DataSheet4.ZIP › Supplemental materials 1/TUNEL/VNS/4-2 merge.jpg]

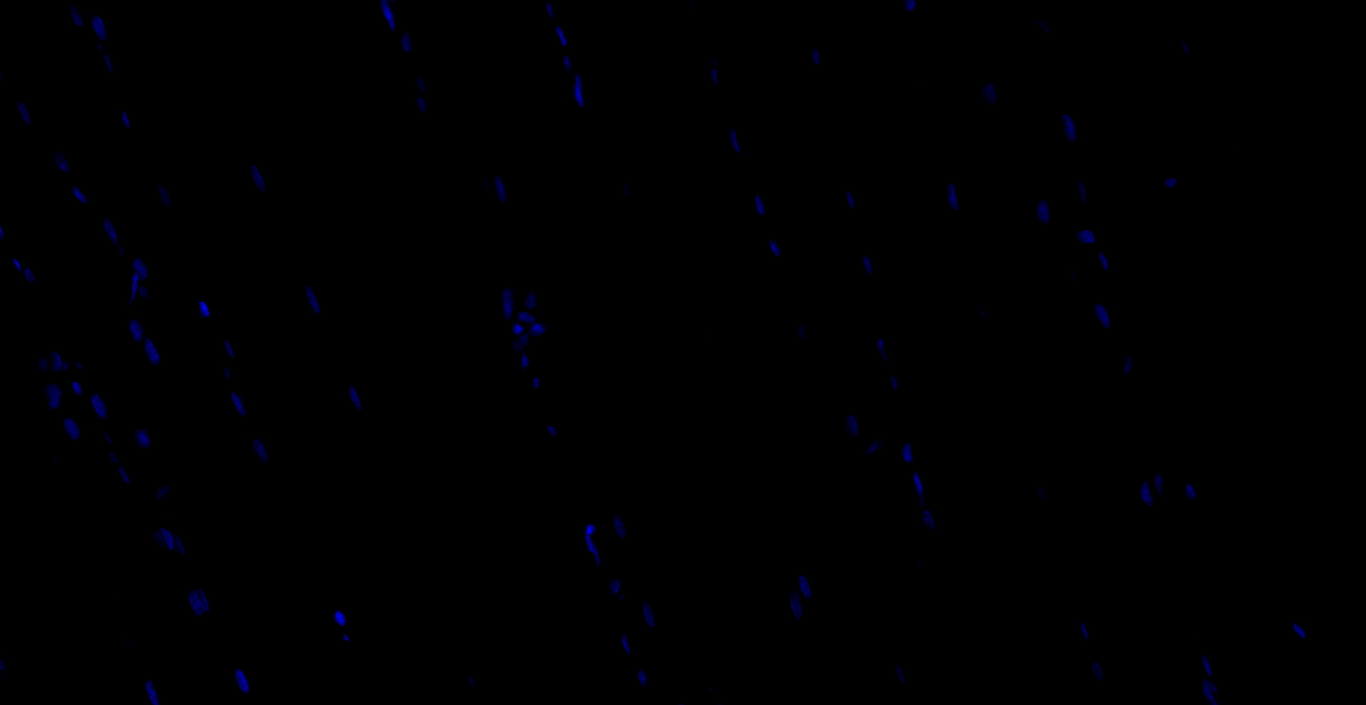

Supplement: Supplementary file 2 [file DataSheet4.ZIP › Supplemental materials 1/TUNEL/VNS/4-3 DAPI.jpg]

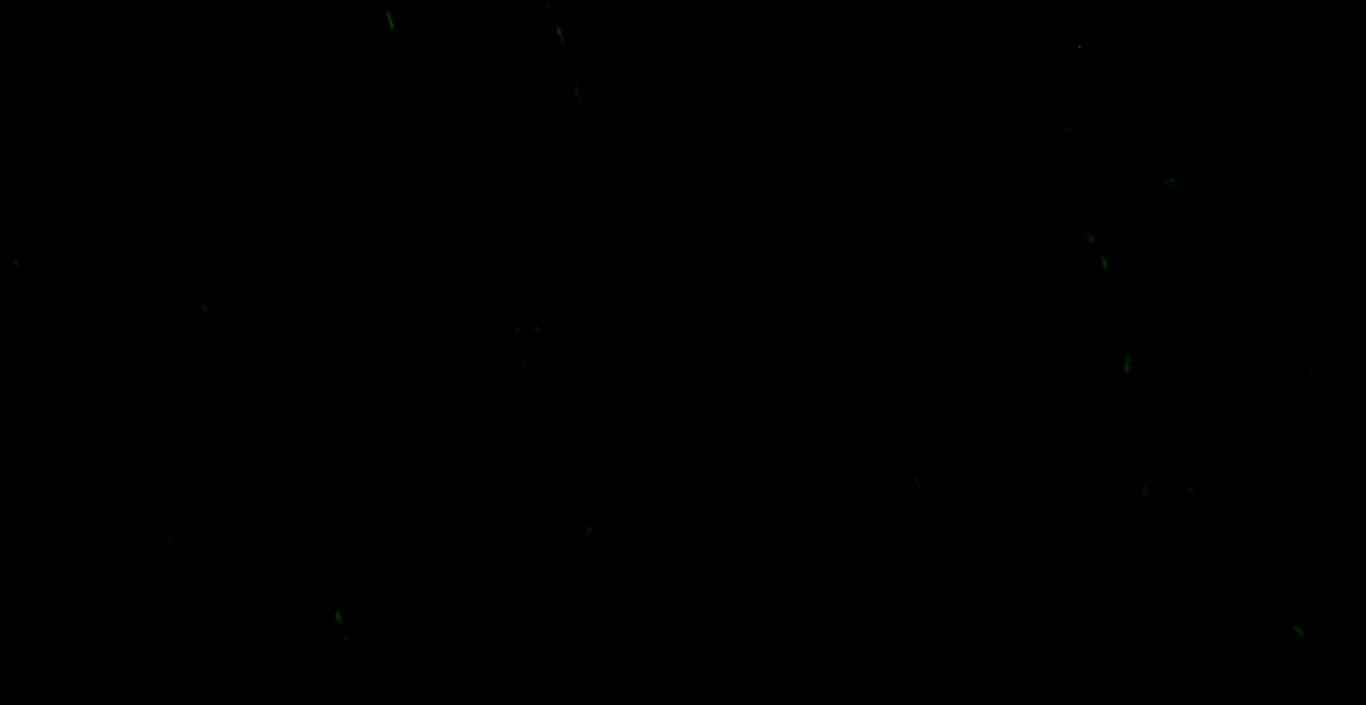

Supplement: Supplementary file 2 [file DataSheet4.ZIP › Supplemental materials 1/TUNEL/VNS/4-3 TUNEL.jpg]

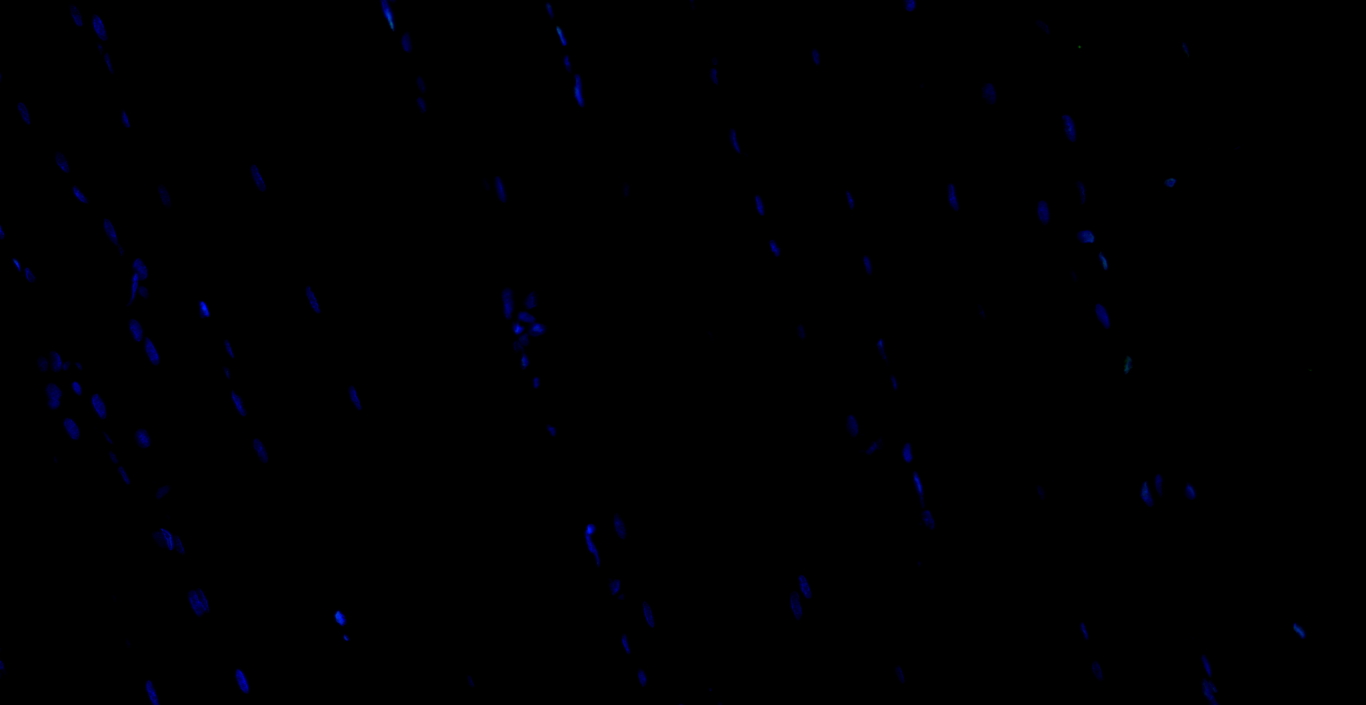

Supplement: Supplementary file 2 [file DataSheet4.ZIP › Supplemental materials 1/TUNEL/VNS/4-3 merge.jpg]

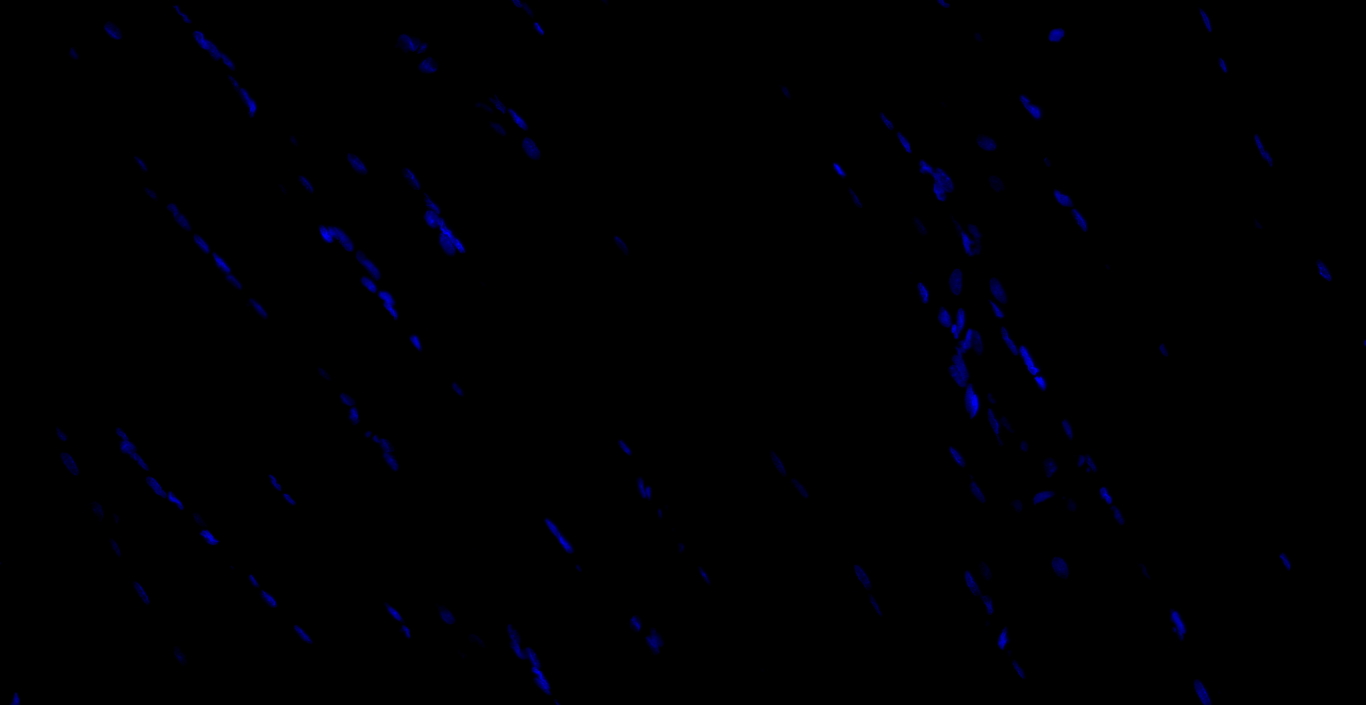

Supplement: Supplementary file 2 [file DataSheet4.ZIP › Supplemental materials 1/TUNEL/VNS/5-1 DAPI.jpg]

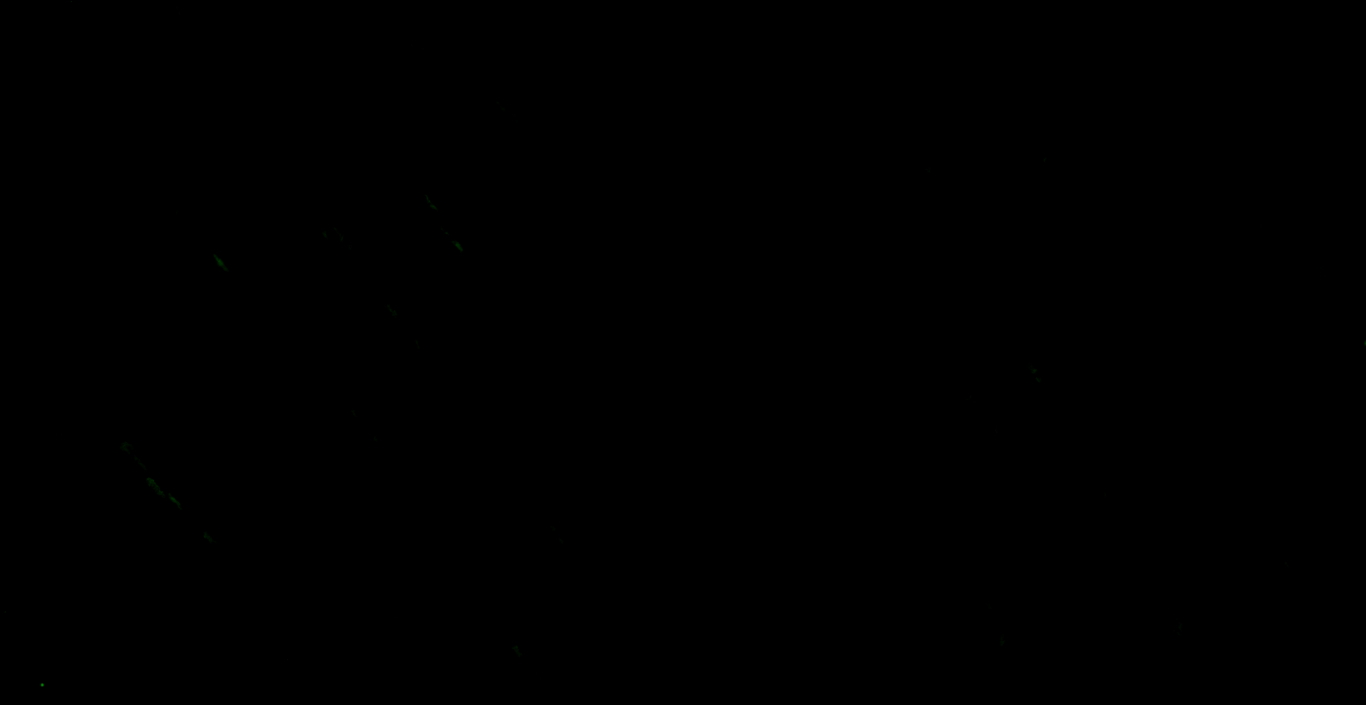

Supplement: Supplementary file 2 [file DataSheet4.ZIP › Supplemental materials 1/TUNEL/VNS/5-1 TUNEL.jpg]

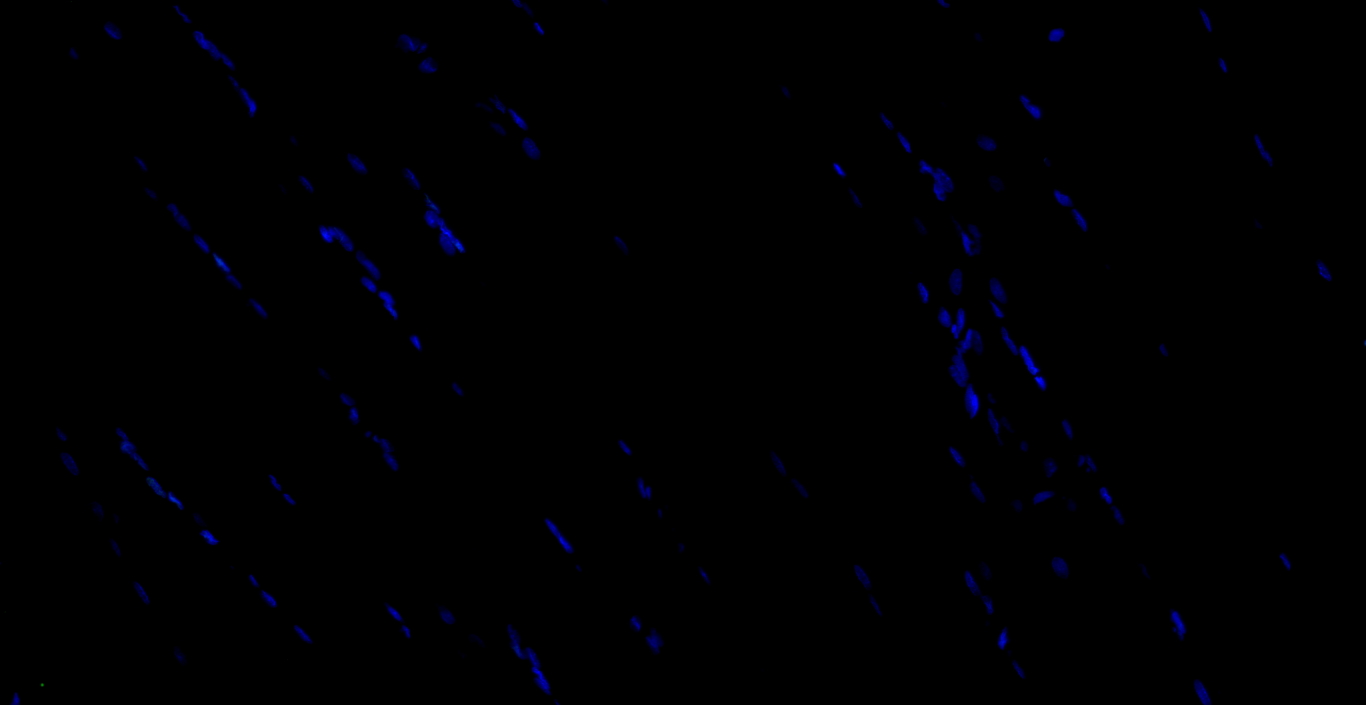

Supplement: Supplementary file 2 [file DataSheet4.ZIP › Supplemental materials 1/TUNEL/VNS/5-1 merge.jpg]

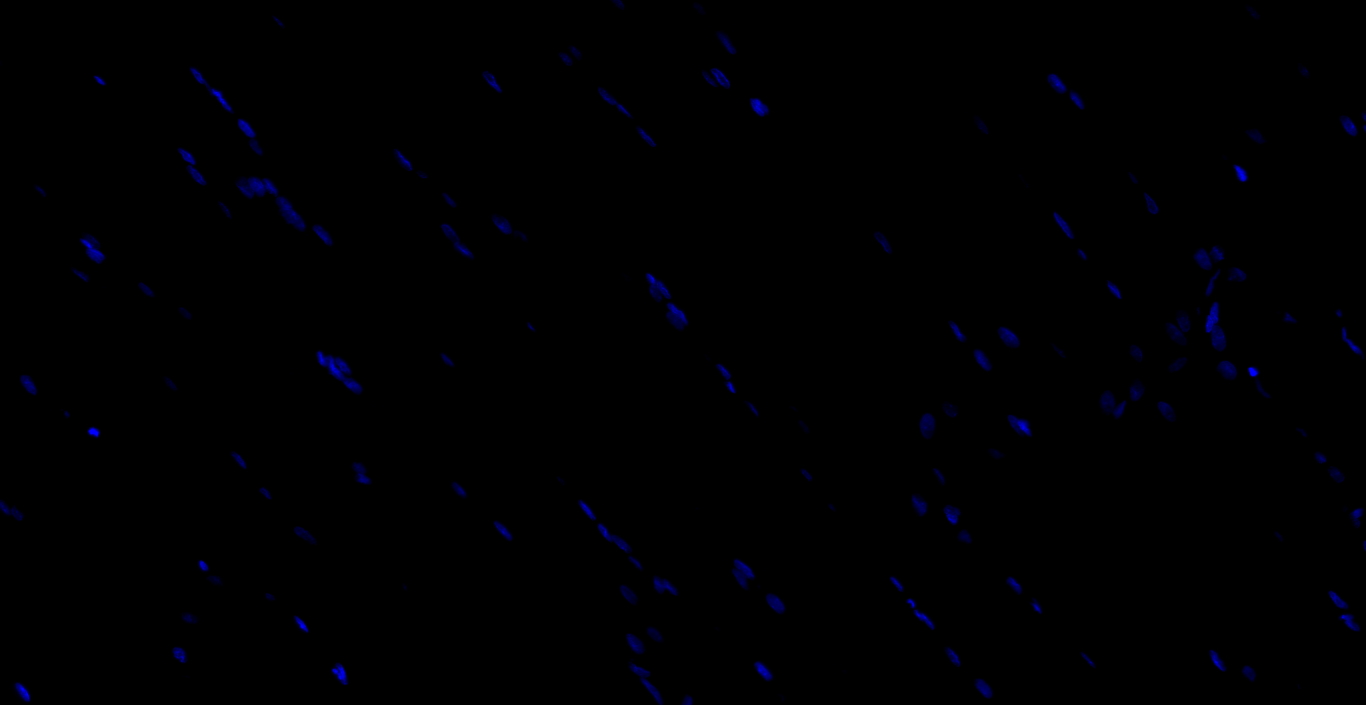

Supplement: Supplementary file 2 [file DataSheet4.ZIP › Supplemental materials 1/TUNEL/VNS/5-2 DAPI.jpg]

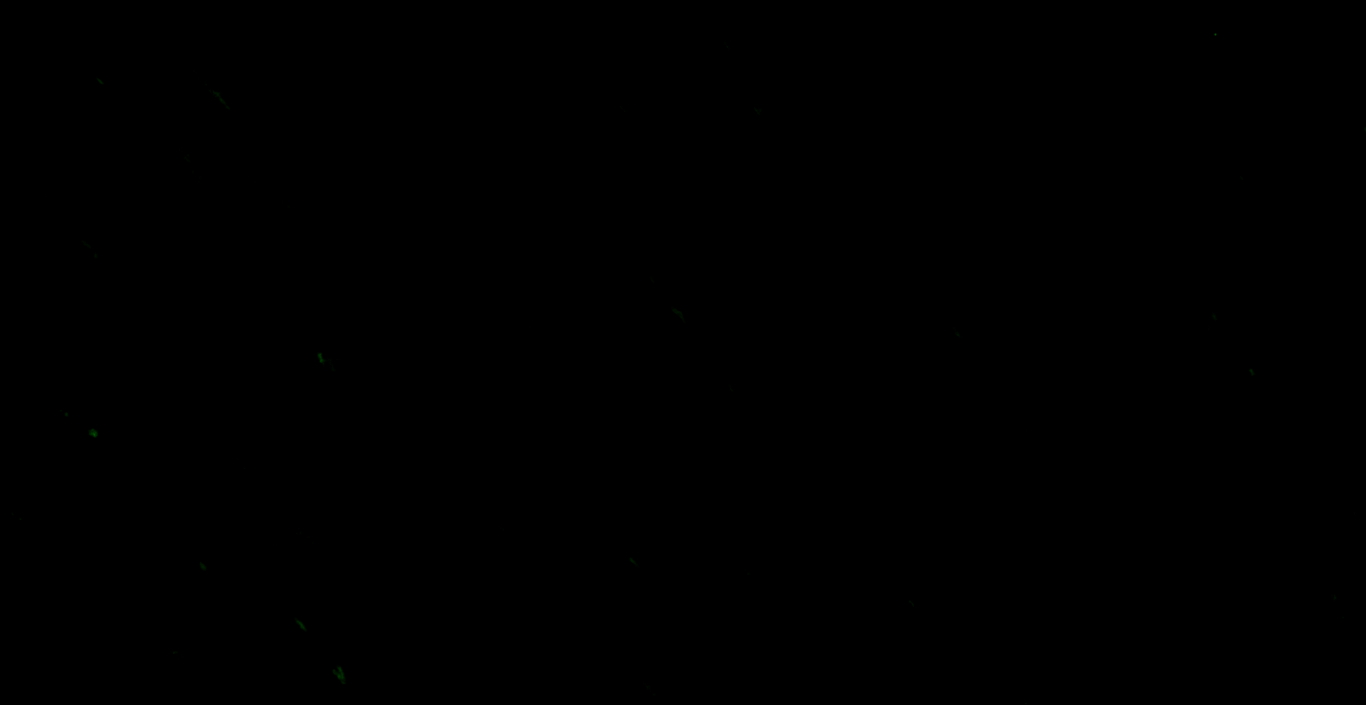

Supplement: Supplementary file 2 [file DataSheet4.ZIP › Supplemental materials 1/TUNEL/VNS/5-2 TUNEL.jpg]

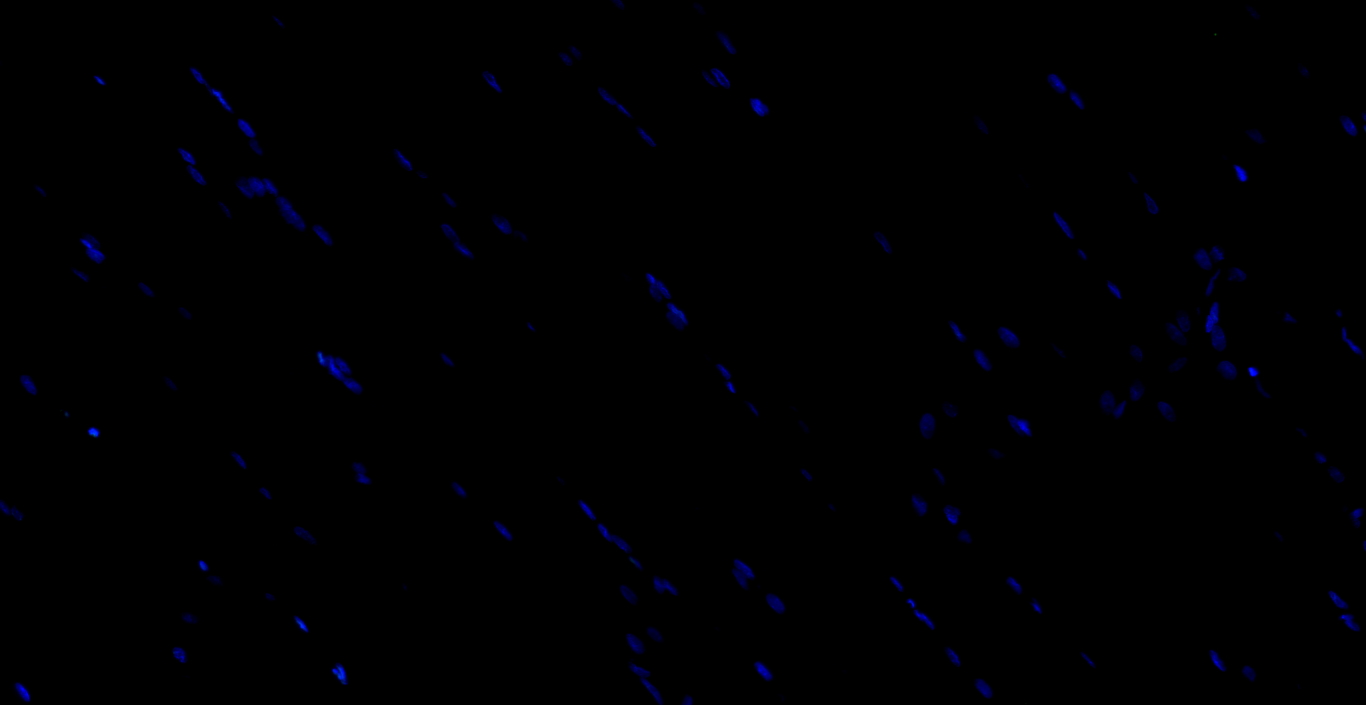

Supplement: Supplementary file 2 [file DataSheet4.ZIP › Supplemental materials 1/TUNEL/VNS/5-2 merge.jpg]

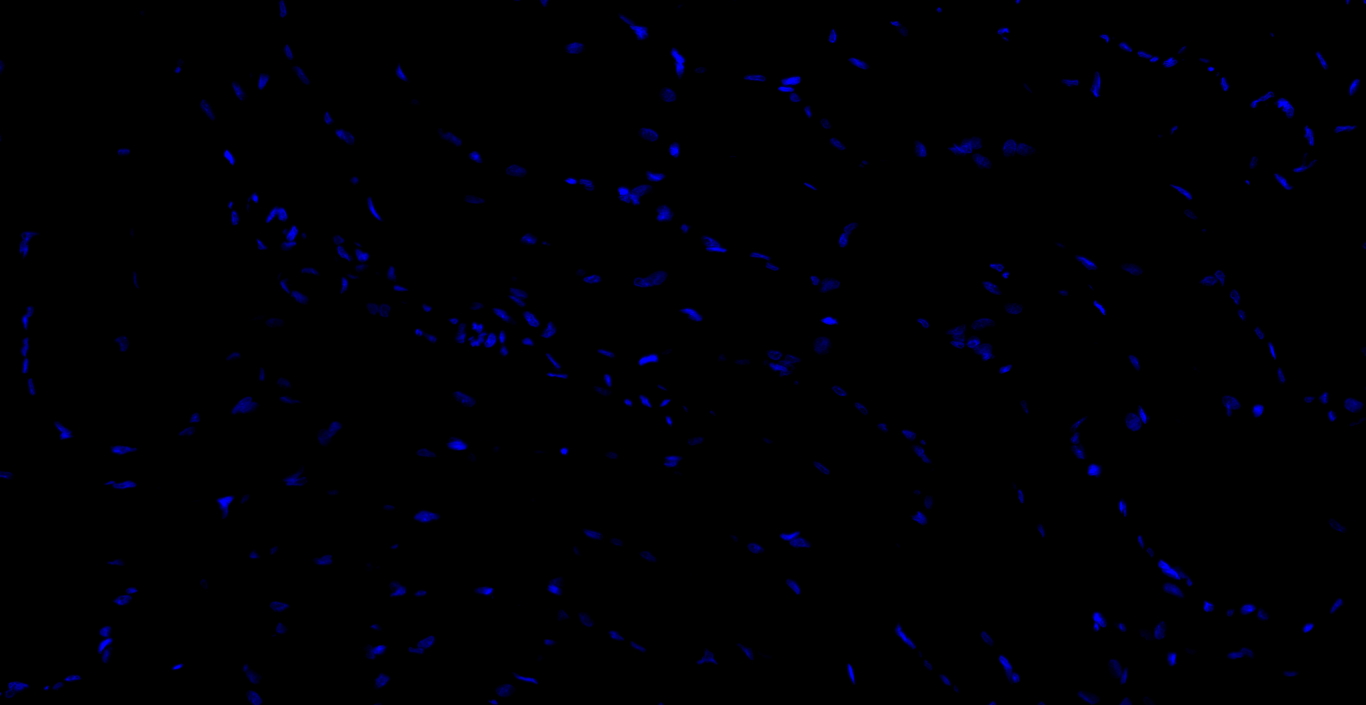

Supplement: Supplementary file 2 [file DataSheet4.ZIP › Supplemental materials 1/TUNEL/VNS/5-3 DAPI.jpg]

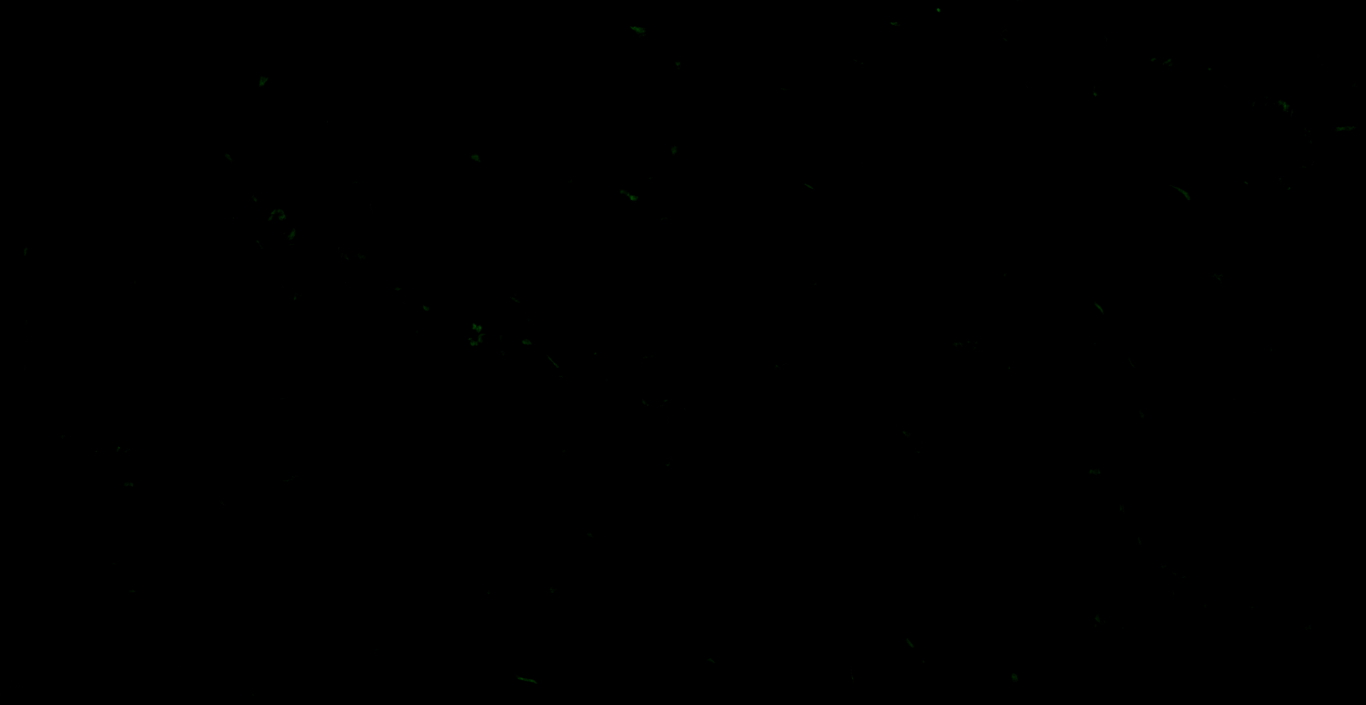

Supplement: Supplementary file 2 [file DataSheet4.ZIP › Supplemental materials 1/TUNEL/VNS/5-3 TUNEL.jpg]

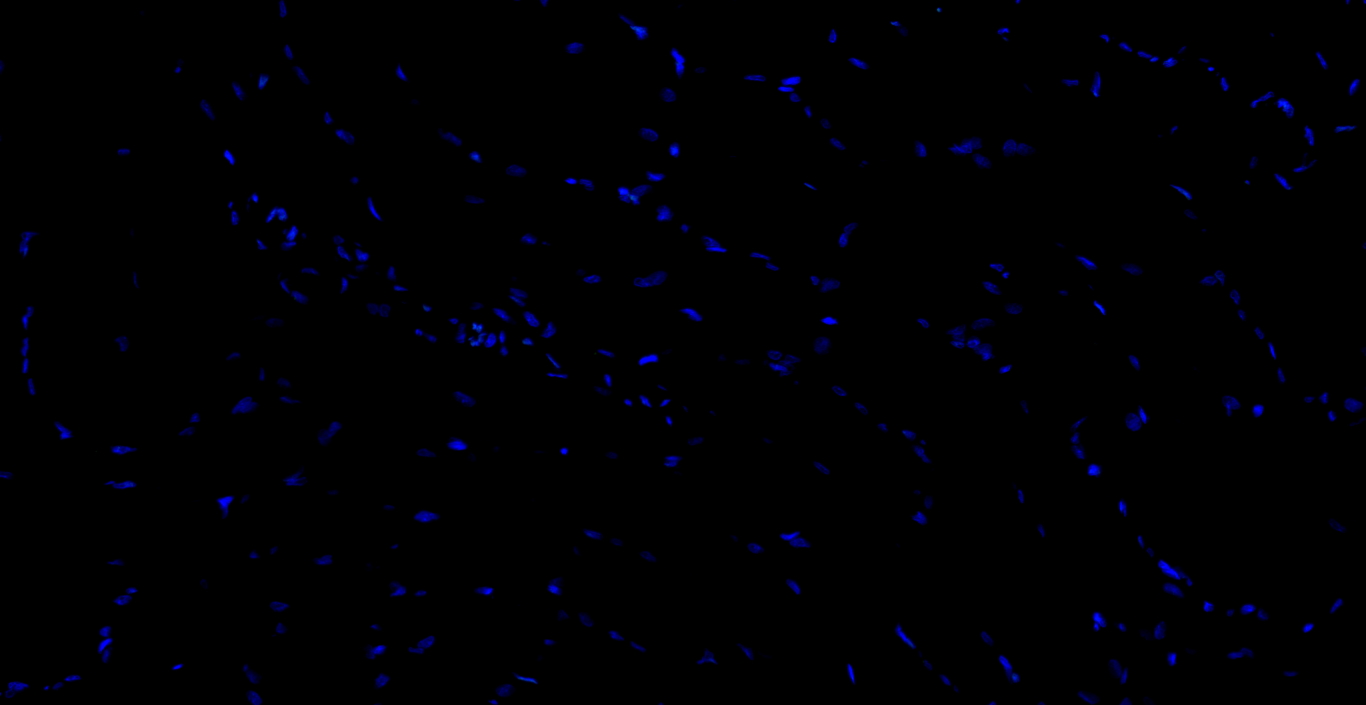

Supplement: Supplementary file 2 [file DataSheet4.ZIP › Supplemental materials 1/TUNEL/VNS/5-3 merge.jpg]

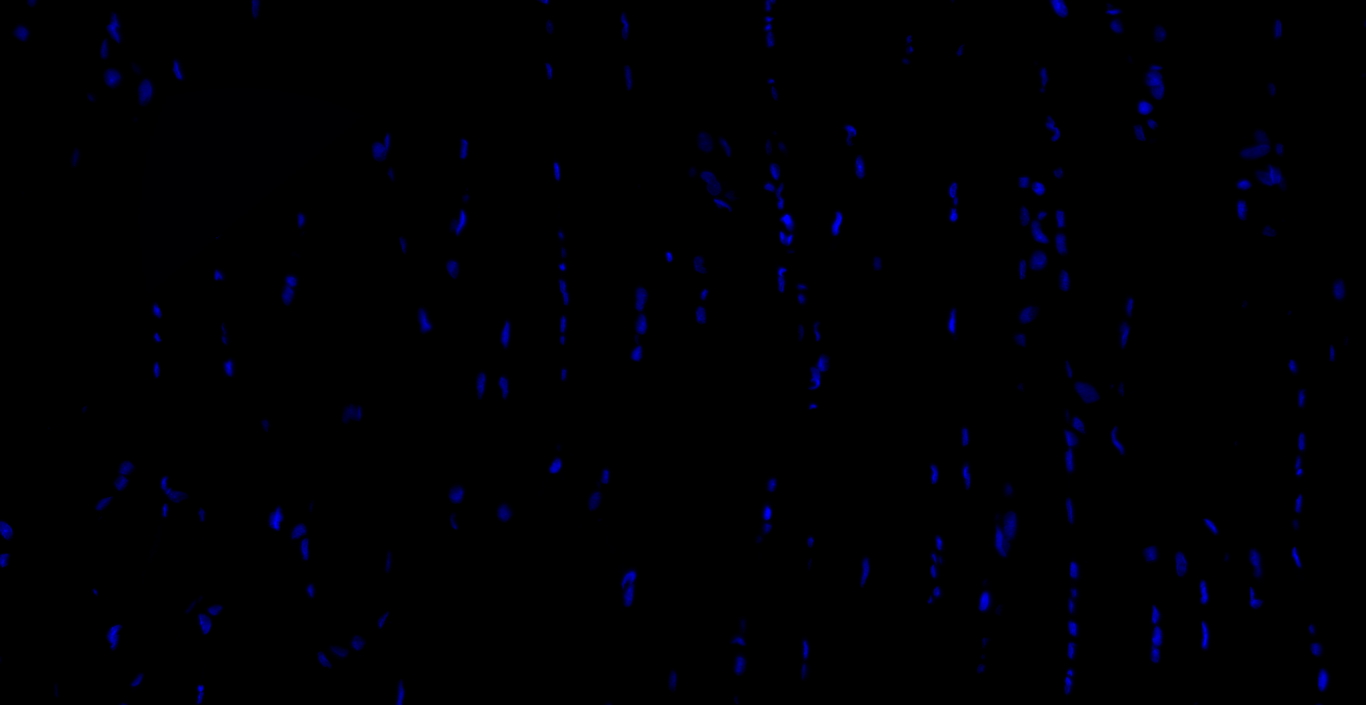

Supplement: Supplementary file 2 [file DataSheet4.ZIP › Supplemental materials 1/TUNEL/VNS/6-1 DAPI.jpg]

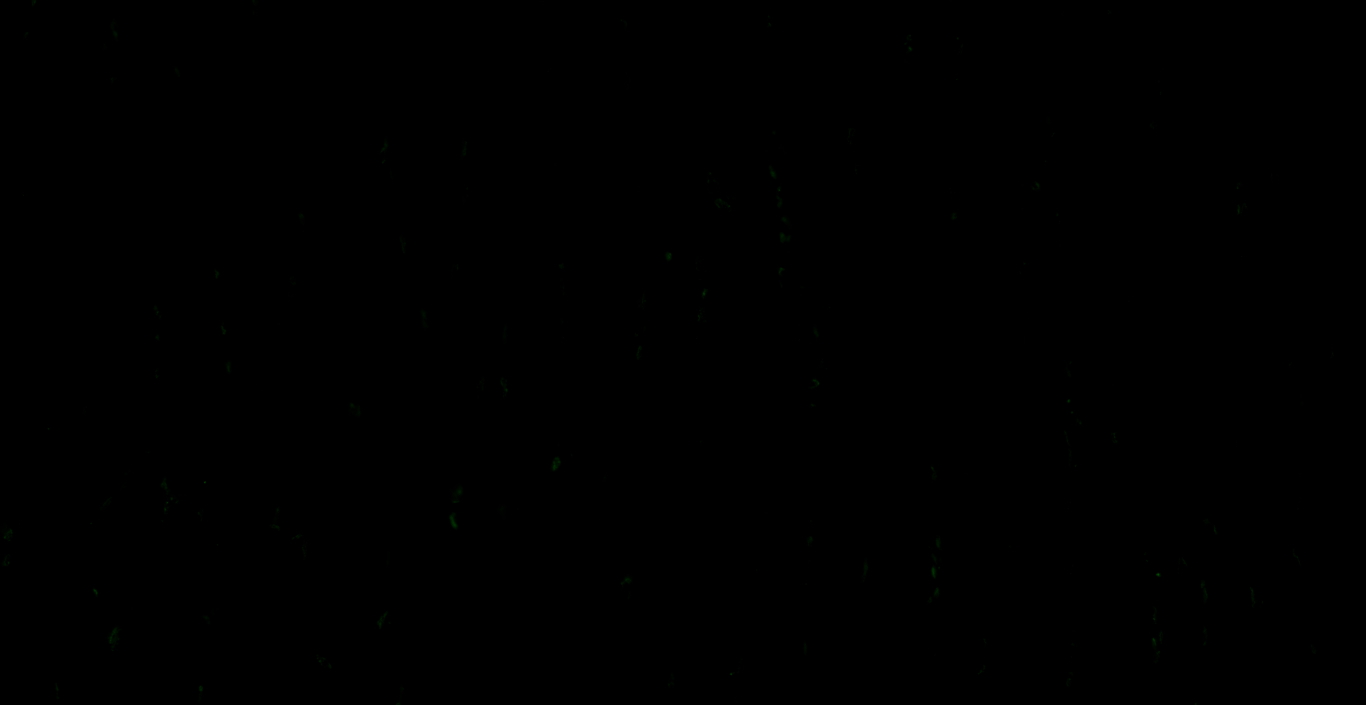

Supplement: Supplementary file 2 [file DataSheet4.ZIP › Supplemental materials 1/TUNEL/VNS/6-1 TUNEL.jpg]

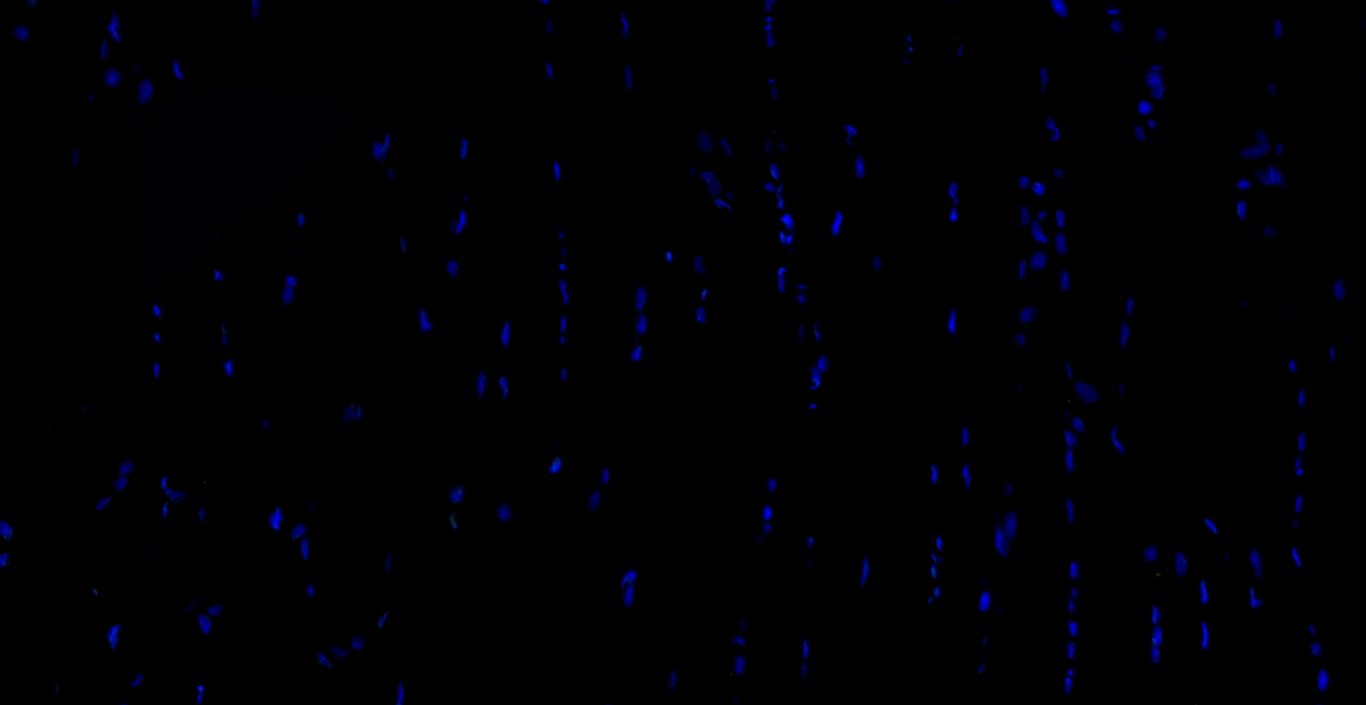

Supplement: Supplementary file 2 [file DataSheet4.ZIP › Supplemental materials 1/TUNEL/VNS/6-1 merge.jpg]

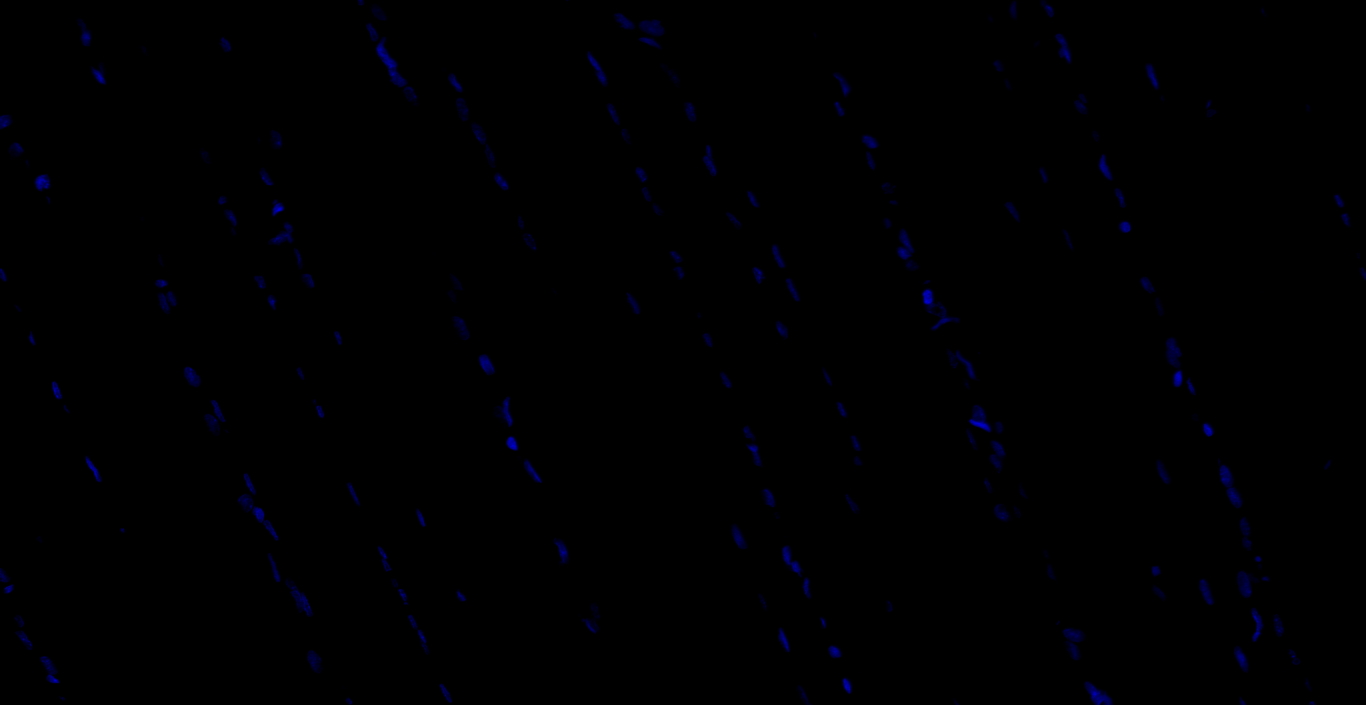

Supplement: Supplementary file 2 [file DataSheet4.ZIP › Supplemental materials 1/TUNEL/VNS/6-2 DAPI.jpg]

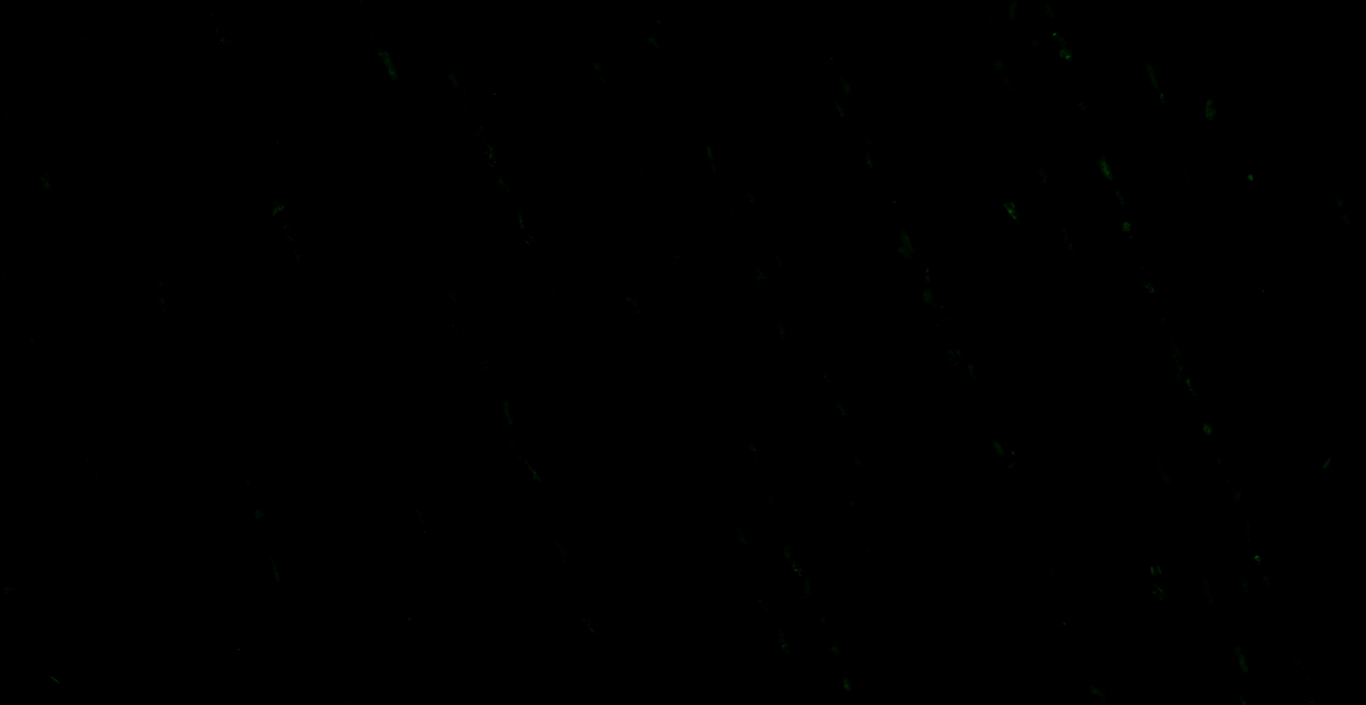

Supplement: Supplementary file 2 [file DataSheet4.ZIP › Supplemental materials 1/TUNEL/VNS/6-2 TUNEL.jpg]

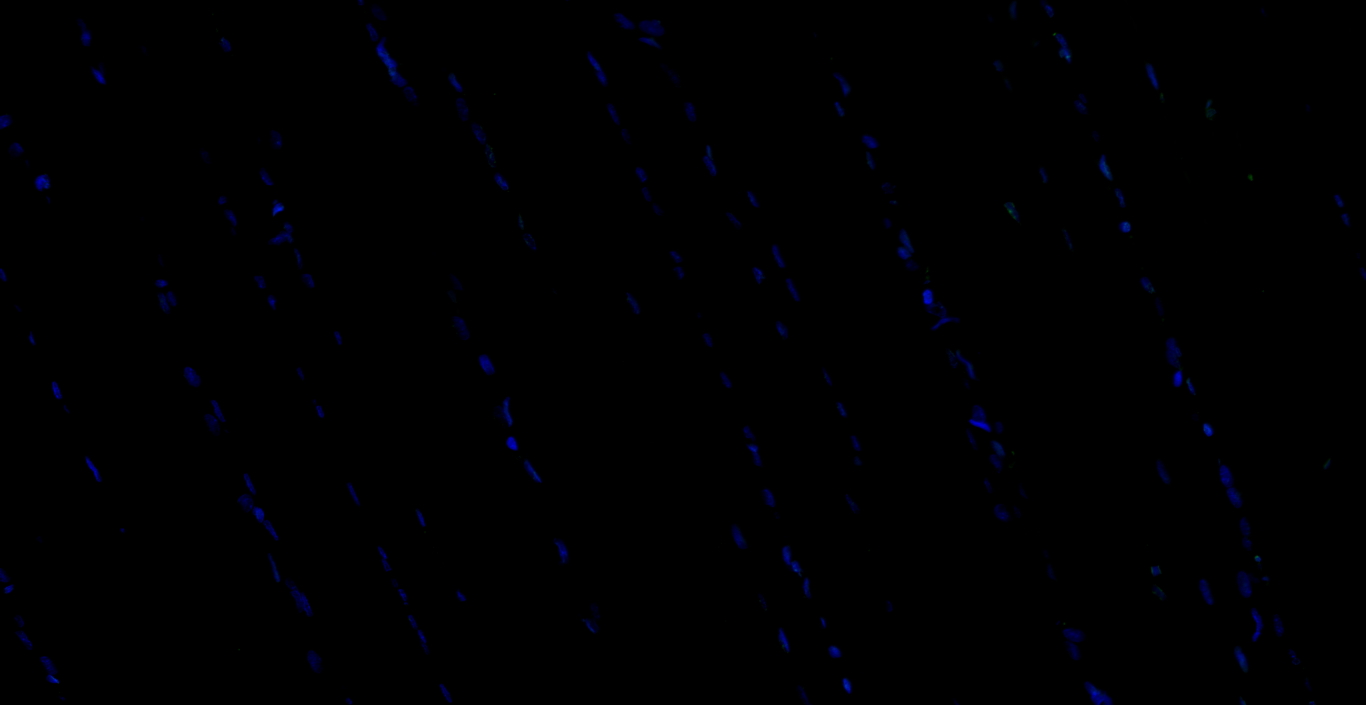

Supplement: Supplementary file 2 [file DataSheet4.ZIP › Supplemental materials 1/TUNEL/VNS/6-2 merge.jpg]

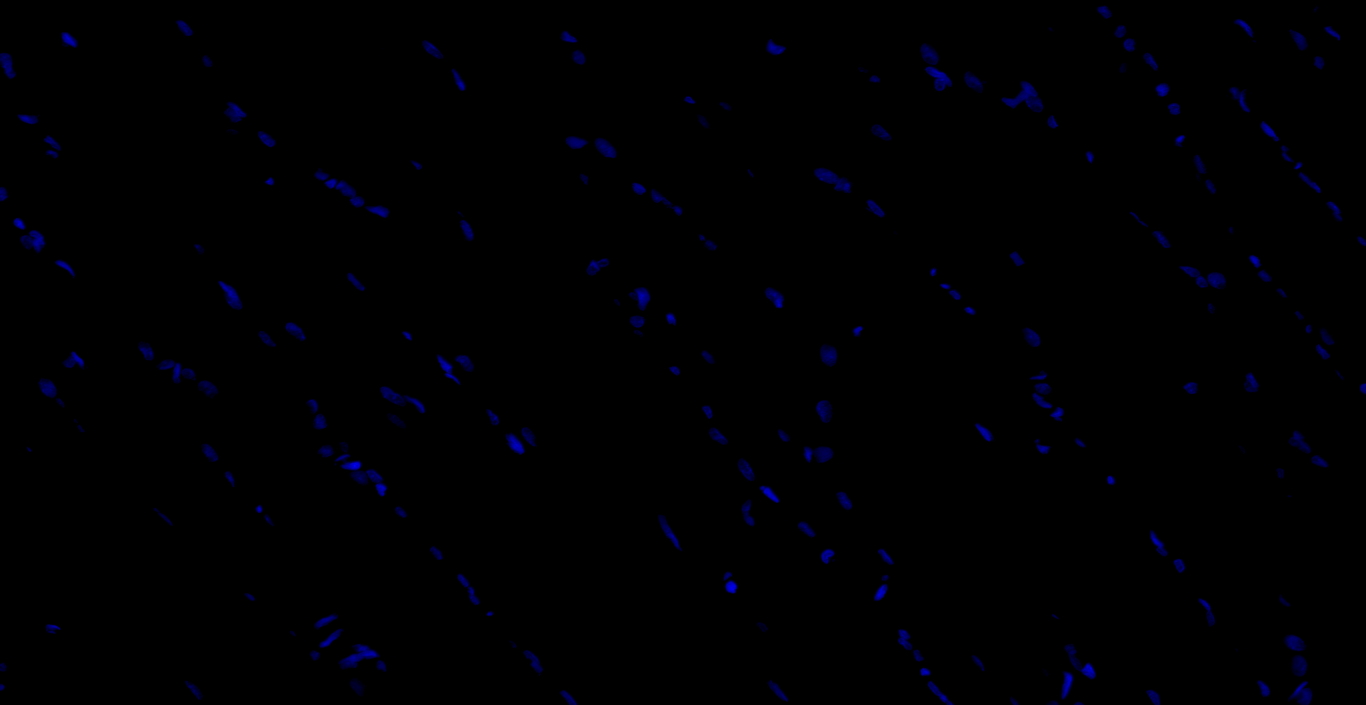

Supplement: Supplementary file 2 [file DataSheet4.ZIP › Supplemental materials 1/TUNEL/VNS/6-3 DAPI.jpg]

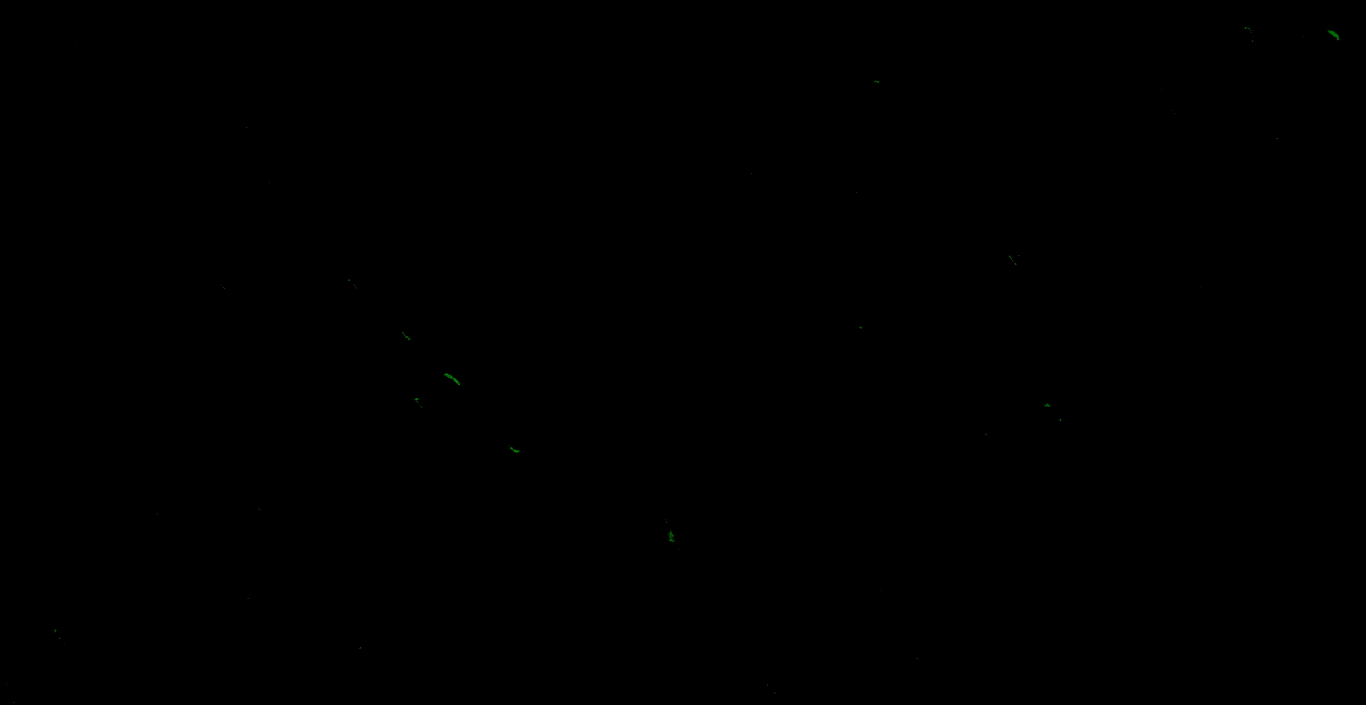

Supplement: Supplementary file 2 [file DataSheet4.ZIP › Supplemental materials 1/TUNEL/VNS/6-3 TUNEL.jpg]

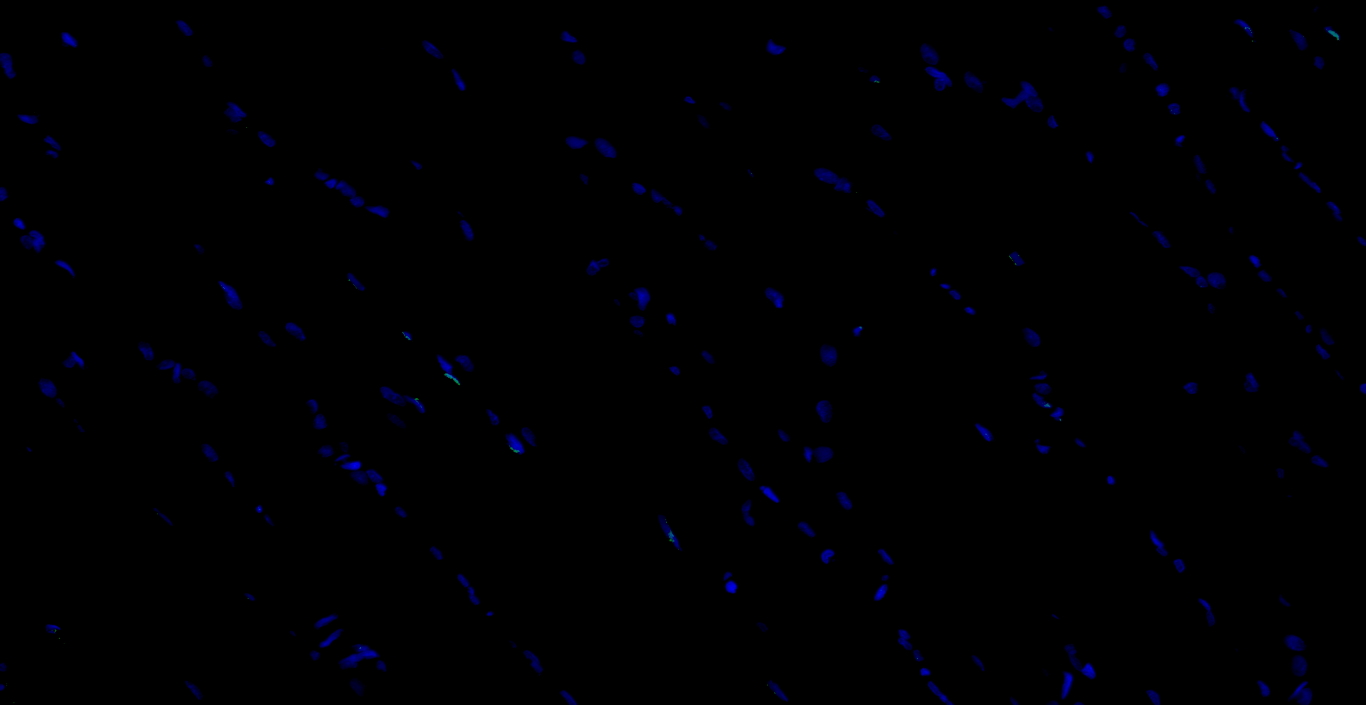

Supplement: Supplementary file 2 [file DataSheet4.ZIP › Supplemental materials 1/TUNEL/VNS/6-3 merge.jpg]

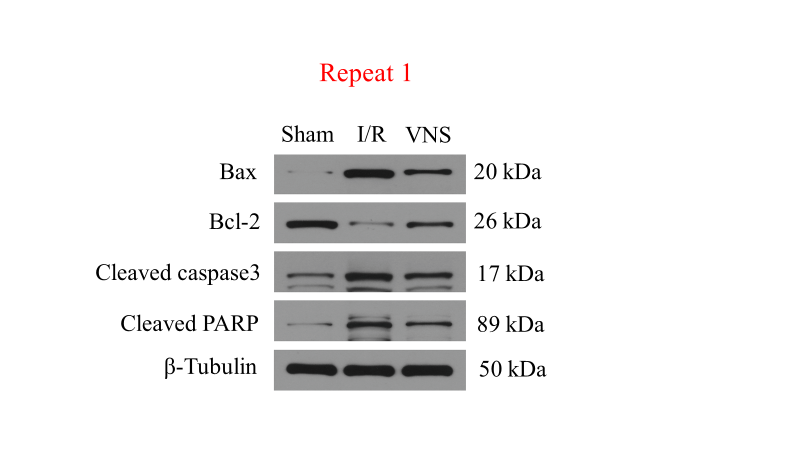

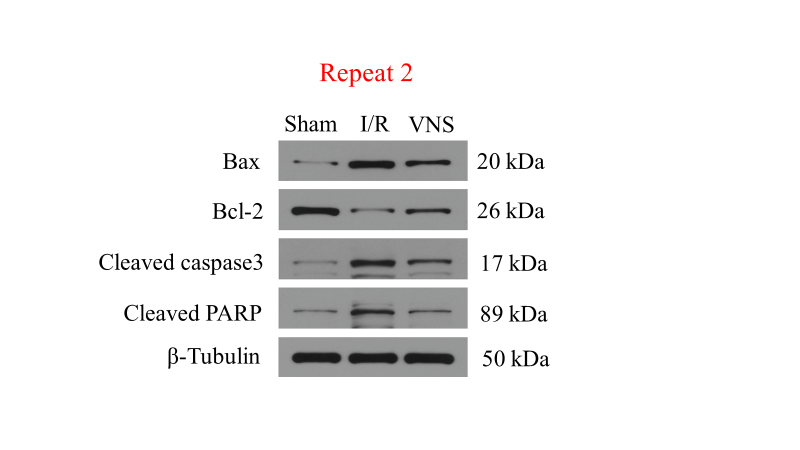

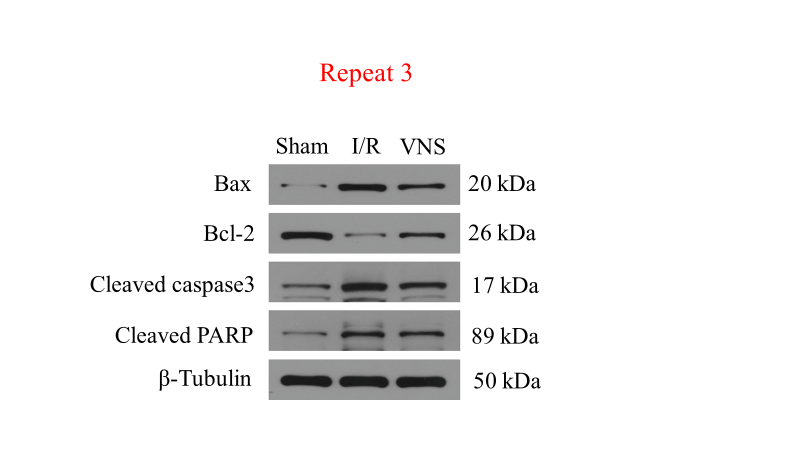

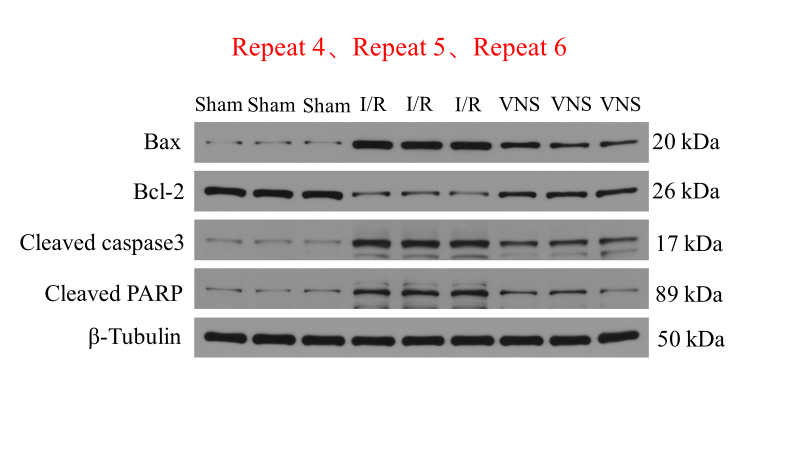

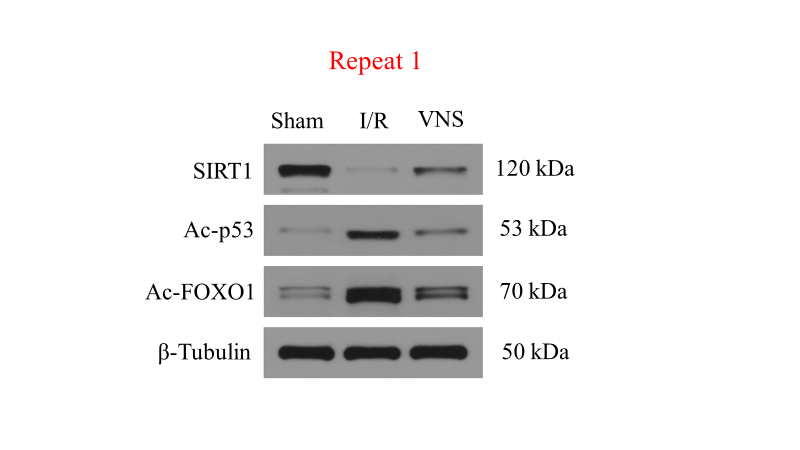

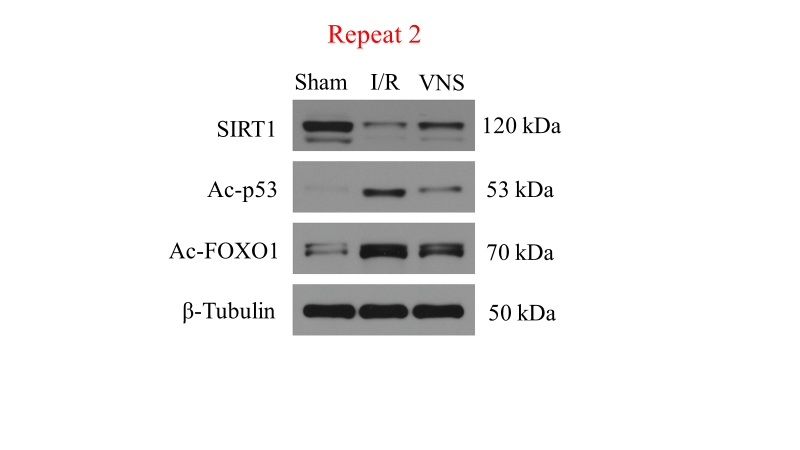

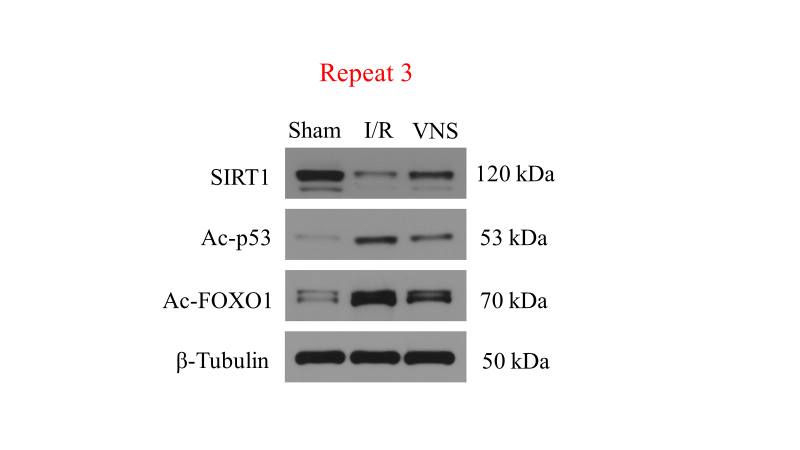

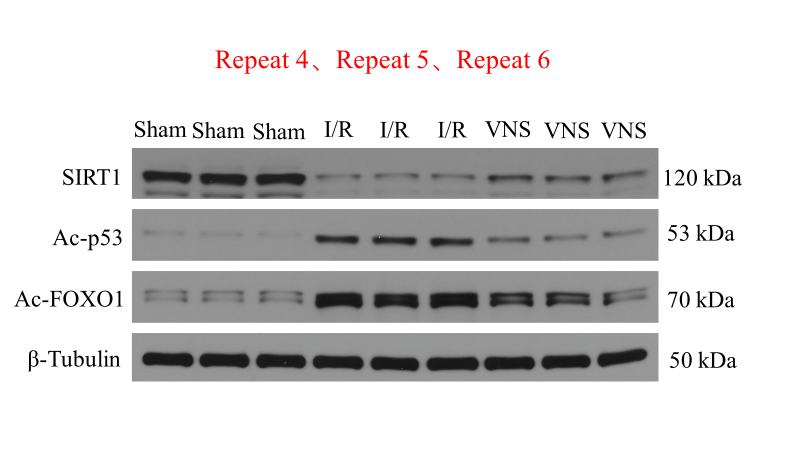

Supplement: Supplementary file 2 [file DataSheet4.ZIP › Supplemental materials 1/WB.docx]

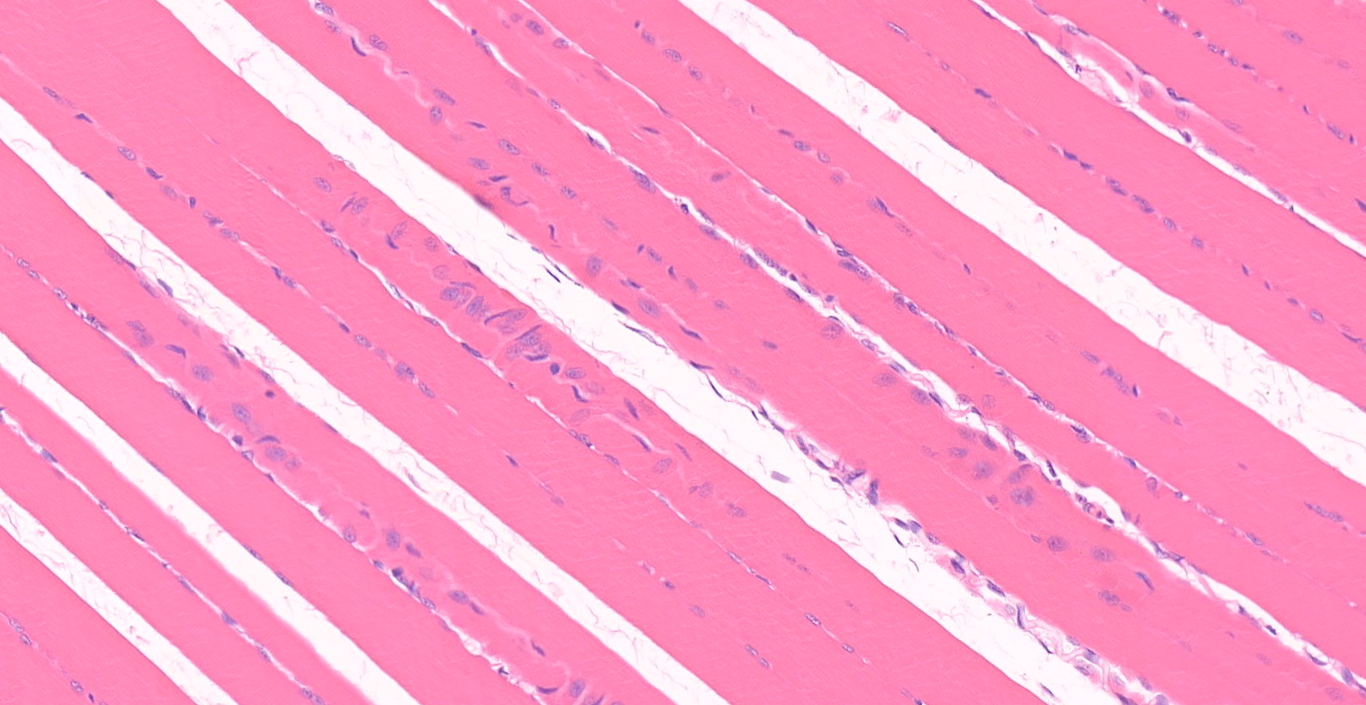

Supplement: Supplementary file 3 [file DataSheet1.ZIP › IR/1-1.jpg]

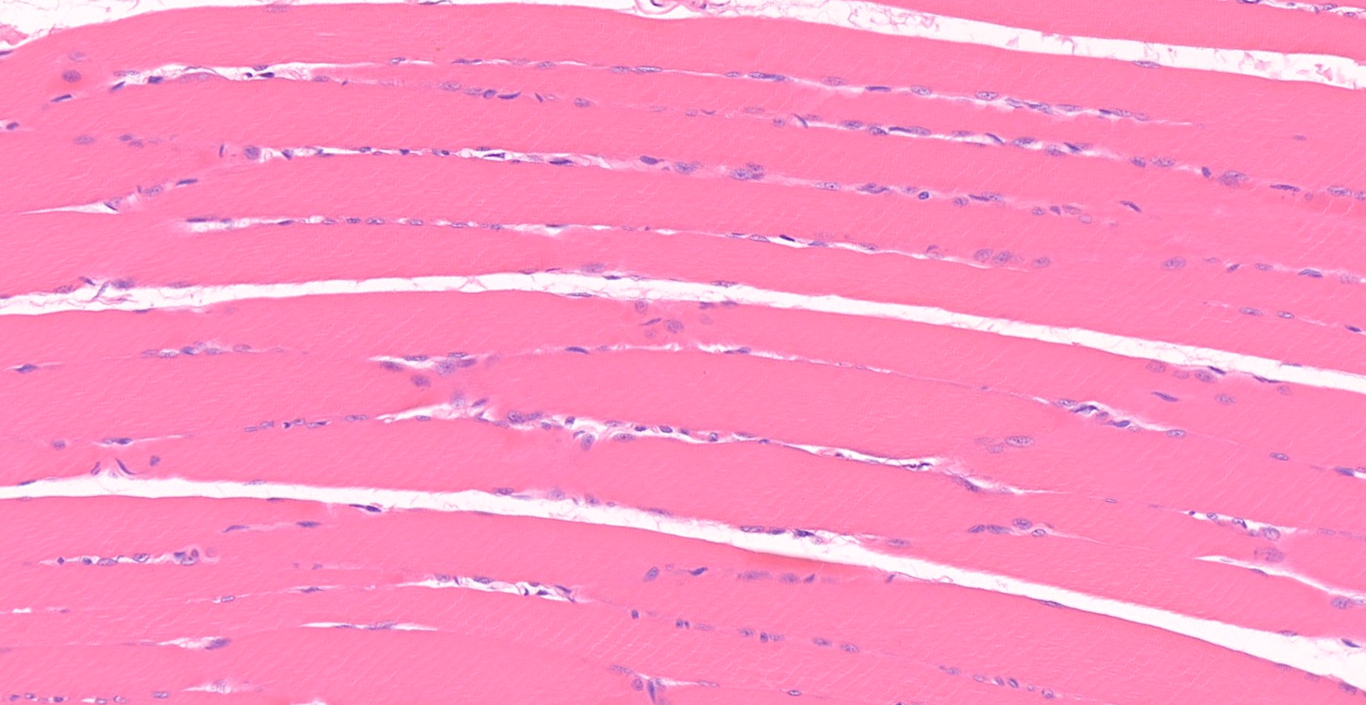

Supplement: Supplementary file 3 [file DataSheet1.ZIP › IR/1-2.jpg]

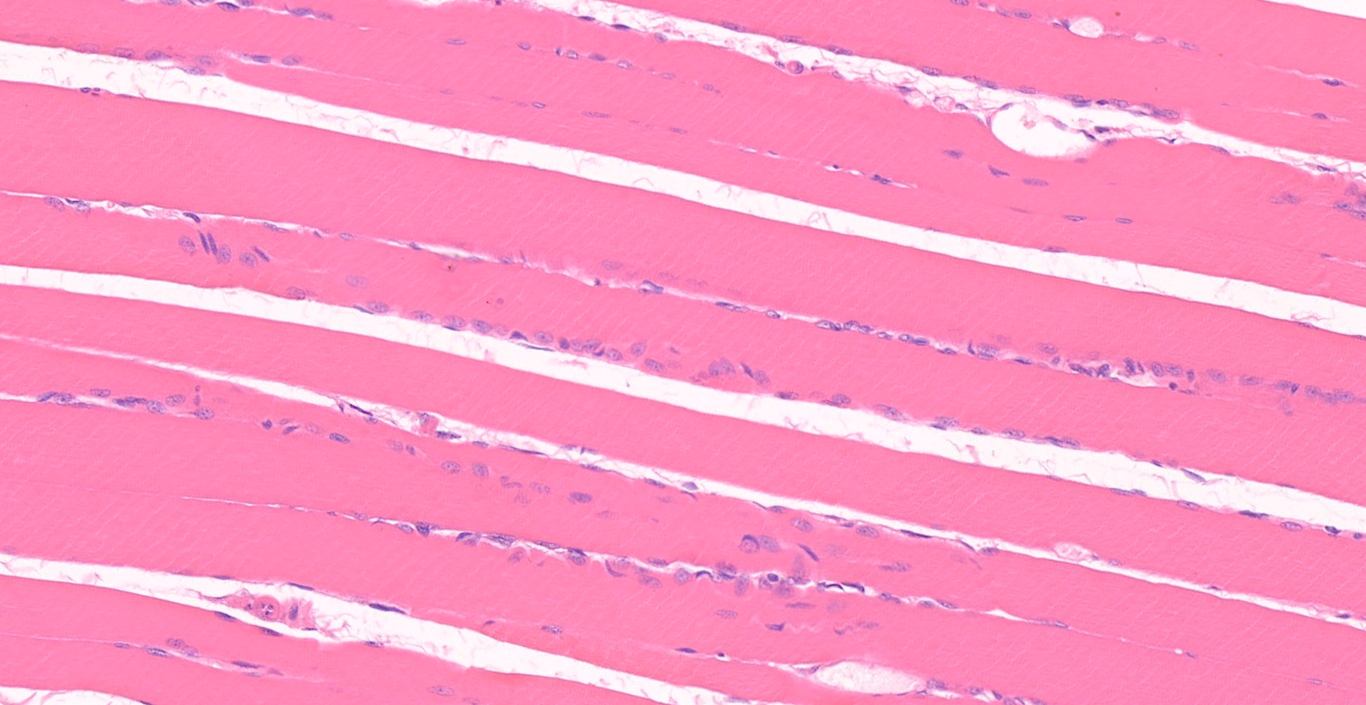

Supplement: Supplementary file 3 [file DataSheet1.ZIP › IR/1-3.jpg]

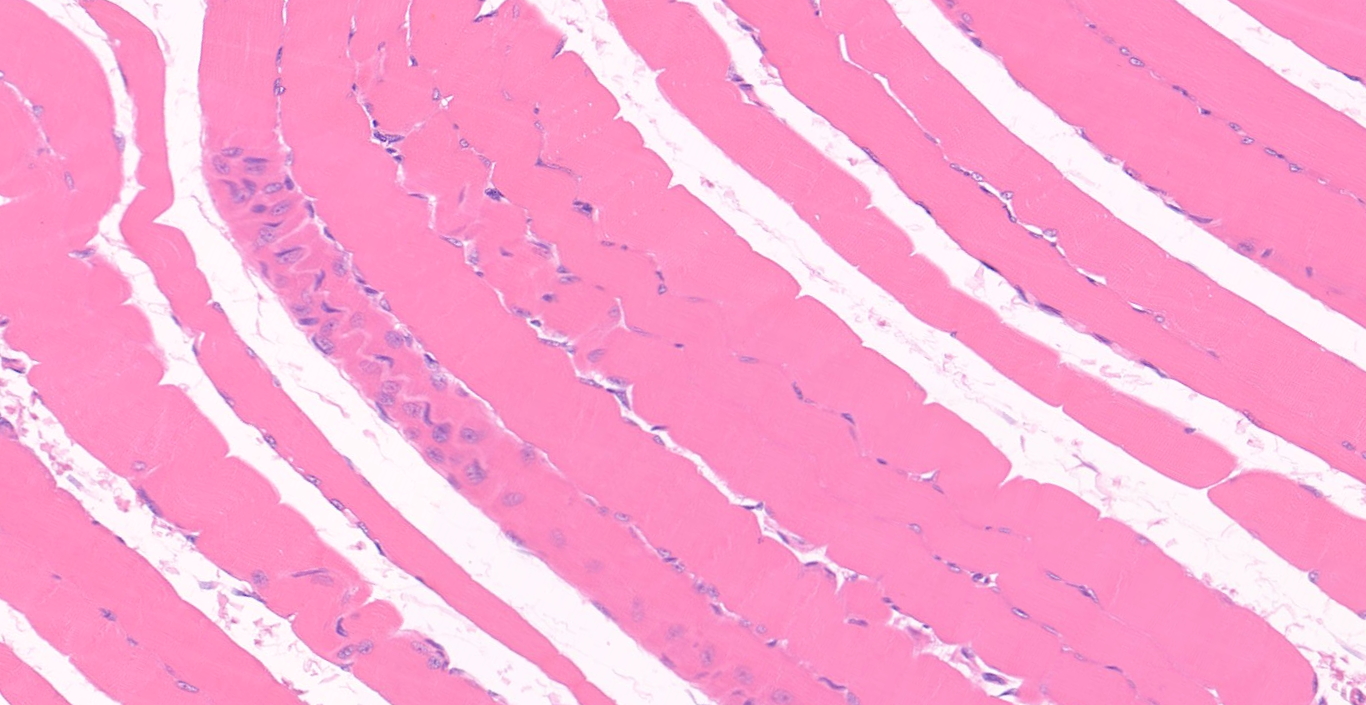

Supplement: Supplementary file 3 [file DataSheet1.ZIP › IR/2-1.jpg]

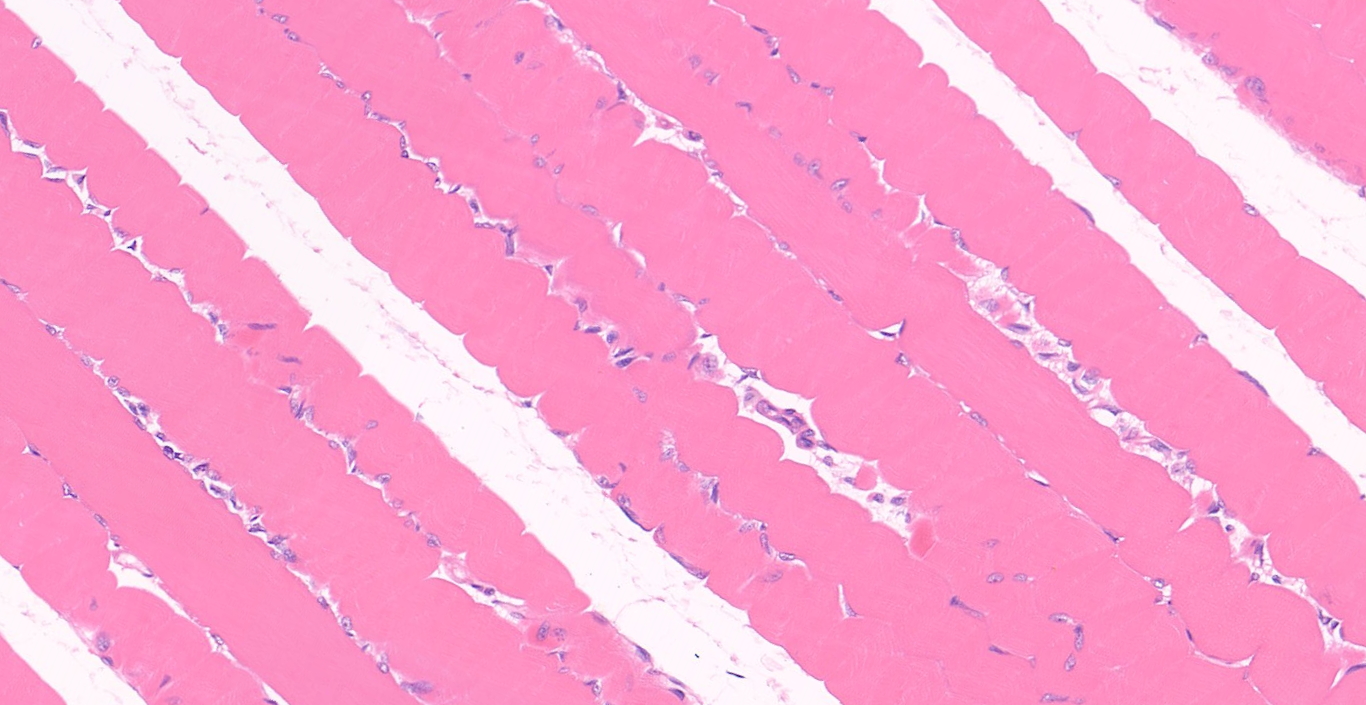

Supplement: Supplementary file 3 [file DataSheet1.ZIP › IR/2-2.jpg]

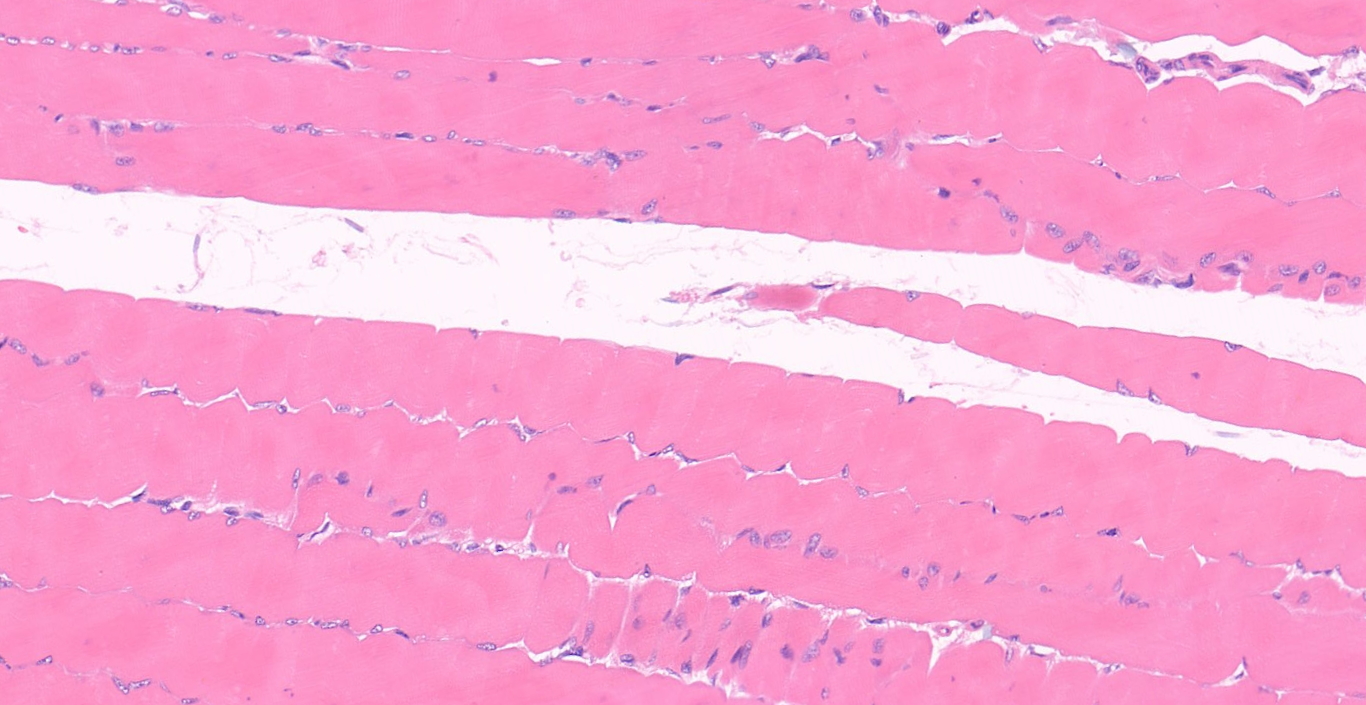

Supplement: Supplementary file 3 [file DataSheet1.ZIP › IR/2-3.jpg]

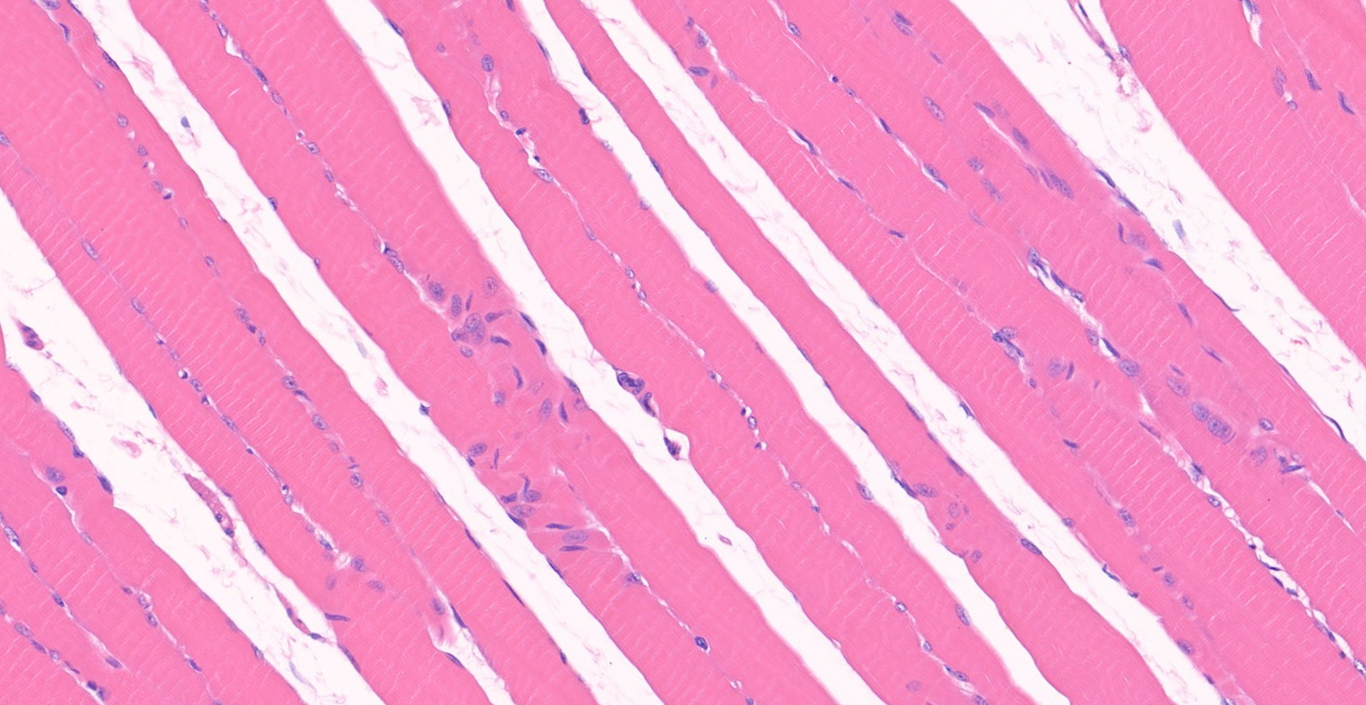

Supplement: Supplementary file 3 [file DataSheet1.ZIP › IR/3-1.jpg]

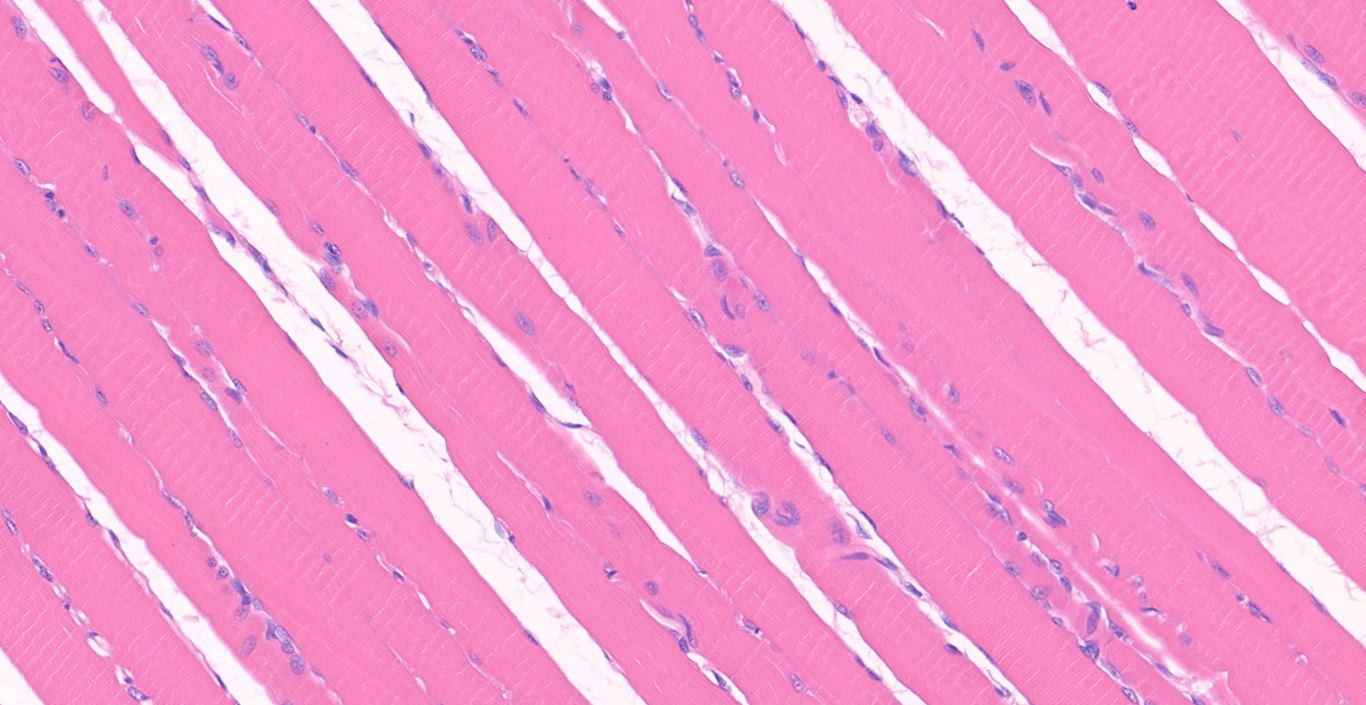

Supplement: Supplementary file 3 [file DataSheet1.ZIP › IR/3-2.jpg]

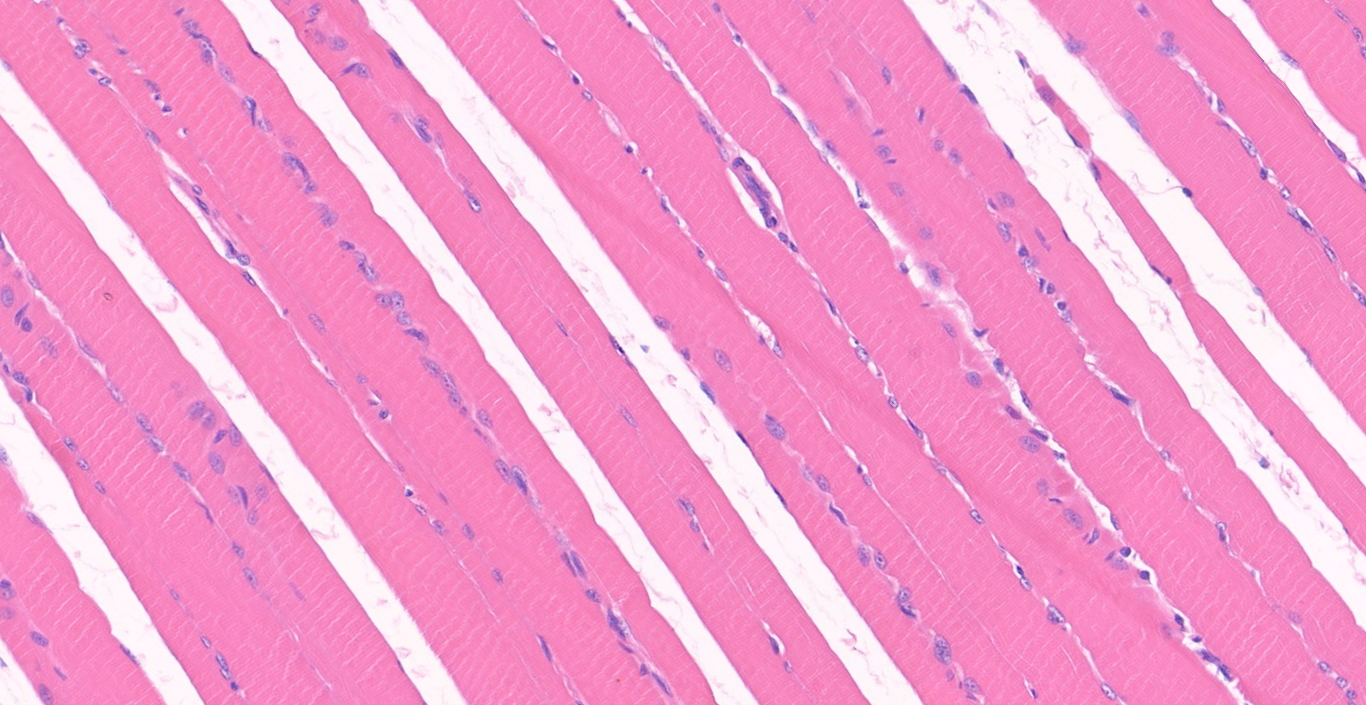

Supplement: Supplementary file 3 [file DataSheet1.ZIP › IR/3-3.jpg]

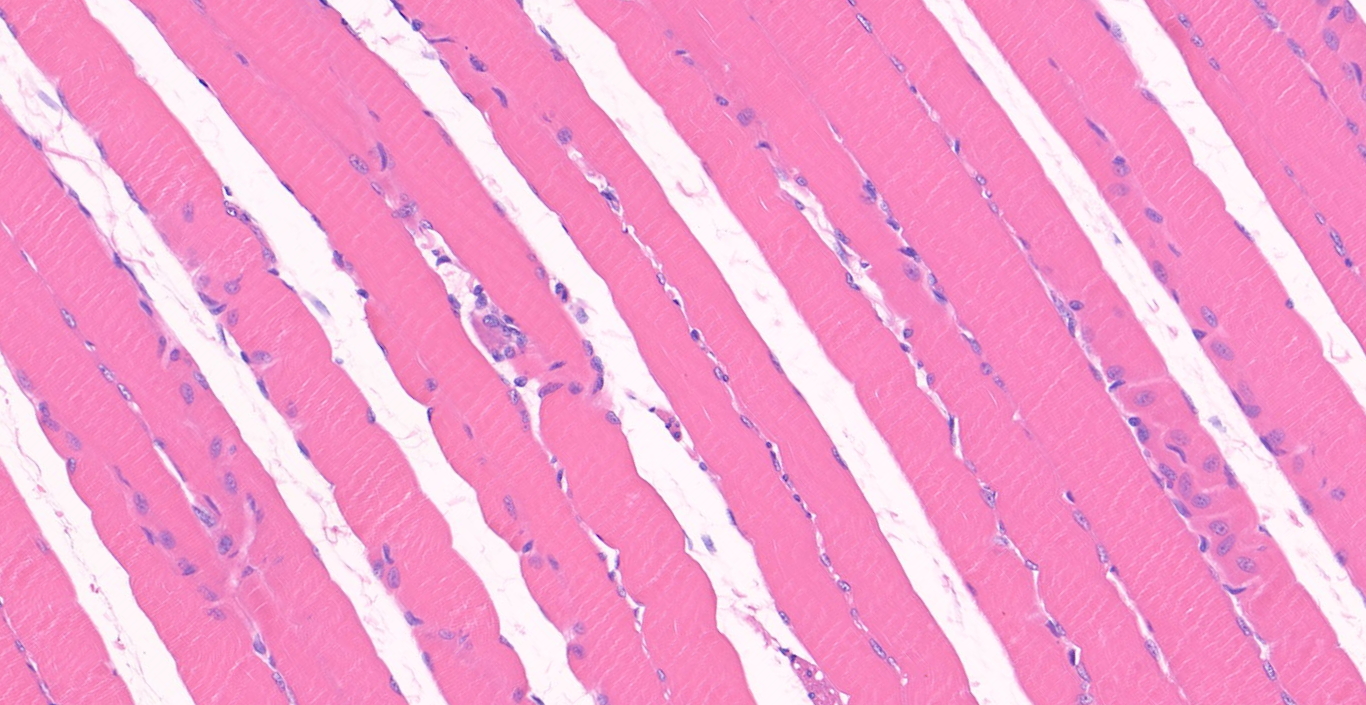

Supplement: Supplementary file 3 [file DataSheet1.ZIP › IR/4-1.jpg]

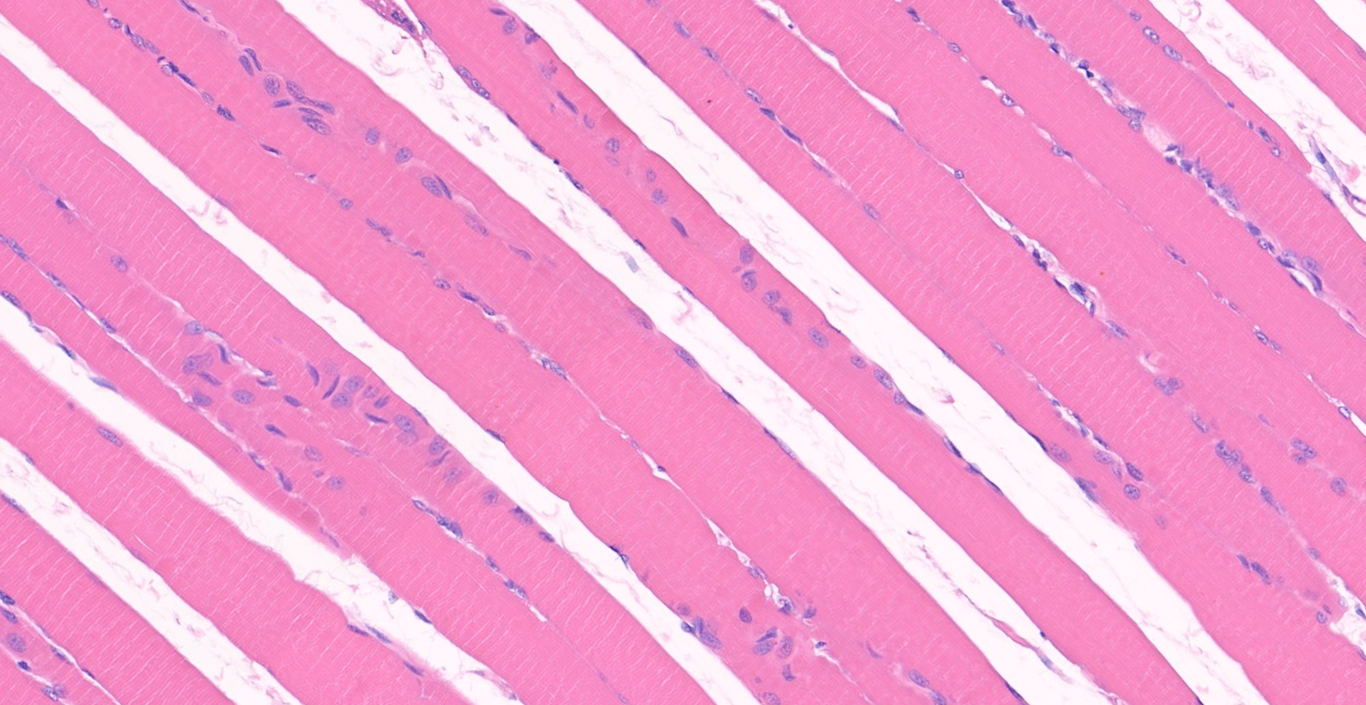

Supplement: Supplementary file 3 [file DataSheet1.ZIP › IR/4-2.jpg]

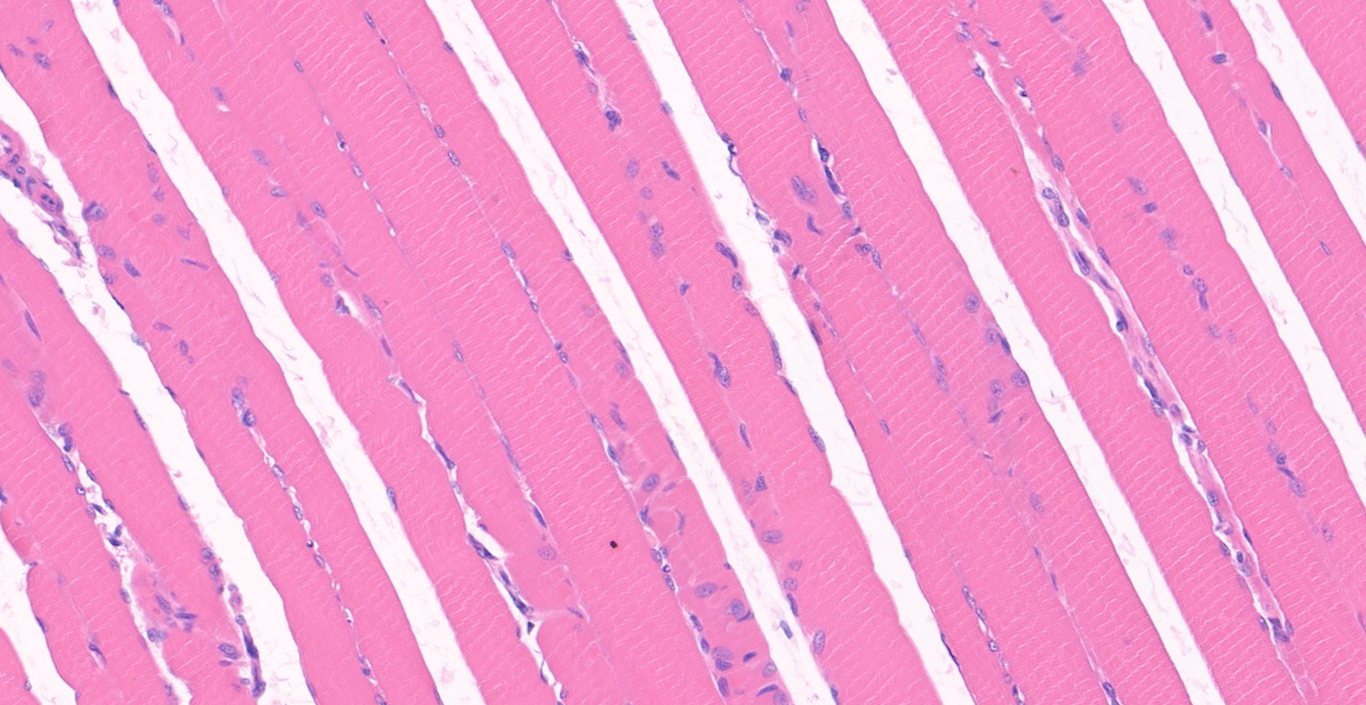

Supplement: Supplementary file 3 [file DataSheet1.ZIP › IR/4-3.jpg]

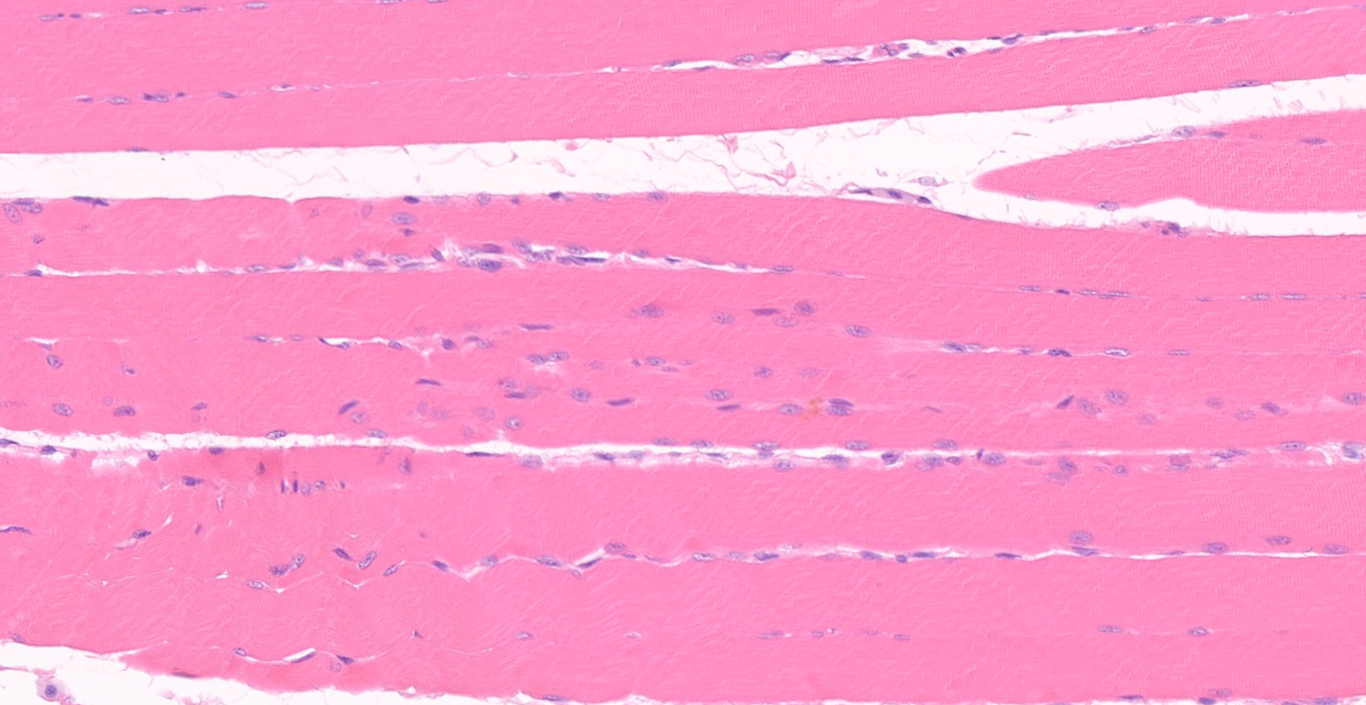

Supplement: Supplementary file 3 [file DataSheet1.ZIP › IR/5-1.jpg]

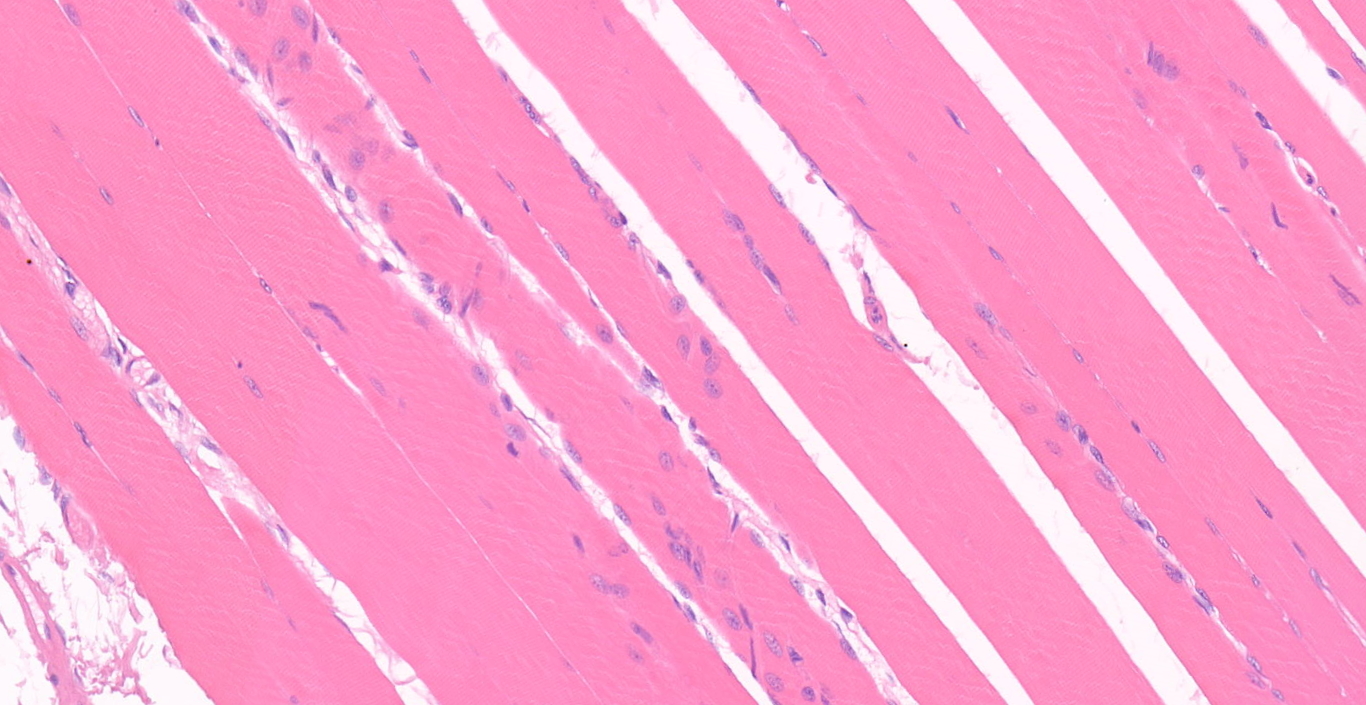

Supplement: Supplementary file 3 [file DataSheet1.ZIP › IR/5-2.jpg]

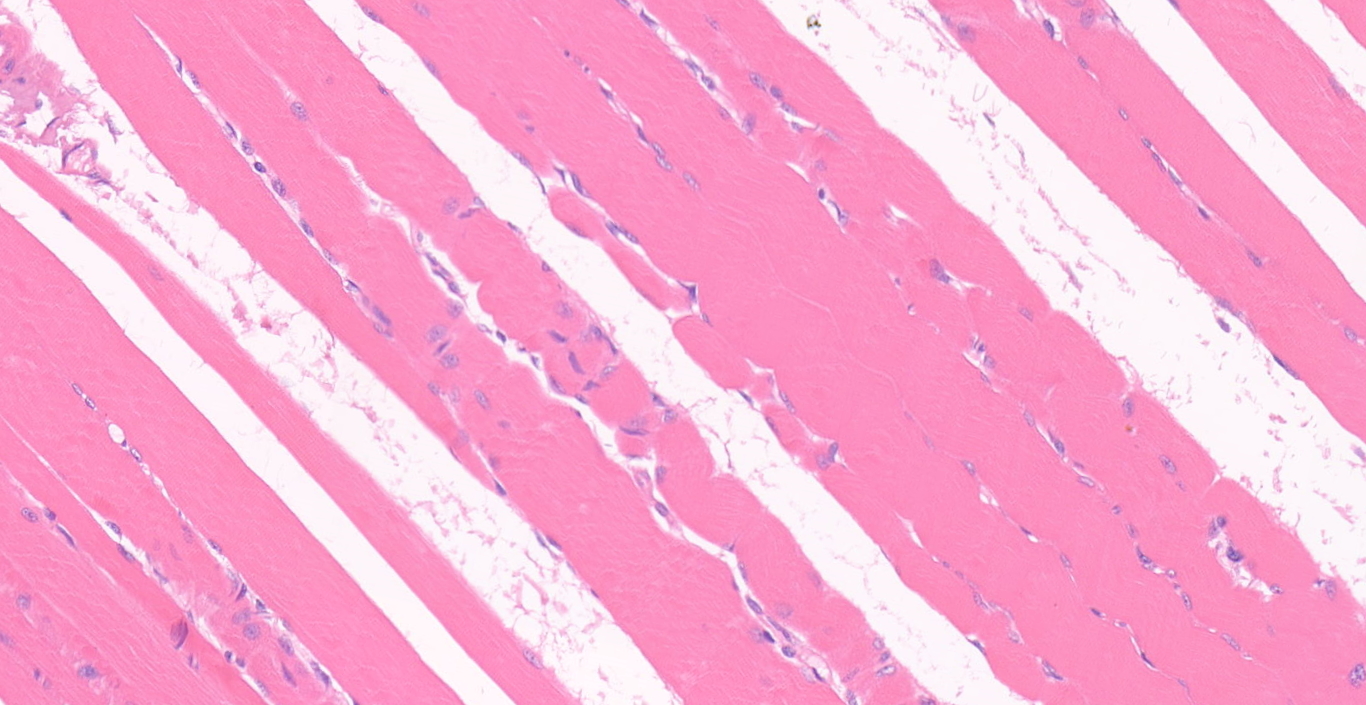

Supplement: Supplementary file 3 [file DataSheet1.ZIP › IR/5-3.jpg]

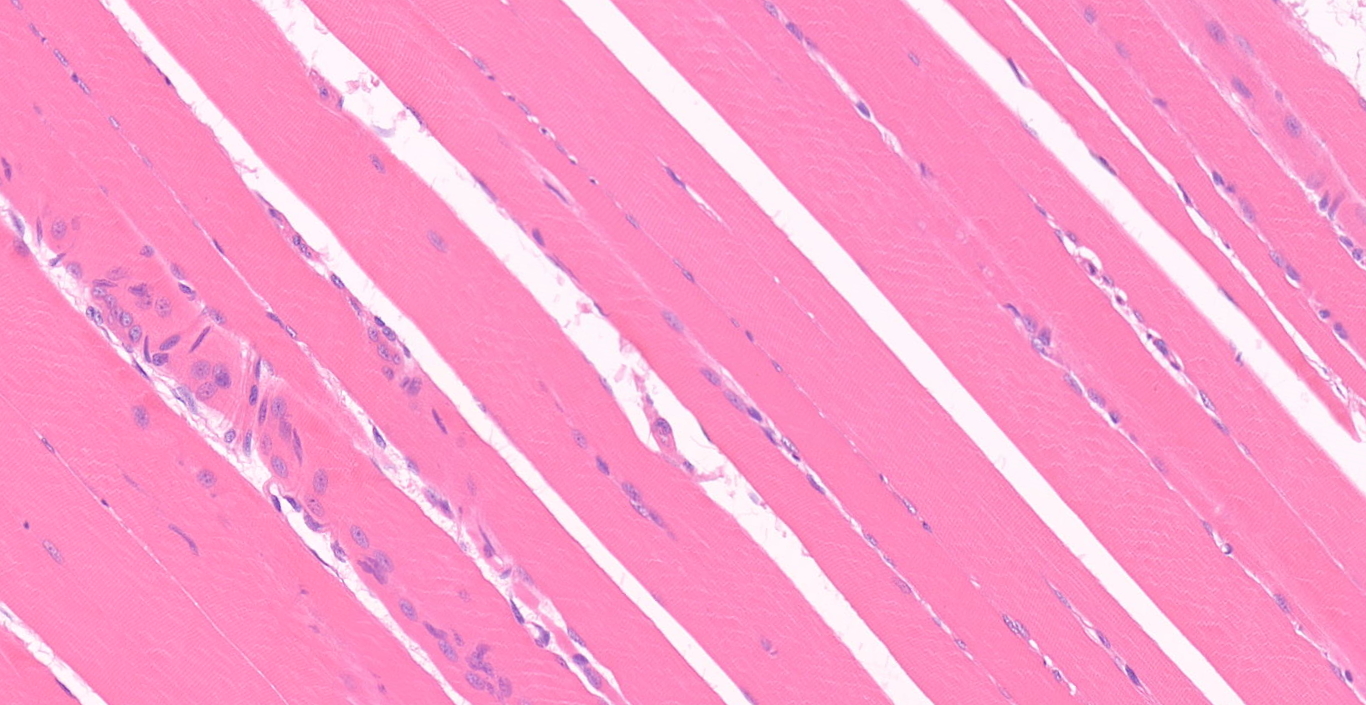

Supplement: Supplementary file 3 [file DataSheet1.ZIP › IR/6-1.jpg]

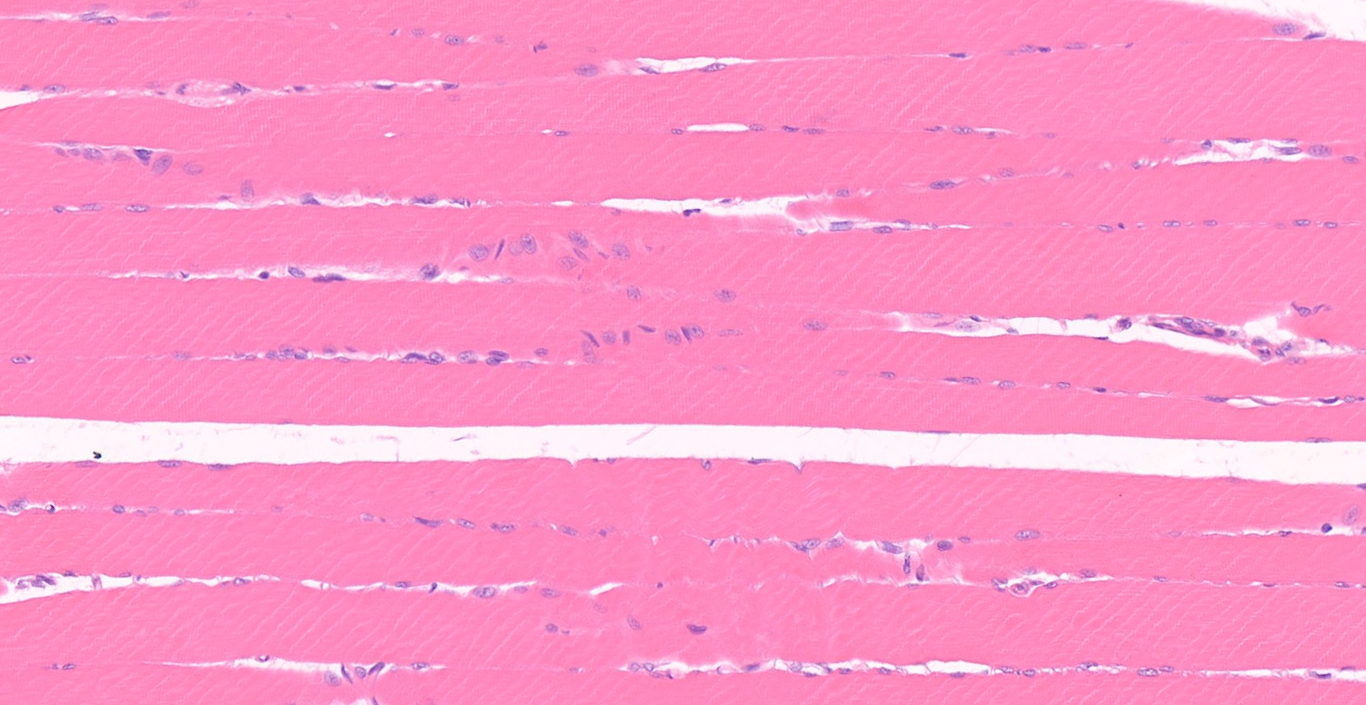

Supplement: Supplementary file 3 [file DataSheet1.ZIP › IR/6-2.jpg]

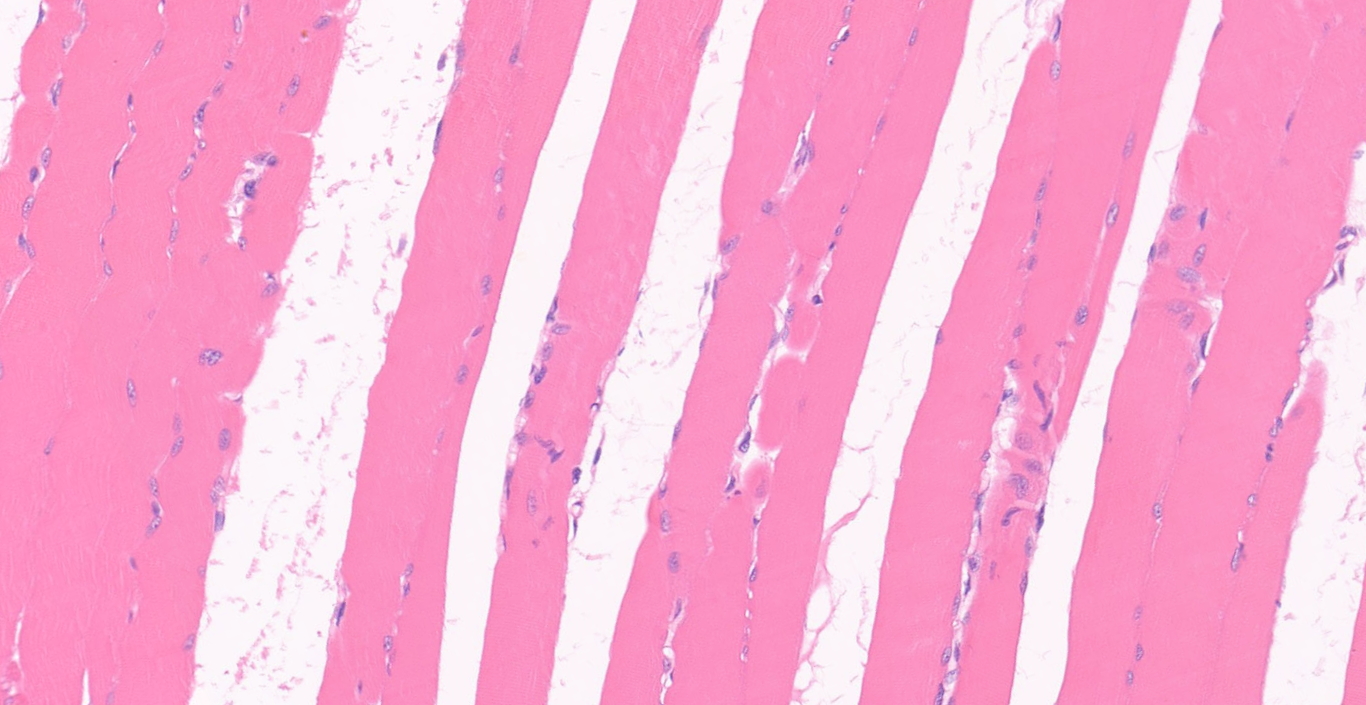

Supplement: Supplementary file 3 [file DataSheet1.ZIP › IR/6-3.jpg]

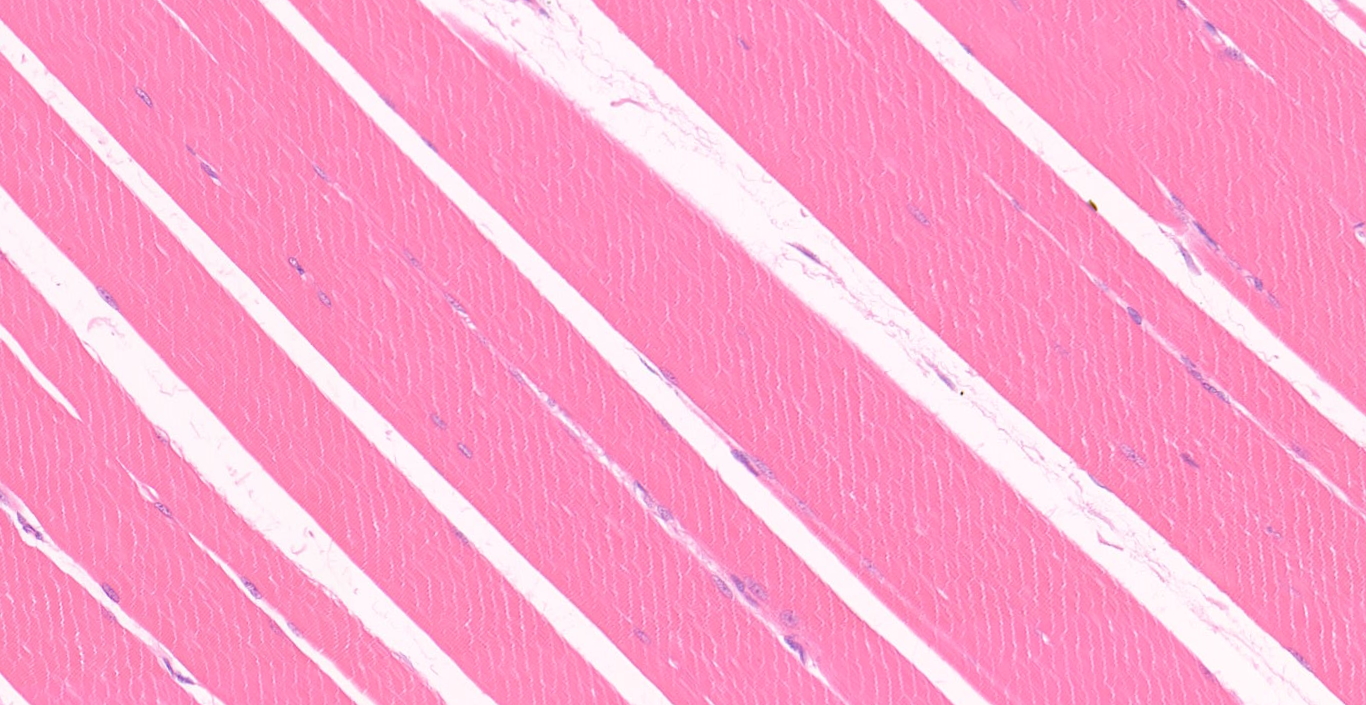

Supplement: Supplementary file 4 [file DataSheet2.ZIP › Sham/1-1.jpg]

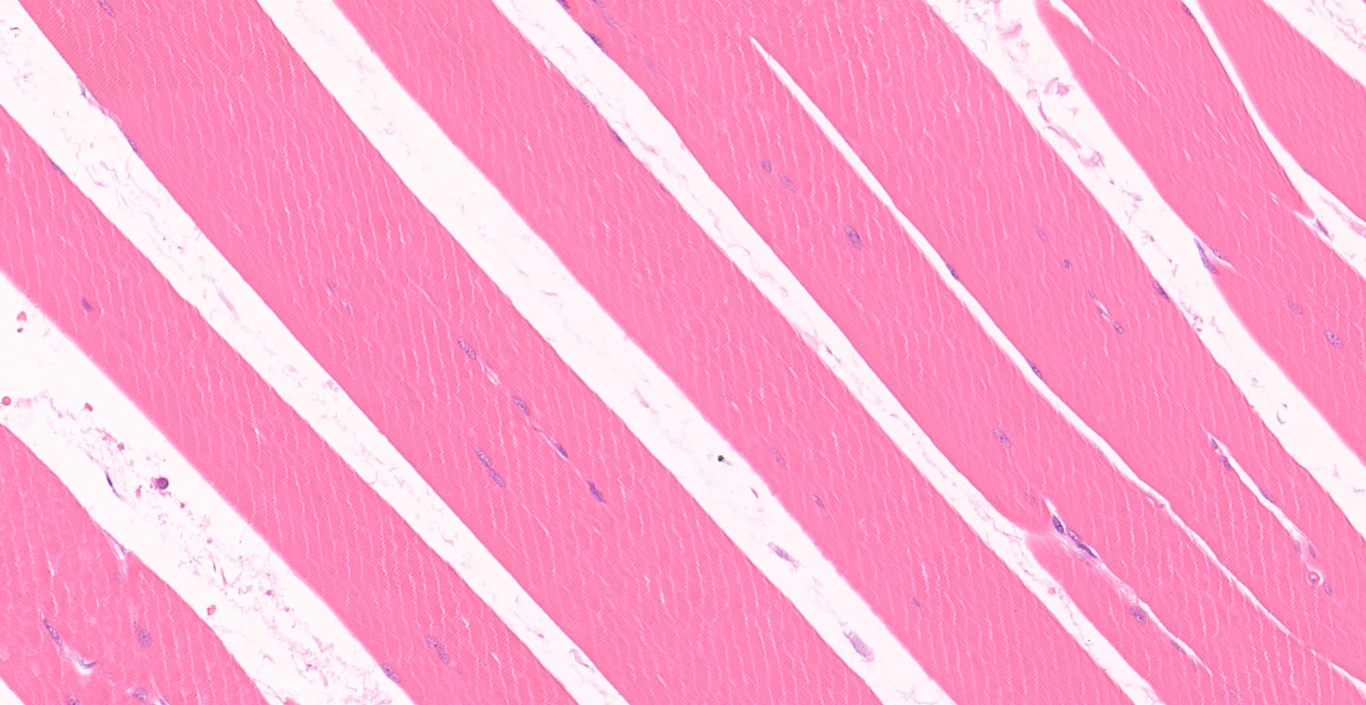

Supplement: Supplementary file 4 [file DataSheet2.ZIP › Sham/1-2.jpg]

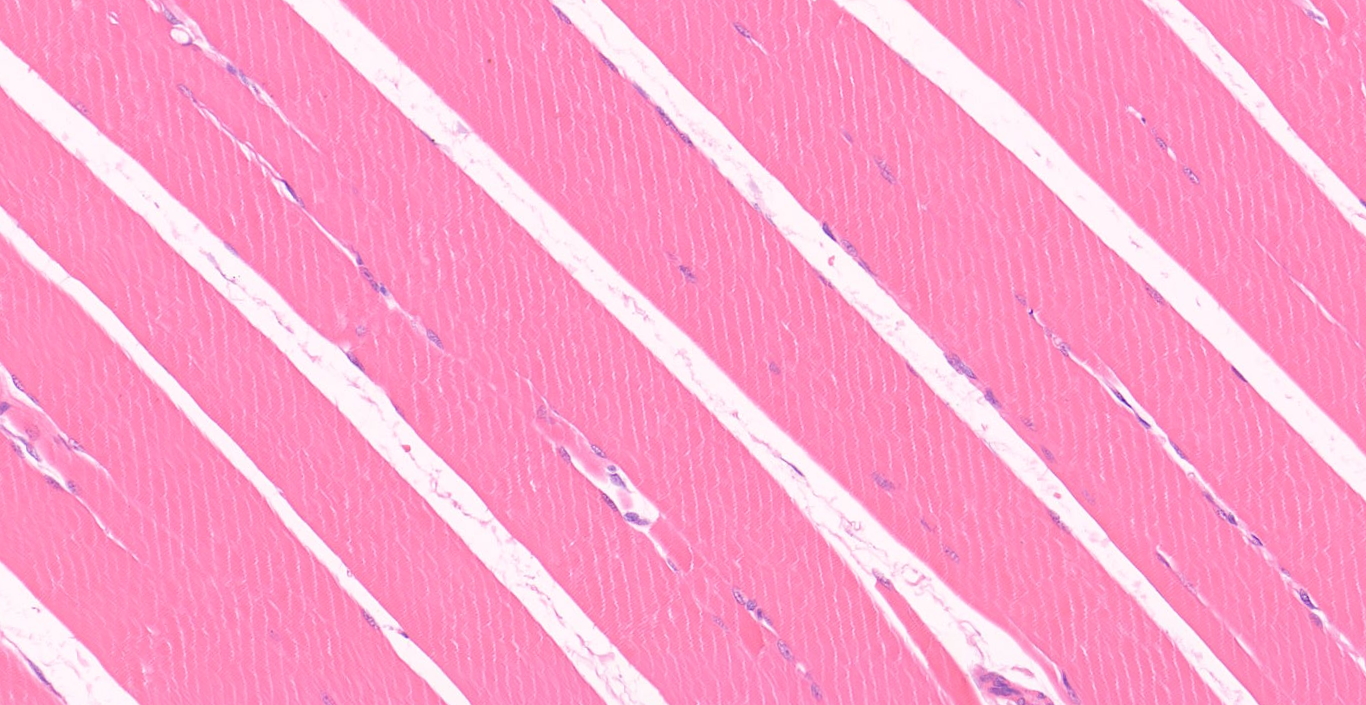

Supplement: Supplementary file 4 [file DataSheet2.ZIP › Sham/1-3.jpg]

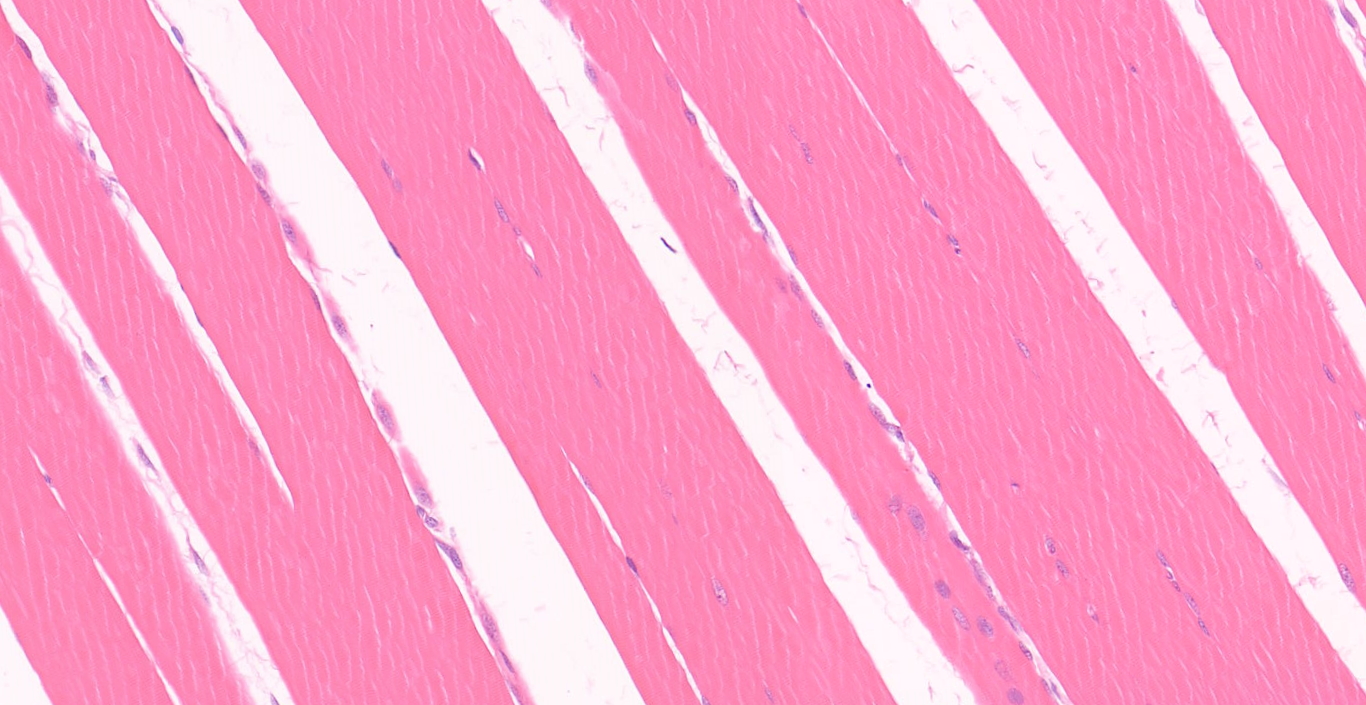

Supplement: Supplementary file 4 [file DataSheet2.ZIP › Sham/2-1.jpg]

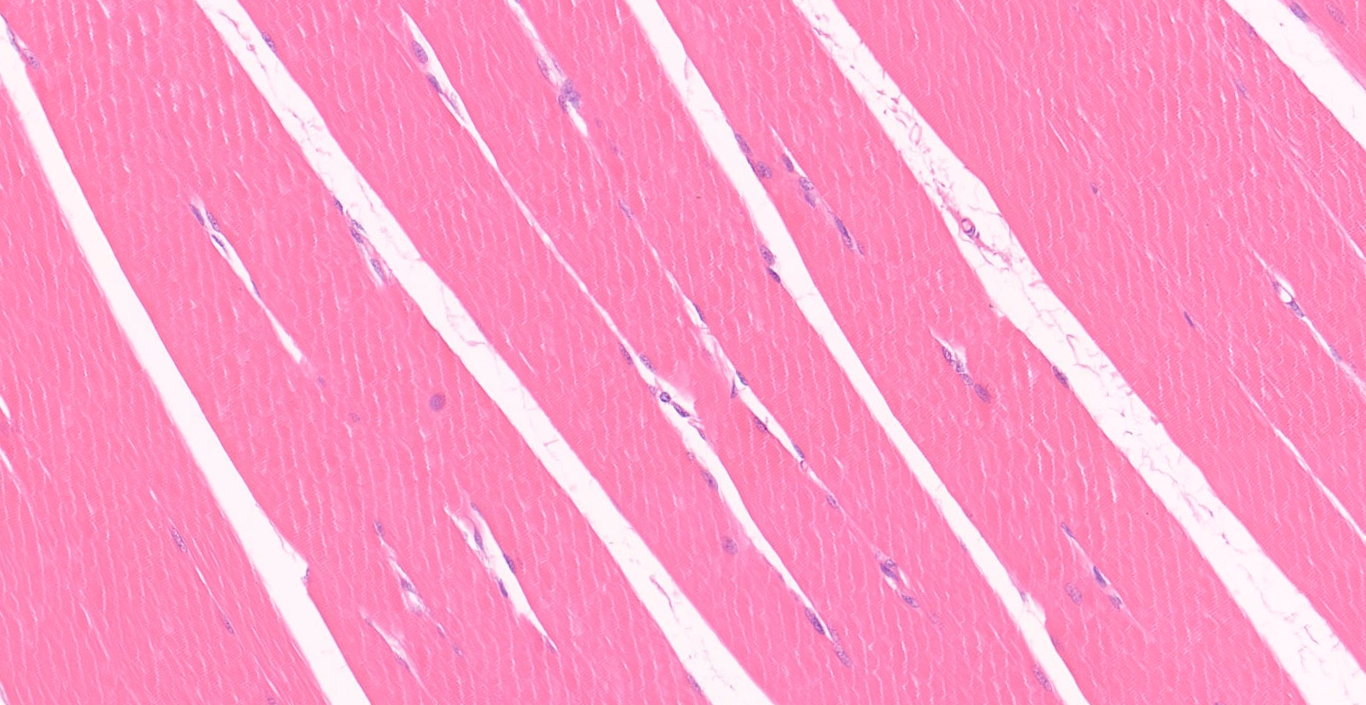

Supplement: Supplementary file 4 [file DataSheet2.ZIP › Sham/2-2.jpg]

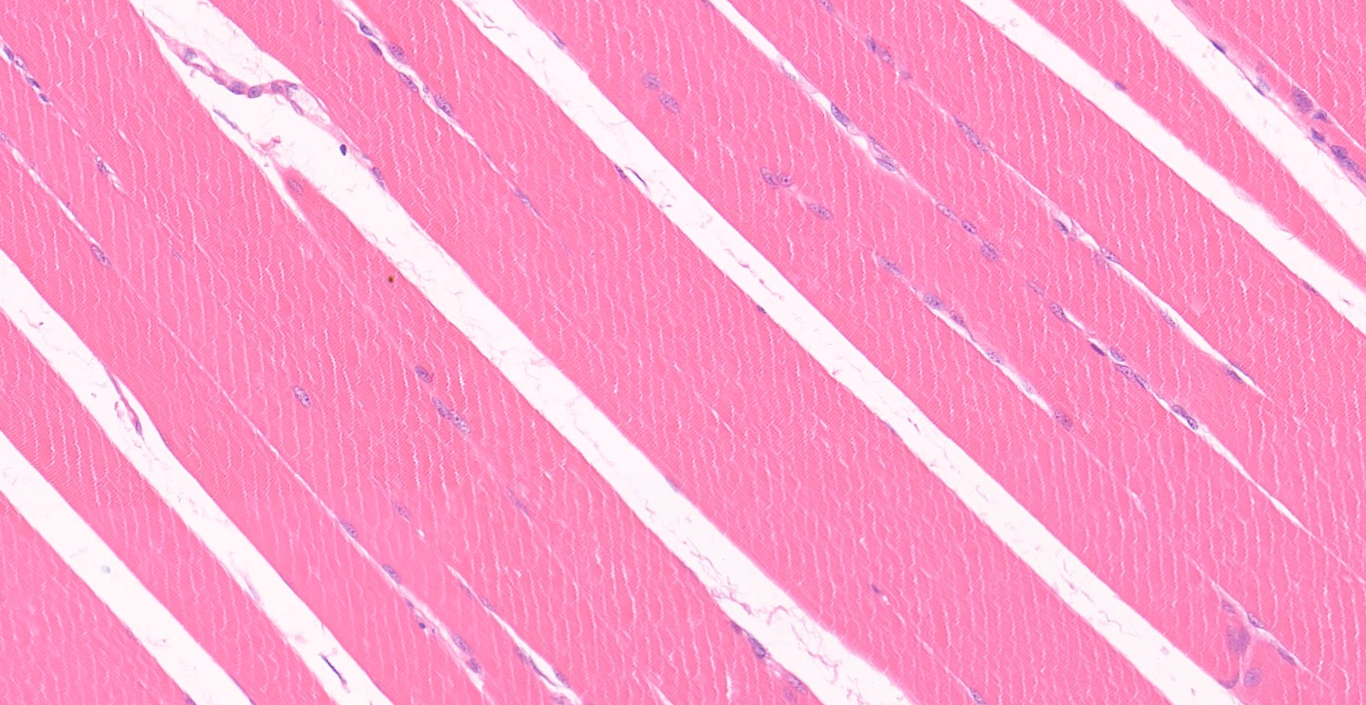

Supplement: Supplementary file 4 [file DataSheet2.ZIP › Sham/2-3.jpg]
