# Supplementary figures and images for: Vagus Nerve Stimulation Attenuates Acute Skeletal Muscle Injury Induced by Hepatic Ischemia/Reperfusion Injury in Rats (part 3 of 3)
Source: Front Pharmacol. 2022 Jan 3;12:756997. doi: 10.3389/fphar.2021.756997 (PMC8762262; doi:10.3389/fphar.2021.756997)

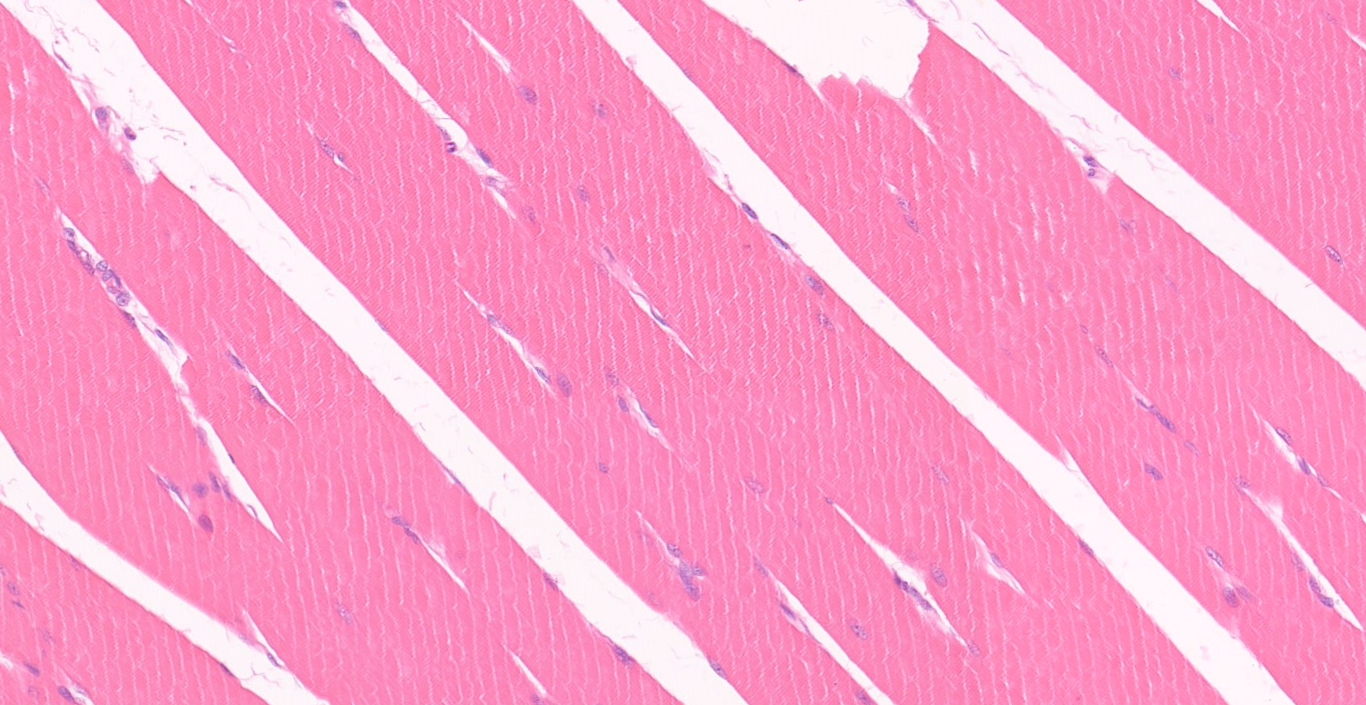

Supplement: Supplementary file 4 [file DataSheet2.ZIP › Sham/3-1.jpg]

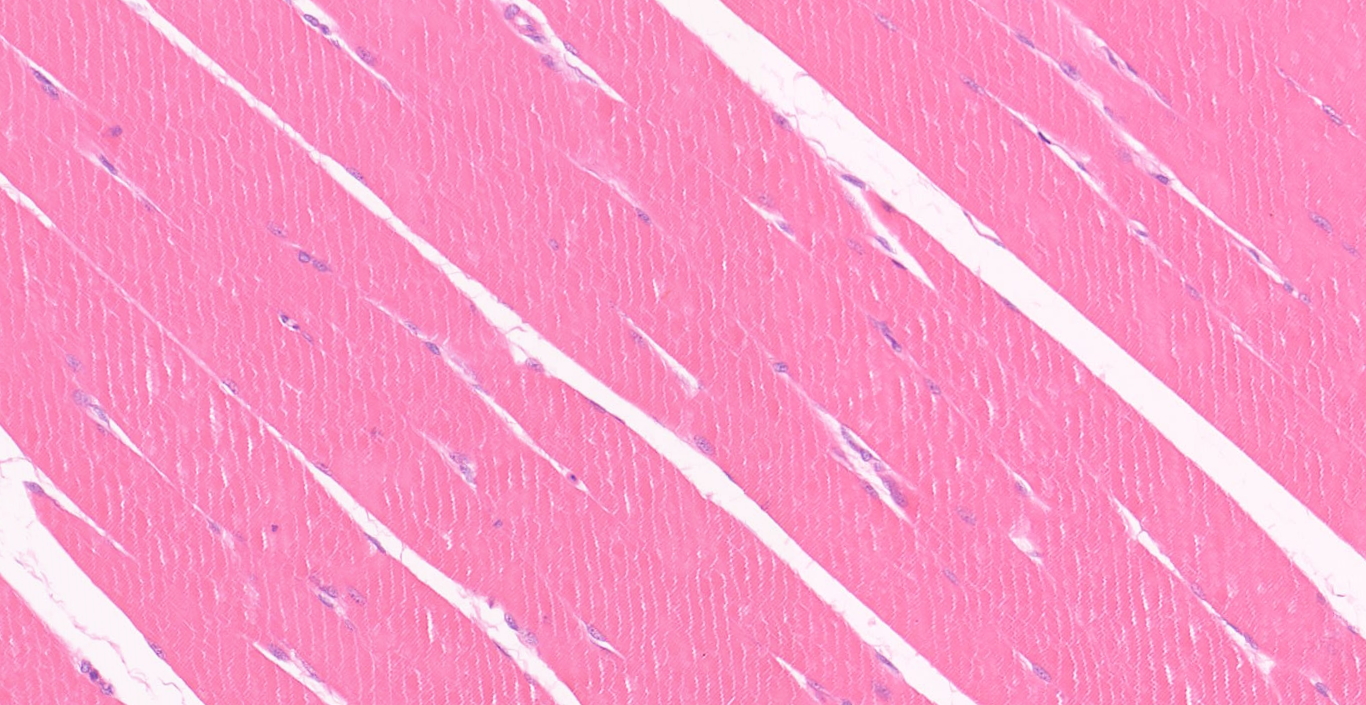

Supplement: Supplementary file 4 [file DataSheet2.ZIP › Sham/3-2.jpg]

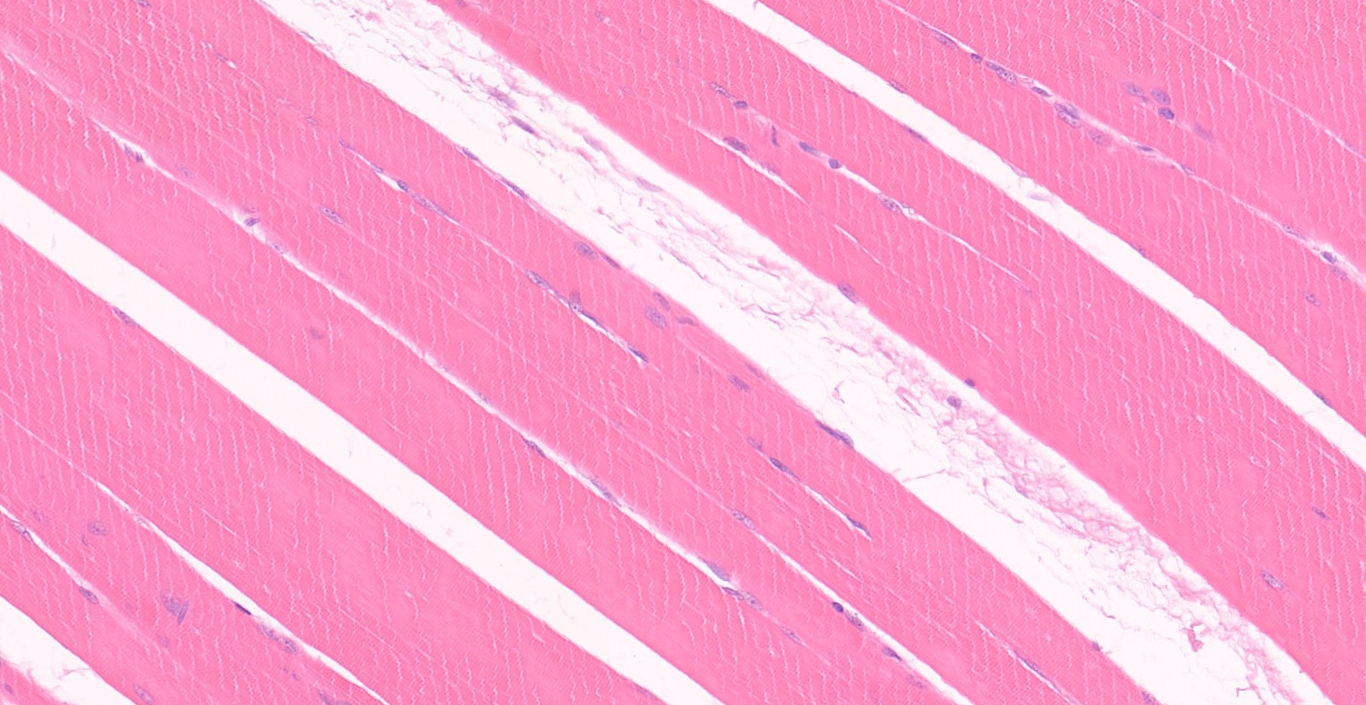

Supplement: Supplementary file 4 [file DataSheet2.ZIP › Sham/3-3.jpg]

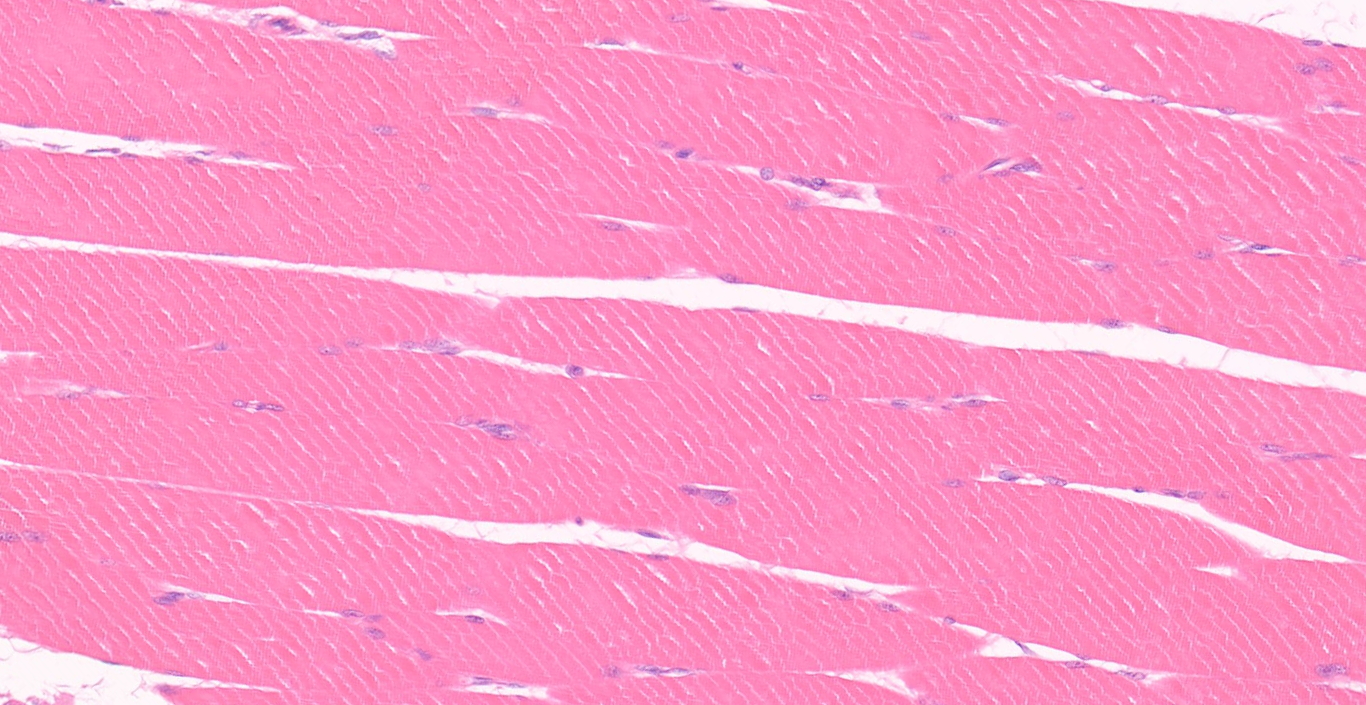

Supplement: Supplementary file 4 [file DataSheet2.ZIP › Sham/4-1.jpg]

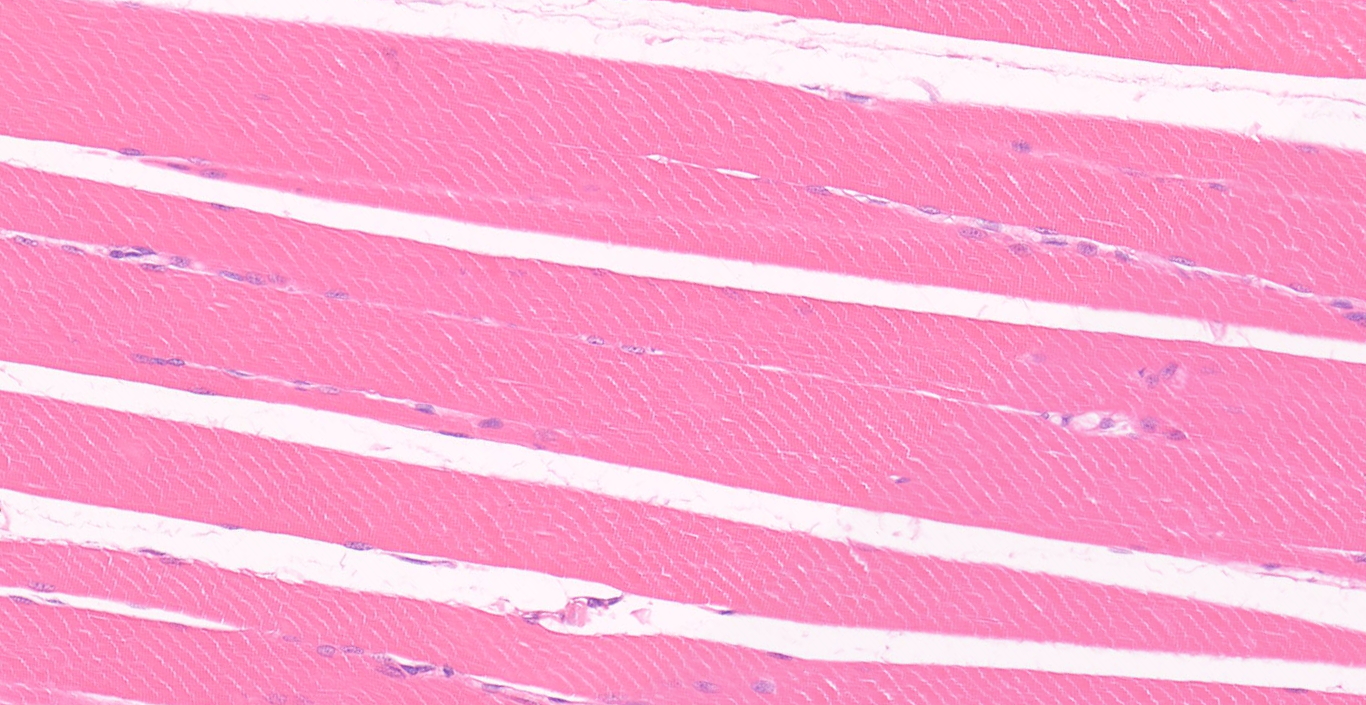

Supplement: Supplementary file 4 [file DataSheet2.ZIP › Sham/4-2.jpg]

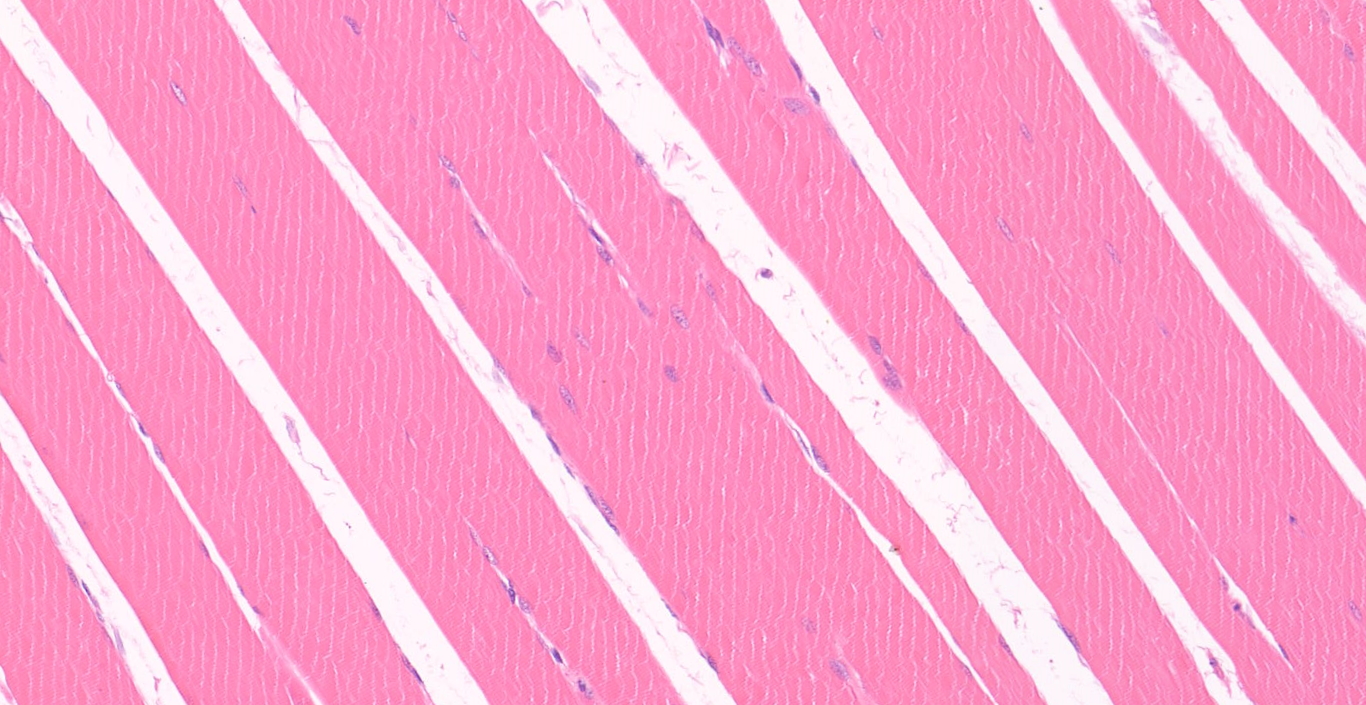

Supplement: Supplementary file 4 [file DataSheet2.ZIP › Sham/4-3.jpg]

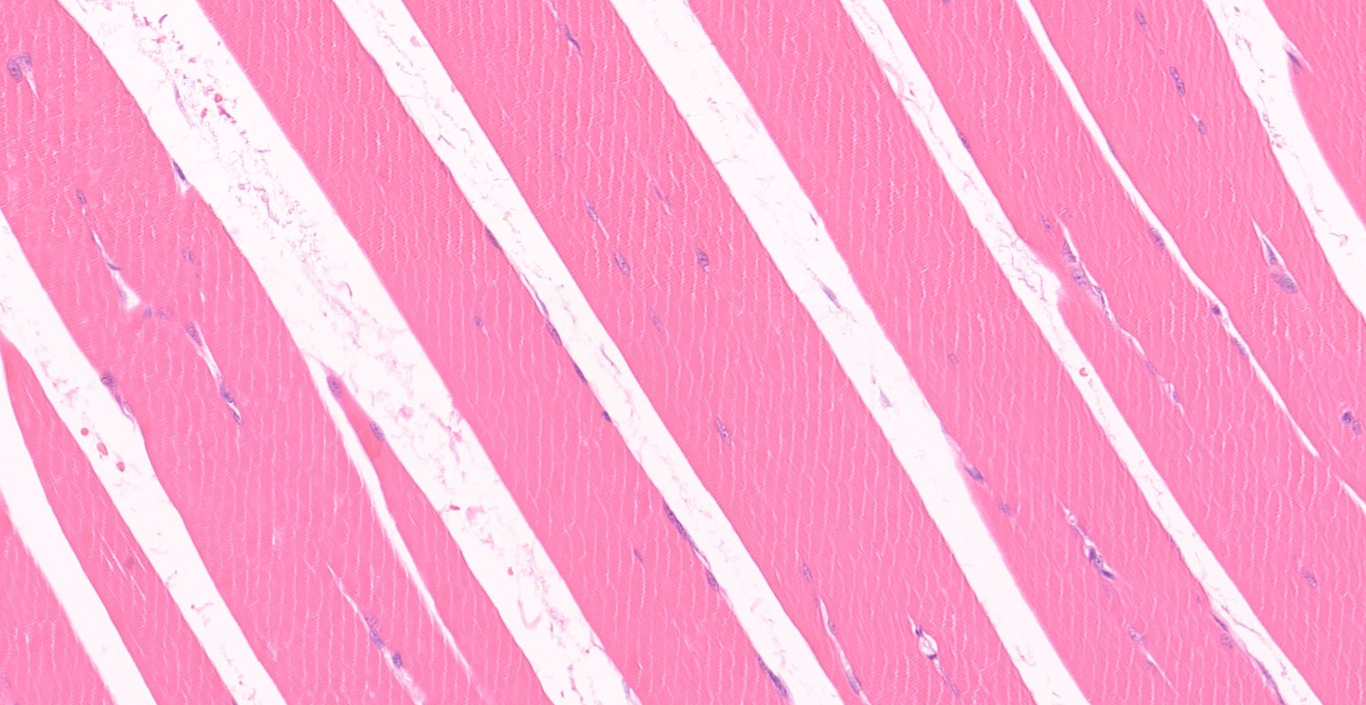

Supplement: Supplementary file 4 [file DataSheet2.ZIP › Sham/5-1.jpg]

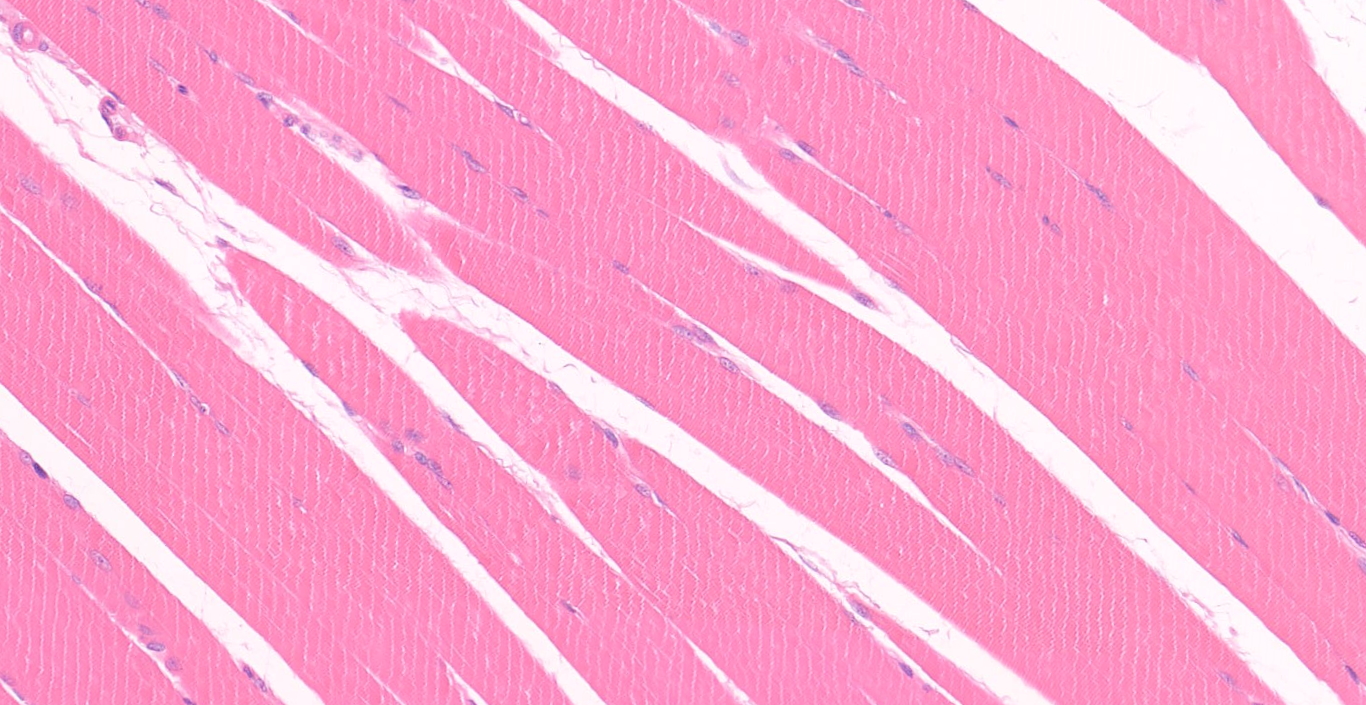

Supplement: Supplementary file 4 [file DataSheet2.ZIP › Sham/5-2.jpg]

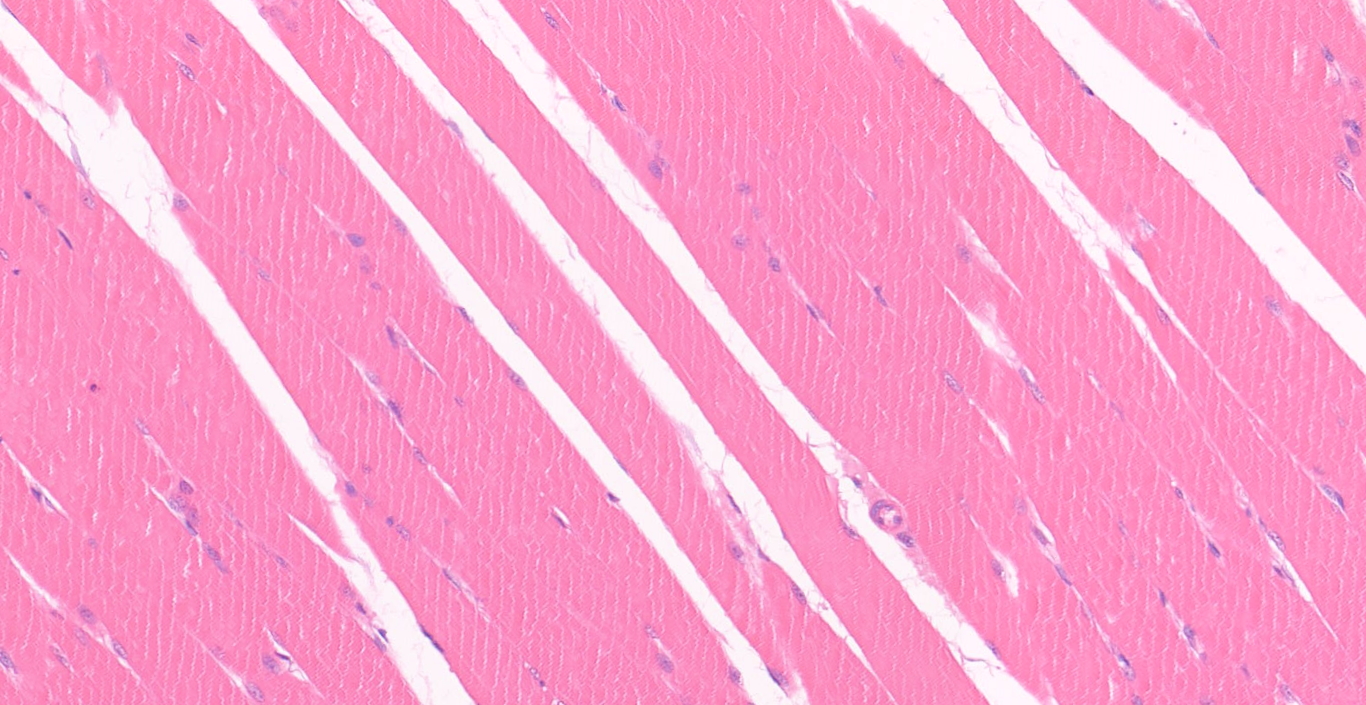

Supplement: Supplementary file 4 [file DataSheet2.ZIP › Sham/5-3.jpg]

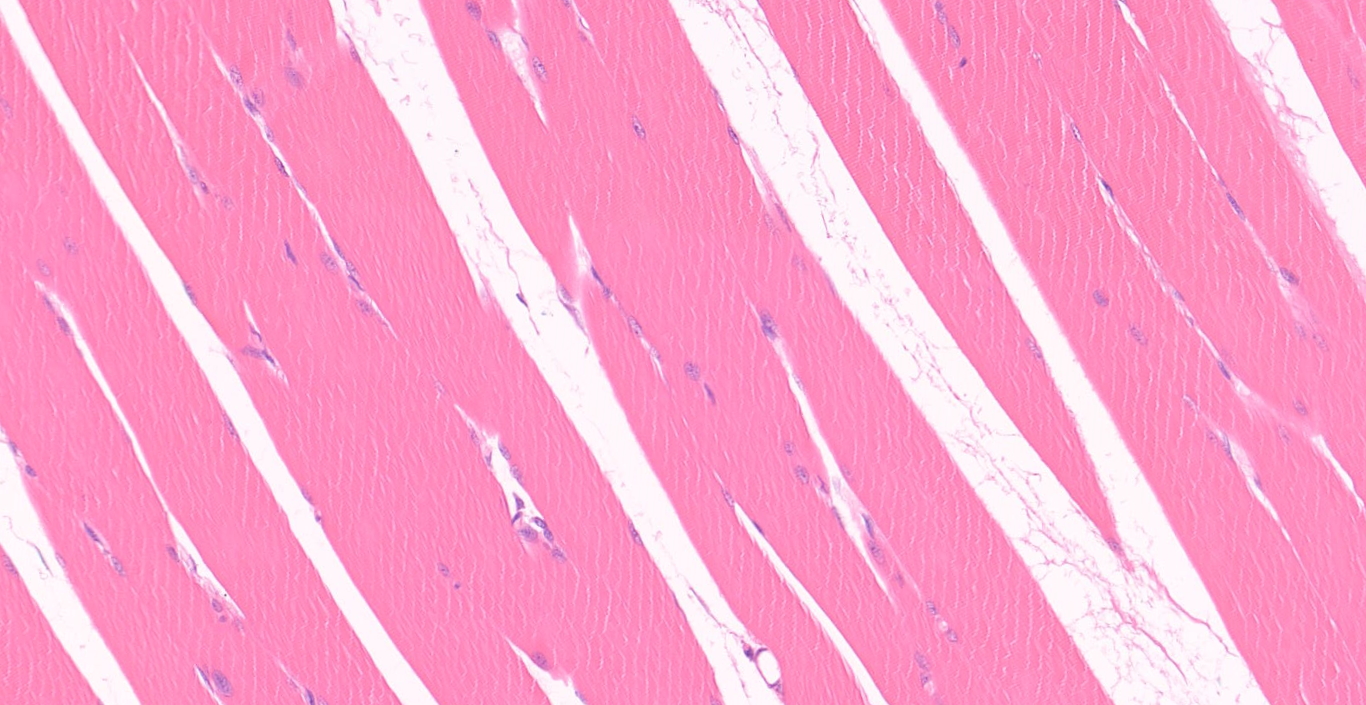

Supplement: Supplementary file 4 [file DataSheet2.ZIP › Sham/6-1.jpg]

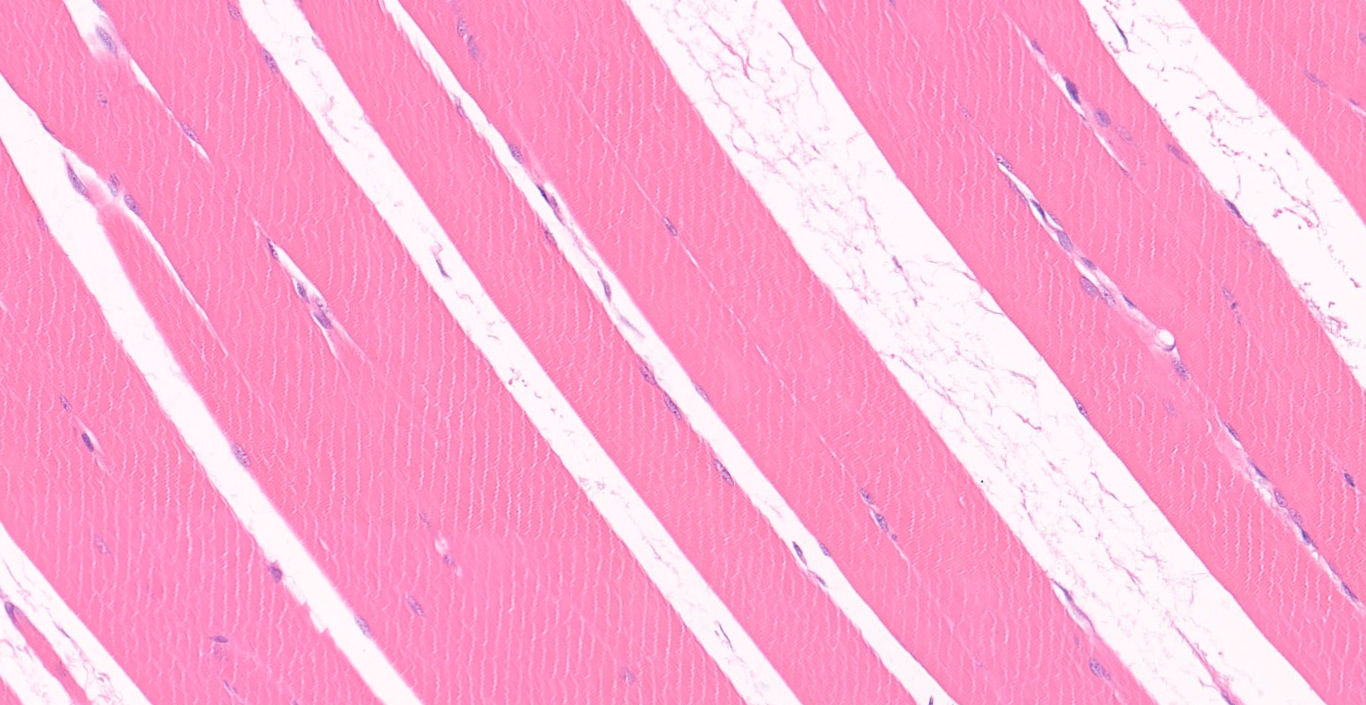

Supplement: Supplementary file 4 [file DataSheet2.ZIP › Sham/6-2.jpg]

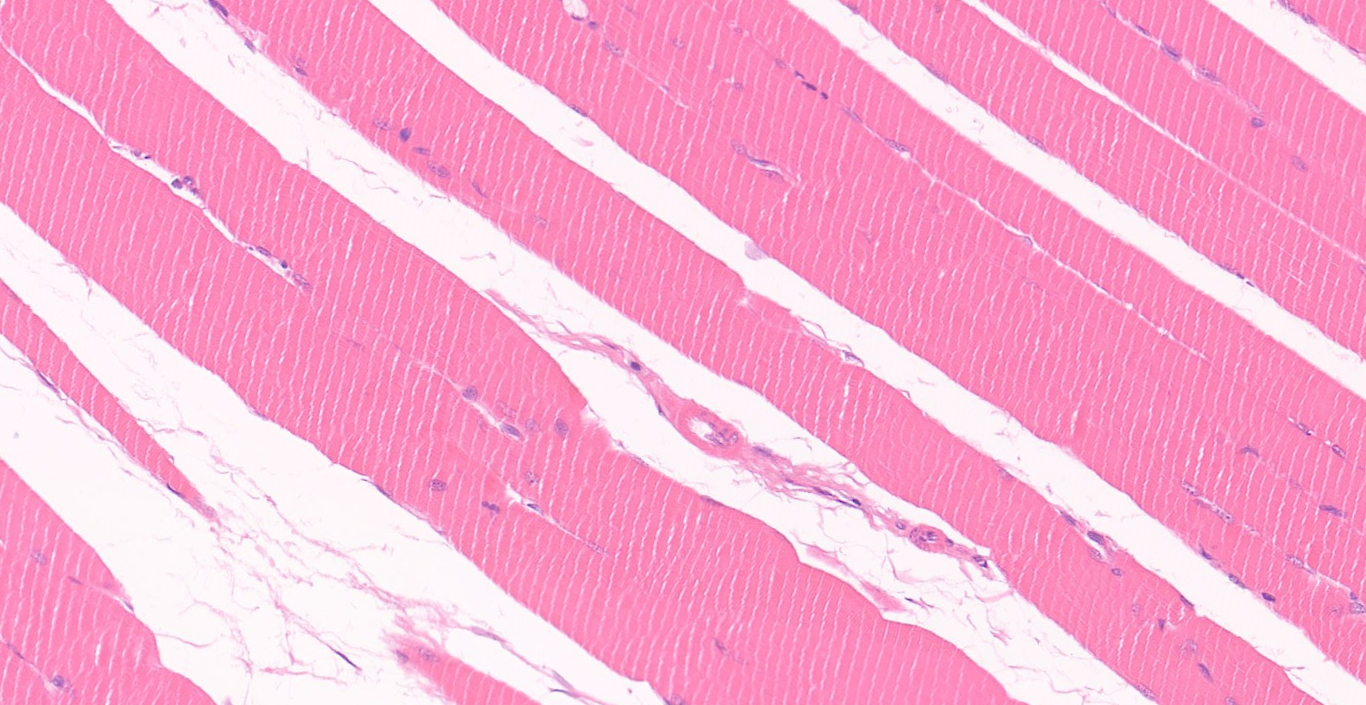

Supplement: Supplementary file 4 [file DataSheet2.ZIP › Sham/6-3.jpg]
